# Expertly Validated Models and Phylogenetically-Controlled Analysis Suggests Responses to Climate Change Are Related to Species Traits in the Order Lagomorpha

**DOI:** 10.1371/journal.pone.0122267

**Published:** 2015-04-15

**Authors:** Katie Leach, Ruth Kelly, Alison Cameron, W. Ian Montgomery, Neil Reid

**Affiliations:** 1 *Quercus*, School of Biological Sciences, Queen’s University Belfast, Belfast, Northern Ireland; 2 School of Biological Sciences, Queen’s University Belfast, Belfast, Northern Ireland; 3 Institute for Global Food Security (IGFS), Queen’s University Belfast, Belfast, Northern Ireland; University of York, UNITED KINGDOM

## Abstract

Climate change during the past five decades has impacted significantly on natural ecosystems, and the rate of current climate change is of great concern among conservation biologists. Species Distribution Models (SDMs) have been used widely to project changes in species’ bioclimatic envelopes under future climate scenarios. Here, we aimed to advance this technique by assessing future changes in the bioclimatic envelopes of an entire mammalian order, the Lagomorpha, using a novel framework for model validation based jointly on subjective expert evaluation and objective model evaluation statistics. SDMs were built using climatic, topographical, and habitat variables for all 87 lagomorph species under past and current climate scenarios. Expert evaluation and Kappa values were used to validate past and current models and only those deemed ‘modellable’ within our framework were projected under future climate scenarios (58 species). Phylogenetically-controlled regressions were used to test whether species traits correlated with predicted responses to climate change. Climate change is likely to impact more than two-thirds of lagomorph species, with leporids (rabbits, hares, and jackrabbits) likely to undertake poleward shifts with little overall change in range extent, whilst pikas are likely to show extreme shifts to higher altitudes associated with marked range declines, including the likely extinction of Kozlov’s Pika (*Ochotona koslowi*). Smaller-bodied species were more likely to exhibit range contractions and elevational increases, but showing little poleward movement, and fecund species were more likely to shift latitudinally and elevationally. Our results suggest that species traits may be important indicators of future climate change and we believe multi-species approaches, as demonstrated here, are likely to lead to more effective mitigation measures and conservation management. We strongly advocate studies minimising data gaps in our knowledge of the Order, specifically collecting more specimens for biodiversity archives and targeting data deficient geographic regions.

## Introduction

Changes in climate are predicted to have strong influences on the ecology and distribution of species [1], [2], with pronounced impacts on terrestrial biodiversity [3]. Although the climate changes naturally (and usually slowly), the rate of recent anthropogenically-induced change [4] is causing concern amongst conservation biologists [5]. Future climate change may have large effects on species niches i.e. the biotic and abiotic conditions in which a species can persist [6]. Species are predicted to adapt their bioclimatic niche, migrate to maintain their current niche, or become range restricted and undergo population decline, local or global extinction under future scenarios [7].

The Lagomorpha are an important mammalian order economically and scientifically as a major human food resource, model laboratory animals, valued game species, pests of agricultural significance and key elements in food webs providing scientific insights into entire trophic systems. Lagomorphs are native on all continents except Antarctica, occurring from sea level to >5,000m and from the equator to 80°N spanning a huge range of environmental conditions, and also include some very successful invasive species [8].

The taxonomy of the Lagomorpha in recent decades has been in a state of flux but all species belong to two families: the Ochotonidae and the Leporidae. The Ochotonidae consists of a monotypic genus, *Ochotona*, containing 25 species of small, social, high-latitude, and usually high-altitude, pikas. The Leporidae has 32 species of large, solitary, cursorial hares and jackrabbits in a single genus *Lepus* and 30 species of medium-sized, semi-social, fossorial rabbits comprising ten genera [8]. A quarter of lagomorphs are listed in the IUCN Red List of Threatened Species (www.iucnredlist.org) with a notable number of highly range-restricted species including fourteen listed under the IUCN Criteria B, with an extent of occurrence estimated to be less than 20,000 km^2^. In addition, pikas as high-altitude specialists with very high body temperatures of 39.3–41.0°C [9] are extremely susceptible to changes in their environment, particularly ambient temperatures [10].

Species Distribution Models (SDMs) are widely used in ecology and relate species occurrences at known locations to environmental variables to produce models of environmental suitability, which can be spatially or temporally extrapolated to unsurveyed areas and into past or future conditions e.g. [11]. Although SDMs have been highly influential in the field of ecology, their limitations have been widely reviewed e.g. [12]. The impact of climate change on species distributions has been modelled in a wide range of studies and a number of responses have been described e.g. [1], [5], [13]. Mammalian distributional changes have been well studied over the past decade and indicate that future climate change will have profound impacts e.g. [5], [14], [15], [16]. Mammals in the Western hemisphere are unlikely to keep pace with climate change, with 87% expected to undergo range contractions [16], and mammals in Mediterranean regions, particularly endemic species, are predicted to be severely threatened by future climate change [15]; shrews are especially vulnerable to future changes [5], [15]. These distributional responses have also been noted in studies of past climatic changes, for example, Moritz *et al.* [14] found that from the early 20^th^ century to the present, small mammals in a North American national park substantially shifted their elevational range upwards corresponding to ∼3°C increase in minimum temperatures. To date, very few SDM studies have included lagomorphs, but the American pika (*Ochotona princeps*) has been studied in detail and is predicted to decline in range [17] and shift upslope [18] in response to future climate. The European rabbit (*Oryctolagus cuniculus*) and mountain hare (*Lepus timidus*) have also been studied but only in a subset of their range in Australia [19] and Great Britain [20].

The predicted impact of climate change on species distributions has only rarely been linked with species traits. Yet, species traits are widely accepted as potentially important indicators of responses to climate change and identifying such traits may be crucial for future conservation planning e.g. [14], [21], [22]. Traits that directly impact climatic conditions experienced by a species, for example, their activity cycle, are likely to be more important in mediating species responses to projected climate change than traits such as diet breadth. If species can broaden their occupied bioclimatic niche through trait plasticity, for example, altering their diel patterns of activity, then they may be less susceptible to future change [23]. Mammalian species active during certain times of the day will experience a limited range of climatic conditions, whereas more flexible species can select the conditions in which they are active [24], and therefore, may be less susceptible to future change [23]. Small body size, nocturnal behaviour and burrowing may have allowed mammalian species to ‘shelter’ from climatic changes during the beginning of the Cenozoic era, following the Cretaceous-Tertiary (K-T) mass extinction event [25]. Burrowing rabbits and pikas may be able to avoid the impacts of climate change by seeking underground refuge from extreme or fluctuating temperatures [26] and inhabiting thermally buffered habitats [27], whilst larger cursorial species, such as the hares and jackrabbits which, in the majority of cases, live above ground may have less variability in microclimate opportunities within which to shelter [28], but they may exhibit greater adaptation to prevailing climate, for example, changing pelage colour [29] and long extremities (e.g. the pinna of the ear) in desert environments [30]. Dispersal is also likely to be very important in future species distributions, especially in regions predicted to have higher climate velocities where species will require greater dispersal rates to track climatic changes. Larger species are likely to be more mobile, and, hence better prepared to track climatic change [16].

Past studies have modelled the response of large numbers of species to predicted climate change [3] or dealt with a few key species from a range of orders [16]. Lagomorphs due to their restricted diversity provide an opportunity to rigorously examine the response of every species yielding a detailed picture of change within an entire order for the first time. Crucially, the small number of lagomorph species, compared to other mammalian groups, means that datasets can be verified in detail, modelled individually and outputs expertly validated. Moreover, lagomorphs have a nearly global terrestrial distribution and occupy a wide range of biomes providing an opportunity to examine the response of similar species from tundra to desert and islands to mountain summits.

Here, we assess the projected change in the bioclimatic envelopes of all ‘modellable’ lagomorph species under future climate change using a framework for model validation based jointly on subjective expert evaluation cross-validated with objective model evaluation statistics. We predict lagomorph species distributions will increase in elevation and poleward movement under future climate change, but with significant differences between pikas, rabbits, hares and jackrabbits due to dissimilarities in species traits, for example, body size. Lagomorph morphological and life history traits are correlated with the predicted responses to future climate change in order to test this hypothesis. We posit that flexibility in activity cycle and larger body sizes, which may lead to greater mobility, will result in species being less vulnerable to future climatic changes and better able to track climate niches.

## Materials and Methods

### Species data

A total of 139,686 records including all 87 lagomorph species were either downloaded from the Global Biodiversity Information Facility (GBIF) Data Portal (data.gbif.org), collated from species experts or members of the IUCN Species Survival Commission (SSC) Lagomorph Specialist Group (LSG) and/or extracted from the literature for data deficient species as advised by experts. All past and current occurrence records, sorted by species, can be viewed on http://lagomorphclimatechange.wordpress.com/. Taxonomic accuracy was dealt with by checking all records against the latest IUCN taxonomy; if names did not match after cross-referencing with taxonomic synonyms and previous names they were rejected. Spatial data accuracy was dealt with by removing any obviously erroneous records for the target species if they fell outside the extent of the IUCN geographic range polygon. In addition, occurrences recorded with a spatial resolution of >2km were removed and duplicate records were eliminated unless they were recorded in different temporal periods (pre-1950 and post-1950). This left 41,874 records of which 3,207 were pre-1950 and 38,667 were post-1950. Spatial and temporal bias in sampling was eliminated by only selecting the background data (a random sample of 10,000 points from the environmental layers describing pseudo-absences) from sites at which any lagomorph species had been recorded and ensuring there were the same proportion of pre-1950 to post-1950 background points as there were pre-1950 to post-1950 species records.

### Environmental parameterisation

Empirical climate data for past, current and future scenarios were downloaded from the R-GIS Data Portal (http://r-gis.org/), WorldClim (http://www.worldclim.org/) and CCAFS GCM Data Portal (http://www.ccafs-climate.org/data/) respectively at 30 arc-second resolution (≈1km grid cells). Records from pre-1950 were associated with mean climate data from 1900–1949 and those post-1950 were associated with mean data from 1950–2000. Projected future climate data were obtained from the IPCC 4^th^ Assessment Report using the A2 emissions scenario. Data from the A2 future climate change scenario was used because although it was originally described as “extreme climate change” it now appears to best represent the trend in observed climate. Eight environmental variables were used in their raw format and seven composite variables were calculated (see S1 Supporting Information).

### Species distribution modelling

SDMs were run using MaxEnt version 3.3.3k [11], [31]. Models were built using two different sets of input data: i) pre- and post-1950 data, or ii) post-1950 data only. The ‘samples with data’ (SWD) input format in MaxEnt was used for data entry, pairing pre-1950 records with mean climatic variables from 1900–1949 and post-1950 records with mean data from 1950–2000. The models were validated using either 10 replicate bootstrapping for species with low numbers of records (<30) or 4-fold cross-validation for species with high numbers of records (≥30). More than 30 records were needed for 4 fold cross-validation as this equates to ∼8 points per replicate, which has been deemed adequate for modelling some species distributions [32], bootstrapping was used with <30 records as this maximised the points used for both training and testing. Ten bootstrap replicates were needed to minimise computational power and maximise accuracy. Linear, quadratic and product feature types were used. The 10 percentile training presence threshold was applied to define likely presence and absence of each species.

Records for each species were associated with global land cover data downloaded from the ESA GlobCover 2009 Project (http://due.esrin.esa.int/globcover/) and any land class not occupied by the target species was marked as unsuitable. Model outputs restricted to suitable land classes, i.e. climate and habitat model, and unrestricted climate-only models will be evaluated by experts. A minimum convex polygon of the species occurrence records, buffered by a dispersal value specific to each species which was verified by experts from the IUCN Species Survival Commission (SSC) Lagomorph Specialist Group (LSG), was used to remove areas of over-prediction. This meant that the suitable area within reach of each species was predicted, and led to more conservative outputs than just simply predicting suitable area. Although this assumes occurrence records are complete, this was a reasonable way to correct for potential biogeographic over-prediction and a similar method was advocated by Kremen *et al.* [33]. Annual dispersal distances were elicited from each species expert during the model evaluation procedure (see each species account in S2 Supporting Information). Dispersal distances were either observed by the lagomorph experts (personal observations), documented in the literature, or for species where no data were available, the average distance was calculated from the specific group to which it belonged (e.g. Asian pikas or African hares).

Non-native ranges for the only three invasive lagomorphs, European hare (*Lepus europaeus*), Eastern cottontail (*Sylvilagus floridanus*) and European rabbit (*Oryctolagus cuniculus*), were not modelled because invasive species are not at equilibrium with the environment and their niches cannot be transferred in space and time [34]. Mountain hare (*Lepus timidus*) populations in Ireland and mainland Eurasia were modelled separately due to the distinct morphological, phenotypic, behavioural, ecological and genetic differences between the Irish sub-species (*L. t. hibernicus*) and other mountain hares e.g. [35], but the outputs for each geographic region were subsequently combined to produce a single model reflecting the current classification of the Irish hare as an endemic sub-species.

### Model evaluation

A bespoke website (lagomorphclimatechange.wordpress.com) was created to allow each species expert to review the output of their allocated species. Expert evaluation, whereby an acknowledged expert on each species judges model predictions for current and past distributions, can be a useful tool prior to making future extrapolations [36]. A framework combining expert evaluation with reliable model evaluation metrics allows species distribution models to be assessed before they are used in future projections to infer likely future changes in distribution.

Forty-six lagomorph experts, including 20 members of the IUCN Species Survival Commission (SSC) Lagomorph Specialist Group (LSG) and 26 recognised lagomorph researchers selected on recent publications, were paired to species (see Table A in S3 Supporting Information) and asked to assess whether model projections accurately, roughly or did not capture the current and past range of each species i.e. good, medium or poor respectively according to the criteria in Anderson *et al.* [36]. Experts were asked to select the most accurate representation of the current and past range from the following models: i) pre- and post-1950 input data showing the suitable bioclimatic envelope, ii) pre- and post-1950 input data showing the suitable bioclimatic envelope restricted to suitable habitat, iii) post-1950 input data only showing the suitable bioclimatic envelope, or iv) post-1950 input data only showing the suitable bioclimatic envelope restricted to suitable habitat.

Independent model evaluation in SDM studies often uses the Area Under the Curve (AUC) value but has been heavily criticised [37]. AUC is not advocated for model evaluation because Receiver Operating Characteristic (ROC) curves cannot be built for presence/absence or presence/pseudo-absence data [38] and AUC values can be influenced by the extent of model predictions [37]. There are also known limitations with using alternative metrics [38] such as sensitivity (proportion of presences which are correctly predicted), specificity (proportion of absences which are correctly predicted) or True Skill Statistic (a prevalence independent metric calculated using sensitivity and specificity). Misleading commission errors, which can arise from species not being at equilibrium with the environment, can affect such metrics. On the other hand, Kappa is an objective measure of prediction accuracy based on input species records and background points adjusted for the proportion of correct predictions expected by random chance [39]. Kappa utilises commission and omission errors [40], although it does not take into account prevalence like the True Skill Statistic [38], and can sometimes produce misleading commission errors [41], it has widely accepted thresholds which can be useful in model evaluation [42], [43], [44]. There are documented flaws with all possible model evaluation statistics. However, Kappa is now commonly used in model evaluation e.g. [45] and here has been paired with an expert validation approach.

The ‘accuracy’ function in the SDMTools package [46] in R (version 3.0.2) was used to calculate the Area under the Curve (AUC) value, omission rate, sensitivity, specificity, proportion correct, Kappa and True Skill Statistic for completeness, but only Kappa was taken forward for use in the model validation framework. The optimum threshold was taken as Kappa >0.4 as this value has been advocated in a range of previous studies [38], [42], [44]. Models that had Kappa >0.4 and those that were ranked as either good or medium by expert evaluation were defined as “modellable” because they demonstrate good model fit and predictive ability; these species were carried forward for projection and further analysis. Those models with a Kappa <0.4 or ranked as poor by expert evaluation were defined as “unmodellable”, with poor model fit and predictive ability, and were rejected from further analysis (see Fig. A in S3 Supporting Information). Hereafter, species are referred to as “modellable” or “unmodellable” as explicitly defined above.

### Future predictions

The model settings, for example, the input data used (pre- or post-1950) or restriction/no restriction to occupied land classes, which provided the optimal outcome i.e. the highest Kappa value and best expert evaluation were used to project modellable species bioclimatic envelopes under future climate during the 2020s, 2050s and 2080s. Future predictions were clipped to the buffered minimum convex polygon of the target species further buffered by the dispersal distance (kilometres/year) of each species multiplied by the number of years elapsed from the present (1950–2000) taken as 1975. Predicted range size, mean latitude and minimum, mean and maximum elevation for each species and each time period were calculated. Model outputs for each species can be found in S2 Supporting Information; unmodellable species projections are included for reference only.

### Species traits

Species trait data were downloaded from the PanTHERIA database [47] and updated by searching the literature. Correlated traits were removed to reduce multicollinearity (i.e. tolerance <2 and VIF >5) and the final set of traits used to describe each modellable species were: activity cycle (nocturnal only, diurnal only, flexible), adult body mass (g), diet breadth (number of dietary categories eaten from: vertebrates, invertebrates, fruit, flowers/nectar/pollen, leaves/branches/bark, seeds, grass and roots/tubers), gestation length (days), habitat breadth (above ground-dwelling, aquatic, fossorial and ground-dwelling), home range (km^2^), litters per year, litter size, population density (*n*/km^2^) and age at sexual maturity in days (Fig. B in S3 Supporting Information).

### Statistical analysis

To illustrate projected changes in the distribution of lagomorph species, the difference in predicted species richness per cell was calculated between the 1930s (1900–1949) and 2080s (2070–2099). The difference in model output metrics (range size, mean latitude and minimum, mean and maximum elevation) was calculated between the 1930s and 2080s. Change in range size was expressed in percentage change but change in latitude was represented as degree movement towards the poles and change in elevation in metres. Generalised Least Square (GLS) models, with an autoregressive moving average (ARMA) correlation structure, in the ‘nlme’ package in R version 3.0.2 [48], were used to test the differences in temporal trends for range change, poleward movement and elevation between lagomorph groups: 1) pikas, 2) rabbits and 3) hares and jackrabbits. ARMA was used to explicitly account for the non-independent nature of the time-series periods. Phylogenetic Generalized Least-Squares (PGLS) regressions were performed to test whether changes in model predictions varied with morphological or life history traits. PGLS analysis was run using the ‘caper’ package in R [49] to fit a linear model which takes into account phylogenetic non-independence between data points. The implementation of this method is described in Freckleton *et al.* [48]. A lagomorph phylogeny was extracted from the mammalian supertree provided by Fritz *et al.* [50]. Likely clade membership for five species not included in this phylogeny was determined from Ge *et al.* [51], and then missing tips were grafted on using an expanded tree approach [52]—see Fig. C in S3 Supporting Information. Outliers were removed prior to analysis, because species with large residuals may overly influence the results of the regression, and they were identified as those with a studentized residual >3 units following Cooper *et al.* [53]. All models exhibited normally distributed residuals tested using Shapiro-Wilk. The scaling parameter lambda varies from 0, where traits are independent of phylogeny, to 1 where species’ traits covary in direct proportion to their shared evolutionary history [54]. We estimated lambda for each model and tested whether it was significantly different from 0 or 1 during the PGLS analysis. All subset regressions were run using the ‘dredge’ function in the ‘MuMIn’ [55] package in R, using AICc as the rank estimate, and then model averaging was used to describe the effect of each variable.

## Results

**Fig 1:**
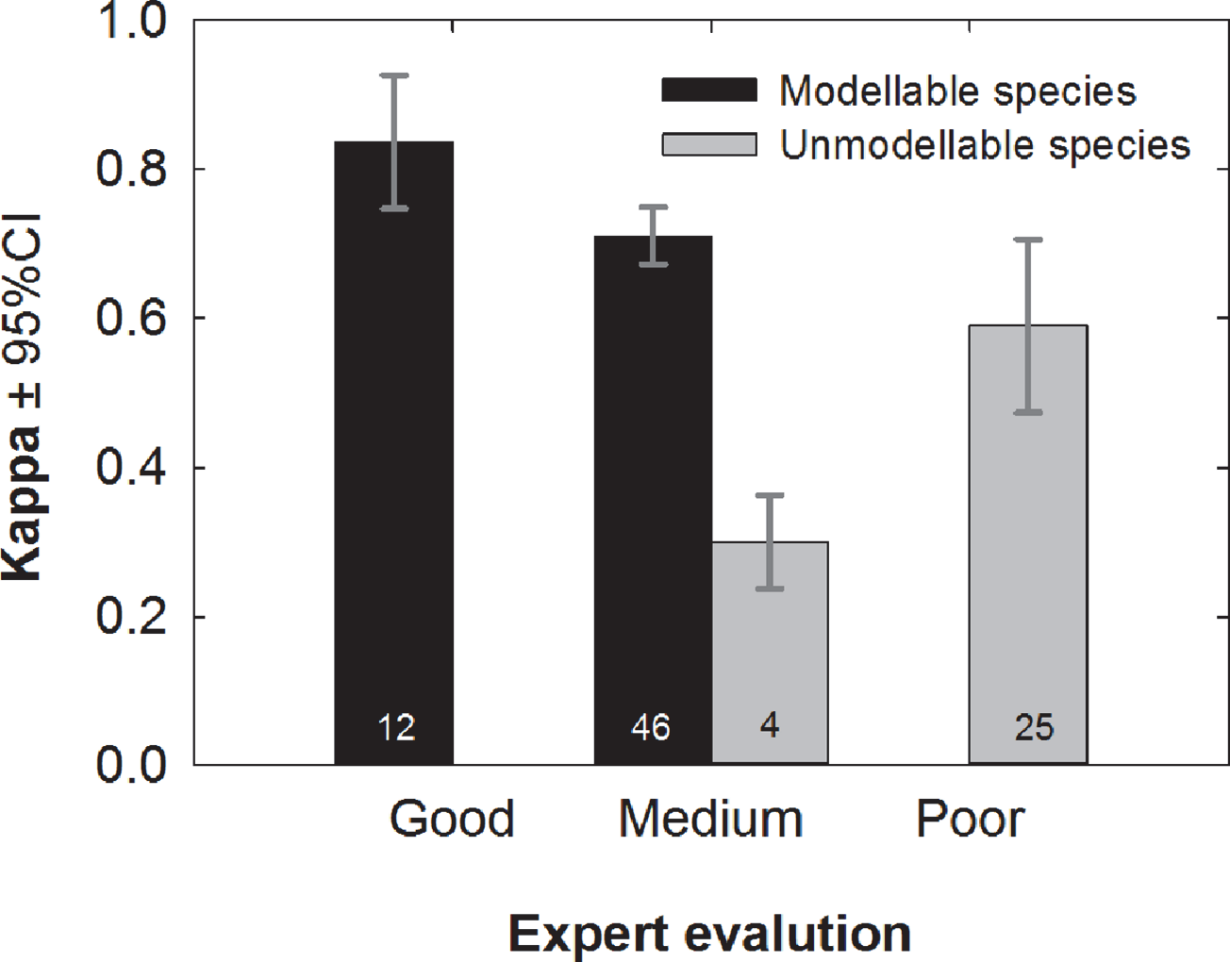
Agreement between expert evaluation and model accuracy. Mean Kappa ±95% confidence intervals within the categories assigned by expert evaluation. Black bars indicate species that were deemed “modellable” and retained for further analyses, whereas grey bars indicate “unmodellable species” that were rejected. Sample sizes (i.e. numbers of species) are shown in the bars.

There was a high degree of agreement between expert model classification and Kappa values. However, experts were often more critical; for example, they classified 25 species models as ‘poor’ but these species had a mean Kappa of ∼0.6 (Fig. 1). Fifty-eight species (67%) were deemed modellable with expert validation classed as medium or good and Kappa >0.4 and 29 species (33%) were rejected as unmodellable with expert validation classed as poor and/or Kappa <0.4. Unmodellable species were 4 times more likely to be listed by the IUCN as Data Deficient than modellable species, with 8 unmodellable species (28%) listed as threatened. The majority of species with small sample sizes were classed as unmodellable; the median number of occurrence points for modellable species was 36 and for unmodellable 13. Hereafter, all results are for modellable species only, and, therefore, are a highly conservative estimate of the impact of climate change on the order Lagomorpha as a whole.

**Fig 2:**
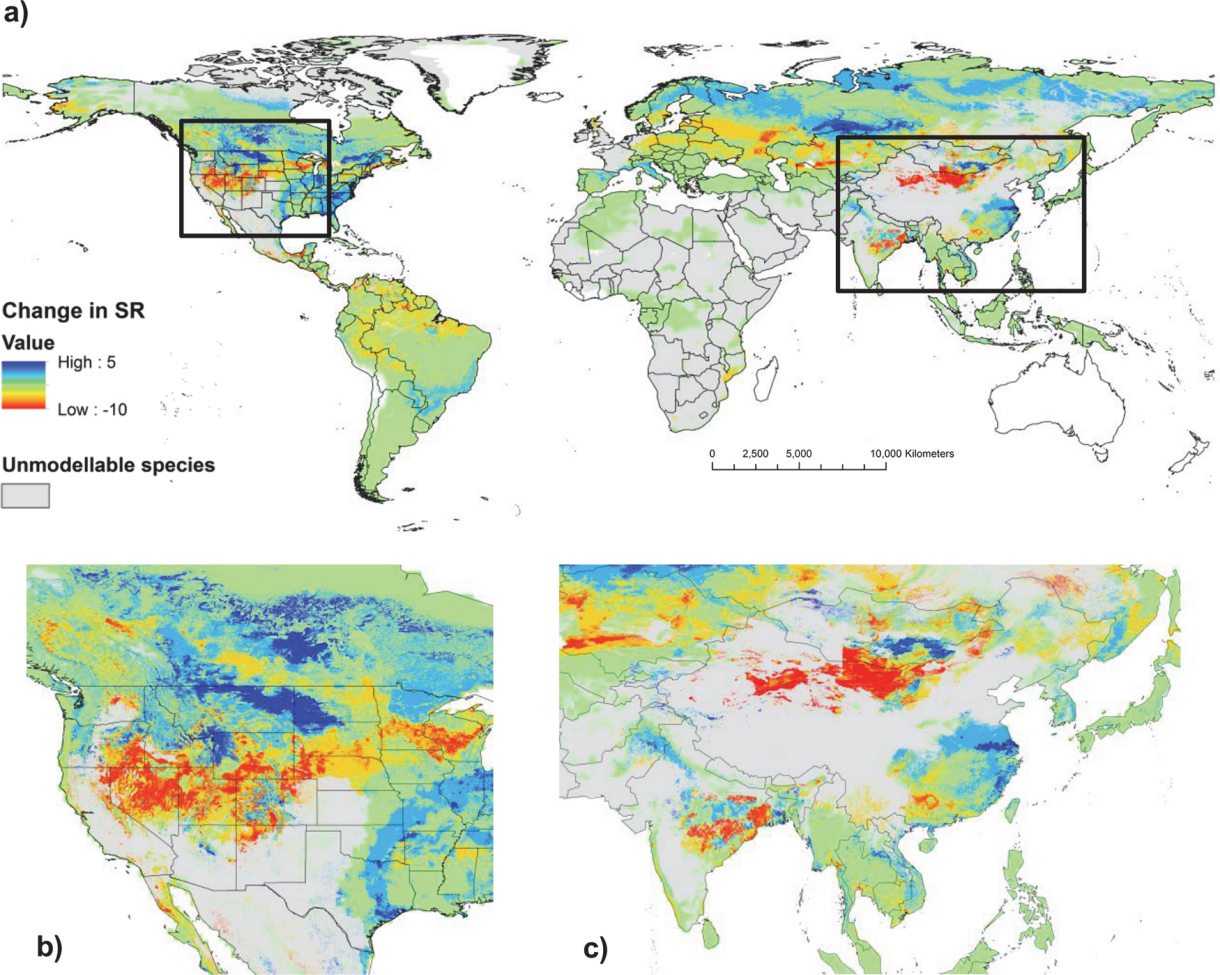
Change in predicted lagomorph species richness from the 1930s to 2080s. (**a**) Global patterns in predicted species loss and gain showing details in (**b**) North America and (**c**) Asia. Light grey indicates areas occupied by “unmodellable” species with uncertain outcomes.

Global changes in predicted lagomorph species richness suggest that almost a third of the Earth’s terrestrial surface (31.5 million km^2^) currently occupied by lagomorphs is predicted to experience loss of lagomorph species by the 2080s (Fig. 2, also see Fig. D in S3 Supporting Information). Areas along the northern border of China become increasingly unsuitable, potentially losing up to 10 species by 2080, including the woolly hare (*Lepus oiostolus*) and Glover’s pika (*Ochotona gloveri*) which are predicted to undergo dramatic movements to higher elevations. In contrast, predicted species gains were notable across: (a) northern Eurasia, due to poleward movement of the mountain hare (*Lepus timidus*); and, (b) North America, where some regions e.g. the Upper Missouri catchment of Montana and North Dakota were predicted to gain up to 5 species. This includes the desert and eastern cottontails (*Sylvilagus audubonii* and *S. floridanus* respectively) which are predicted to exhibit strong poleward movements. The majority of African lagomorph species were classed as unmodellable and as such Fig. 2 is largely data deficient for the continent.

**Fig 3:**
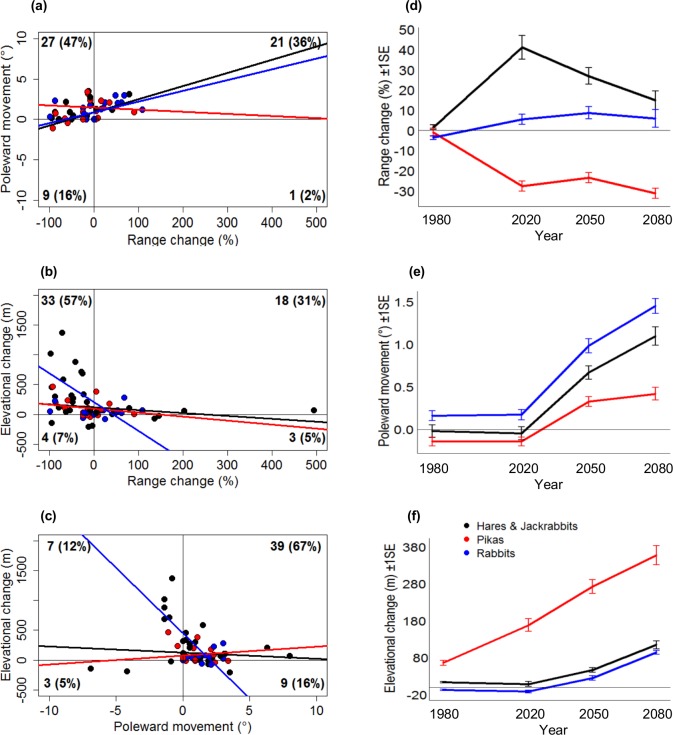
Characterisation of predicted lagomorph bioclimatic envelope change. Scatterplots show the linear relationship between range change (%) and (**a**) poleward movement (°), (**b**) elevational change (m) and (**c**) poleward movement and elevational change. The numbers of species in each quadrant that exhibited positive or negative change on each axis are shown with percentages in parentheses. Temporal trends for (**d**) range change, (**e**) poleward movement and (**f**) elevational change ± 1 standard error for each species group; pikas (red), rabbits (blue) and hares and jackrabbits (black).

Thirty-six lagomorph species are predicted to experience range loss (63%), 48 poleward movements (83%) and 51 elevational increases (88%). However, 22 (38%) species are predicted to increase their range, 7 (12%) decrease in elevation and 10 (17%) move away from the poles due to future climatic changes. Thirty-five species (60%) are predicted to undergo range declines *and* either poleward movements *or* elevational increases. On average, all three groups of lagomorphs exhibited significant poleward shifts (*F*_df = 1,234_ = 13.5, *p*<0.001) and elevational increases (*F*_df = 1,234_ = 44.2, *p*<0.001) by 2080 (Fig. 3, also see Table B in S3 Supporting Information). The average poleward shift for the order was 1.1° and elevational shift 165m. Pikas exhibited the most substantial mean increase in elevation becoming increasingly isolated on mountain summits (e.g. the Rockies in North America and the Tibetan Plateau and high Himalayas) resulting in a significant 31% range contraction (*F*_df = 1,234_ = 3.7, *p* = 0.03), but showed little poleward movement. In addition, lagomorph species occupying islands (*n* = 6) will, on average, lose 8km^2^ of their ranges compared to 0.2km^2^ gained by continental species (*n* = 52), whilst mountain dwelling species (*n* = 24) will lose 37km^2^ of their ranges compared to 25km^2^ gained by lowland species (*n* = 34).

**Fig 4:**
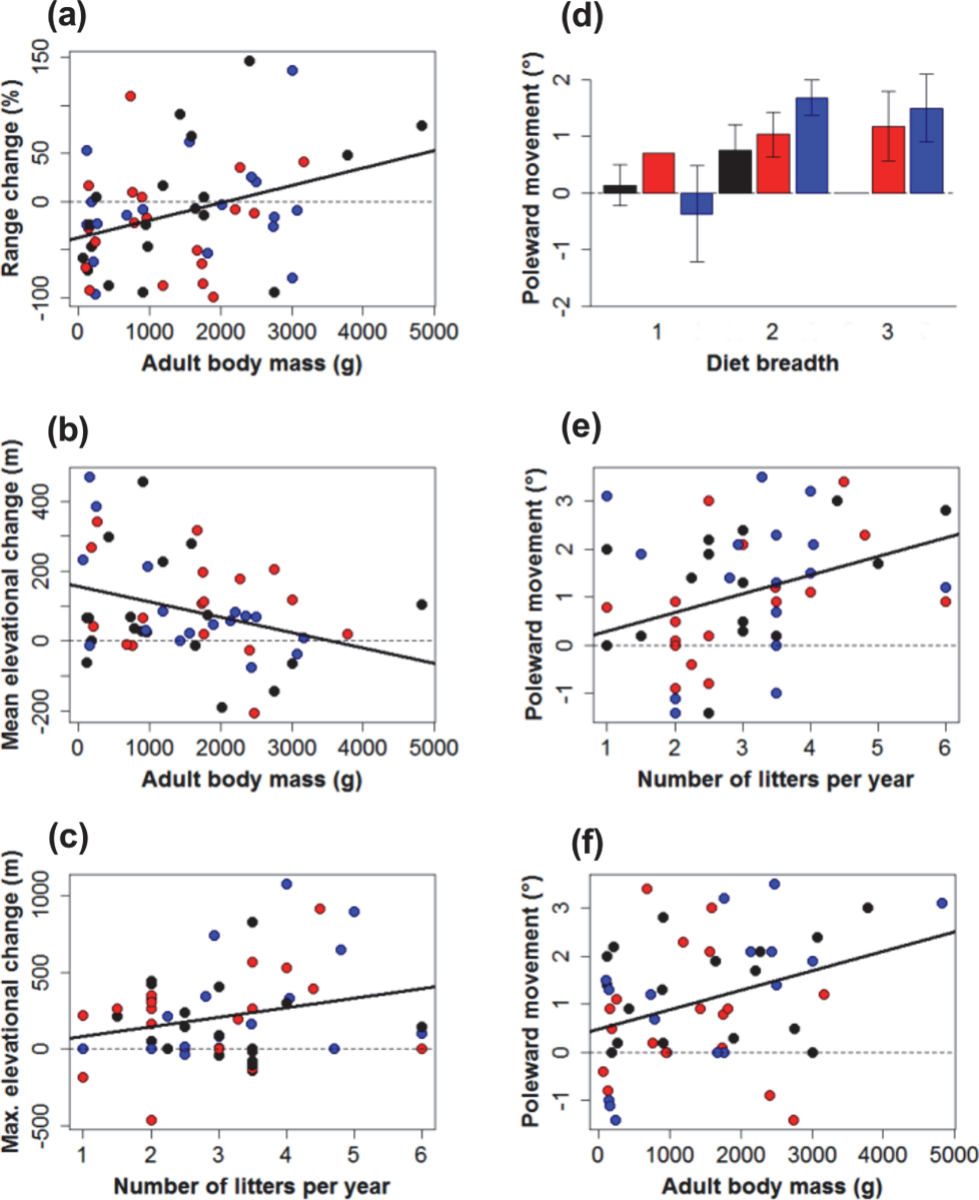
Relationships between species traits and responses to future climate change. The ability of species’ traits to predict changes in (**a**) range (**b**) mean elevation (**c**) maximum elevation and (**d**) (**e**) (**f**) poleward movement under future climate (between ∼1930s and ∼2080s) for each group; pikas (red), rabbits (blue) and hares and jackrabbits (black). Diet breadth is a categorical variable and is therefore represented as a bar plot (±1 standard error) with sample sizes (i.e. numbers of species) shown above the bars. Only significant predictors of change are shown here. The dashed line at zero indicates no change in the response variable.

PGLS models indicate that members of each group were capable of showing a variety of responses i.e. species of pika, rabbits, hares or jackrabbits exhibited both increases and decreases in each response variable (Fig. 4). All traits used in the PGLS models are described in the methods and full results can be found in Table C in S3 Supporting Information. Here, we present only the significant results. There was a significant positive relationship between predicted range change and adult body mass (*β* = 0.258, *F*_df = 4,52_ = 2.308, *p* = 0.021) with hares and jackrabbits generally increasing their range by 2080 and pikas exhibiting range contraction (Fig. 4; also see Table C in S3 Supporting Information). Adult body mass was positively associated with predicted poleward movement (*β* = 0.196, *F*_df = 5,49_ = 1.989, *p* = 0.047) and inversely related to predicted elevational change (*β* = -0.183, *F*_df = 2,50_ = 2.019, *p* = 0.043). The average adult body mass for lagomorph species living in mountainous regions was 836g, compared to 1.8kg in lowland areas. The number of litters a species produces per year was positively related to predicted latitudinal and maximum elevational shifts (*β* = 0.215, *F*_df = 5,49_ = 2.731, *p* = 0.006 and *β* = 0.160, *F*_df = 2,53_ = 1.746, *p* = 0.081 respectively) with more fecund species being capable of more extreme upward movement or poleward shifts. There was also a positive relationship between species dietary niche and the degree of predicted poleward shift (*β* = 0.181, *F*_df = 5, 49_ = 2.190, *p* = 0.029). No significant relationships were found between activity cycle and predicted changes in species distribution.

## Discussion

The Lagomorpha as a whole are predicted to exhibit much greater poleward (mean ± 95%CI = 1.1° ± 0.5°) and elevational shifts (165m ± 73m) by 2100 than calculated in a recent meta-analysis collating information on a wide variety of taxonomic groups [1]. On average birds, butterflies and alpine herbs were predicted to shift ∼0.8° poleward and increase in elevation by ∼90m by 2100. Thuiller *et al.* [5] found that mammals were less vulnerable to change than other groups, but more than 50% of shrews could lose more than 30% of their suitable climatic space. In comparison, our study shows that only 34% of modellable lagomorphs will lose more than 30% of their suitable climatic space, but lagomorphs appear to show more notable changes in elevation and poleward movement. A study by Schloss *et al.* [16] found that, in some scenarios, 50% of mammal species in North and South America will be unable to keep pace with future climate change. Our results indicate that lagomorphs follow similar trends to other mammalian climate change studies, but with more substantial poleward and elevational shifts. Furthermore, our results are conservative estimates due to the exclusion of unmodellable species, most notably African species. Parts of Africa are expected to become drier and warmer under future climate, with substantial increases in arid land [4] which will likely lead to negative consequences for lagomorphs not assessed here. Our results are also conservative due to the exclusion of most Data Deficient lagomorph species (as classified by the IUCN) which are highly likely to be threatened with extinction [56]. We strongly advocate studies minimising data gaps in our knowledge of the Order, specifically collecting more specimens for biodiversity archives and targeting data deficient geographic regions.

In contrast, a recent study of empirical climate studies by McCain & King [23] has shown that only about 50% of, mostly North American, mammal species respond to climate change by shifting in latitude and elevation. Results of four lagomorph studies are included which show that the pygmy rabbit (*Brachylagus idahoensis*) undergoes extirpation and contraction due to climatic changes [57], the ranges of the snowshoe hare (*Lepus americanus*) and collared pika (*Ochotona collaris*) do not change [58], [59] and the American pika mostly undergoes extirpation and upslope contraction, but some sites within the range show no change [14], [60]. Although we find that more than 50% of lagomorphs respond to climate change, our results are largely congruent with these empirical studies, indicating range contraction in the American pika and pygmy rabbit, and little change in the ranges of the snowshoe hare and collared pika.

Pikas are predicted to show elevational rather than latitudinal shifts because these high-altitude specialists are known to be extremely susceptible to increases in temperature or unpredictable seasonality, which could potentially lead to heat stress [9]. On the other hand leporids, typically being lowland species, exhibited less substantial increases in elevation but greater poleward shifts, for example, the mountain hare (*Lepus timidus*). This is probably due to the high sensitivity of its boreo-alpine niche to changes in temperature [61], [62]. Indeed, even the Irish hare (*L. t. hibernicus*) which inhabits temperate grasslands, unlike other mountain hares, is predicted to experience a contraction from the south-east to the north-west of Ireland. As global temperatures increase, northern latitudes will become more climatically suitable for southern leporids and, therefore, species bioclimatic envelopes will track poleward to match. Rabbits, hares and jackrabbits were thus predicted to exhibit little overall change in the total extent of their ranges. The majority of the modellable rabbit species were from the genus *Sylvilagus* inhabiting south and eastern USA and Mexico; a region projected to become generally drier [63] inducing latitudinal shifts in species to track suitable habitats or vegetation. Thus, by shifting their ranges poleward, leporids are predicted to be able to maintain or increase their range extent suggesting they are considerably less sensitive to projected climate change than pikas.

PGLS models estimated lambda to be close to zero for changes in range, poleward movement and elevation shifts indicating that observed trends were independent of phylogeny [48]. Smaller species, principally pikas and some rabbits, were typically more likely to make elevational shifts in range whilst larger species, principally hares and jackrabbits, had a greater tendency for poleward shifts. This relationship has also been described in empirical climate studies e.g. [23] and is probably due to the large number of small-bodied mountainous species (*n* = 24) which have less opportunity to shift latitudinally because they are confined to high-elevation areas that contract upslope as climate changes. An elevational shift is almost always accompanied by a range decline, whereas a latitudinal shift could be accompanied by declines, increases or no change in range. Only when a species occupies high latitudes, edges of continents or islands will latitudinal shifts cause drastic range declines. A 1m elevational shift is equivalent to a 6km latitudinal shift [1], so it is easier for smaller species to shift altitudinally rather than latitudinally. This could be explained due to a relationship between adult body mass and dispersal distance, which was used to clip model projections, but a Spearman’s Rank correlation suggested no correlation between these two variables in our dataset (*r*s[30,2] = 0.077, *p* = 0.565). Trait analyses also showed a significant positive relationship between fecundity and extreme elevational or poleward movement which is comparable to a study by Moritz *et al.* [14], who found that fecund small mammals in Yosemite National Park, USA, were more likely to expand their ranges upward than less fecund counterparts. We found no association between activity cycle and vulnerability to climate change in the Lagomorpha as hypothesized and reported in McCain & King [23], but this may be due to the varied nature of lagomorph activity. The activity of the European hare is known to be less consistent and partially diurnal in the summer [64] and the American pika also shows seasonal changes in activity patterns [65]. However, we acknowledge the potential drawbacks of linking traits to modelled distributional changes rather than empirical-based studies, i.e. non-independence of trait results; nevertheless, the analysis presented here facilitates the understanding of the traits which could potentially lead to vulnerability to future climate change.

The uncertainties in SDM using projected future climate scenarios are well described [12]. Models are vulnerable to sampling bias [66], spatial scale issues [67], lack of data for rare species [32], uncertainty regarding future climate conditions [68] and insufficient independent evaluation [67]. We have tackled these explicitly by accounting for sampling bias by restricting the set of background points used, using data with the highest spatial resolution available (30 arc-second) and selecting species records to match, bootstrapping models for rare species with few records, averaging climatic data from five global circulation models and using a framework of joint expert and metric-driven model validation to segregate current distribution model outputs into those that were modellable and unmodellable before subsequent projection and analysis. However, our predictions could still be potentially confounded by species-area relationships [69] and biological interactions [70].

Regardless of individual model outcomes (see S2 Supporting Information), the overall trends observed across the order Lagomorpha as a whole are compelling. This study did not take account of shifts in habitats, vegetation or human impact in response to climate change, but we have shown that adaptation to future climate conditions may be possible as some species were predicted to exhibit poleward movements, with only modest shifts in range or elevation e.g. eastern and Appalachian (*Sylvilagus obscurus*) cottontails.

The predicted changes in climatic conditions are likely to have greater impacts on isolated lagomorph populations, i.e. those on islands and in high-altitude systems. If changes continue at the rate currently predicted until the 2080s, then there may be no climatically suitable range available for some montane or isolated species e.g. the Tres Marias cottontail (*Sylvilagus graysoni*) or black jackrabbit (*Lepus insularis*). Conservation strategies, such as assisted migration, could be the only option for these highly range-restricted species. Furthermore, conservation management will need to focus on small-bodied mammals as these are predicted to show more dramatic responses to changing climate. Small mammals are key in food webs sustaining predator populations, impacting plant communities by grazing and soil biology and hydrology by burrowing. Thus, fundamental shifts in lagomorphs globally may cause trophic cascade effects, especially in northern latitudes such as the cyclic systems of the Arctic.

The advancing knowledge of past extinction rates for the Lagomorpha [51], along with better bioclimatic envelope modelling, could aid the prediction and prevention of future extinctions. Our models suggest that Kozlov’s pika (*Ochotona koslowi*) may become extinct by the 2080s as the elevational increases required to maintain its current bioclimatic envelope disappear as it reaches the maximum elevation available. We have shown here how expert validation can be effectively integrated into the model evaluation process in order to improve model predictions and advocate use of this framework in future SDM studies. Assessment of vulnerability to climate change is needed urgently to identify how and to what extent species, taxa, communities and ecosystems are susceptible to future changes, taking into account likely changes to vegetation, human pressures and interspecific interactions, and to direct conservation management in an efficient and effective manner. Multi-species approaches are likely to lead to more effective mitigation measures and contribute to our understanding of the general principles underpinning the biogeographical and ecological consequences of climate change impacts.

## Supporting Information

S1 Supporting Information Supplementary Methods. (PDF)

S2 Supporting Information 89 x 1 page species accounts (including: species information, predicted changes in distributions, plots of change in range, latitude and elevation, model evaluation metrics and variable response curves). (PDF)

S3 Supporting Information Table A. Lagomorph experts, institutions and species evaluated. **Fig. A**. Framework for assessing whether species were “modellable” or “unmodellable” based on Kappa values and expert evaluation classification. **Fig. B**. Trait values for all three lagomorph groups (rabbits, hares & jackrabbits, and pikas). **Fig. C**. Full lagomorph phylogeny used in phylogenetically controlled regressions. **Fig. D**. Percentage change in predicted lagomorph species richness from the 1930s to 2080s. **Table B**. Results of generalised least square models characterising predicted lagomorph bioclimatic envelope changes. **Table C**. Results of phylogenetically-controlled generalised least square regressions.
(PDF)
