# Supplementary material for: Expertly Validated Models and Phylogenetically-Controlled Analysis Suggests Responses to Climate Change Are Related to Species Traits in the Order Lagomorpha
Source: PLoS One. 2015 Apr 15;10(4):e0122267. doi: 10.1371/journal.pone.0122267 (PMC4398435; doi:10.1371/journal.pone.0122267)
Supplement: S1 Supporting Information — (PDF) [file pone.0122267.s001.pdf]

S1 Supporting Information. Supplementary Methods.

Environmental parameterisation

Evapotranspiration was calculated using the Hargreaves equation [1]:

$$\text{Evapotranspiration} = 0.0023 \text{ x } (T_{mean} + 17.8) \text{ x } (T_{max} - T_{min})^{0.5} \text{ x } R_a$$

where,

$R_a = ((24 \text{ x } 60)/\pi) \text{ x } G_{sc} \text{ x } d_r [(\omega_s \text{ x } \sin\varphi \text{ x } \sin\delta) + (\cos\varphi \text{ x } \cos\delta \text{ x } \sin\omega_s)]) \text{ x } 0.408$
G_{sc} = solar constant = 0.0820 MJ m-2 min-1
d_r = inverse relative distance Earth-Sun = 1 + 0.033 cos ((2π/365) x J)
ω_s = sunset hour angle [rad] = arcos [-tan (φ) tan (δ)]
φ = latitude [radians] (grid file in decimal degrees converted to radians (multiply by π/180))
δ = solar decimation [rad] = 0.409 sin (((2π/365) x J) – 1.39)
J = number of days in the year. J at the middle of the month is approximately given by J = INTEGER (30.4 Month – 15) = 15 on average.

Annual evapotranspiration was taken as the sum of all monthly values. Annual water balance was calculated by subtracting annual evapotranspiration from mean annual precipitation. The number of months with a positive water balance was calculated by subtracting each monthly evapotranspiration from its corresponding monthly precipitation, and then converting these into a binary format, where a value greater than zero was given a value of one and a value less than zero was kept at zero [2]. The twelve binary files were then summed to calculate the number of months with a positive water balance.

Mean annual Normalised Difference Vegetation Index (NDVI) was calculated from monthly values which were downloaded from the EDIT Geoplatform (http://edit.csic.es/Soil-Vegetation-LandCover.html). NDVI is commonly used to measure the density of plant growth and is obtained from NOAA AVHRR satellite images. A negative value indicates snow or ice, a value around 0 indicates barren areas, values between 0.2 and 0.3 indicate grassland, and values near 1 indicate rainforests [3]. Human influence index was downloaded from the SEDAC website (http://sedac.ciesin.columbia.edu/data/set/wildareas-v2-human-influence-index-geographic). This was a composite of human population density, railways, roads, navigable rivers, coastlines, night-time lights, built-up areas and agricultural and urban land cover. Values within the index range from 0 to 64, where zero equalled no human influence and 64 represented maximum human influence [4]. Solar radiation was calculated using the Spatial Analyst function in ArcGIS 10.1 (ESRI, California, USA). Solar radiation is defined as the total amount of incoming solar insolation (direct and diffuse), or global radiation, and was measured in watt hours per square meter or WH/m^2 [5]. An index of surface roughness was also calculated by finding the difference between maximum and minimum gradient values, based on a global Digital Elevation Model at 30 arc-second resolution downloaded from WorldClim [6]. Altitude was not included as a variable

independently because organisms perceive climatic and habitat variables as proxies for altitude [7].

Future climate data

Pierce *et al.* [8] report that using data averaged across five global circulation models (GCMs) is substantially better than any one individual model and significantly reduces model error. We averaged all variables for the following five GCMs: CCCma-CGCM3.1/T47, CNRM-CM3, CSIRO-Mk3.0, HadCM3 and NASA-GISS-ER. Future projections adopted the time periods: 2020s (2010-2039), 2050s (2040-2069) and 2080s (2070-2099). Data from the A2 future climate change scenario was used because although it was originally described as "extreme climate change" it now appears to best represent the trend in observed climate.

REFERENCES

[1] Allen RG, Pereira LS, Raes D, Smith M. Crop evapotranspiration – guidelines for computing crop water requirements – FAO Irrigation and Drainage paper 56; 1998.

[2] Kremen C, Cameron A, Moilanen A, Phillips SJ, Thomas CD, Beentje H, et al. Aligning conservation priorities across taxa in Madagascar with high-resolution planning tools. Science. 2008; 320: 222–226.

[3] Holben BN. Characteristics of maximum-value composite images from temporal AVHRR data. Int. J. Remote Sens. 1986; 7: 1417-1434.

[4] Wildlife Conservation Society (WCS) and Centre for International Earth Science Information Network (CIESIN). Last of the Wild Project: Global Human Influence Index dataset (geographic), Palisades, NY: NASA Socio-Economic Data and Applications Centre (SEDAC); 2005.

[5] Fu P, Rich PM. The Solar Analyst 1.0 Manual, Helios Environmental Modelling Institute (HEMI), USA; 2000.

[6] Newton-Cross G, White PCL, Harris S. Modelling the distribution of badgers *Meles meles*: comparing predictions from field-based and remotely derived habitat data. Mamm. Rev. 2007; 37: 54-70.

[7] Austin MP. Spatial prediction of species distribution: an interface between ecological theory and statistical modelling. Ecol. Mod. 2002; 157: 101-118.

[8] Pierce DW, Barnett TP, Santer BD, Gleckler PJ. Selecting global climate models for regional climate change studies. PNAS. 2009; 106: 8441-8446.
