# Supplementary material for: Expertly Validated Models and Phylogenetically-Controlled Analysis Suggests Responses to Climate Change Are Related to Species Traits in the Order Lagomorpha
Source: PLoS One. 2015 Apr 15;10(4):e0122267. doi: 10.1371/journal.pone.0122267 (PMC4398435; doi:10.1371/journal.pone.0122267)

S2 Supporting Information.

Species accounts (#1-89)

Key for response curves

Abbreviation	Description
**HII**	Human Influence index
**Hilliness**	Surface Roughness index
**MaxPrec**	Maximum precipitation
**MaxTemp**	Maximum temperature
**MinPrec**	Minimum precipitation
**MinTemp**	Minimum temperature
**PrecSea**	Precipitation seasonality
**RealMAR**	Mean annual precipitation
**RealMAT**	Mean annual temperature
**TempSea**	Temperature seasonality
**etpsum**	Annual evapotranspiration
**ndvi**	Normalised difference vegetation index
**radiation**	Solar radiation
**wbann**	Annual water balance
**wbpos**	Number of months with a positive water balance

The past and current occurrence records underlying these species accounts can be viewed on http://lagomorphclimatechange.wordpress.com/.

Species account #1 - Pygmy rabbit (*Brachylagus idahoensis*)
n = 39
Expert: Penny Becker, Washington Dept. of Fish & Wildlife, USA
Expert evaluation: Medium
Data: Modern and historic
Envelope: Climatic and habitat
Dispersal distance: 15km/year (Expert)
Status: MODELLABLE; **Included in final analysis:** √

Model evaluation metric	
AUC	0.95
Omission rate	0.10
Sensitivity	0.90
Specificity	1.00
Proportion correct	1.00
**Kappa**	**0.75**
True Skill Statistic	0.90

Summary: The Pygmy rabbit's bioclimatic envelope is predicted to decline by 87% with a 1° mean latitudinal poleward shift and mean increase in elevation of ~300m driven predominately by an increase in mean minimum elevation (>600m) with little change in mean maximum elevation (~50m). 95% of the permutation importance of the model was contributed to by mean annual temperature (64.5%), maximum temperature (25.2%) and annual water balance (5.9%).

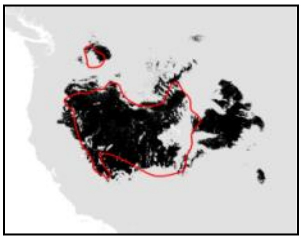

a) ~ 1930s (1900-1949)

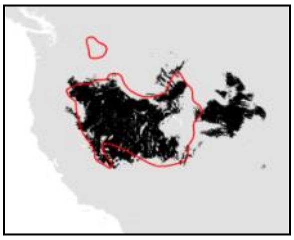

b) ~ 1980s (1950-2000)

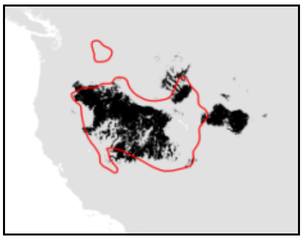

c) ~ 2020s (2010-2039)

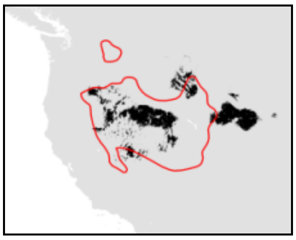

d) ~ 2050s (2040-2069)

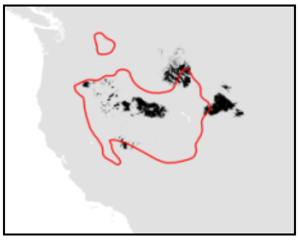

e) ~ 2080s (2070-2099)

Black = predicted suitable envelope

Red polygon = IUCN range (Beauvais GP, *et al*. 2008)

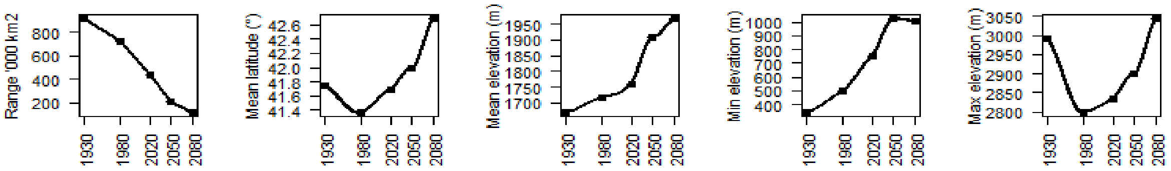

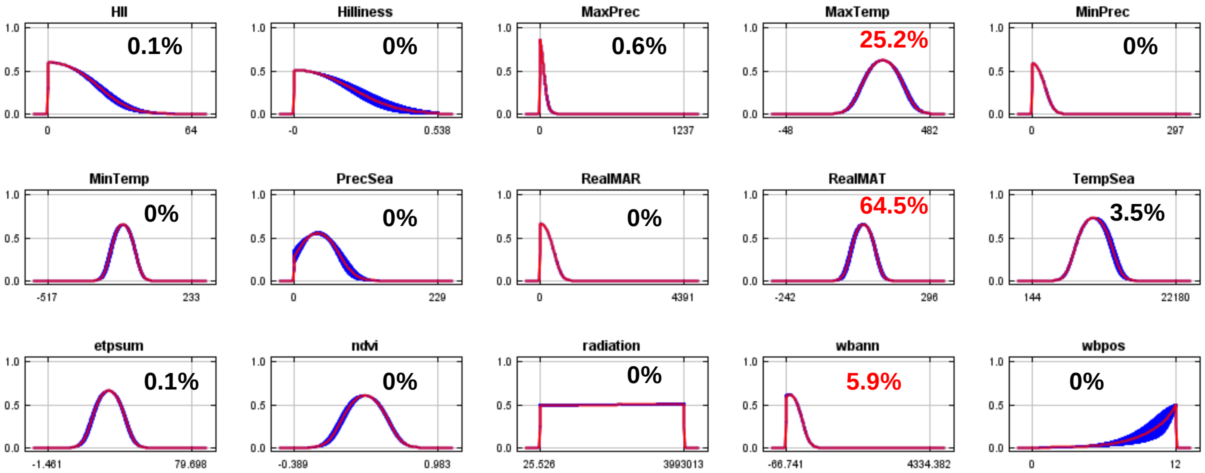

#2 - Riverine rabbit (*Bunolagus monticularis*)
n = 109
Status: Kai Collins, University of Pretoria, South Africa
Expert evaluation: Good
Data: Modern and historic
Envelope: Climatic and habitat
Dispersal distance: 7.5km/year (Expert)
Status: MODELLABLE; **Included in final analysis:** √

Model evaluation metric	
AUC	0.98
Omission rate	0.03
Sensitivity	0.97
Specificity	1.00
Proportion correct	1.00
**Kappa**	**0.85**
True Skill Statistic	0.97

Summary: The Riverine rabbit's bioclimatic envelope is predicted to decline by 85% with a ~1° mean latitudinal poleward shift and mean increase in elevation of ~200m driven by similar increases in both minimum and maximum elevation. 95% of the permutation importance of the model was contributed to by minimum temperature (33.1%) and precipitation (22.5%), temperature seasonality (22.3%), mean annual temperature (6.7%) and precipitation (5.0%), annual evapotranspiration (4.3%) and precipitation seasonality (2.6%).

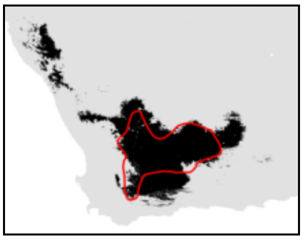

a) ~ 1930s (1900-1949)

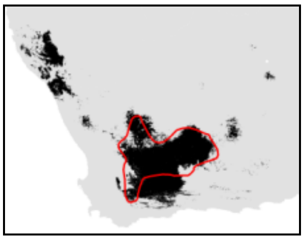

b) ~ 1980s (1950-2000)

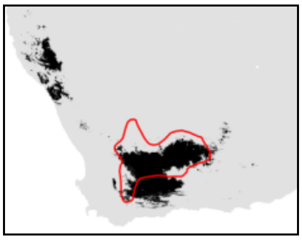

c) ~ 2020s (2010-2039)

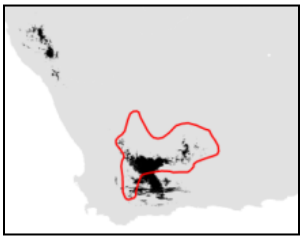

d) ~ 2050s (2040-2069)

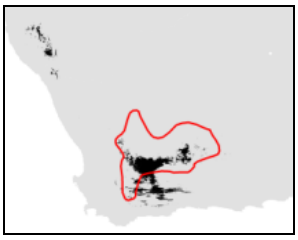

e) ~ 2080s (2070-2099)

Black = predicted suitable envelope

Red polygon = IUCN range (Beauvais GP, *et al*. 2008)

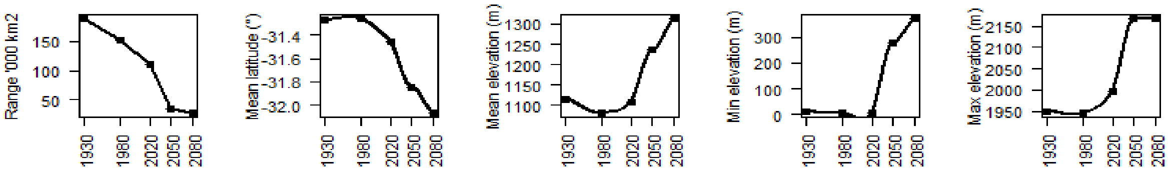

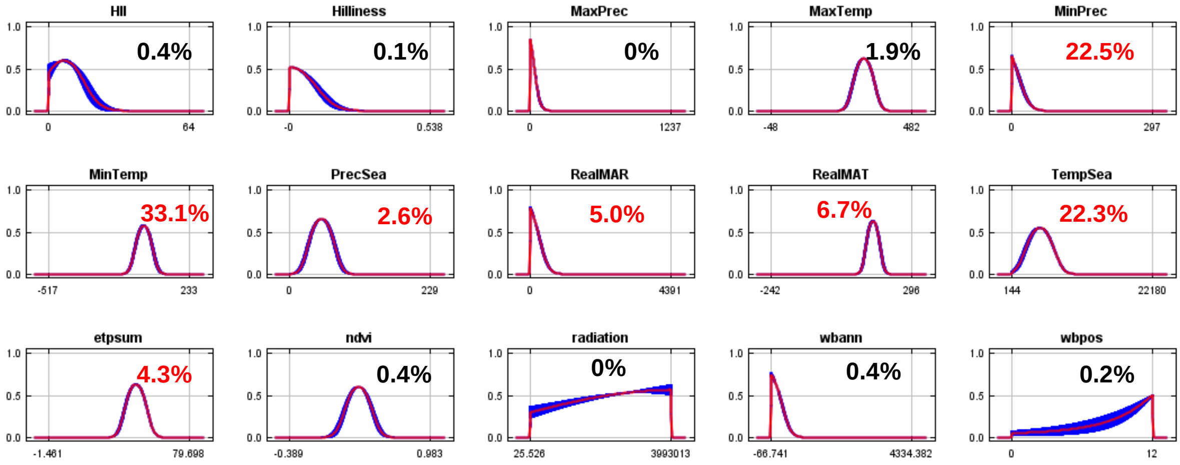

Model evaluation metric	
AUC	0.97
Omission rate	0.06
Sensitivity	0.94
Specificity	0.99
Proportion correct	0.99
**Kappa**	**0.81**
True Skill Statistic	0.94

#3 - Hispid hare (*Caprolagus hispidus*)
n = 18
Expert: Gopinathan Maheswaran, Zoological Survey of India
Expert evaluation: Medium
Data: Modern and historic
Envelope: Climatic and habitat
Dispersal distance: 5km/year (Expert)
Status: MODELLABLE; **Included in final analysis:** √

Summary: The Hispid hare's bioclimatic envelope is predicted to increase by 21% with a ~1.5° mean latitudinal poleward shift and mean increase in elevation of ~70m driven by increases in maximum elevation. 95% of the permutation importance of the model was contributed to by mean annual temperature (52.7%), precipitation seasonality (29.0%), annual evapotranspiration (6.6%), number of months with a positive water balance (2.9%), maximum precipitation (2.2%) and minimum temperature (1.7%).

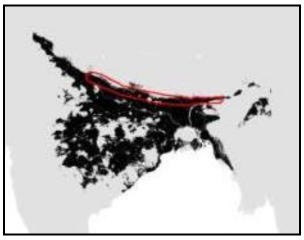
a) ~ 1930s (1900-1949)

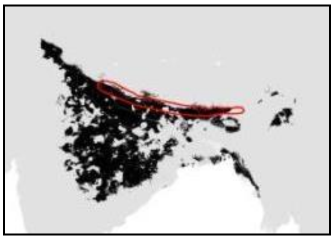
b) ~ 1980s (1950-2000)

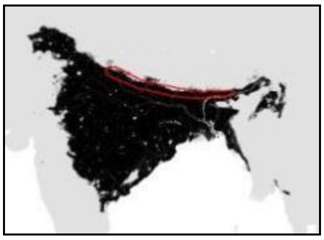
c) ~ 2020s (2010-2039)

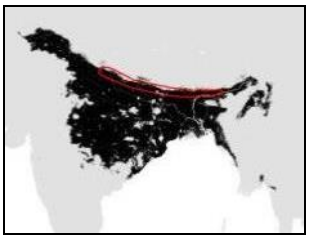
d) ~ 2050s (2040-2069)

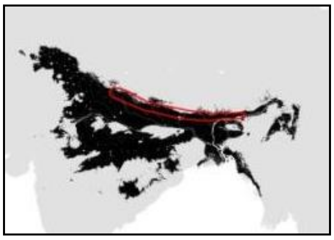
e) ~ 2080s (2070-2099)

Black = predicted suitable envelope

Red polygon = IUCN range (Maheswaran, G. & Smith, A.T. 2008)

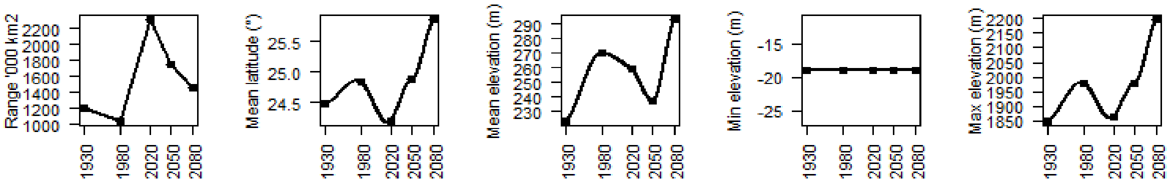

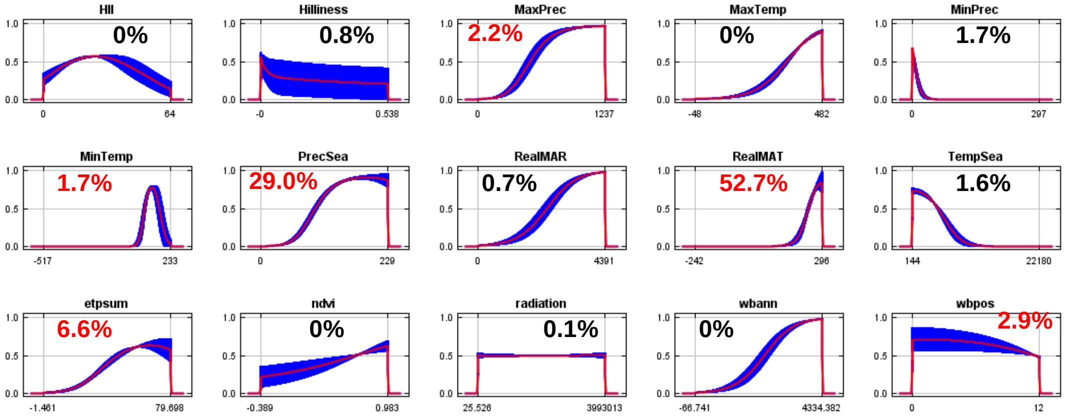

#4 – Antelope jackrabbit (*Lepus alleni*)
n = 32
Expert: Paul Krausman , University of Montana
Expert evaluation: Medium
Data: Modern and historic
Envelope: Climatic and habitat
Dispersal distance: 25km/year (Expert)
Status: UNMODELLABLE; **Included in final analysis:** X

Model evaluation metric	
AUC	0.91
Omission rate	0.16
Sensitivity	0.84
Specificity	0.99
Proportion correct	0.98
**Kappa**	**0.26**
True Skill Statistic	0.83

Summary: The Antelope jackrabbit's bioclimatic envelope is predicted to increase by 172% with a ~3° mean latitudinal poleward shift and mean increase in elevation of ~20m driven by increases in maximum elevation. 95% of the permutation importance of the model was contributed to by precipitation seasonality (24.8%), minimum precipitation (21.1%), annual water balance (16.3%), minimum temperature (13.0%), temperature seasonality (7.3%), normalised difference vegetation index (5.8%), annual evapotranspiration (5.2%) and human influence index (3.7%).

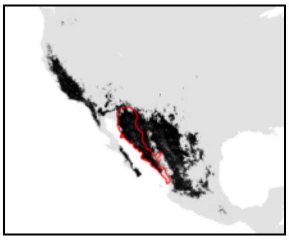

a) ~ 1930s (1900-1949)

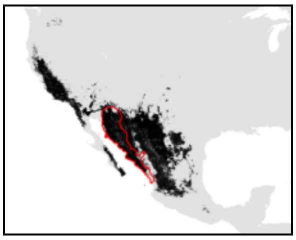

b) ~ 1980s (1950-2000)

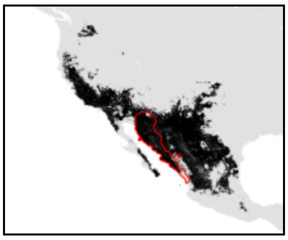

c) ~ 2020s (2010-2039)

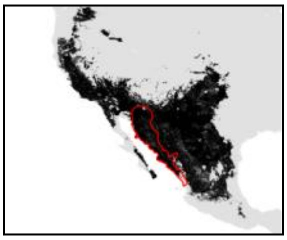

d) ~ 2050s (2040-2069)

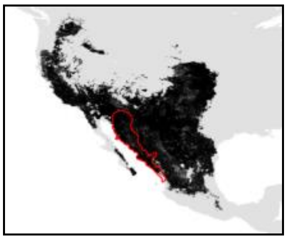

e) ~ 2080s (2070-2099)

Black = predicted suitable envelope

Red polygon = IUCN range (Malpica, F.J. & Rangel Cordero, H. 2008)

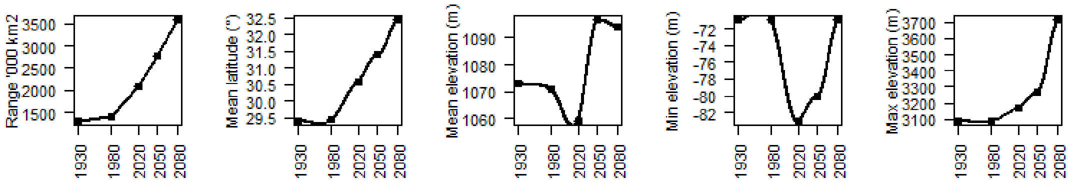

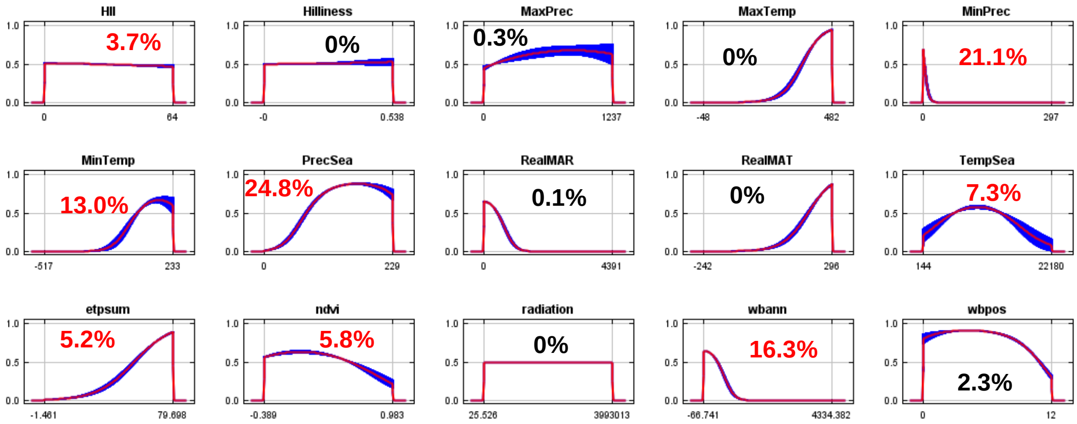

#5 – Snowshoe hare (*Lepus americanus*)
n = 506
Expert: Charles Krebs, University of British Colombia & Rudy Boonstra, University of Toronto Scarborough
Expert evaluation: Good
Data: Only modern
Envelope: Climatic and habitat
Dispersal distance: 24km/year (Expert)
Status: MODELLABLE; **Included in final analysis:** √

Model evaluation metric	
AUC	0.95
Omission rate	0.07
Sensitivity	0.93
Specificity	0.97
Proportion correct	0.97
**Kappa**	**0.72**
True Skill Statistic	0.90

Summary: The Snowshoe hare's bioclimatic envelope is predicted to decline by 7% with a ~2° mean latitudinal poleward shift and mean decrease in elevation of ~10m, but with increases in both minimum and maximum elevation. 95% of the permutation importance of the model was contributed to by mean annual temperature (83.1%), maximum temperature (7.2%), normalised difference vegetation index (2.1%), mean annual precipitation (1.5%) and annual evapotranspiration (1.2%).

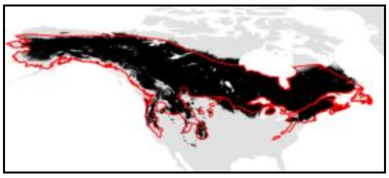

a) ~ 1930s (1900-1949)

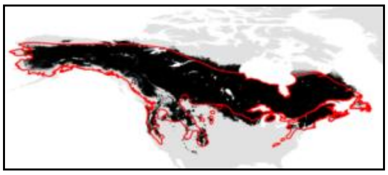

b) ~ 1980s (1950-2000)

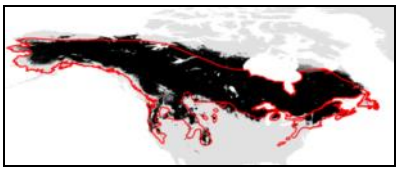

c) ~ 2020s (2010-2039)

Black = predicted suitable envelope

Red polygon = IUCN range (Murray, D. & Smith, A.T. 2008)

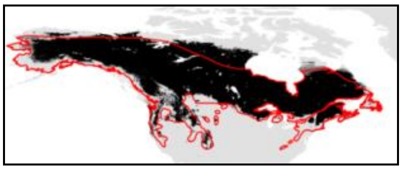

d) ~ 2050s (2040-2069)

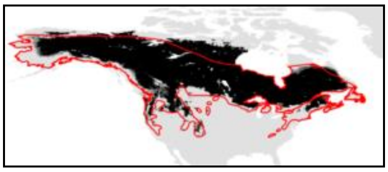

e) ~ 2080s (2070-2099)

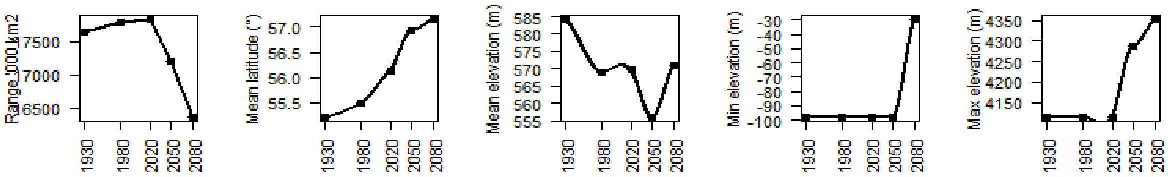

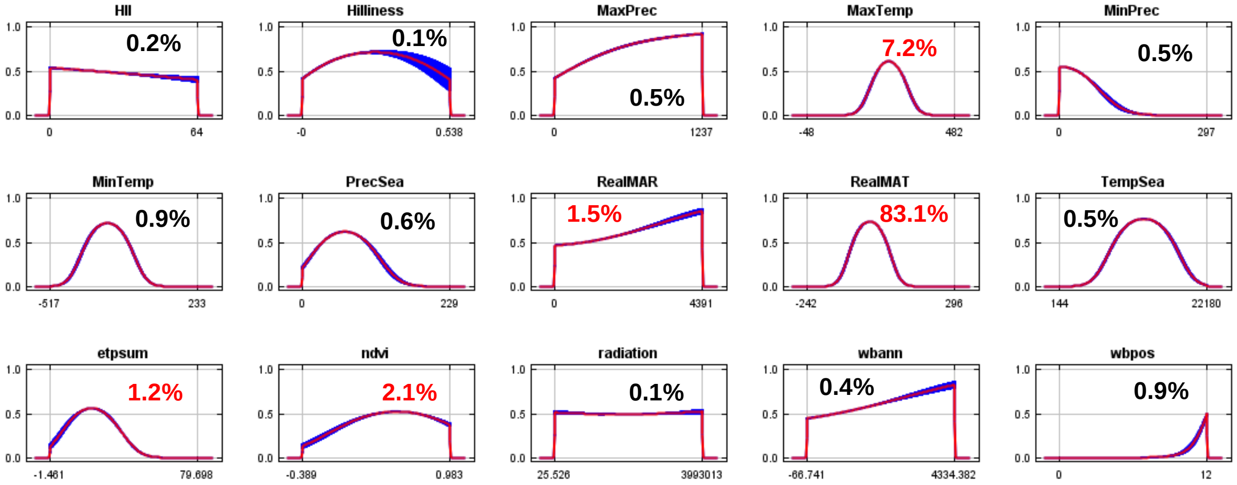

#6 – Arctic hare (*Lepus arcticus*)
n = 18
Expert: David Gray, Grayhound Information Services
Expert evaluation: Poor
Data: Modern and historic
Envelope: Climatic and habitat
Dispersal distance: 2km/year (Chapman & Flux, 1990)
Status: UNMODELLABLE; **Included in final analysis:** X

Model evaluation metric	
AUC	0.97
Omission rate	0.06
Sensitivity	0.94
Specificity	0.99
Proportion correct	0.99
**Kappa**	**0.36**
True Skill Statistic	0.94

Summary: The Arctic hare's bioclimatic envelope is predicted to decline by 30% with a ~2° mean latitudinal poleward shift and mean decrease in elevation of ~10m driven by decreases in maximum elevation. 95% of the permutation importance of the model was contributed to by maximum temperature (34.0%), annual evapotranspiration (15.4%), surface roughness index (14.4%), human influence index (10.1%), normalised difference vegetation index (9.2%), mean annual temperature (4.4%), precipitation seasonality (1.5%), mean annual precipitation (1.5%), maximum precipitation (0.3%), minimum precipitation (0.3%), solar radiation (0.3%), minimum temperature (0.1%) and number of months with a positive water balance (0.1%).

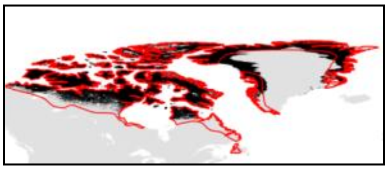

a) ~ 1930s (1900-1949)

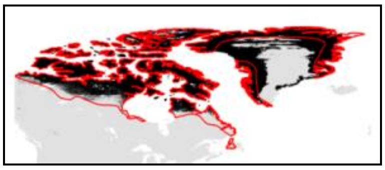

b) ~ 1980s (1950-2000)

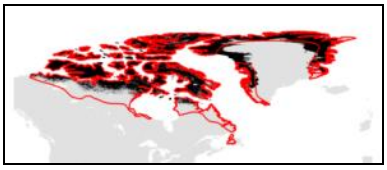

c) ~ 2020s (2010-2039)

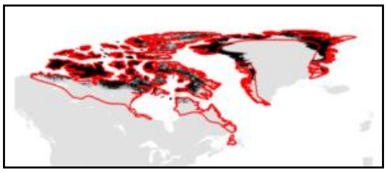

d) ~ 2050s (2040-2069)

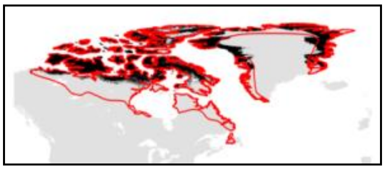

e) ~ 2080s (2070-2099)

Black = predicted suitable envelope

Red polygon = IUCN range (Murray, D. & Smith, A.T. 2008)

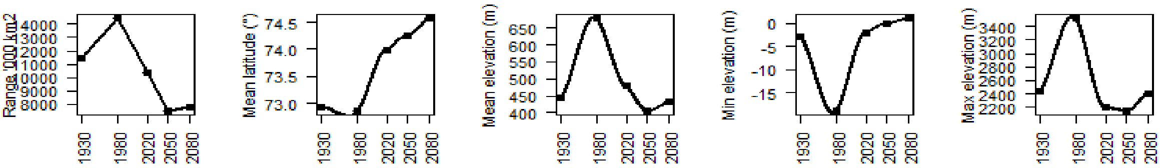

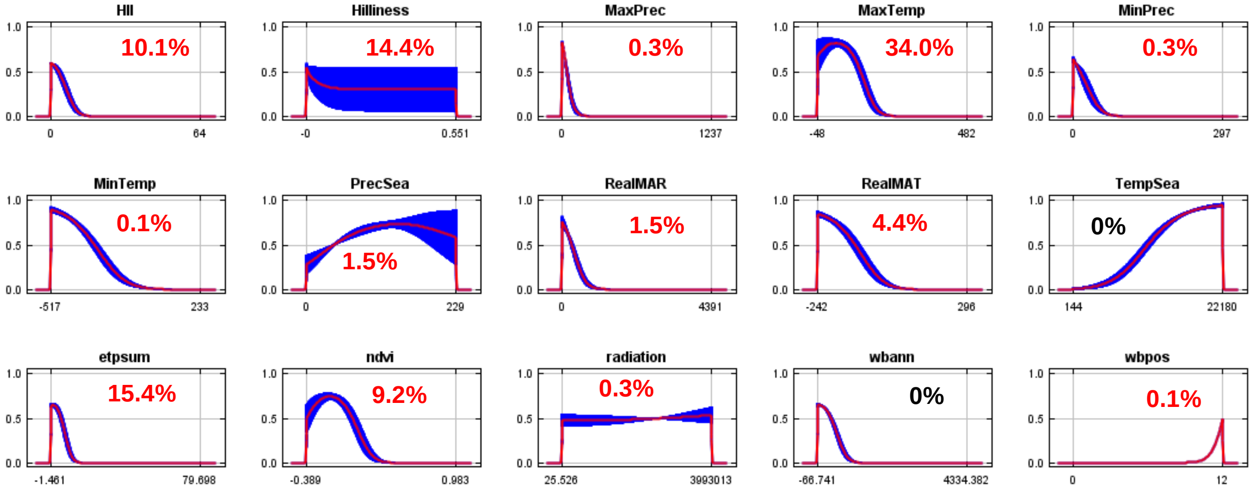

#7 – Japanese hare (*Lepus brachyurus*)
n = 9
Expert: Koji Shimano, Shinshu University, Japan
Expert evaluation: Medium
Data: Modern and historic
Envelope: Climatic and habitat
Dispersal distance: 1km/year (Expert)
Status: MODELLABLE; **Included in final analysis:** √

Model evaluation metric	
AUC	0.99
Omission rate	0.00
Sensitivity	1.00
Specificity	0.99
Proportion correct	0.99
**Kappa**	**0.43**
True Skill Statistic	0.99

Summary: The Japanese hare's bioclimatic envelope is predicted to increase by 9% with no latitudinal poleward shift and a mean increase in elevation of ~10m driven by a decrease in minimum elevation. 95% of the permutation importance of the model was contributed to by temperature seasonality (31.8%), annual water balance (20.6%), human influence index (13.5%), mean annual precipitation (8.9%), maximum precipitation (6.2%), precipitation seasonality (5.4%) and minimum precipitation (4.4%).

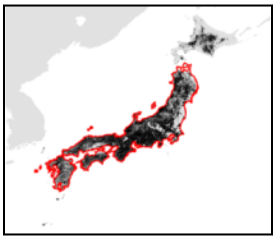

a) ~ 1930s (1900-1949)

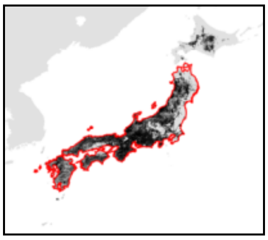

b) ~ 1980s (1950-2000)

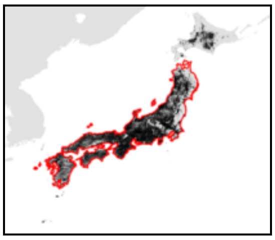

c) ~ 2020s (2010-2039)

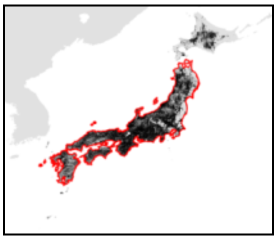

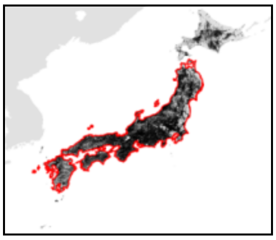

Black = predicted suitable envelope

Red polygon = IUCN range (Yamada, F. & Smith, A.T. 2008)

d) ~ 2050s (2040-2069)

e) ~ 2080s (2070-2099)

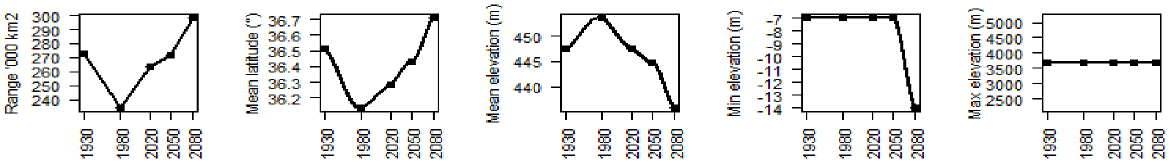

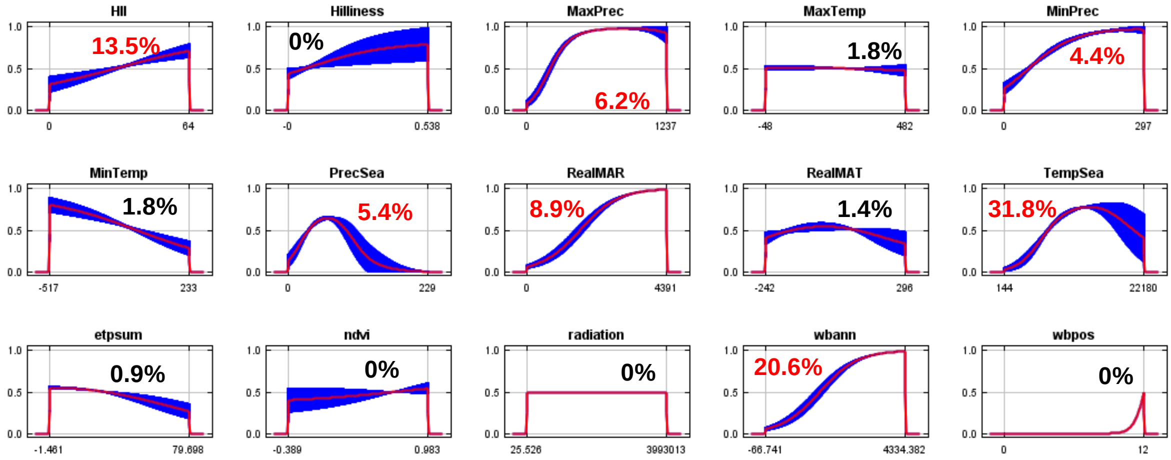

#8 – Black-tailed jackrabbit (*Lepus californicus*)

n = 970
Expert: Alejandro Velasquez, UNAM-Canada
Expert evaluation: Medium
Data: Modern and historic
Envelope: Climatic and habitat
Dispersal distance: 18.9km/year (N.Am. leporids, range 2-25)
Status: MODELLABLE; **Included in final analysis:** √

Model evaluation metric	
AUC	0.93
Omission rate	0.07
Sensitivity	0.93
Specificity	0.94
Proportion correct	0.94
**Kappa**	**0.69**
True Skill Statistic	0.87

Summary: The Black-tailed jackrabbit's bioclimatic envelope is predicted to decline by 25% with a ~2° mean latitudinal poleward shift and mean decrease in elevation of ~75m, but with increases in both minimum and maximum elevation. 95% of the permutation importance of the model was contributed to by precipitation seasonality (31.8%), annual evapotranspiration (18.5%), maximum temperature (17.0%), mean annual temperature (8.9%), minimum temperature (8.0%), minimum precipitation (4.0%), human influence index (3.9%) and temperature seasonality (3.5%).

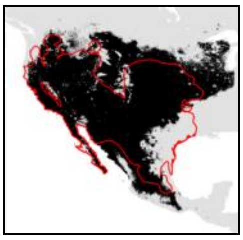
a) ~ 1930s (1900-1949)

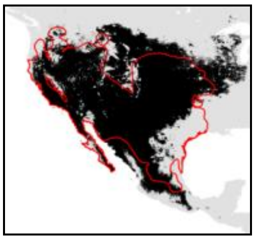
b) ~ 1980s (1950-2000)

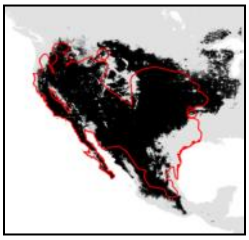
c) ~ 2020s (2010-2039)

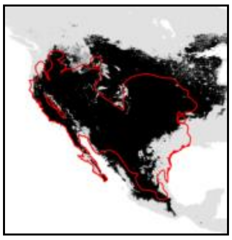
d) ~ 2050s (2040-2069)

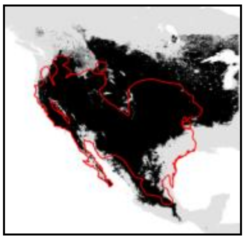
e) ~ 2080s (2070-2099)

Black = predicted suitable envelope

Red polygon = IUCN range (Romero Malpica, F.J. & Rangel Cordero, H. 2008)

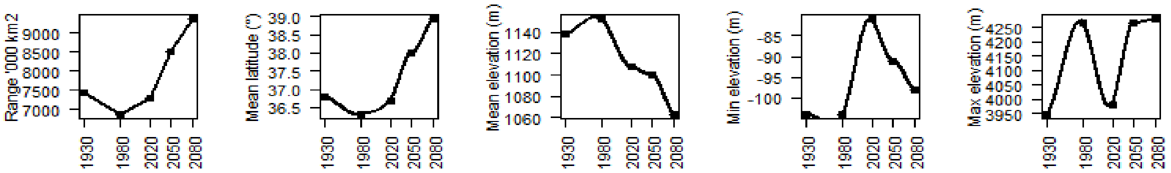

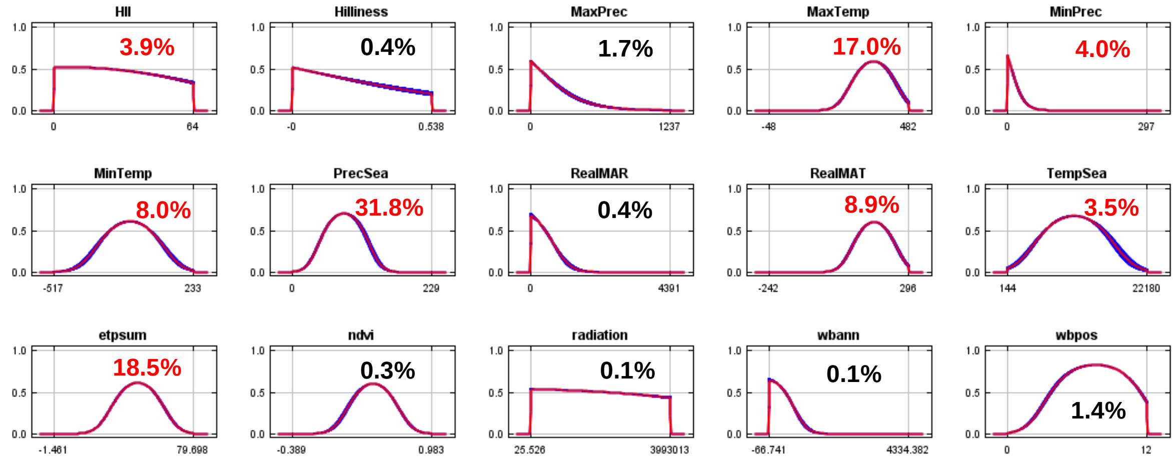

#9 – White-sided jackrabbit (*Lepus callotis*)
n = 37
Expert: Jennifer Frey, New Mexico State University
Expert evaluation: Medium
Data: Modern and historic
Envelope: Climatic and habitat
Dispersal distance: 18.9km/year (N.Am. leporids, range 2-25)
Status: UNMODELLABLE; **Included in final analysis:** X

Model evaluation metric	
AUC	0.97
Omission rate	0.05
Sensitivity	0.95
Specificity	0.99
Proportion correct	0.99
**Kappa**	**0.36**
True Skill Statistic	0.93

Summary: The White-sided jackrabbit's bioclimatic envelope is predicted to increase by 3% with a ~1° mean latitudinal poleward shift and a mean increase in elevation of ~150m driven by an increase in maximum elevation. 95% of the permutation importance of the model was contributed to by precipitation seasonality (35.4%), annual evapotranspiration (22.3%), minimum temperature (17.0%), mean annual temperature (10.5%), minimum precipitation (6.5%), maximum precipitation (6.5%), surface roughness index (3.0%) and number of months with a positive water balance (2.7%).

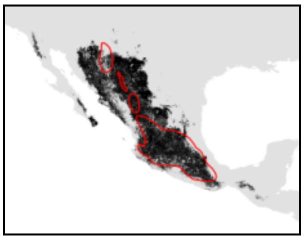
a) ~ 1930s (1900-1949)

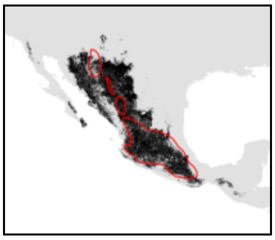
b) ~ 1980s (1950-2000)

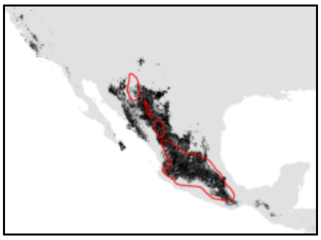
c) ~ 2020s (2010-2039)

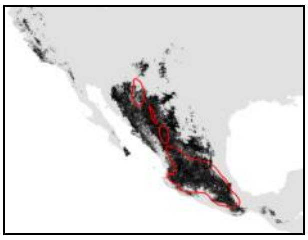
d) ~ 2050s (2040-2069)

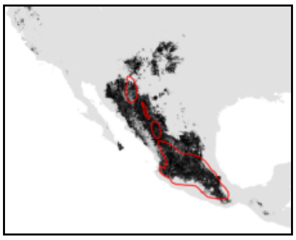
e) ~ 2080s (2070-2099)

Black = predicted suitable envelope

Red polygon = IUCN range (Romero Malpica, F.J. & Rangel Cordero, H. 2008)

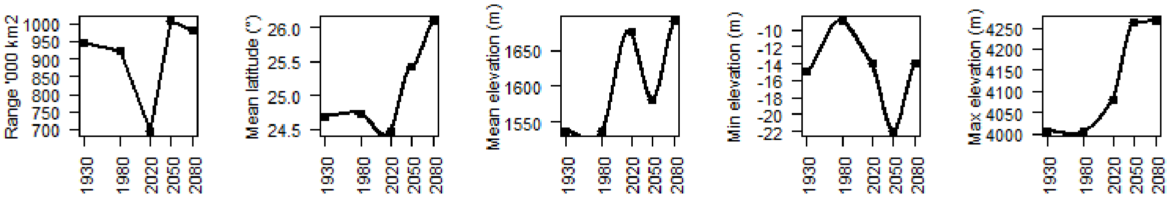

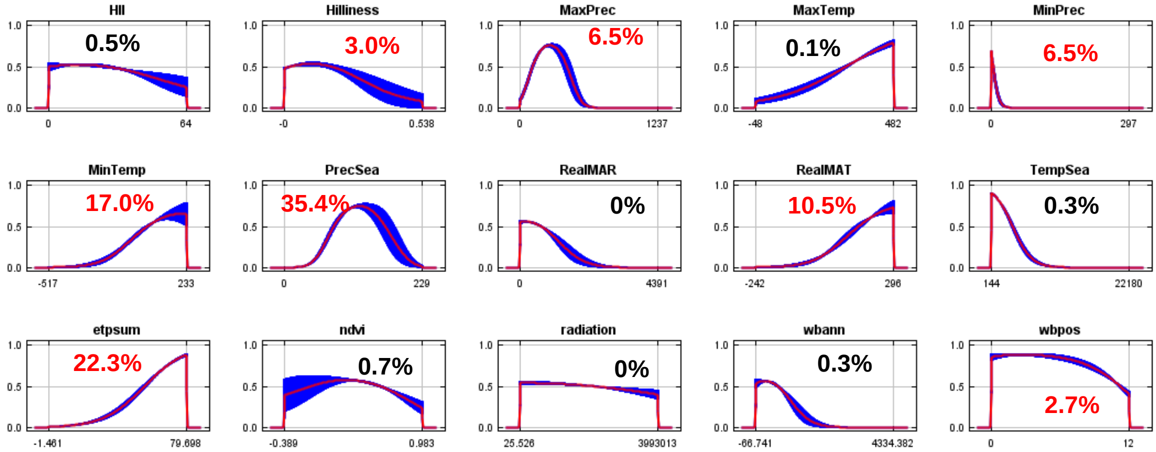

#10 – Cape hare (*Lepus capensis*)
n = 231
Expert: John Flux, IUCN Lagomorph Specialist Group
Expert evaluation: Poor
Data: Modern and historic
Envelope: Climatic and habitat
Dispersal distance: 35km/year (Expert)
Status: UNMODELLABLE; **Included in final analysis:** X

Model evaluation metric	
AUC	0.93
Omission rate	0.10
Sensitivity	0.90
Specificity	0.97
Proportion correct	0.97
**Kappa**	**0.56**
True Skill Statistic	0.87

Summary: The Cape hare's bioclimatic envelope is predicted to decrease by 45% with ~2° mean latitudinal shift towards the Equator and a mean increase in elevation of ~330m driven by an increase in maximum elevation. 95% of the permutation importance of the model was contributed to by annual evapotranspiration (33.1%), minimum precipitation (29.6%), maximum temperature (9.7%), human influence index (7.2%), normalised difference vegetation index (4.6%), minimum temperature (3.2%), number of months with a positive water balance (2.9%), maximum precipitation (2.2%), mean annual precipitation (2.1%) and precipitation seasonality (2.0%).

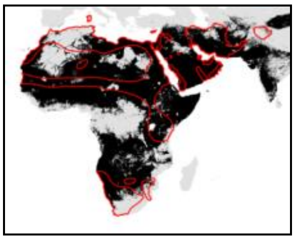
a) ~ 1930s (1900-1949)

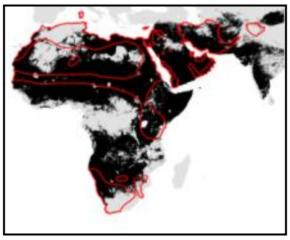
b) ~ 1980s (1950-2000)

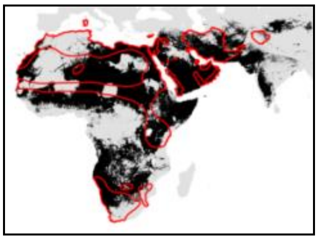
c) ~ 2020s (2010-2039)

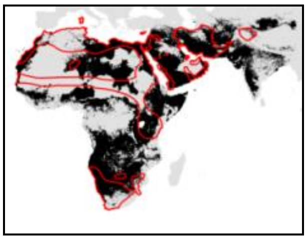
d) ~ 2050s (2040-2069)

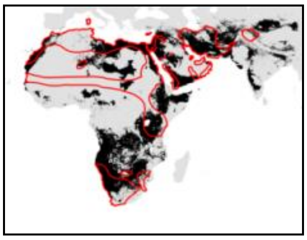
e) ~ 2080s (2070-2099)

Black = predicted suitable envelope

Red polygon = IUCN range (Drew, C., *et al*. 2008)

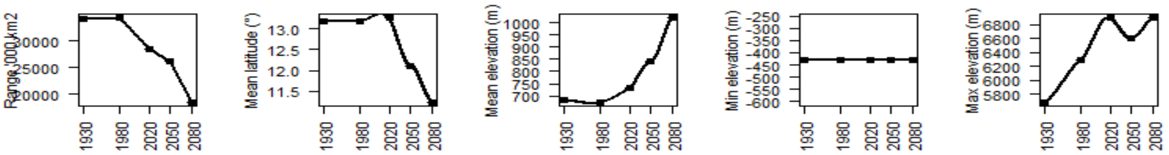

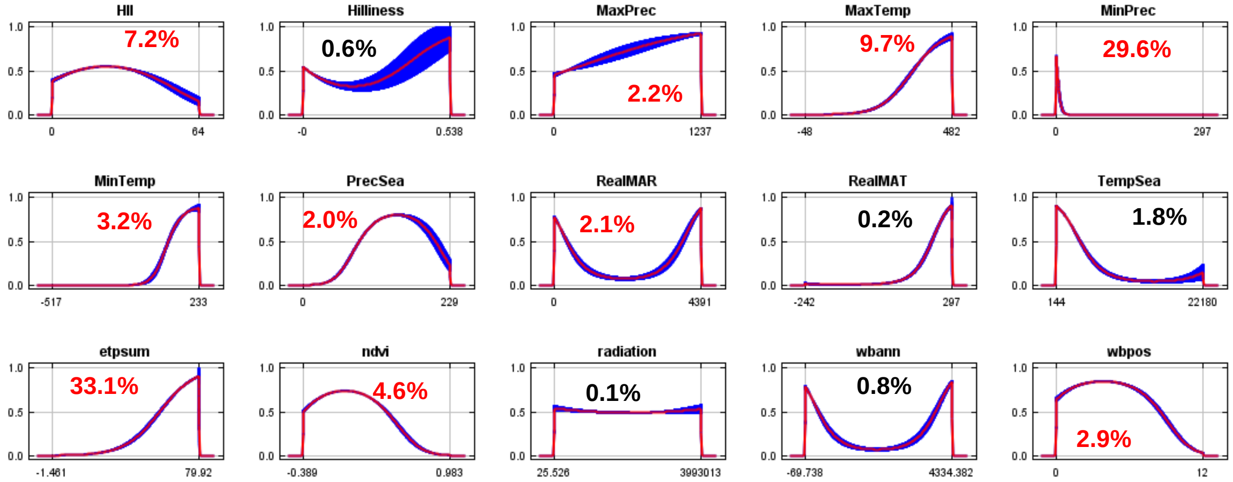

#11 – Broom hare (*Lepus castroviejoi*)
n = 164
Expert: Pelayo Acevedo, University of Porto
Expert evaluation: Medium
Data: Only modern
Envelope: Climatic and habitat
Dispersal distance: 1km/year (Expert)
Status: MODELLABLE; **Included in final analysis:** √

Model evaluation metric	
AUC	0.94
Omission rate	0.11
Sensitivity	0.89
Specificity	0.99
Proportion correct	0.99
**Kappa**	**0.80**
True Skill Statistic	0.89

Summary: The Broom hare's bioclimatic envelope is predicted to decrease by 90% with a ~0.2° mean latitudinal poleward shift and a mean increase in elevation of ~450m driven by an increase in minimum elevation. 95% of the permutation importance of the model was contributed to by mean annual temperature (62.0%), maximum temperature (20.6%), temperature seasonality (10.9%) and surface roughness index (3.8%).

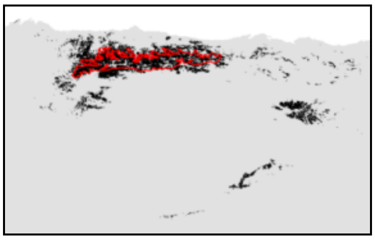
a) ~ 1930s (1900-1949)

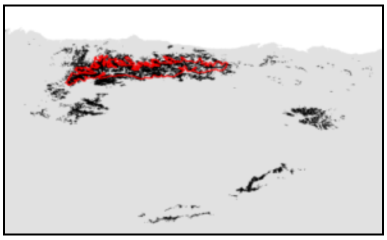
b) ~ 1980s (1950-2000)

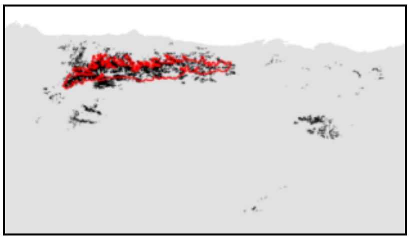
c) ~ 2020s (2010-2039)

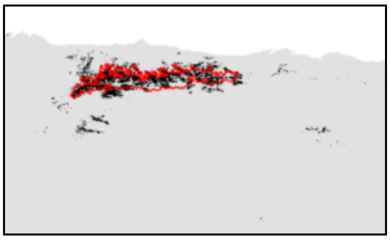
d) ~ 2050s (2040-2069)

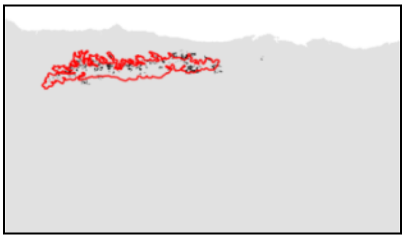
e) ~ 2080s (2070-2099)

Black = predicted suitable envelope

Red polygon = IUCN range (Smith, A.T. & Johnston, C.H. 2008)

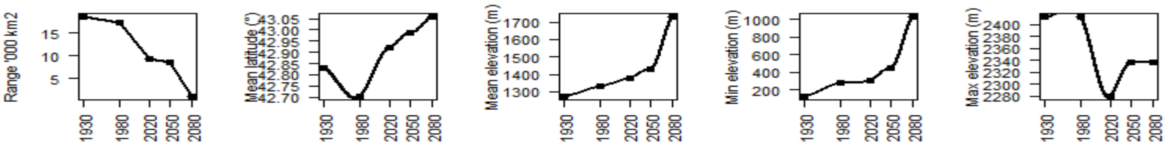

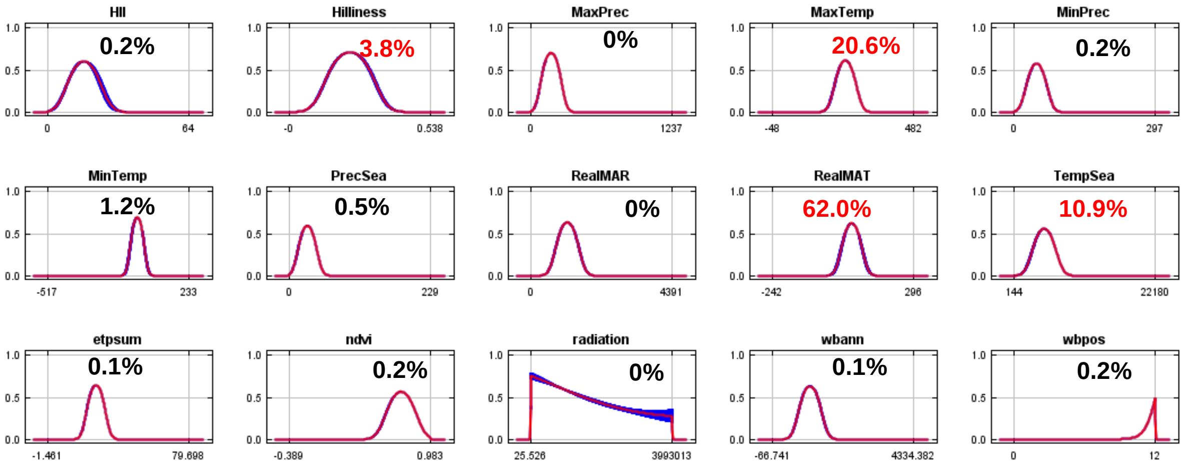

#12 – Yunnan hare (*Lepus comus*)
n = 59
Expert: Weihe Yang, Institute of Zoology, Chinese Academy of Sciences
Expert evaluation: Medium
Data: Modern and historic
Envelope: Climatic and habitat
Dispersal distance: 2.5km/year (Asian leporids, range 1-35)
Status: MODELLABLE; **Included in final analysis:** √

Model evaluation metric	
AUC	0.92
Omission rate	0.15
Sensitivity	0.85
Specificity	0.99
Proportion correct	0.99
**Kappa**	**0.67**
True Skill Statistic	0.84

Summary: The Yunnan hare's bioclimatic envelope is predicted to decrease by 65% with a ~0.1° mean latitudinal poleward shift and a mean increase in elevation of ~100m driven by both increases in maximum and minimum elevation. 95% of the permutation importance of the model was contributed to by precipitation seasonality (59.9%), maximum temperature (26.8%), temperature seasonality (2.9%), number of months with a positive water balance (2.6%), annual evapotranspiration (2.0%) and mean annual temperature (1.7%).

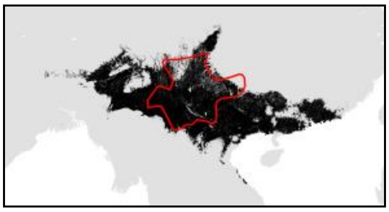
a) ~ 1930s (1900-1949)

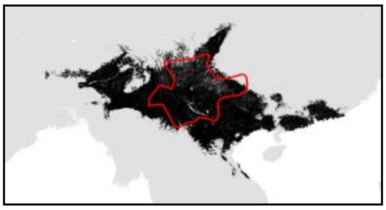
b) ~ 1980s (1950-2000)

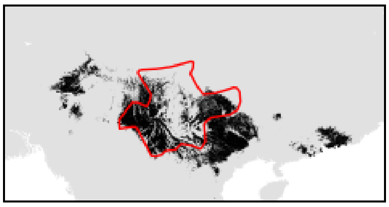
c) ~ 2020s (2010-2039)

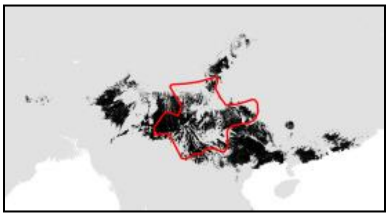
d) ~ 2050s (2040-2069)

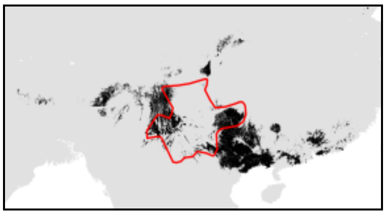
e) ~ 2080s (2070-2099)

Black = predicted suitable envelope

Red polygon = IUCN range (Smith, A.T. & Johnston, C.H. 2008)

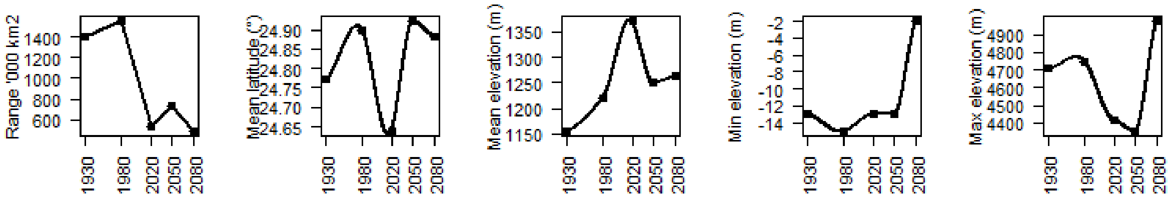

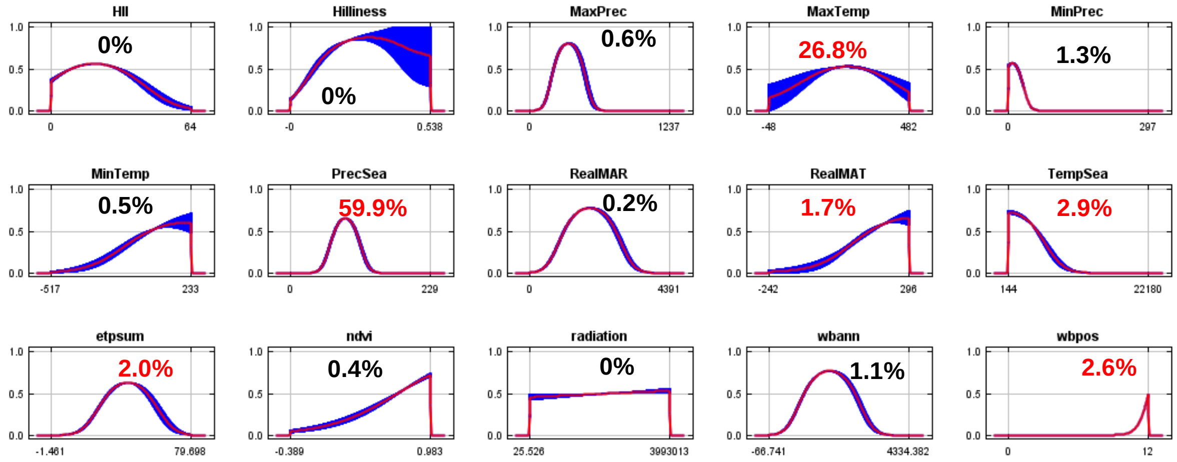

#13 – Korean hare (*Lepus coreanus*)
n = 6
Expert: Weihe Yang, Institute of Zoology, Chinese Academy of Sciences
Expert evaluation: Medium
Data: Modern and historic
Envelope: Climatic and habitat
Dispersal distance: 2.5km/year (Asian leporids, range 1-35)
Status: MODELLABLE; **Included in final analysis:** √

Model evaluation metric	
AUC	0.99
Omission rate	0.00
Sensitivity	1.00
Specificity	0.99
Proportion correct	0.99
**Kappa**	**0.86**
True Skill Statistic	0.99

Summary: The Korean hare's bioclimatic envelope is predicted to increase by 500% with a ~8° mean latitudinal poleward shift and a mean increase in elevation of ~70m driven by an increase in minimum elevation. 95% of the permutation importance of the model was contributed to by temperature seasonality (27.2%), mean annual precipitation (25.6%), minimum temperature (17.7%), annual water balance (13.1%), normalised difference vegetation index (4.7%), precipitation seasonality (4.5%) and human influence index (2.4%).

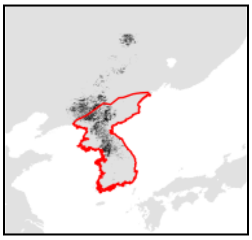
a) ~ 1930s (1900-1949)

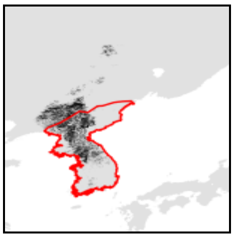
b) ~ 1980s (1950-2000)

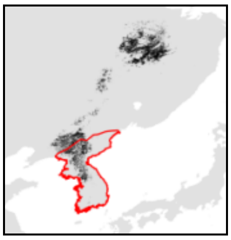
c) ~ 2020s (2010-2039)

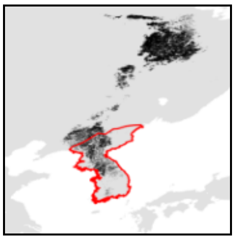
d) ~ 2050s (2040-2069)

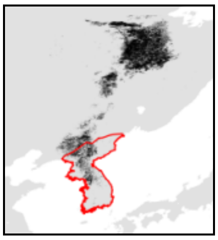
e) ~ 2080s (2070-2099)

Black = predicted suitable envelope

Red polygon = IUCN range (Smith, A.T. & Johnston, C.H. 2008)

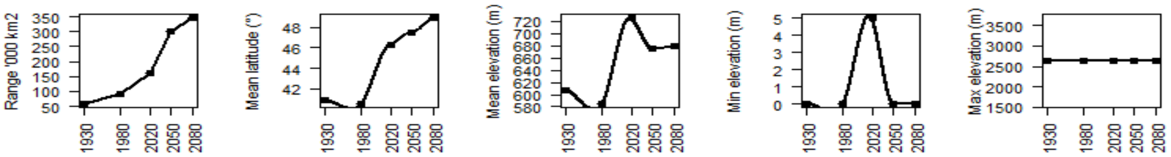

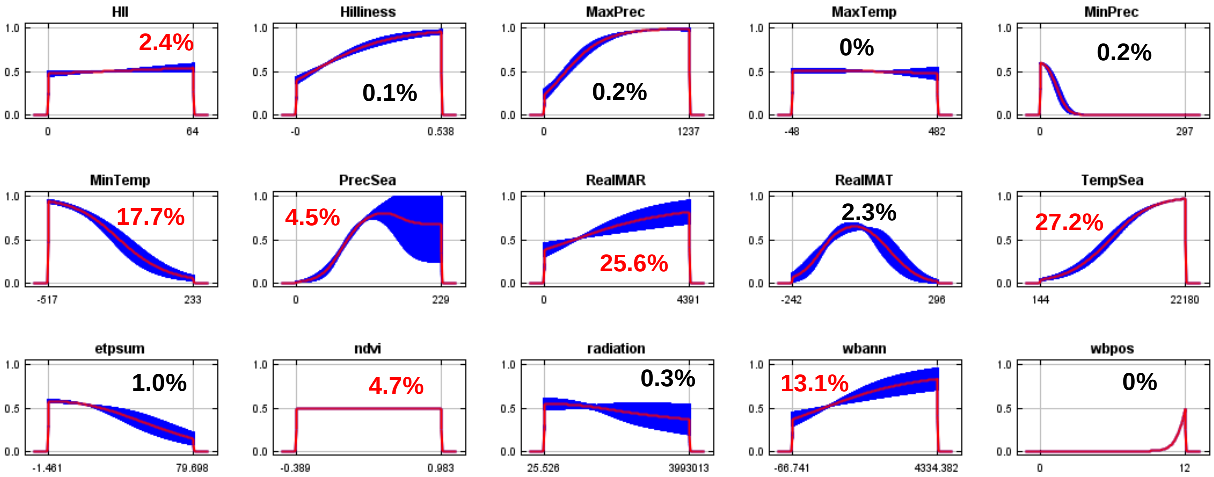

#14 – Apennine hare (*Lepus corsicanus*)
n = 59
Expert: Francesco Angelici, Italian Foundation of Vertebrate Zoology
Expert evaluation: Medium
Data: Only modern
Envelope: Climatic and habitat
Dispersal distance: 3km/year (Expert)
Status: MODELLABLE; **Included in final analysis:** √

Model evaluation metric	
AUC	0.99
Omission rate	0.00
Sensitivity	1.00
Specificity	0.99
Proportion correct	0.99
**Kappa**	**0.53**
True Skill Statistic	0.99

Summary: The Apennine hare's bioclimatic envelope is predicted to increase by 125% with a ~2° mean latitudinal poleward shift and a mean decrease in elevation of ~60m. 95% of the permutation importance of the model was contributed to by minimum temperature (37.9%), annual evapotranspiration (34.3%), temperature seasonality (11.4%), minimum precipitation (11.1%) and maximum temperature (2.8%).

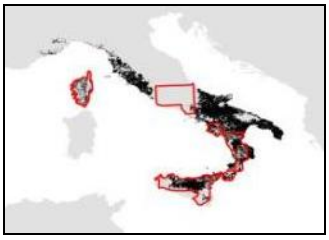
a) ~ 1930s (1900-1949)

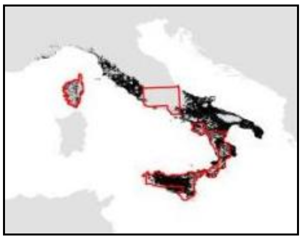
b) ~ 1980s (1950-2000)

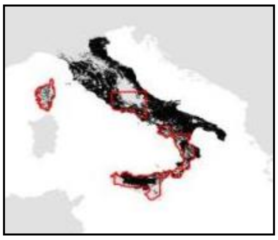
c) ~ 2020s (2010-2039)

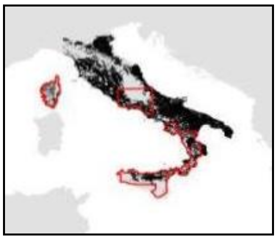
d) ~ 2050s (2040-2069)

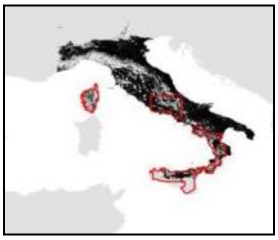
e) ~ 2080s (2070-2099)

Black = predicted suitable envelope

Red polygon = IUCN range (Angelici, F.M., Randi, E., Riga, F. & Trocchi, V. 2008)

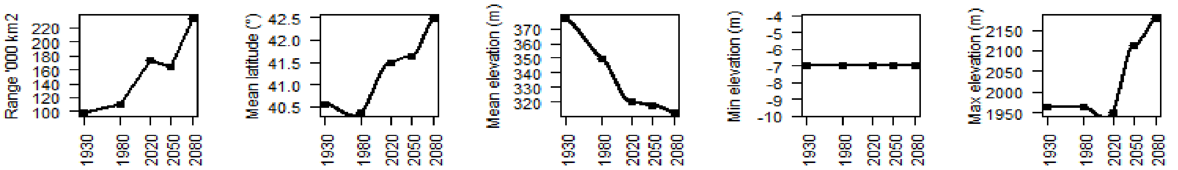

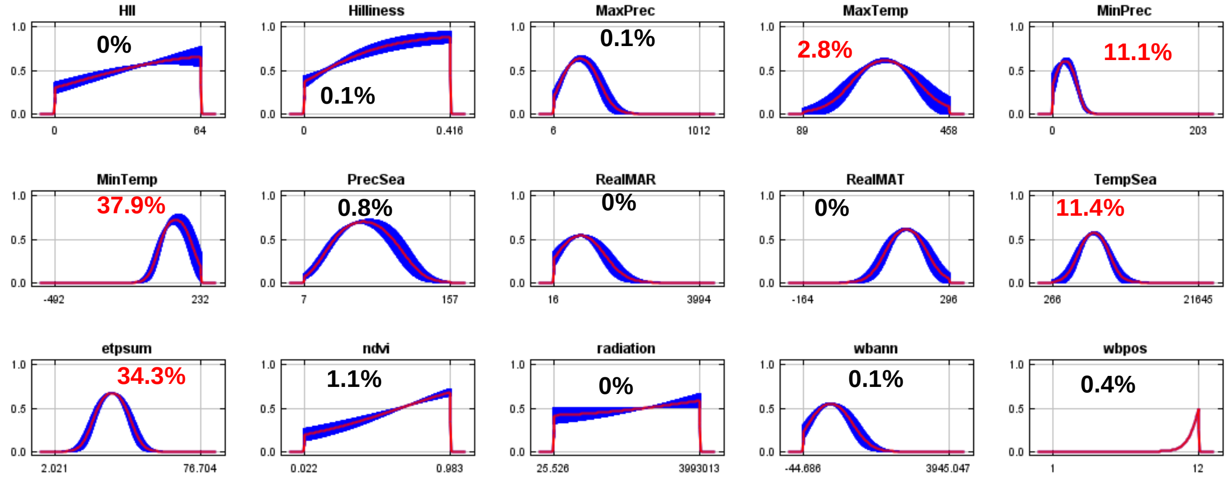

#15 – European hare (*Lepus europaeus*)-native range only
n = 6,186
Expert: Neil Reid, Queen's University Belfast
Expert evaluation: Medium
Data: Only modern
Envelope: Climatic and habitat
Dispersal distance: 2km/year (Chapman & Flux, 1990)
Status: MODELLABLE; **Included in final analysis:** √

Model evaluation metric	
AUC	0.81
Omission rate	0.07
Sensitivity	0.93
Specificity	0.69
Proportion correct	0.78
**Kappa**	**0.57**
True Skill Statistic	0.62

Summary: The European hare's bioclimatic envelope is predicted to increase by 50% with a ~3° mean latitudinal poleward shift and a mean increase in elevation of ~20m driven by an increase in both maximum and minimum elevation. 95% of the permutation importance of the model was contributed to by annual evapotranspiration (58.0%), minimum temperature (9.0%), temperature seasonality (7.3%), surface roughness index (5.5%), human influence index (5.1%), precipitation seasonality (3.3%), annual water balance (3.3%), minimum precipitation (3.0%), normalised difference vegetation index (2.4%) and mean annual temperature (1.5%).

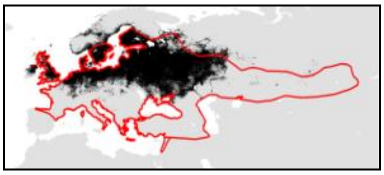

a) ~ 1930s (1900-1949)

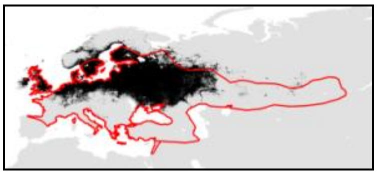

b) ~ 1980s (1950-2000)

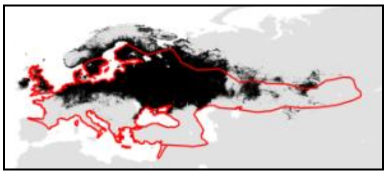

c) ~ 2020s (2010-2039)

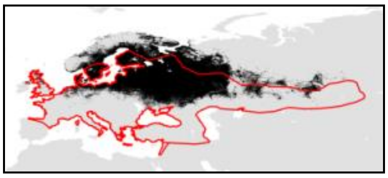

d) ~ 2050s (2040-2069)

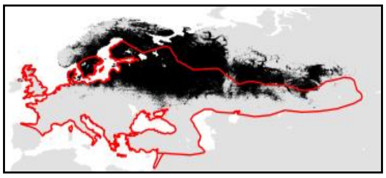

e) ~ 2080s (2070-2099)

Black = predicted suitable envelope

Red polygon = IUCN range (Smith, A.T. & Johnston, C.H. 2008)

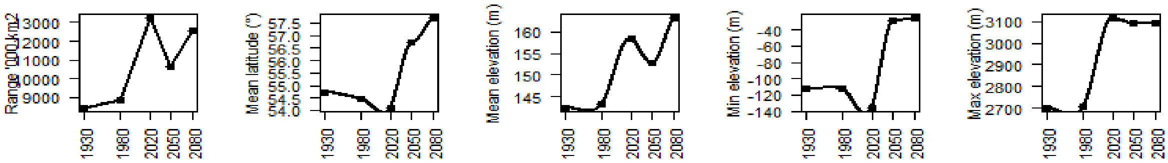

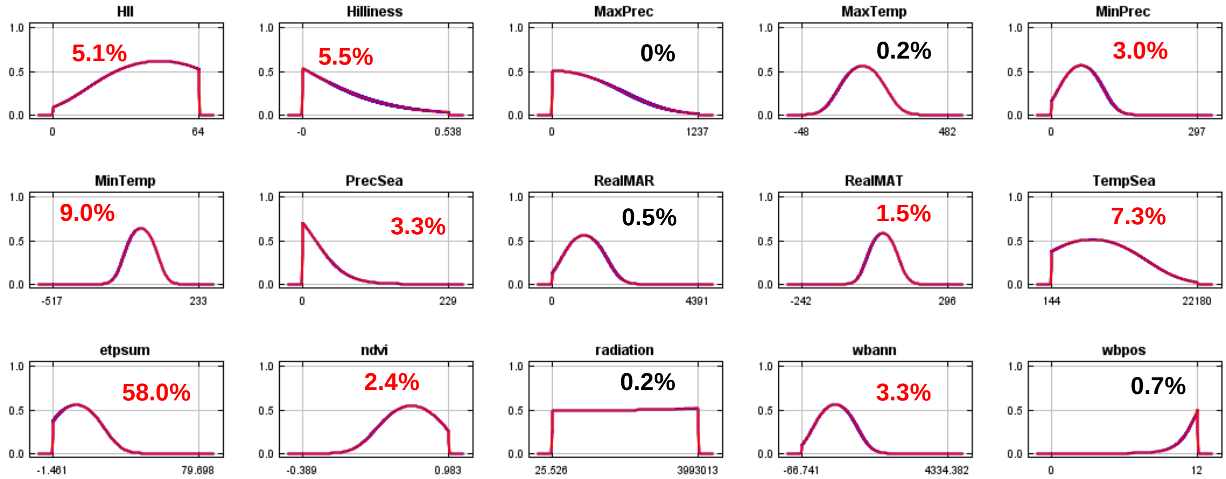

#16 – Ethiopian hare (*Lepus fagani*)
n = 9
Expert: Zelalem Tolesa, Addis Ababa University
Expert evaluation: Poor
Data: Modern and historic
Envelope: Climatic and habitat
Dispersal distance: 25km/year (African leporids, range 15-35)
Status: UNMODELLABLE; **Included in final analysis:** X

Model evaluation metric	
AUC	0.99
Omission rate	0.00
Sensitivity	1.00
Specificity	0.99
Proportion correct	0.99
**Kappa**	**0.56**
True Skill Statistic	0.99

Summary: The Ethiopian hare's bioclimatic envelope is predicted to decrease by 15% with no latitudinal poleward shift and a mean increase in elevation of ~200m driven by an increase in maximum and minimum elevation. 95% of the permutation importance of the model was contributed to by temperature seasonality (92.7%) and annual evapotranspiration (5.3%).

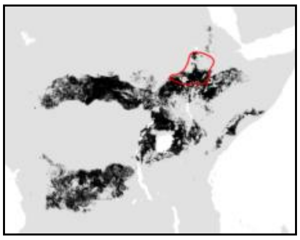
a) ~ 1930s (1900-1949)

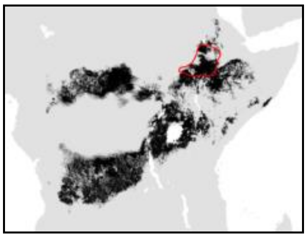
b) ~ 1980s (1950-2000)

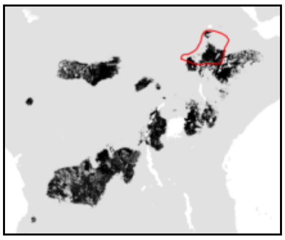
c) ~ 2020s (2010-2039)

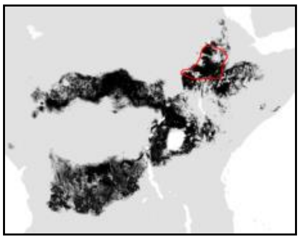
d) ~ 2050s (2040-2069)

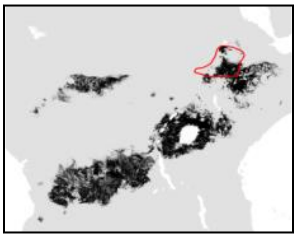
e) ~ 2080s (2070-2099)

Black = predicted suitable envelope

Red polygon = IUCN range (Smith, A.T. & Johnston, C.H. 2008)

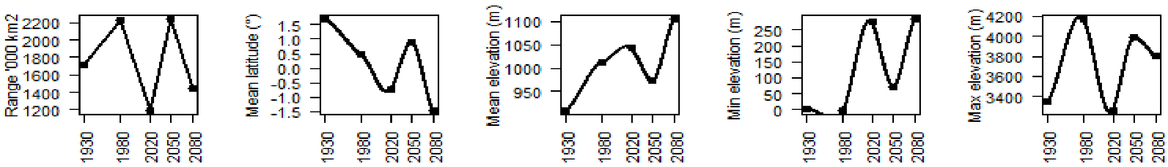

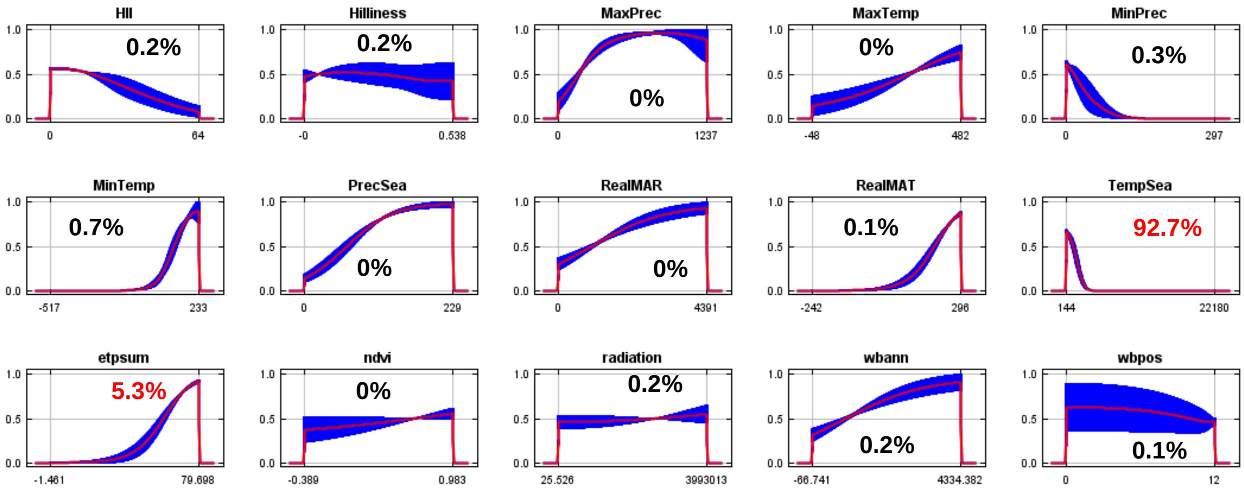

#17 – Tehuantepec jackrabbit (*Lepus flavigularis*)
n = 8
Expert: Arturo Carillo-Reyes, Universidad de Ciencias y Artes de Chiapas
Expert evaluation: Poor
Data: Modern and historic
Envelope: Climatic and habitat
Dispersal distance: 0.01km/year (Expert)
Status: UNMODELLABLE; **Included in final analysis:** X

Model evaluation metric	
AUC	0.99
Omission rate	0.00
Sensitivity	1.00
Specificity	0.99
Proportion correct	0.99
**Kappa**	**0.95**
True Skill Statistic	0.99

Summary: The Tehuantepec jackrabbit's bioclimatic envelope is predicted to decrease by 45% with a ~1° mean latitudinal poleward shift and a mean increase in elevation of ~450m driven by an increase in maximum and minimum elevation. 95% of the permutation importance of the model was contributed to by temperature seasonality (81.7%), mean annual temperature (2.7%) and normalised difference vegetation index (1.7%).

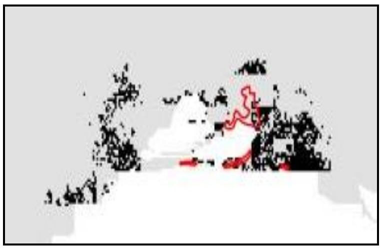
a) ~ 1930s (1900-1949)

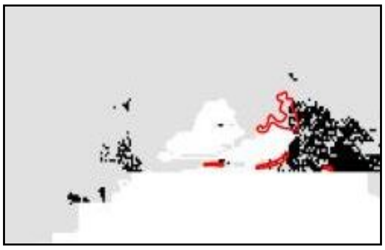
b) ~ 1980s (1950-2000)

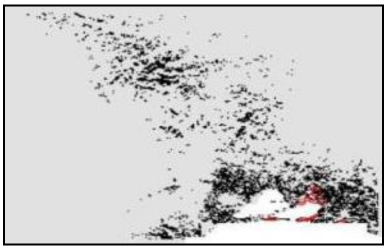
c) ~ 2020s (2010-2039)

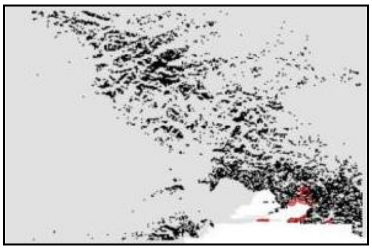
d) ~ 2050s (2040-2069)

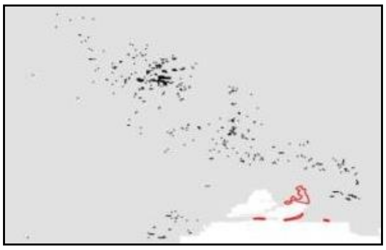
e) ~ 2080s (2070-2099)

Black = predicted suitable envelope

Red polygon = IUCN range (Cervantes, F.A., *et al*. 2008)

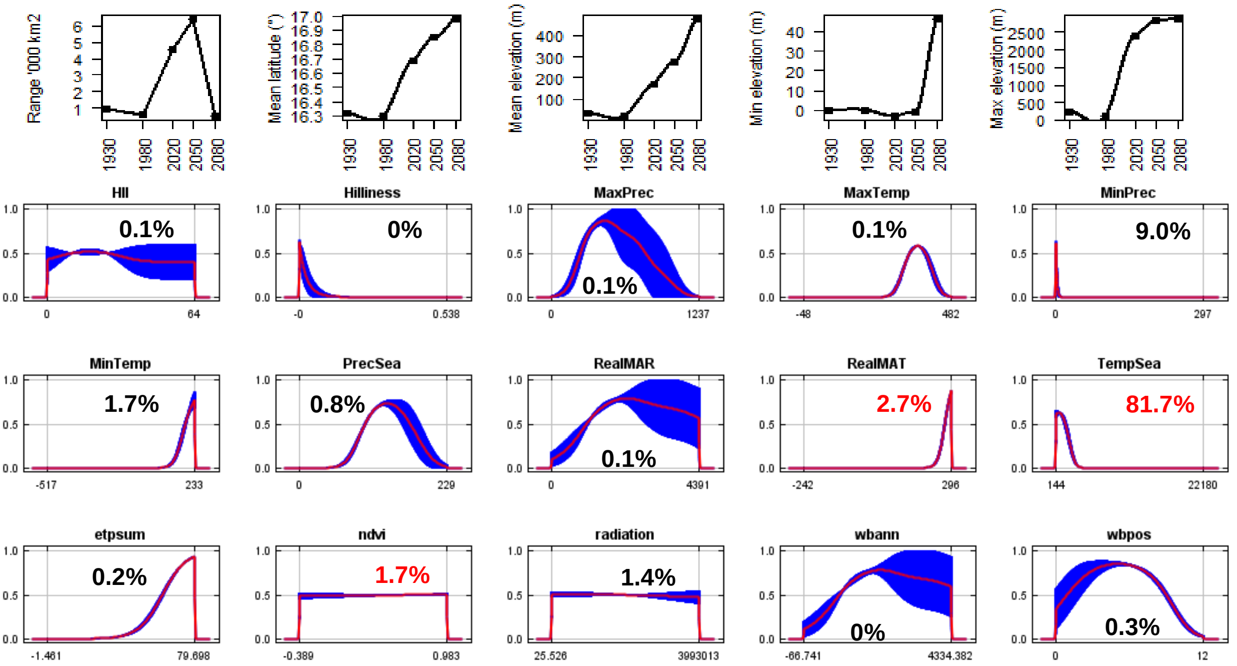

#18 – Iberian hare (*Lepus granatensis*)
n = 1675
Expert: Pelayo Acevedo, University of Porto
Expert evaluation: Medium
Data: Modern and historic
Envelope: Climatic and habitat
Dispersal distance: 7km/year (Expert)
Status: MODELLABLE; **Included in final analysis:** √

Model evaluation metric	
AUC	0.94
Omission rate	0.08
Sensitivity	0.92
Specificity	0.95
Proportion correct	0.95
**Kappa**	**0.81**
True Skill Statistic	0.87

Summary: The Iberian hare's bioclimatic envelope is predicted to increase by 40% with a ~1° mean latitudinal poleward shift and a mean increase in elevation of ~10m driven by an increase in maximum elevation. 95% of the permutation importance of the model was contributed to by maximum precipitation (39.0%), annual evapotranspiration (38.0%), minimum temperature (15.0%) and maximum temperature (3.0%).

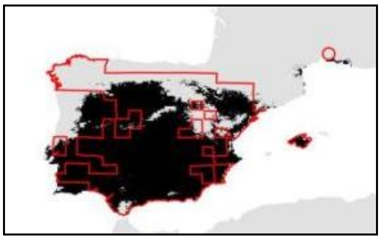
a) ~ 1930s (1900-1949)

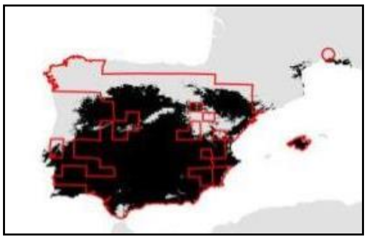
b) ~ 1980s (1950-2000)

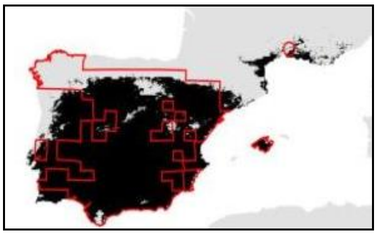
c) ~ 2020s (2010-2039)

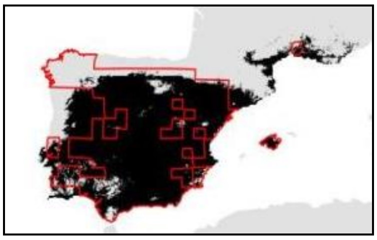
d) ~ 2050s (2040-2069)

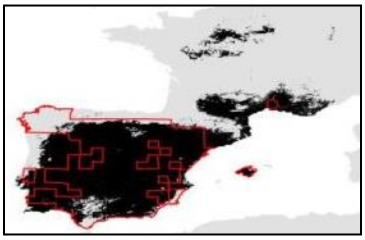
e) ~ 2080s (2070-2099)

Black = predicted suitable envelope

Red polygon = IUCN range (Smith, A.T. & Johnston, C.H. 2008)

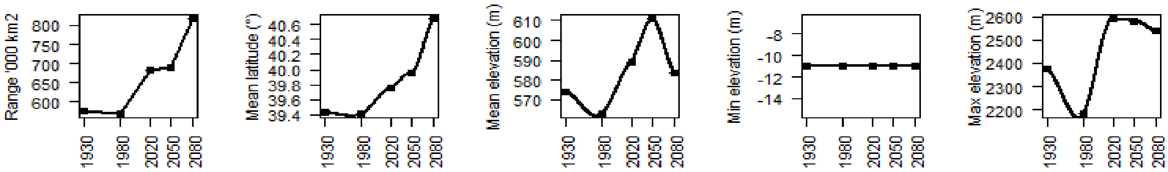

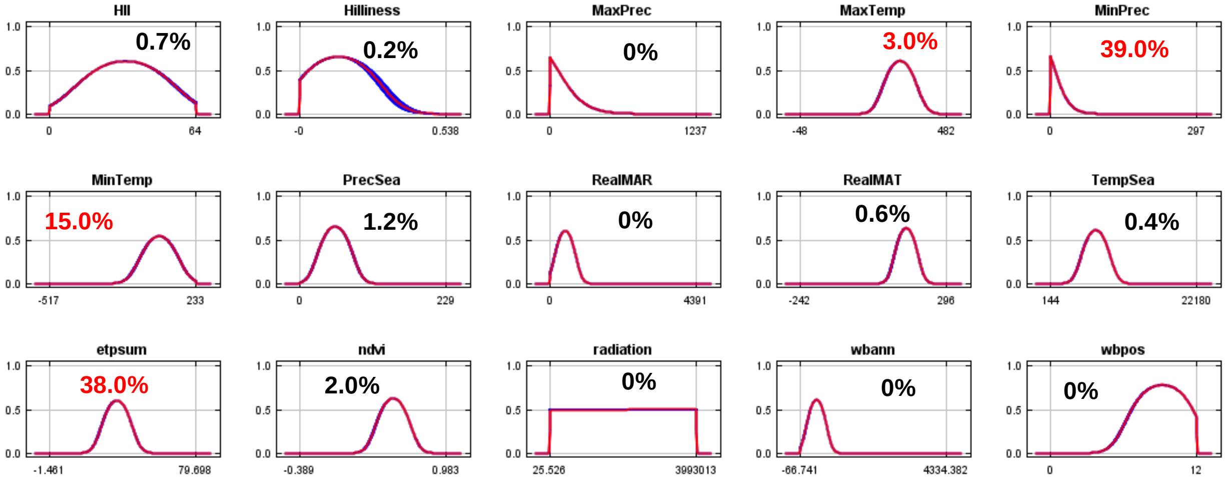

#19 – Abyssinian hare (*Lepus habessinicus*)
n = 7
Expert: Zelalem Tolesa, Addis Ababa University
Expert evaluation: Medium
Data: Modern and historic
Envelope: Climatic and habitat
Dispersal distance: 25km/year (African leporids, range 15-35)
Status: MODELLABLE; **Included in final analysis:** √

Model evaluation metric	
AUC	0.99
Omission rate	0.00
Sensitivity	1.00
Specificity	0.99
Proportion correct	0.99
**Kappa**	**0.82**
True Skill Statistic	0.99

Summary: The Abyssinian hare's bioclimatic envelope is predicted to decrease by 4% with a ~4° mean latitudinal shift towards the Equator and a mean decrease in elevation of ~200m driven by an decrease in maximum elevation. 95% of the permutation importance of the model was contributed to by temperature seasonality (87.6%), annual evapotranspiration (4.9%) and minimum precipitation (4.1%).

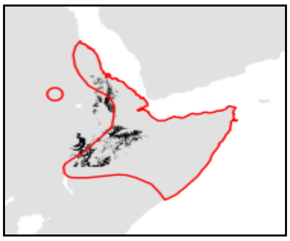

a) ~ 1930s (1900-1949)

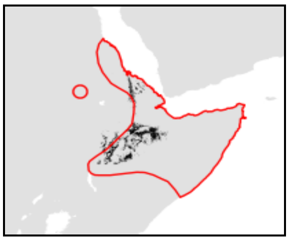

b) ~ 1980s (1950-2000)

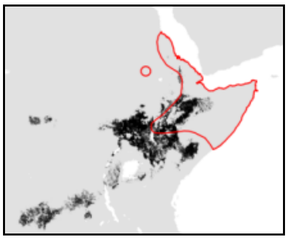

c) ~ 2020s (2010-2039)

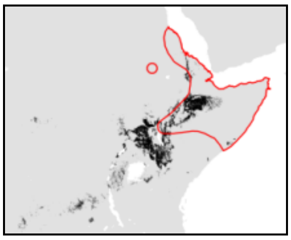

d) ~ 2050s (2040-2069)

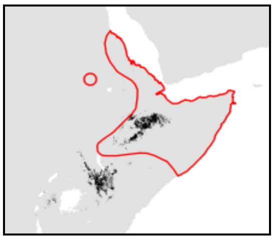

e) ~ 2080s (2070-2099)

Black = predicted suitable envelope

Red polygon = IUCN range (Smith, A.T. & Johnston, C.H. 2008)

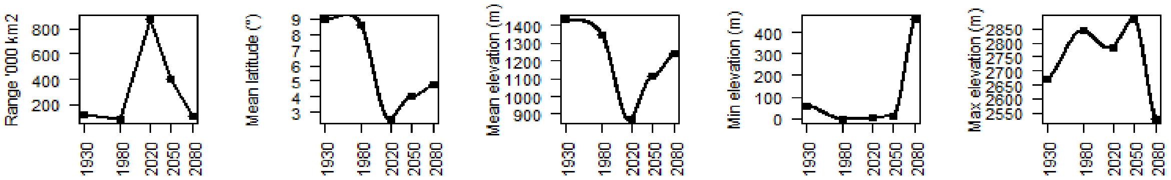

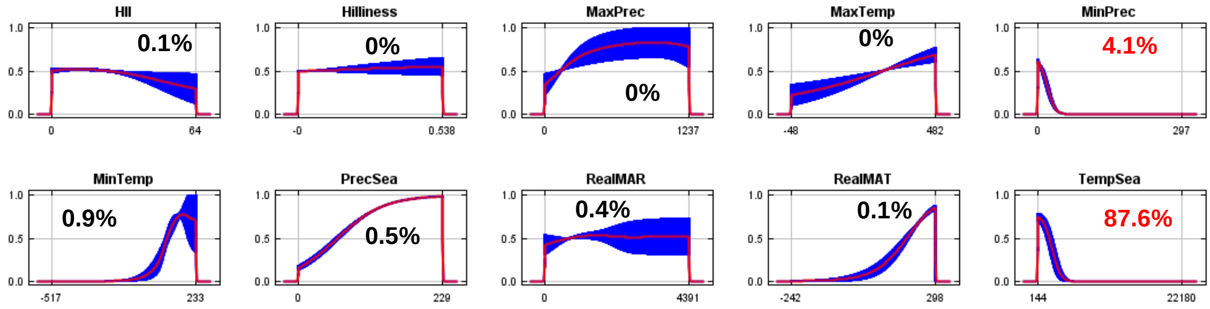

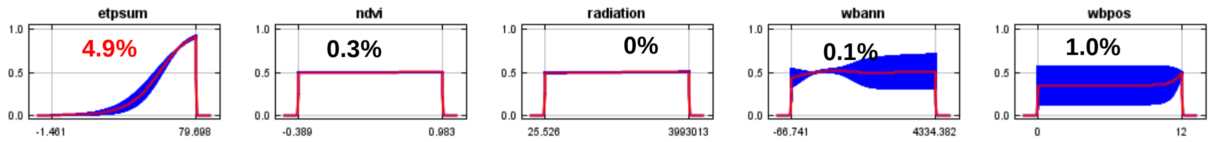

#20 – Hainan hare (*Lepus hainanus*)
n = 9
Expert: Youhua Chen, Wuhan University, China
Expert evaluation: Good
Data: Modern and historic
Envelope: Climatic and habitat
Dispersal distance: 0.01km/year (Island species, range 0.01-0.01)
Status: MODELLABLE; **Included in final analysis:** √

Model evaluation metric	
AUC	0.99
Omission rate	0.00
Sensitivity	1.00
Specificity	0.99
Proportion correct	0.99
**Kappa**	**0.86**
True Skill Statistic	0.99

Summary: The Hainan hare's bioclimatic envelope is predicted to increase by 4% with no latitudinal poleward shift and a mean increase in elevation of ~20m driven by an increase in maximum elevation. 95% of the permutation importance of the model was contributed to by minimum temperature (71.0%), mean annual temperature (22.8%) and temperature seasonality (5.4%).

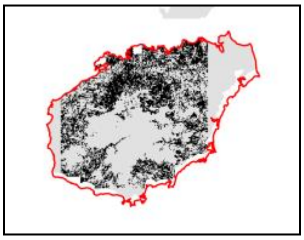
a) ~ 1930s (1900-1949)

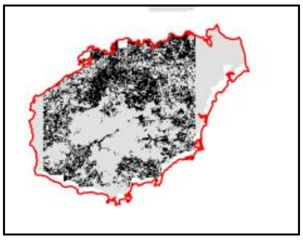
b) ~ 1980s (1950-2000)

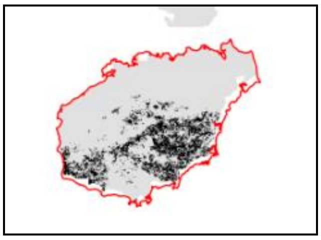
c) ~ 2020s (2010-2039)

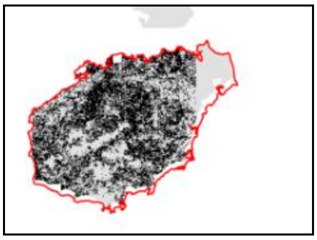
d) ~ 2050s (2040-2069)

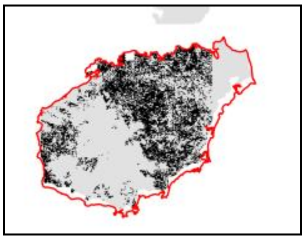
e) ~ 2080s (2070-2099)

Black = predicted suitable envelope

Red polygon = IUCN range (Lazell, J.,*et al*. 2008)

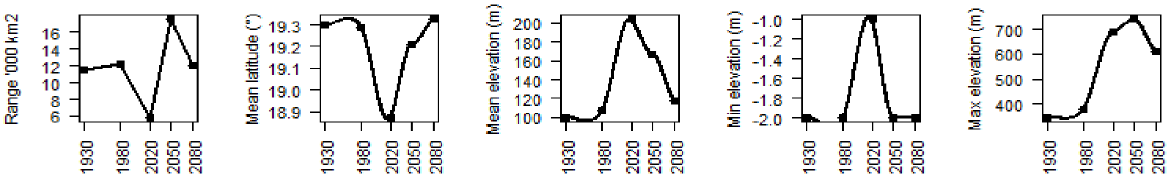

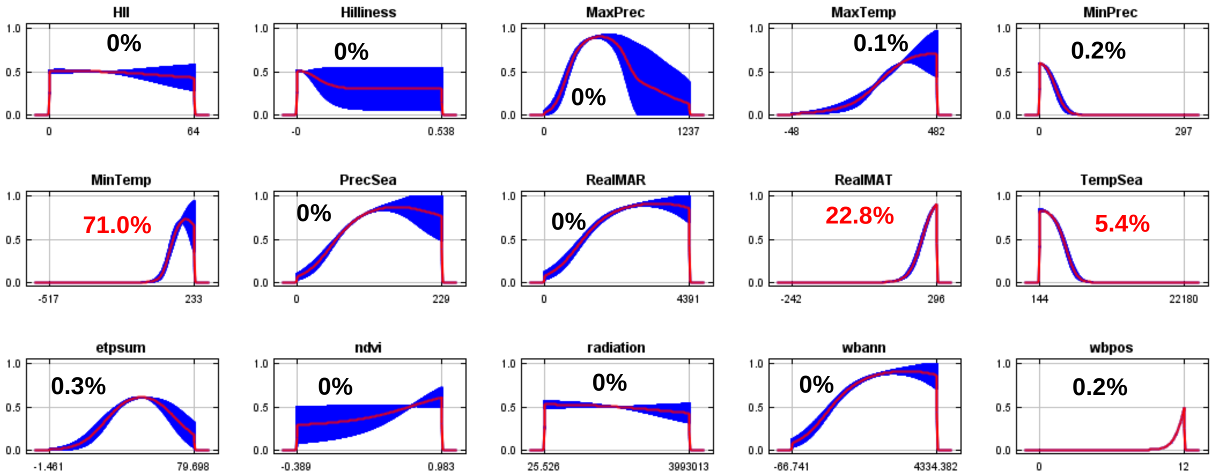

#21 – Black jackrabbit (*Lepus insularis*)
n = 3
Expert: Tamara Rioja Pardela, Universidad de Ciencias y Artes de Chiapas, Mexico
Expert evaluation: Good
Data: Modern and historic
Envelope: Climatic and habitat
Dispersal distance: 0.01km/year (Island species, range 0.01-0.01)
Status: MODELLABLE; **Included in final analysis:** √

Model evaluation metric	
AUC	1.00
Omission rate	0.00
Sensitivity	1.00
Specificity	1.00
Proportion correct	1.00
**Kappa**	**1.00**
True Skill Statistic	1.00

Summary: The Black jackrabbit's bioclimatic envelope is predicted to decrease by 100% with a ~0.3° mean latitudinal polewards shift and a mean increase in elevation of ~50m driven by an increase in both minimum and maximum elevation. 95% of the permutation importance of the model was contributed to by precipitation seasonality (28.6%), minimum precipitation (16.5%), annual water balance (13.3%), minimum temperature (12.9%), mean annual temperature (7.1%), temperature seasonality (5.5%), surface roughness index (5.0%) and normalised difference vegetation index (3.2%).

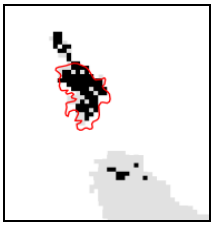
a) ~ 1930s (1900-1949)

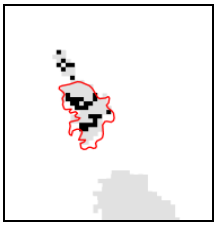
b) ~ 1980s (1950-2000)

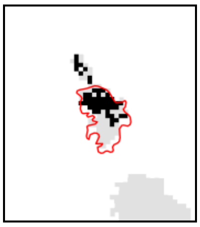
c) ~ 2020s (2010-2039)

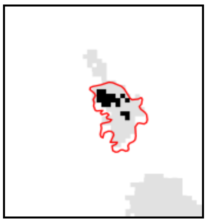
d) ~ 2050s (2040-2069)

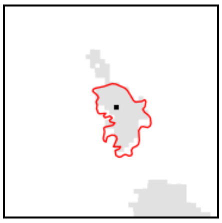
e) ~ 2080s (2070-2099)

Black = predicted suitable envelope

Red polygon = IUCN range (Romero Malpica, F.J. & Rangel Cordero, H. 2008)

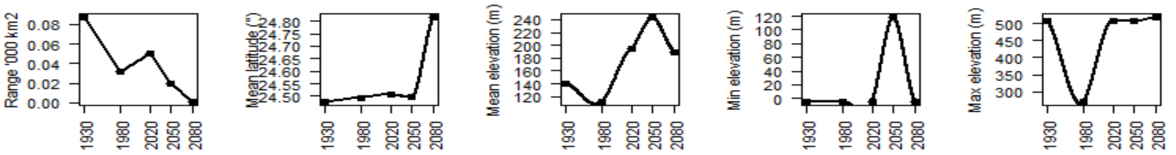

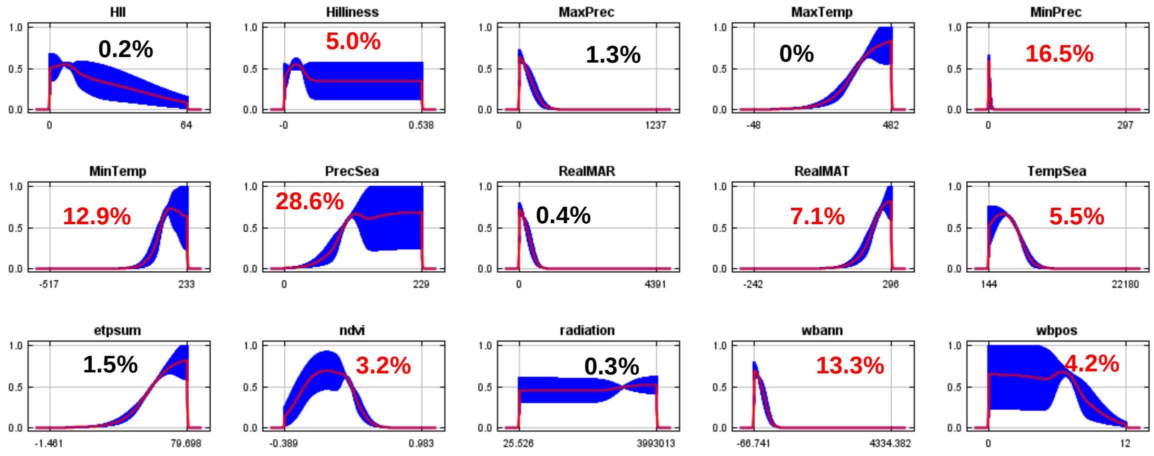

#22 – Manchurian hare (*Lepus mandshuricus*)
n = 36
Expert: Deyan Ge, Institute of Zoology, Chinese Academy of Sciences
Expert evaluation: Medium
Data: Modern and historic
Envelope: Climatic and habitat
Dispersal distance: 3km/year (Sokolov, V.E. *et al*., 2009)
Status: MODELLABLE; **Included in final analysis:** √

Model evaluation metric	
AUC	0.96
Omission rate	0.08
Sensitivity	0.92
Specificity	0.99
Proportion correct	0.99
**Kappa**	**0.78**
True Skill Statistic	0.92

Summary: The Manchurian hare’s bioclimatic envelope is predicted to decrease by 50% with a ~1° mean latitudinal polewards shift and a mean increase in elevation of ~70m driven by an increase in maximum and minimum elevation. 95% of the permutation importance of the model was contributed to by precipitation seasonality (77.3%), mean annual temperature (10.4%), minimum temperature (4.5%), normalised difference vegetation index (2.6%) and annual evapotranspiration (2.4%).

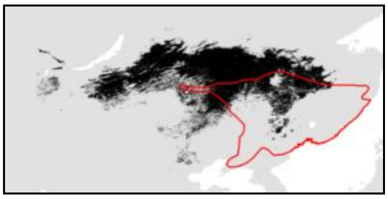
a) ~ 1930s (1900-1949)

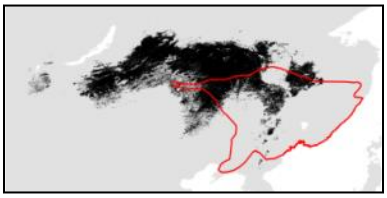
b) ~ 1980s (1950-2000)

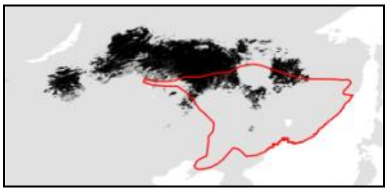
c) ~ 2020s (2010-2039)

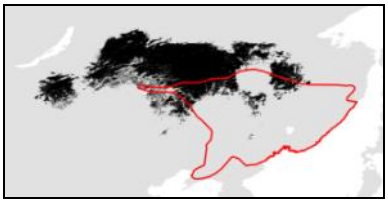
d) ~ 2050s (2040-2069)

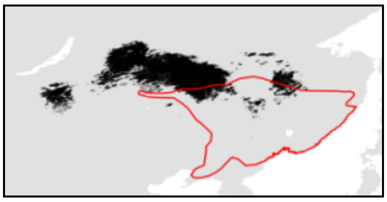
e) ~ 2080s (2070-2099)

Black = predicted suitable envelope

Red polygon = IUCN range (Smith, A.T. & Johnston, C.H. 2008)

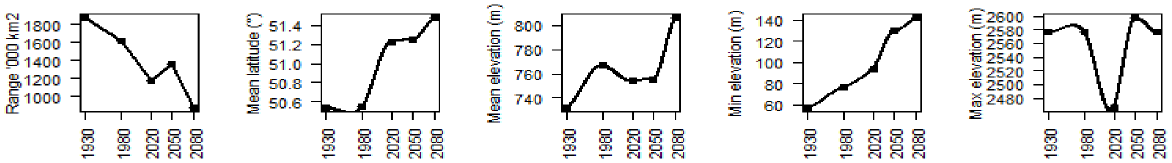

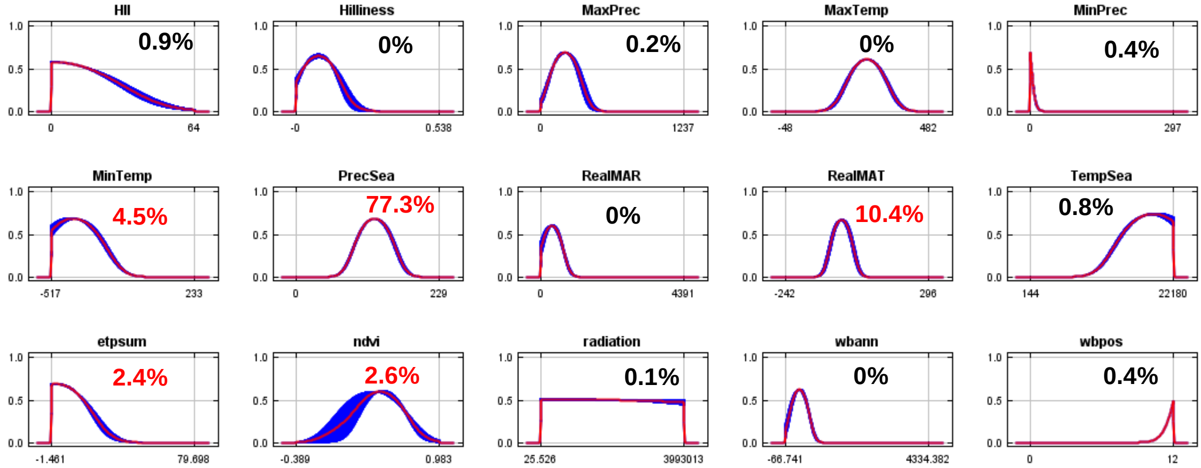

#23 – African savannah hare (*Lepus microtis*)
n = 82
Expert: John Flux, IUCN Lagomorph Specialist Group
Expert evaluation: Medium
Data: Modern and historic
Envelope: Climatic only
Dispersal distance: 15km/year (Expert)
Status: MODELLABLE; **Included in final analysis:** √

Model evaluation metric	
AUC	0.93
Omission rate	0.13
Sensitivity	0.87
Specificity	0.99
Proportion correct	0.99
**Kappa**	**0.62**
True Skill Statistic	0.86

Summary: The African savannah hare's bioclimatic envelope is predicted to decrease by 15% with a ~3° mean latitudinal polewards shift and a mean increase in elevation of ~100m driven by an increase in maximum and minimum elevation. 95% of the permutation importance of the model was contributed to by mean annual temperature (40.9%), maximum temperature (18.5%), annual evapotranspiration (9.2%), minimum precipitation (6.2%), temperature seasonality (5.4%), mean annual precipitation (5.4%), minimum temperature (4.9%), precipitation seasonality (4.1%) and annual water balance (1.7%).

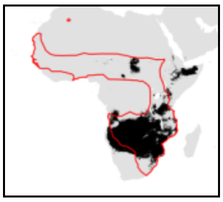

a) ~ 1930s (1900-1949)

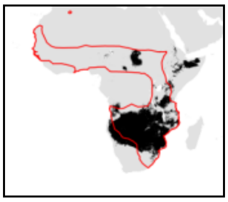

b) ~ 1980s (1950-2000)

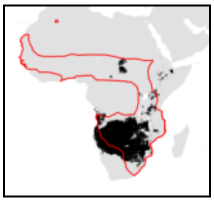

c) ~ 2020s (2010-2039)

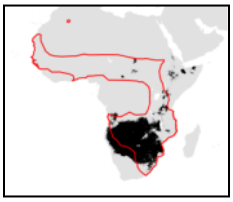

d) ~ 2050s (2040-2069)

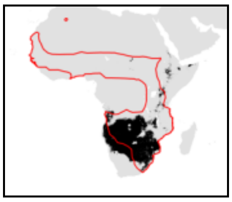

e) ~ 2080s (2070-2099)

Black = predicted suitable envelope

Red polygon = IUCN range (Smith, A.T. & Johnston, C.H. 2008)

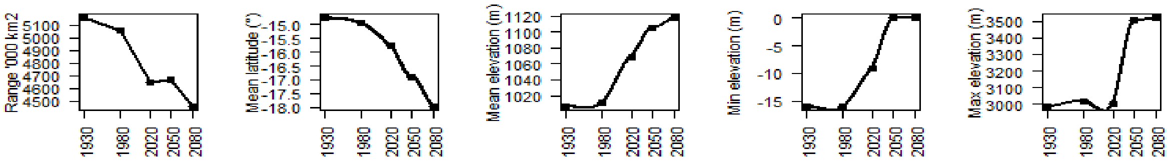

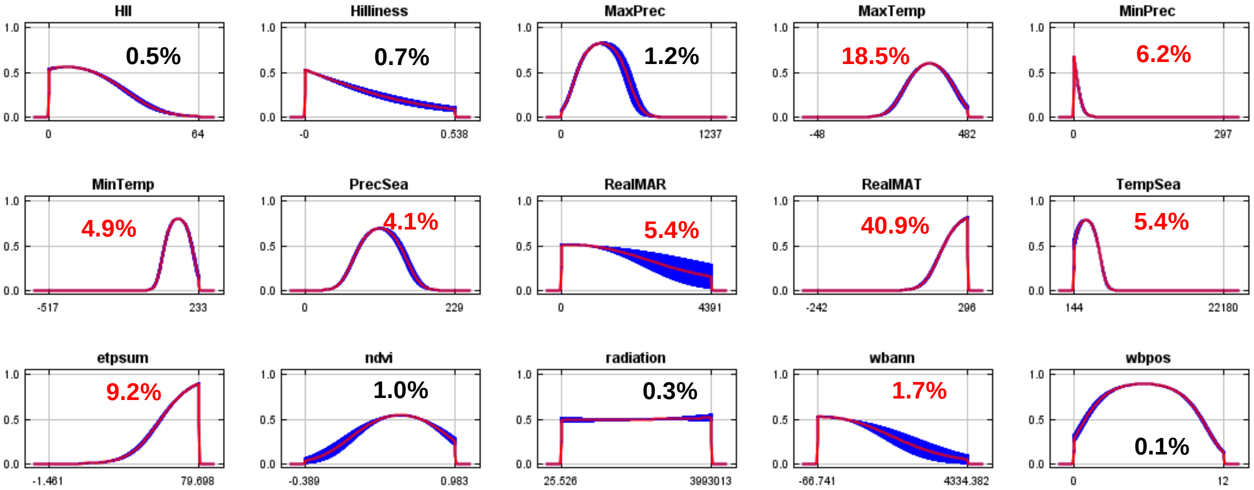

#24 – Indian hare (*Lepus nigricollis*)
n = 17
Expert: Gopinathan Maheswaran , Zoological Survey of India
Expert evaluation: Good
Data: Modern and historic
Envelope: Climatic and habitat
Dispersal distance: 6km/year (Expert)
Status: MODELLABLE; **Included in final analysis:** √

Model evaluation metric	
AUC	0.99
Omission rate	0.00
Sensitivity	1.00
Specificity	0.99
Proportion correct	0.99
**Kappa**	**0.59**
True Skill Statistic	0.99

Summary: The Indian hare’s bioclimatic envelope is predicted to decrease by 10% with a ~2° mean latitudinal polewards shift and a mean increase in elevation of ~80m driven by an increase in maximum and minimum elevation. 95% of the permutation importance of the model was contributed to by precipitation seasonality (48.0%), mean annual temperature (14.9%), human influence index (11.9%), minimum temperature (10.3%), temperature seasonality (7.0%), number of months with a positive water balance (2.8%) and minimum precipitation (1.5%).

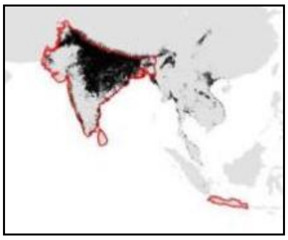
a) ~ 1930s (1900-1949)

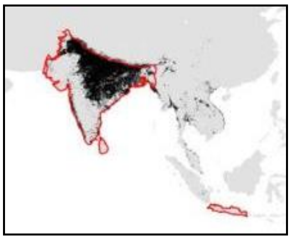
b) ~ 1980s (1950-2000)

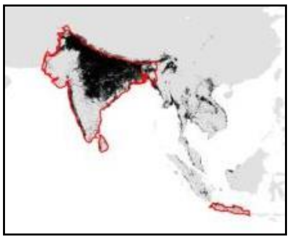
c) ~ 2020s (2010-2039)

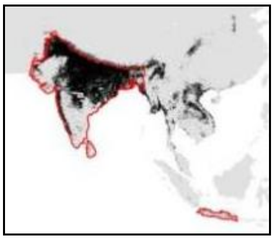
d) ~ 2050s (2040-2069)

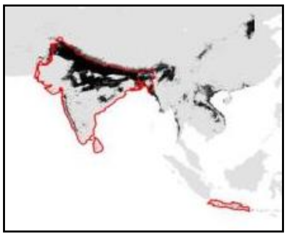
e) ~ 2080s (2070-2099)

Black = predicted suitable envelope

Red polygon = IUCN range (Maheswaran, G. & Jordan, M. 2008)

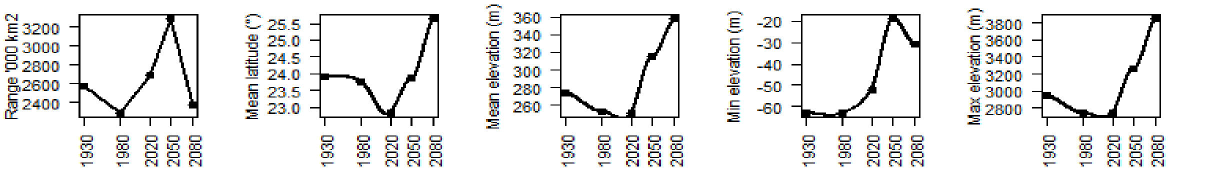

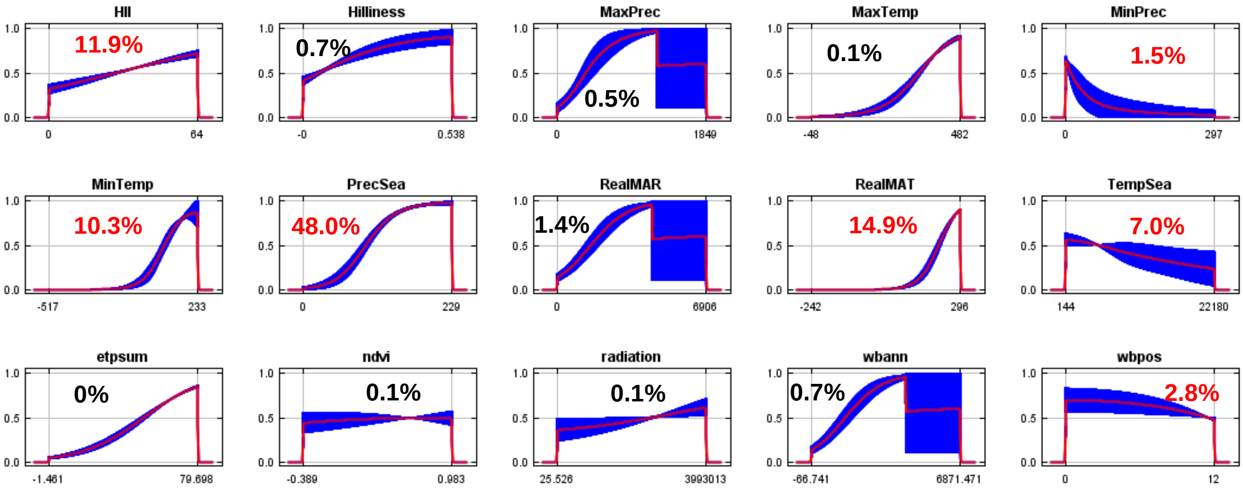

#25 – Woolly hare (*Lepus oiostolus*)
n = 84
Expert: Weihe Yang , Institute of Zoology, Chinese Academy of Sciences
Expert evaluation: Medium
Data: Only modern
Envelope: Climatic and habitat
Dispersal distance: 2.5km/year (Asian leporids, range 1-35)
Status: MODELLABLE; **Included in final analysis:** √

Model evaluation metric	
AUC	0.94
Omission rate	0.11
Sensitivity	0.89
Specificity	0.99
Proportion correct	0.99
**Kappa**	**0.63**
True Skill Statistic	0.89

Summary: The Woolly hare's bioclimatic envelope is predicted to decrease by 25% with a ~1° mean latitudinal shift towards the Equator and a mean increase in elevation of ~680m driven by an increase in maximum and minimum elevation. 95% of the permutation importance of the model was contributed to by minimum precipitation (82.1%), maximum temperature (5.0%), minimum temperature (4.6%) and annual evapotranspiration (3.8%).

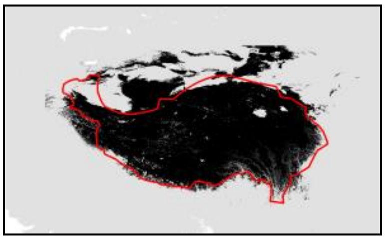
a) ~ 1930s (1900-1949)

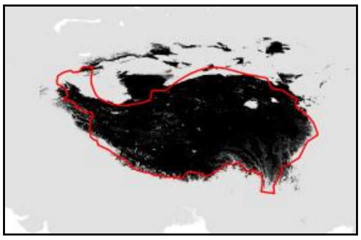
b) ~ 1980s (1950-2000)

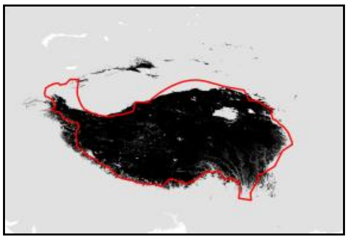
c) ~ 2020s (2010-2039)

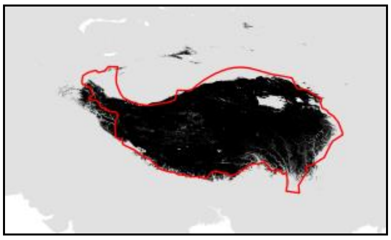
d) ~ 2050s (2040-2069)

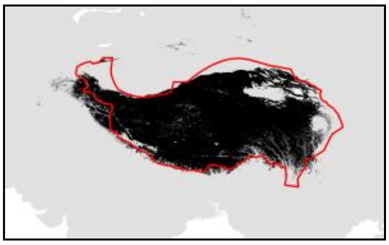
e) ~ 2080s (2070-2099)

Black = predicted suitable envelope

Red polygon = IUCN range (Indian CAMP Workshop & Johnston, C.H. 2008)

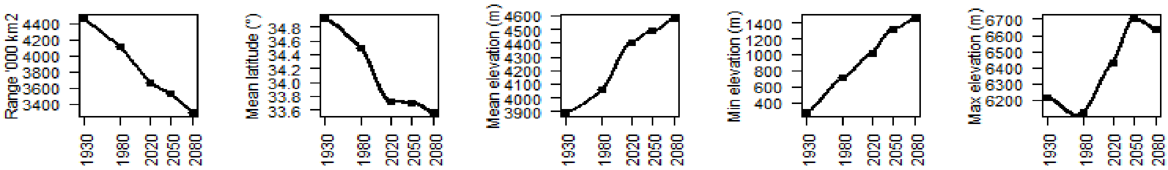

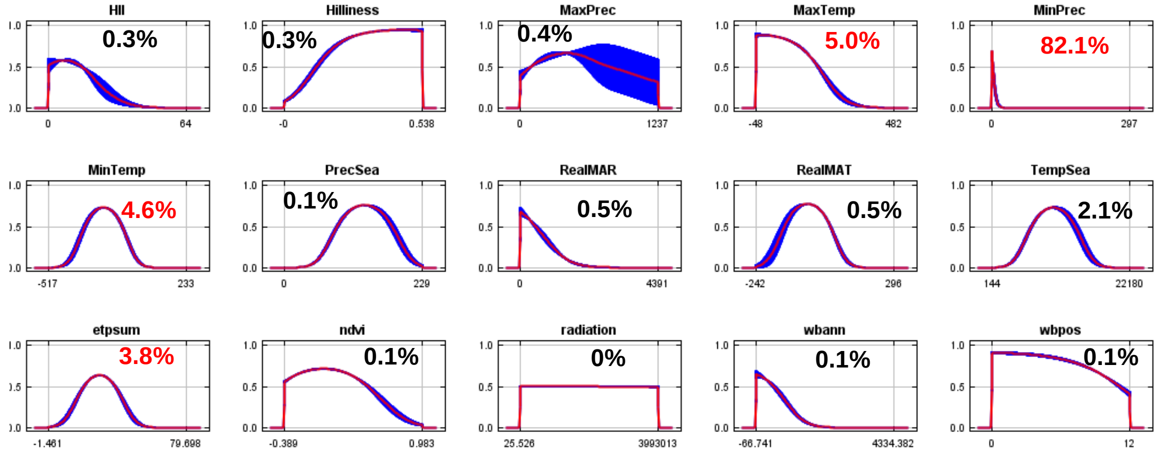

#26 – Alaskan hare (*Lepus othus*)
n = 8
Expert: Eric Waltari, City University of New York
Expert evaluation: Medium
Data: Modern and historic
Envelope: Climatic only
Dispersal distance: 2km/year (similar to Arctic hare)
Status: MODELLABLE; **Included in final analysis:** √

Model evaluation metric	
AUC	0.99
Omission rate	0.00
Sensitivity	1.00
Specificity	0.99
Proportion correct	0.99
**Kappa**	**0.89**
True Skill Statistic	0.99

Summary: The Alaskan hare's bioclimatic envelope is predicted to increase by 80% with a ~3° mean latitudinal polewards shift and a mean increase in elevation of ~100m driven by an increase in minimum elevation. 95% of the permutation importance of the model was contributed to by annual evapotranspiration (52.8%), mean annual temperature (26.4%), precipitation seasonality (11.3%) and mean annual precipitation (5.0%).

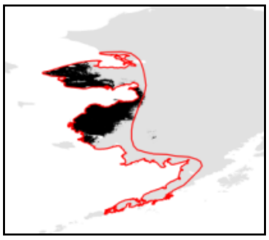

a) ~ 1930s (1900-1949)

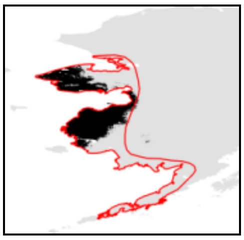

b) ~ 1980s (1950-2000)

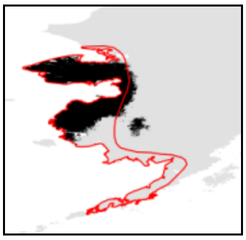

c) ~ 2020s (2010-2039)

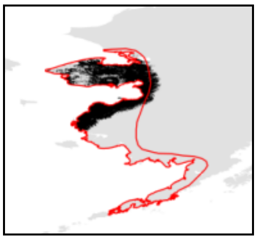

d) ~ 2050s (2040-2069)

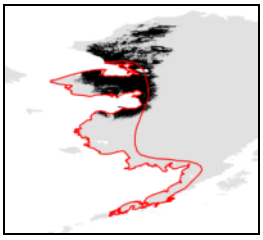

e) ~ 2080s (2070-2099)

Black = predicted suitable envelope

Red polygon = IUCN range (Murray, D. & Smith, A.T. 2008)

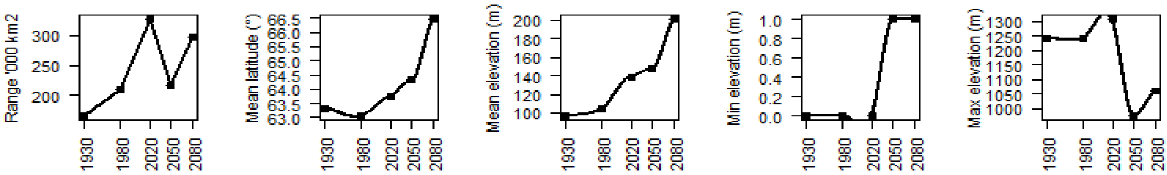

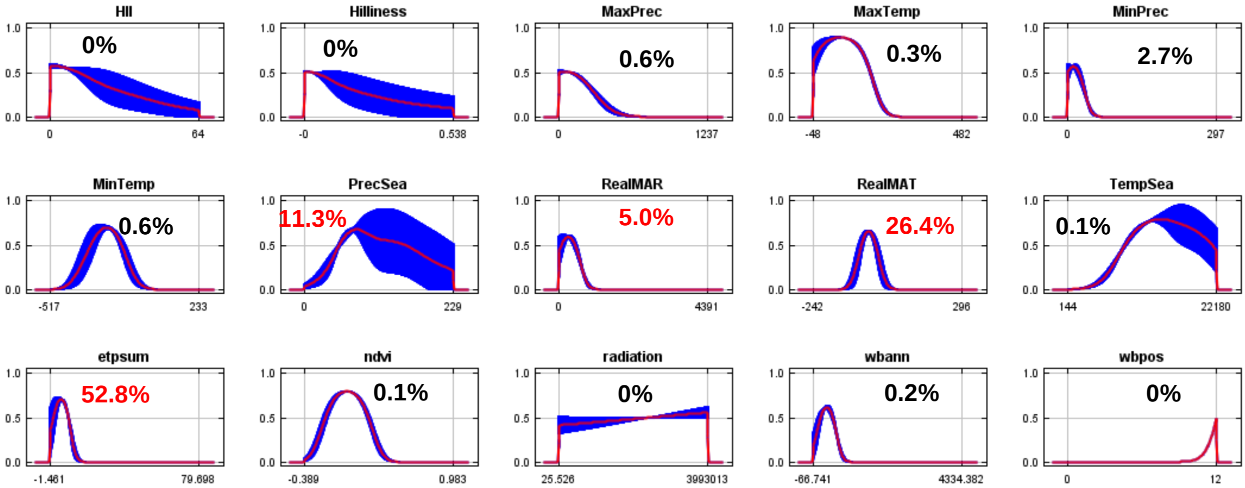

#27 – Burmese hare (*Lepus peguensis*)
n = 7
Expert: Thomas Gray, WWF Greater Mekong
Expert evaluation: Medium
Data: Modern and historic
Envelope: Climatic and habitat
Dispersal distance: 2.5km/year (Asian leporids, range 1-35)
Status: MODELLABLE; **Included in final analysis:** √

Model evaluation metric	
AUC	0.99
Omission rate	0.00
Sensitivity	1.00
Specificity	0.99
Proportion correct	0.99
**Kappa**	**0.58**
True Skill Statistic	0.99

Summary: The Burmese hare's bioclimatic envelope is predicted to increase by 40% with a ~2° mean latitudinal polewards shift and a mean increase in elevation of ~180m driven by an increase in minimum and maximum elevation. 95% of the permutation importance of the model was contributed to by temperature seasonality (52.6%), minimum precipitation (32.9%), solar radiation (6.5%) and mean annual temperature (4.3%).

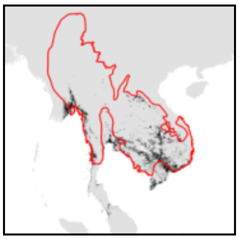
a) ~ 1930s (1900-1949)

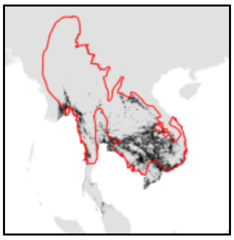
b) ~ 1980s (1950-2000)

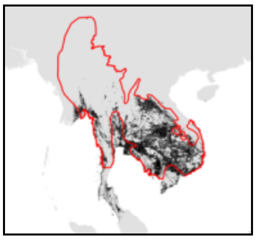
c) ~ 2020s (2010-2039)

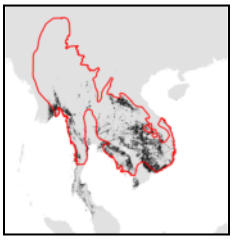
d) ~ 2050s (2040-2069)

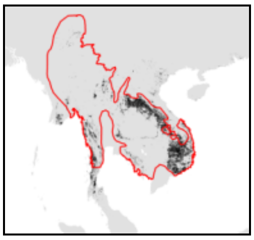
e) ~ 2080s (2070-2099)

Black = predicted suitable envelope

Red polygon = IUCN range (Duckworth, J.W., *et al*. 2008)

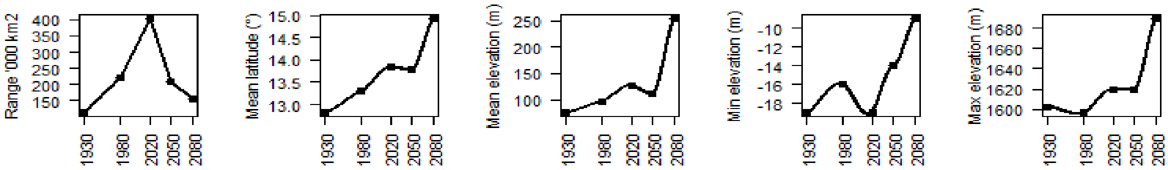

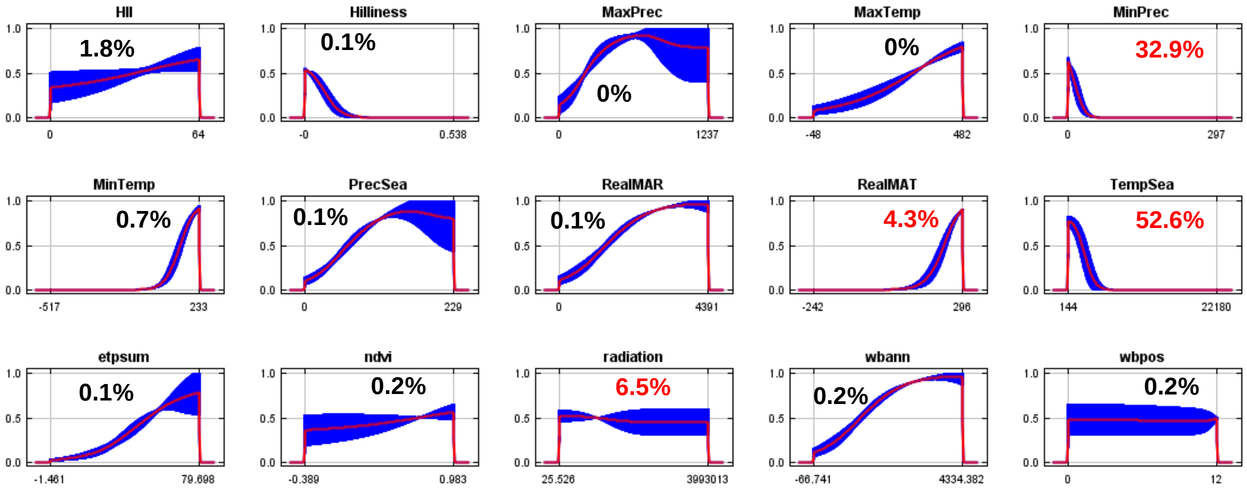

#28 – Scrub hare (*Lepus saxatilis*)
n = 39
Expert: Kai Collins, University of Pretoria
Expert evaluation: Poor
Data: Only modern
Envelope: Climatic and habitat
Dispersal distance: 25km/year (African leporids, range 15-35)
Status: UNMODELLABLE; **Included in final analysis:** X

Model evaluation metric	
AUC	0.95
Omission rate	0.08
Sensitivity	0.92
Specificity	0.97
Proportion correct	0.97
**Kappa**	**0.18**
True Skill Statistic	0.89

Summary: The Scrub hare's bioclimatic envelope is predicted to decrease by 15% with a ~2° mean latitudinal shift towards the Equator and a mean increase in elevation of ~65m driven by an increase in maximum and minimum elevation. 95% of the permutation importance of the model was contributed to by annual evapotranspiration (52.8%), mean annual temperature (26.4%), precipitation seasonality (11.3%) and mean annual precipitation (5.0%).

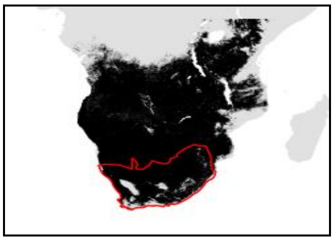
a) ~ 1930s (1900-1949)

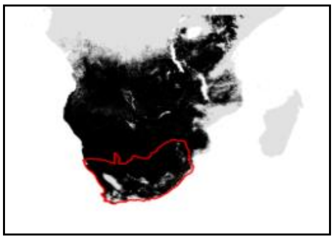
b) ~ 1980s (1950-2000)

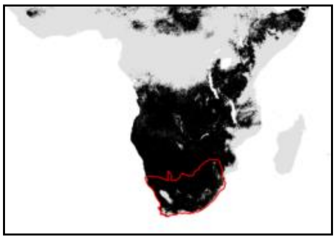
c) ~ 2020s (2010-2039)

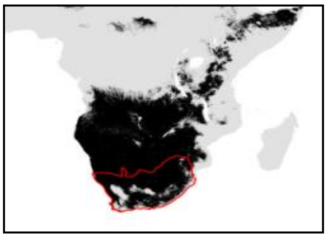
d) ~ 2050s (2040-2069)

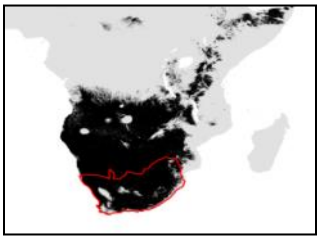
e) ~ 2080s (2070-2099)

Black = predicted suitable envelope

Red polygon = IUCN range (Collins, K., *et al*, 2008)

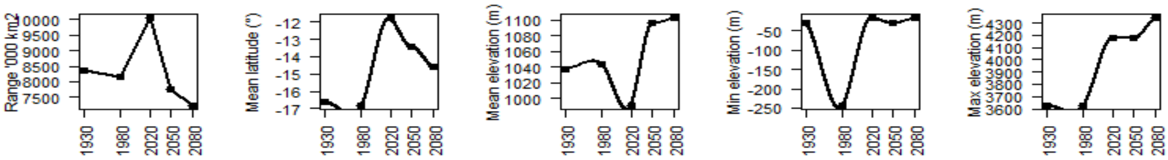

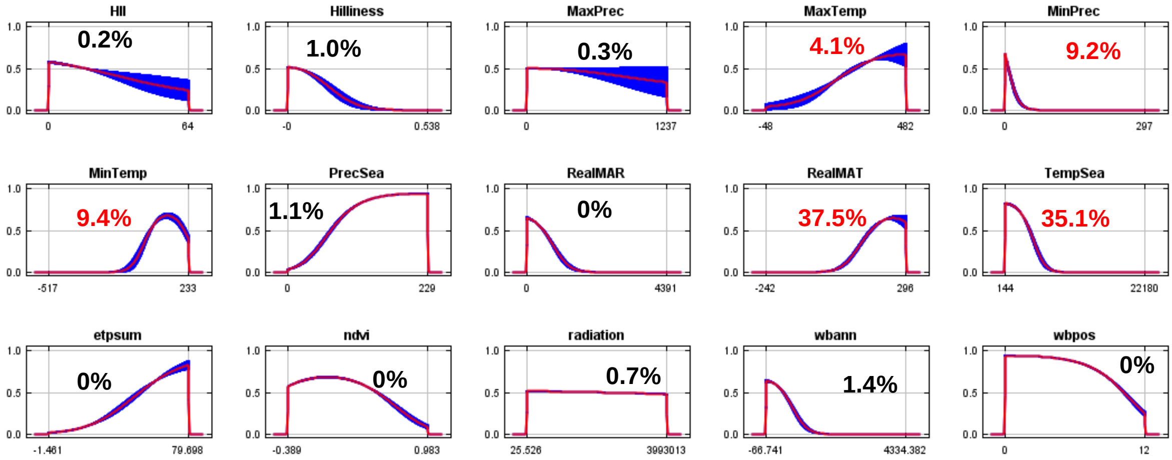

#29 – Chinese hare (*Lepus sinensis*)
n = 141
Expert: Weihe Yang, Institute of Zoology, Chinese Academy of Sciences
Expert evaluation: Medium
Data: Modern and historic
Envelope: Climatic and habitat
Dispersal distance: 2.5km/year (Asian leporids, range 1-35)
Status: MODELLABLE; **Included in final analysis:** √

Model evaluation metric	
AUC	0.94
Omission rate	0.11
Sensitivity	0.89
Specificity	0.99
Proportion correct	0.99
**Kappa**	**0.81**
True Skill Statistic	0.89

Summary: The Chinese hare's bioclimatic envelope is predicted to increase by 60% with a ~2° mean latitudinal polewards shift and a mean increase in elevation of ~25m driven by an increase in maximum elevation. 95% of the permutation importance of the model was contributed to by mean annual temperature (56.5%), temperature seasonality (22.9%), precipitation seasonality (8.0%), mean annual precipitation (5.4%) and annual evapotranspiration (3.5%).

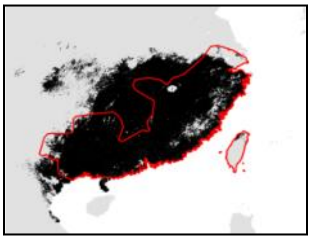
a) ~ 1930s (1900-1949)

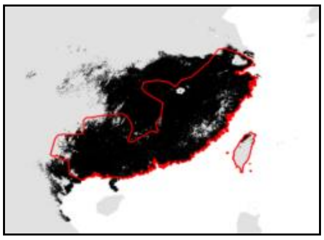
b) ~ 1980s (1950-2000)

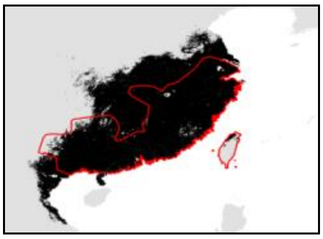
c) ~ 2020s (2010-2039)

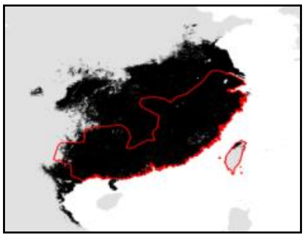
d) ~ 2050s (2040-2069)

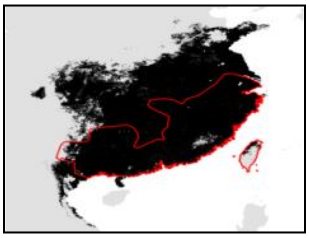
e) ~ 2080s (2070-2099)

Black = predicted suitable envelope

Red polygon = IUCN range (Smith, A.T. & Johnston, C.H. 2008)

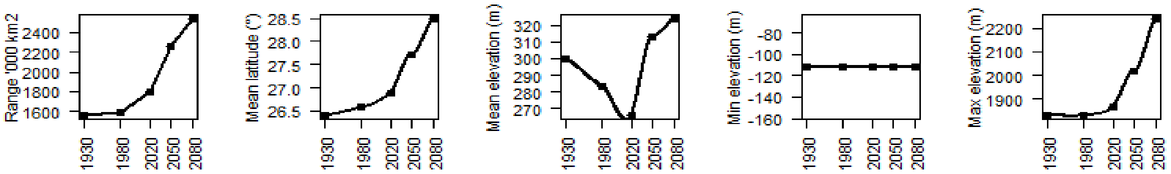

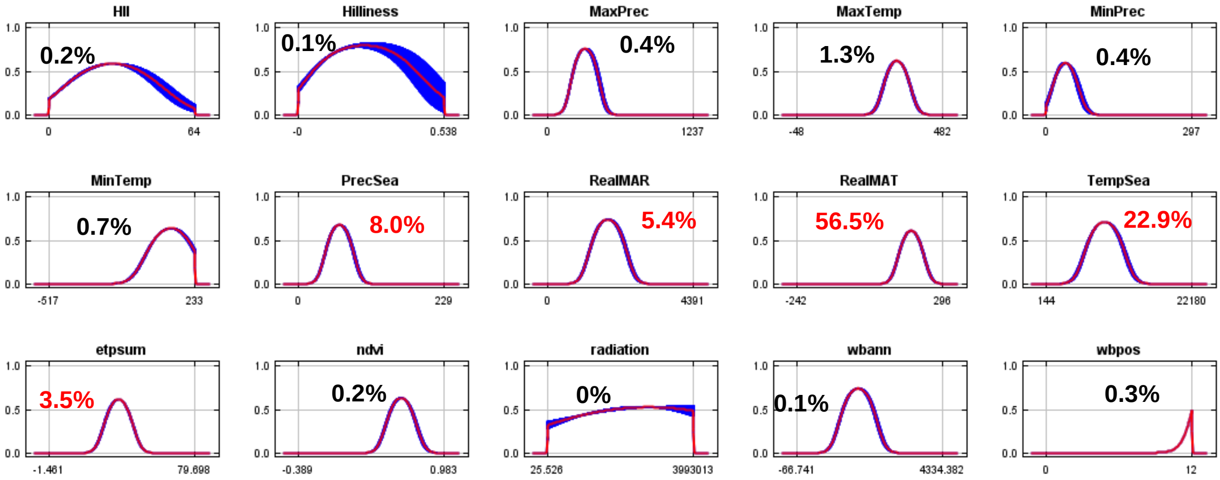

#30 – Ethiopian highland hare (*Lepus starcki*)
n = 13
Expert: Zelalem Tolesa, Addis Ababa University
Expert evaluation: Medium
Data: Modern and historic
Envelope: Climatic and habitat
Dispersal distance: 25km/year (African leporids, range 15-35)
Status: MODELLABLE; **Included in final analysis:** √

Model evaluation metric	
AUC	0.96
Omission rate	0.08
Sensitivity	0.92
Specificity	0.99
Proportion correct	0.99
**Kappa**	**0.86**
True Skill Statistic	0.92

Summary: The Ethiopian highland hare's bioclimatic envelope is predicted to decrease by 90% with a ~7° mean latitudinal shift towards the Equator and a mean decrease in elevation of ~140m driven by a decrease in maximum elevation. 95% of the permutation importance of the model was contributed to by temperature seasonality (80.6%) and minimum temperature (18.5%).

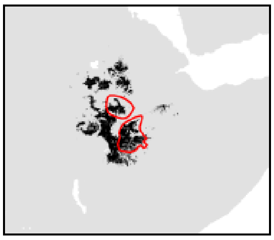

a) ~ 1930s (1900-1949)

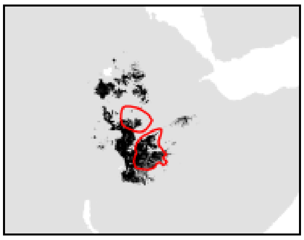

b) ~ 1980s (1950-2000)

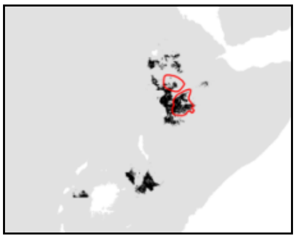

c) ~ 2020s (2010-2039)

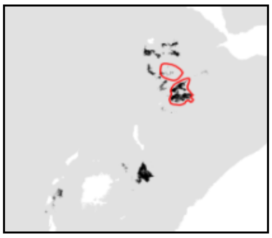

d) ~ 2050s (2040-2069)

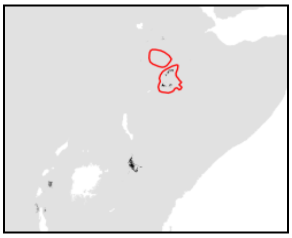

e) ~ 2080s (2070-2099)

Black = predicted suitable envelope

Red polygon = IUCN range (Smith, A.T. & Johnston, C.H. 2008)

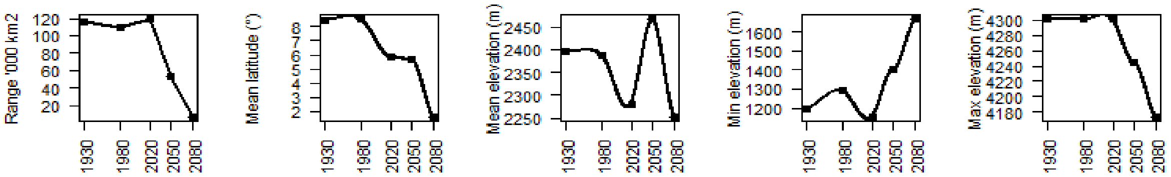

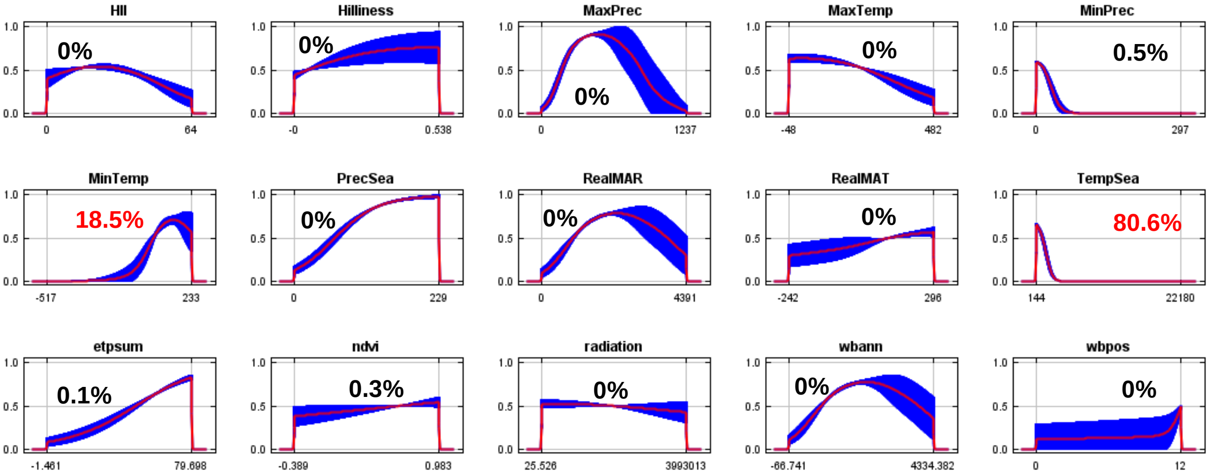

#31 – Desert hare (*Lepus tibetanus*)
n = 55
Expert: Chelmala Srinivasulu, Osmania University, India
Expert evaluation: Medium
Data: Only modern
Envelope: Climatic and habitat
Dispersal distance: 2.5km/year (Asian leporids, range 1-35)
Status: MODELLABLE; **Included in final analysis:** √

Model evaluation metric	
AUC	0.92
Omission rate	0.15
Sensitivity	0.85
Specificity	0.99
Proportion correct	0.99
**Kappa**	**0.57**
True Skill Statistic	0.85

Summary: The Desert hare's bioclimatic envelope is predicted to decrease by 50% with no latitudinal shift towards the Equator, but a mean increase in elevation of ~320m driven by an increase in maximum elevation. 95% of the permutation importance of the model was contributed to by minimum precipitation (54.5%), minimum temperature (19.3%), maximum precipitation (17.3%), annual evapotranspiration (2.8%) and human influence index (1.7%).

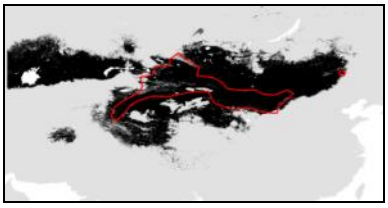

a) ~ 1930s (1900-1949)

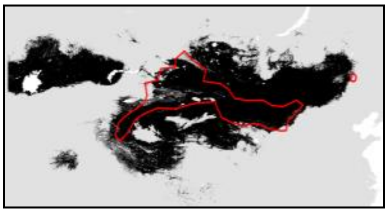

b) ~ 1980s (1950-2000)

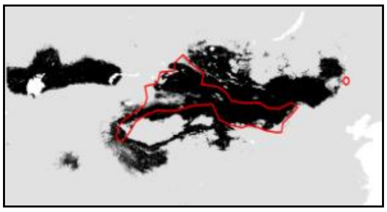

c) ~ 2020s (2010-2039)

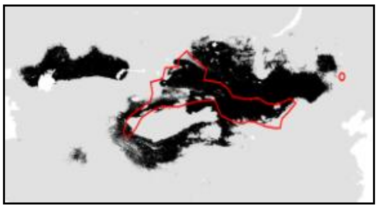

d) ~ 2050s (2040-2069)

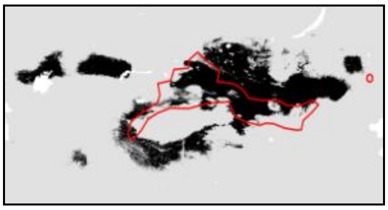

e) ~ 2080s (2070-2099)

Black = predicted suitable envelope

Red polygon = IUCN range (China Red List & Johnston, C.H. 2008)

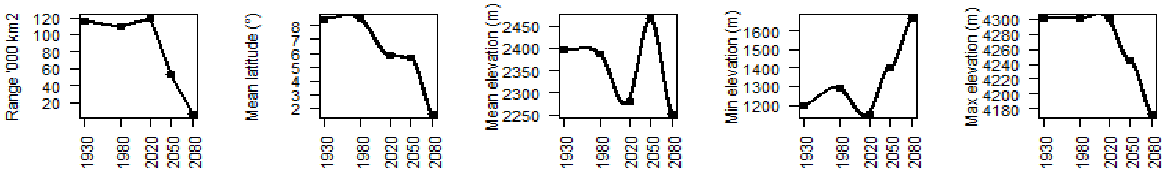

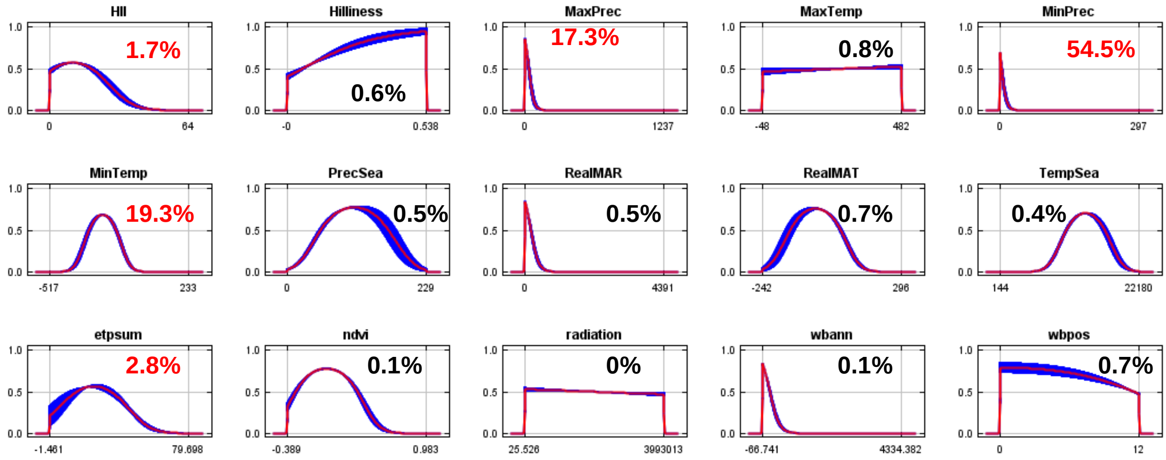

#32 – Mountain hare (*Lepus timidus*) – Eurasian populations
n = 2,460
Expert: Neil Reid, Queen's University Belfast
Expert evaluation: Medium
Data: Only modern
Envelope: Climatic and habitat
Dispersal distance: 2km/year (Expert)
Status: MODELLABLE; **Included in final analysis:** √

Model evaluation metric	
AUC	0.91
Omission rate	0.08
Sensitivity	0.92
Specificity	0.90
Proportion correct	0.91
**Kappa**	**0.74**
True Skill Statistic	0.82

Summary: The Mountain hare's bioclimatic envelope is predicted to decrease by 10% with a ~4° mean latitudinal polewards shift and a mean decrease in elevation of ~10m driven by a decrease in maximum elevation. 95% of the permutation importance of the model was contributed to by annual evapotranspiration (87.9%), temperature seasonality (3.9%), minimum precipitation (2.1%) and minimum temperature (1.6%).

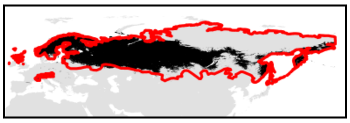

a) ~ 1930s (1900-1949)

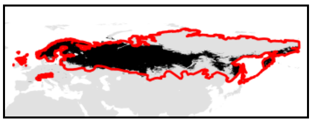

b) ~ 1980s (1950-2000)

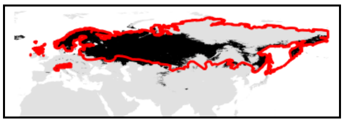

c) ~ 2020s (2010-2039)

Black = predicted suitable envelope

Red polygon = IUCN range (Smith, A.T. & Johnston, C.H. 2008)

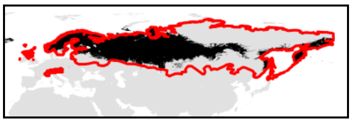

d) ~ 2050s (2040-2069)

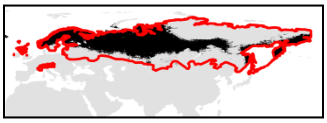

e) ~ 2080s (2070-2099)

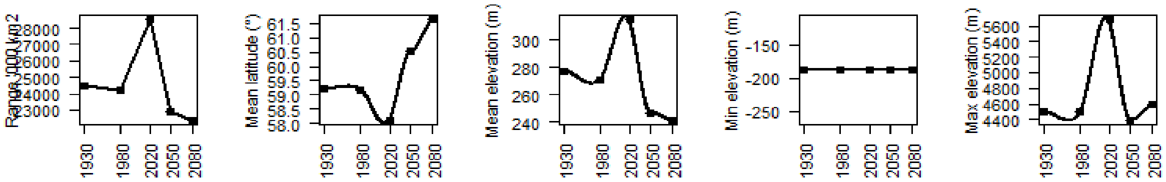

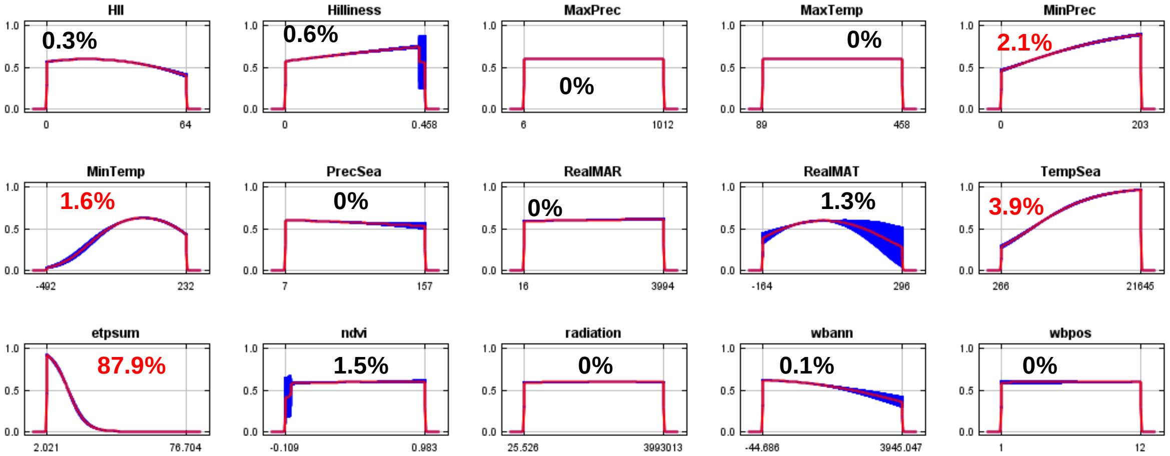

#33 – Irish hare (*Lepus timidus hibernicus*)
n = 706
Expert: Neil Reid, Queen's University Belfast
Expert evaluation: Medium
Data: Only modern
Envelope: Climatic and habitat
Dispersal distance: 2km/year (Expert)
Status: MODELLABLE; **Included in final analysis:** √

Model evaluation metric	
AUC	0.94
Omission rate	0.08
Sensitivity	0.92
Specificity	0.97
Proportion correct	0.96
**Kappa**	**0.75**
True Skill Statistic	0.88

Summary: The Irish hare's bioclimatic envelope is predicted to decrease by 50% with a ~0.5° mean latitudinal polewards shift and a mean decrease in elevation of ~10m driven by a decrease in maximum elevation. 95% of the permutation importance of the model was contributed to by temperature seasonality (44.6%), annual evapotranspiration (41.5%), normalised difference vegetation index (6.4%), precipitation seasonality (3.6%) and maximum precipitation (2.5%).

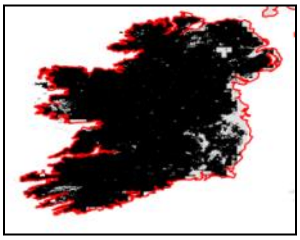

a) ~ 1930s (1900-1949)

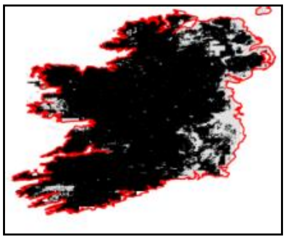

b) ~ 1980s (1950-2000)

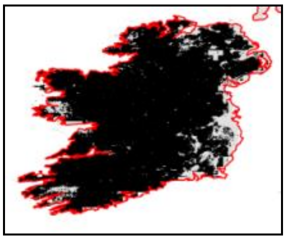

c) ~ 2020s (2010-2039)

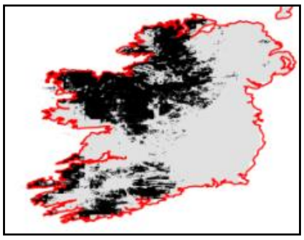

d) ~ 2050s (2040-2069)

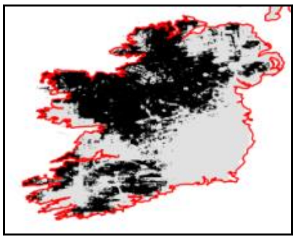

e) ~ 2080s (2070-2099)

Black = predicted suitable envelope

Red polygon = IUCN range (Smith, A.T. & Johnston, C.H. 2008)

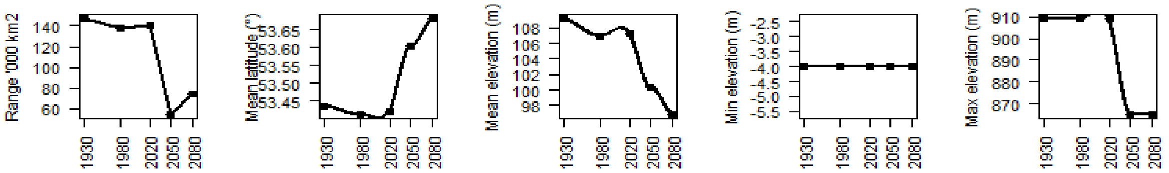

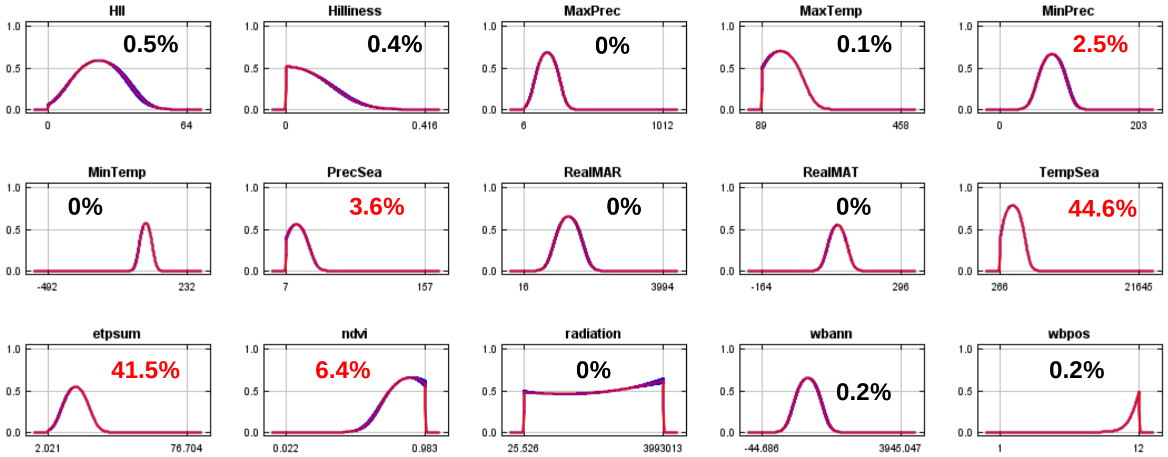

#34 – Mountain hare (*Lepus timidus*)
– Eurasian & Irish populations combined
n = 3,166
Expert: Neil Reid, Queen's University Belfast
Expert evaluation: Medium
Data: Only modern
Envelope: Climatic and habitat
Dispersal distance: 2km/year (Expert)
Status: MODELLABLE; **Included in final analysis:** √

Model evaluation metric	
AUC	0.92
Omission rate	0.07
Sensitivity	0.93
Specificity	0.91
Proportion correct	0.91
**Kappa**	**0.78**
True Skill Statistic	0.84

Summary: The Mountain hare's bioclimatic envelope is predicted to decrease by 10% with a ~2° mean latitudinal polewards shift and a mean decrease in elevation of ~40m driven by a decrease in maximum elevation. 95% of the permutation importance of the model was contributed to by annual evapotranspiration (87.6%), temperature seasonality (4.1%), minimum precipitation (2.1%) and minimum temperature (1.6%).

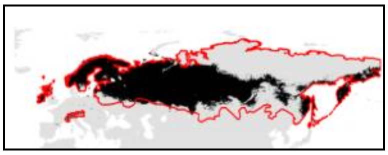
a) ~ 1930s (1900-1949)

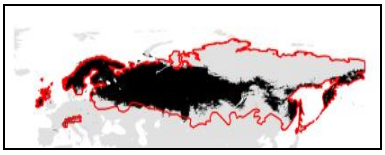
b) ~ 1980s (1950-2000)

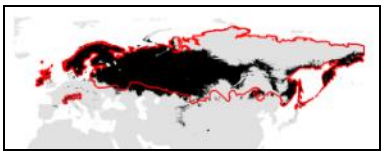
c) ~ 2020s (2010-2039)

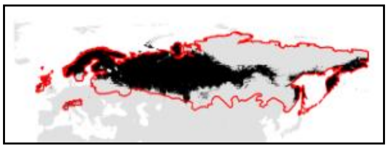
d) ~ 2050s (2040-2069)

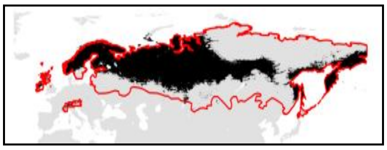
e) ~ 2080s (2070-2099)

Black = predicted suitable envelope

Red polygon = IUCN range (Smith, A.T. & Johnston, C.H. 2008)

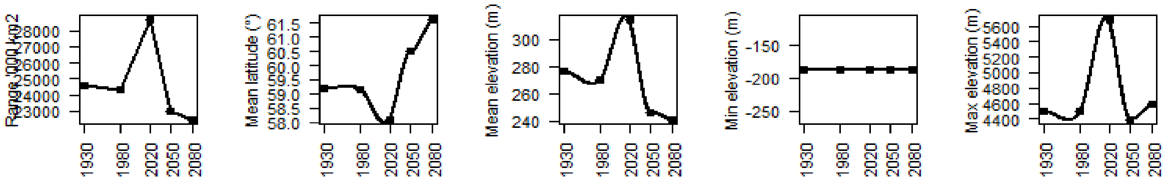

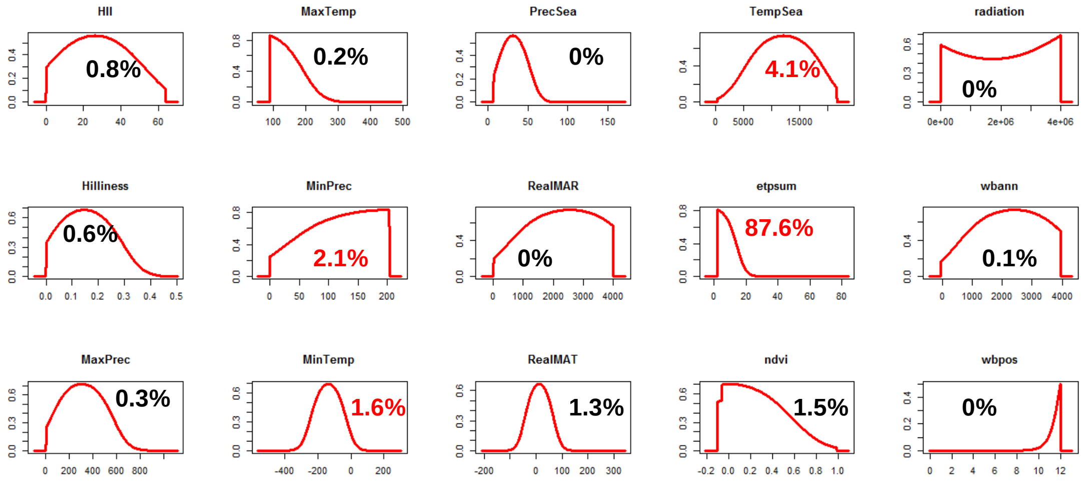

#35 – Tolai hare (*Lepus tolai*)
n = 316
Expert: Chelmala Srinivasulu, Osmania University, India
Expert evaluation: Medium
Data: Only modern
Envelope: Climatic and habitat
Dispersal distance: 2.5km/year (Asian leporids, range 1-35)
Status: MODELLABLE; **Included in final analysis:** √

Model evaluation metric	
AUC	0.94
Omission rate	0.11
Sensitivity	0.89
Specificity	0.99
Proportion correct	0.98
**Kappa**	**0.76**
True Skill Statistic	0.88

Summary: The Tolai hare's bioclimatic envelope is predicted to increase by 70% with a ~3° mean latitudinal polewards shift and a mean increase in elevation of ~280m driven by an increase in maximum elevation. 95% of the permutation importance of the model was contributed to by temperature seasonality (43.5%), maximum temperature (17.3%), mean annual temperature (15.2%), precipitation seasonality (9.6%), annual evapotranspiration (4.6%), mean annual precipitation (3.7%) and minimum precipitation (1.8%).

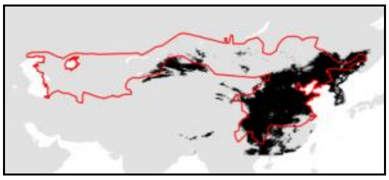
a) ~ 1930s (1900-1949)

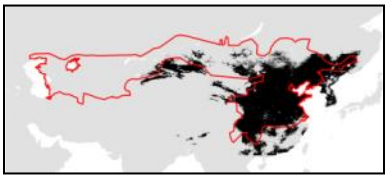
b) ~ 1980s (1950-2000)

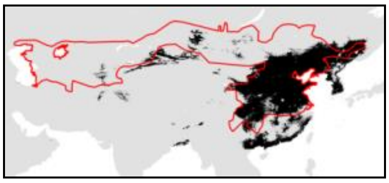
c) ~ 2020s (2010-2039)

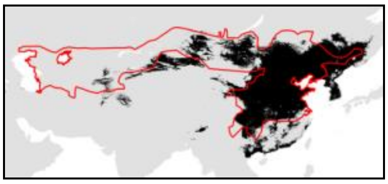
d) ~ 2050s (2040-2069)

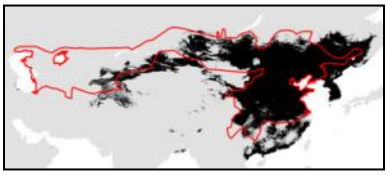
e) ~ 2080s (2070-2099)

Black = predicted suitable envelope

Red polygon = IUCN range (China Red List & Johnston, C.H. 2008)

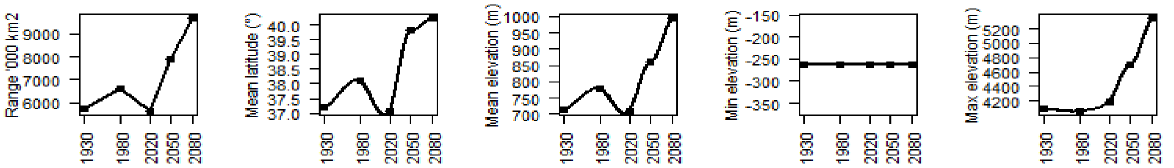

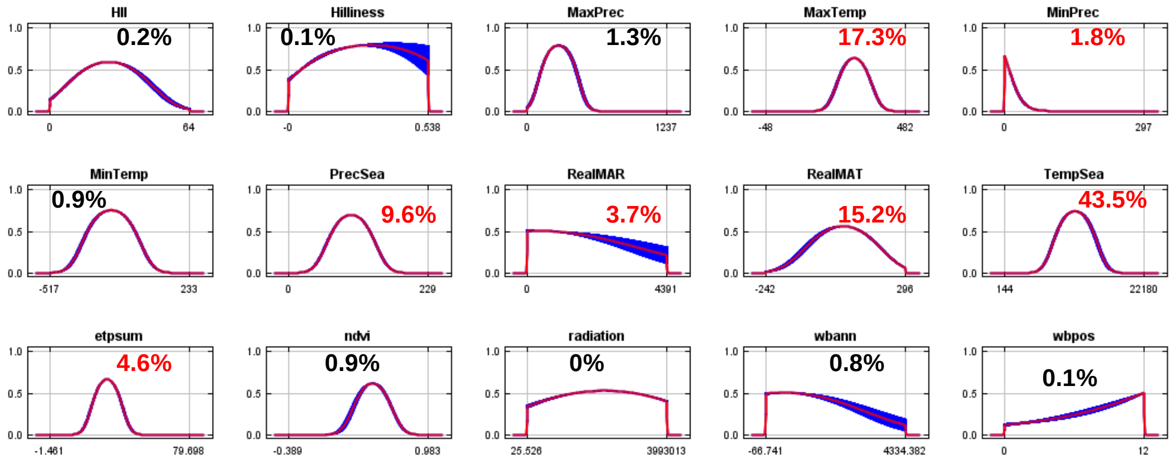

#36 – White-tailed jackrabbit (*Lepus townsendii*)
n = 275
Expert: Eric Waltari, City University of New York
Expert evaluation: Medium
Data: Only modern
Envelope: Climatic and habitat
Dispersal distance: 18.9km/year (N.Am. leporids, range 2-25)
Status: MODELLABLE; **Included in final analysis:** √

Model evaluation metric	
AUC	0.94
Omission rate	0.10
Sensitivity	0.90
Specificity	0.99
Proportion correct	0.99
**Kappa**	**0.76**
True Skill Statistic	0.89

Summary: The White-tailed jackrabbit's bioclimatic envelope is predicted to decrease by 10% with a ~4° mean latitudinal polewards shift and a mean increase in elevation of ~200m driven by an increase in minimum elevation. 95% of the permutation importance of the model was contributed to by mean annual temperature (62.5%), maximum temperature (28.5%), temperature seasonality (3.4%) and annual evapotranspiration (1.6%).

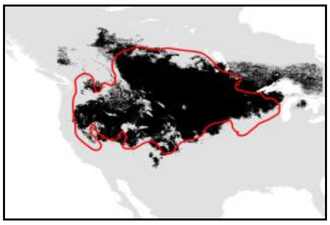
a) ~ 1930s (1900-1949)

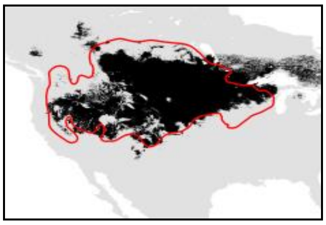
b) ~ 1980s (1950-2000)

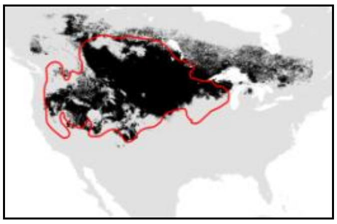
c) ~ 2020s (2010-2039)

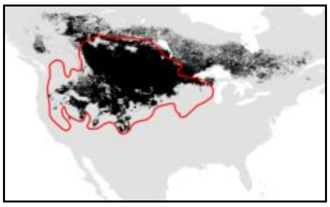
d) ~ 2050s (2040-2069)

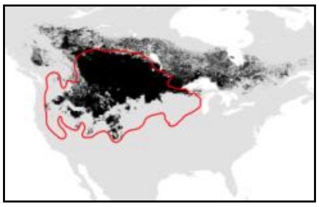
e) ~ 2080s (2070-2099)

Black = predicted suitable envelope

Red polygon = IUCN range (Smith, A.T. & Johnston, C.H. 2008)

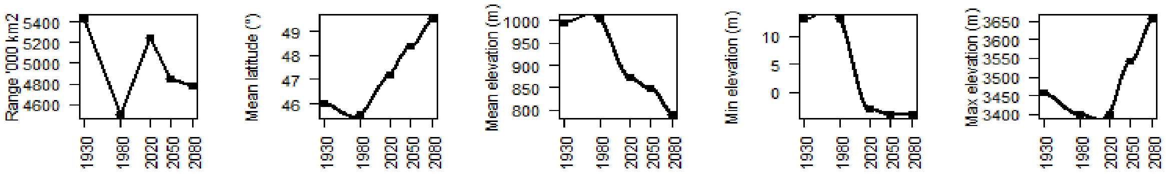

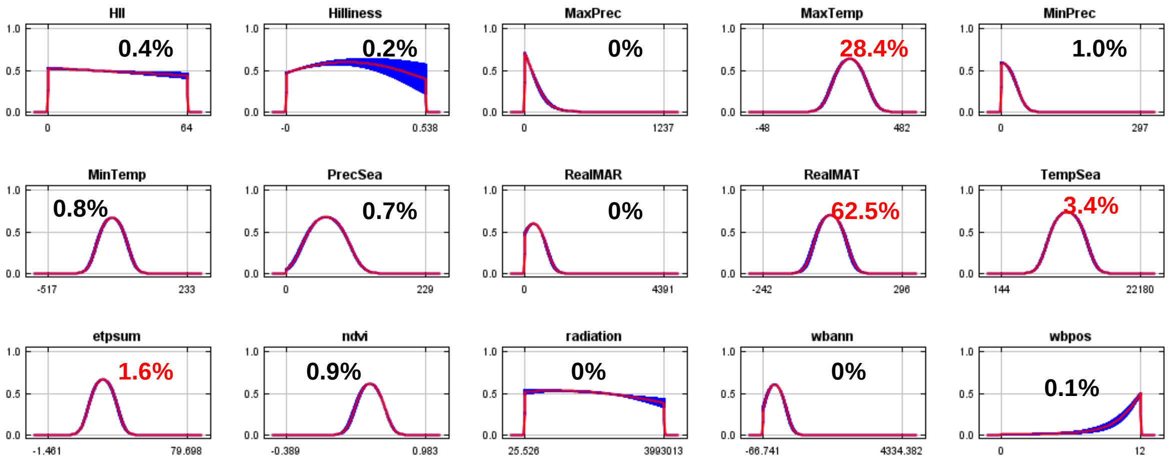

#37 – Yarkand hare (*Lepus yarkandensis*)
n = 49
Expert: Weihe Yang, Institute of Zoology, Chinese Academy of Sciences
Expert evaluation: Medium
Data: Modern and historic
Envelope: Climatic and habitat
Dispersal distance: 2km/year (Smith & Xie, 2008)
Status: MODELLABLE; **Included in final analysis:** √

Model evaluation metric	
AUC	0.95
Omission rate	0.10
Sensitivity	0.90
Specificity	0.99
Proportion correct	0.99
**Kappa**	**0.74**
True Skill Statistic	0.90

Summary: The Yarkand hare's bioclimatic envelope is predicted to decrease by 100% with a ~1° mean latitudinal shift towards the Equator and a mean increase in elevation of ~1000m driven by an increase in minimum elevation. 95% of the permutation importance of the model was contributed to by minimum precipitation (58.8%), maximum precipitation (16.3%), mean annual precipitation (11.9%) and minimum temperature (11.7%).

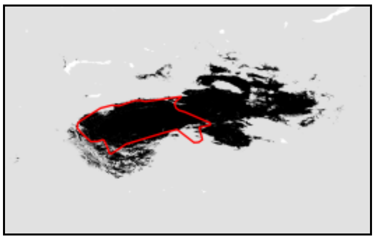
a) ~ 1930s (1900-1949)

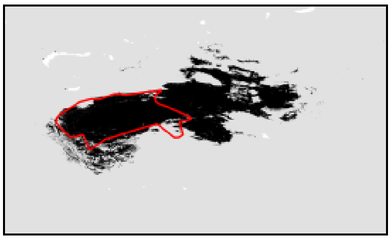
b) ~ 1980s (1950-2000)

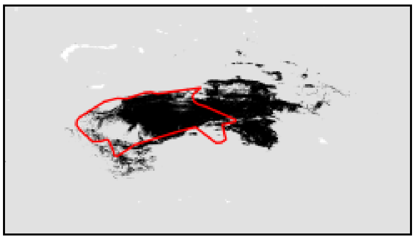
c) ~ 2020s (2010-2039)

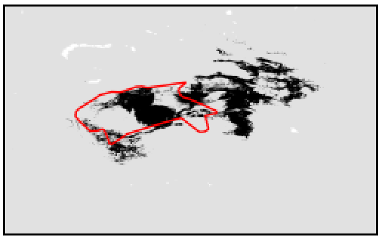
d) ~ 2050s (2040-2069)

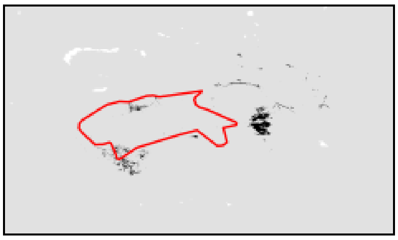
e) ~ 2080s (2070-2099)

Black = predicted suitable envelope

Red polygon = IUCN range (Smith, A.T. & Johnston, C.H. 2008)

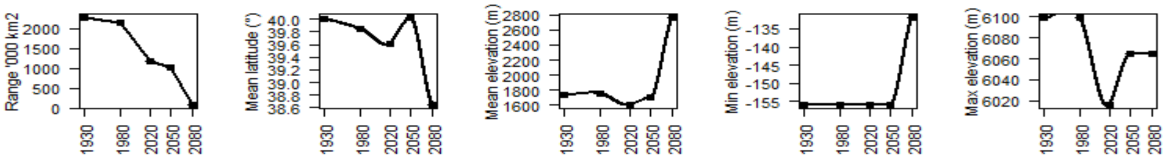

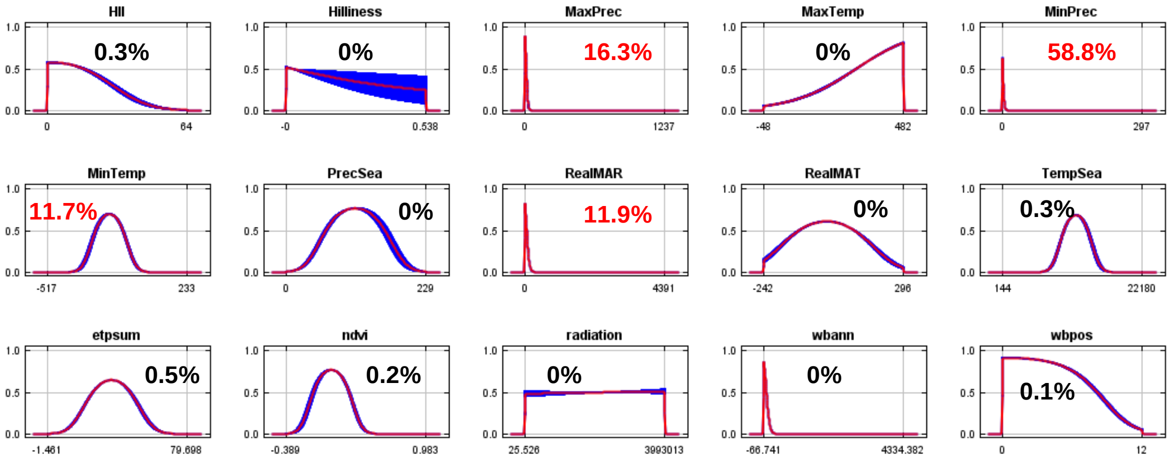

#38 – Sumatran striped rabbit (*Nesolagus netscheri*)
n = 11
Expert: Hariyo Wibisono, Wildlife Conservation Society, Indonesia
Expert evaluation: Poor
Data: Modern and historic
Envelope: Climatic and habitat
Dispersal distance: 0.01km/year (Expert)
Status: UNMODELLABLE; **Included in final analysis:** X

Model evaluation metric	
AUC	0.99
Omission rate	0.00
Sensitivity	1.00
Specificity	0.99
Proportion correct	0.99
**Kappa**	**0.95**
True Skill Statistic	0.99

Summary: The Sumatran striped rabbit's bioclimatic envelope is predicted to decrease by 91% with a ~1° mean latitudinal shift towards the Equator and a mean increase in elevation of ~330m driven by an increase in minimum elevation. 95% of the permutation importance of the model was contributed to by temperature seasonality (99.3%).

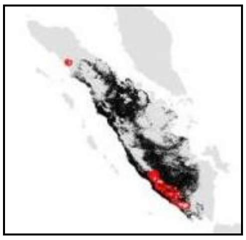

a) ~ 1930s (1900-1949)

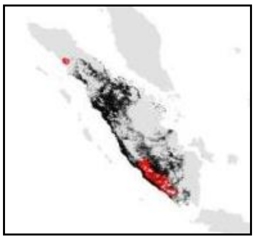

b) ~ 1980s (1950-2000)

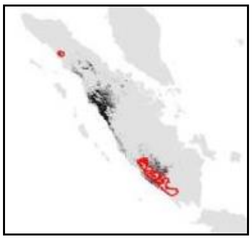

c) ~ 2020s (2010-2039)

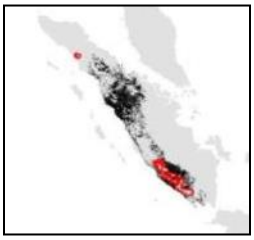

d) ~ 2050s (2040-2069)

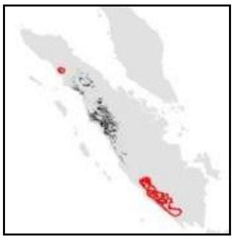

e) ~ 2080s (2070-2099)

Black = predicted suitable envelope

Red polygon = IUCN range (Meijaard, E. & Sugardjito, J. 2008)

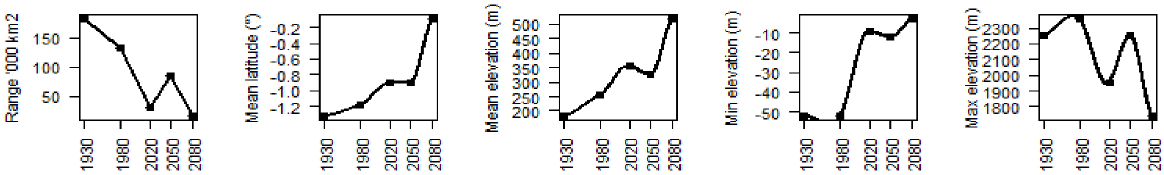

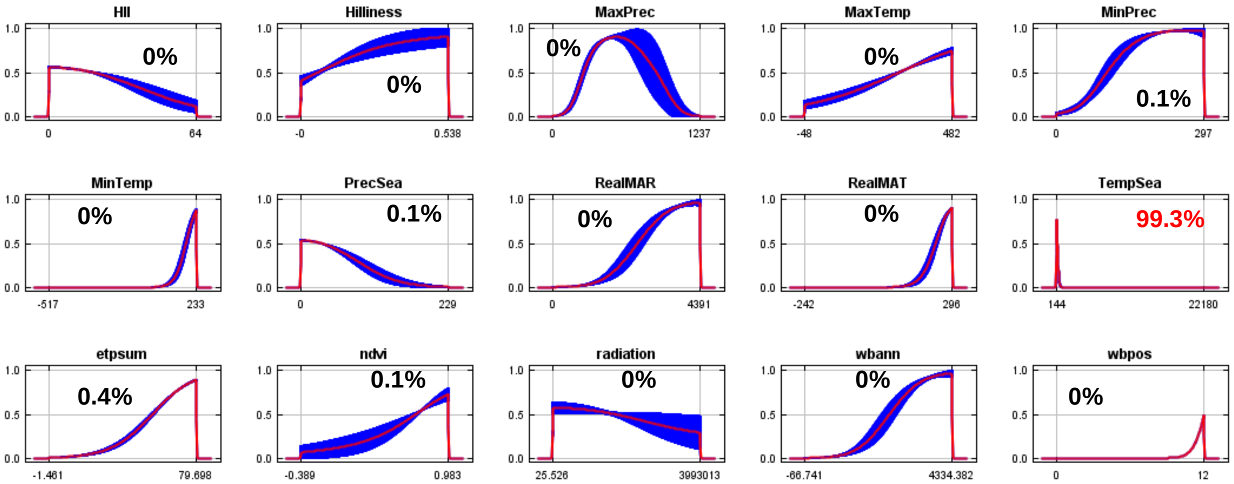

#39 – Annamite striped rabbit (*Nesolagus timminsi*)
n = 4
Expert: Thomas Gray, WWF Greater Mekong & Andrew Tilker, University of Texas Austin
Expert evaluation: Poor
Data: Only modern
Envelope: Climatic and habitat
Dispersal distance: 10km/year (Expert)
Status: UNMODELLABLE; **Included in final analysis:** X

Model evaluation metric	
AUC	0.99
Omission rate	0.00
Sensitivity	1.00
Specificity	0.99
Proportion correct	0.99
**Kappa**	**0.50**
True Skill Statistic	0.99

Summary: The Annamite striped rabbit's bioclimatic envelope is predicted to decrease by 1500% with a ~0.3^{o} mean latitudinal shift towards the Equator and a mean decrease in elevation of ~20m driven by an decrease in minimum elevation. 95% of the permutation importance of the model was contributed to by solar radiation (43.3%), minimum temperature (30.0%), mean annual temperature (14.1%), human influence index (6.4%) and annual evapotranspiration (2.3%).

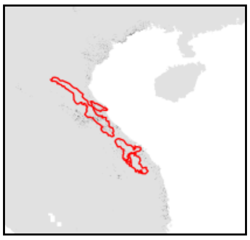

a) ~ 1930s (1900-1949)

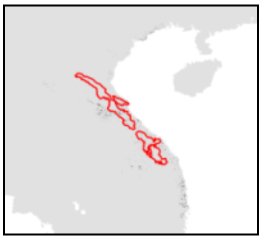

b) ~ 1980s (1950-2000)

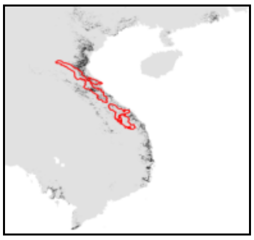

c) ~ 2020s (2010-2039)

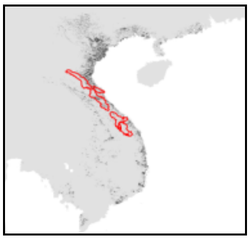

d) ~ 2050s (2040-2069)

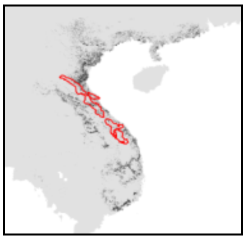

e) ~ 2080s (2070-2099)

Black = predicted suitable envelope

Red polygon = IUCN range (Abramov, A., *et al.* 2008)

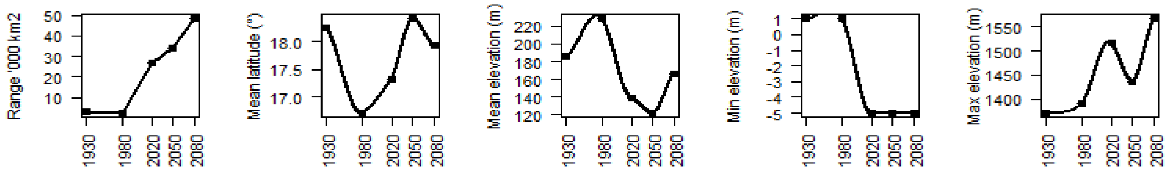

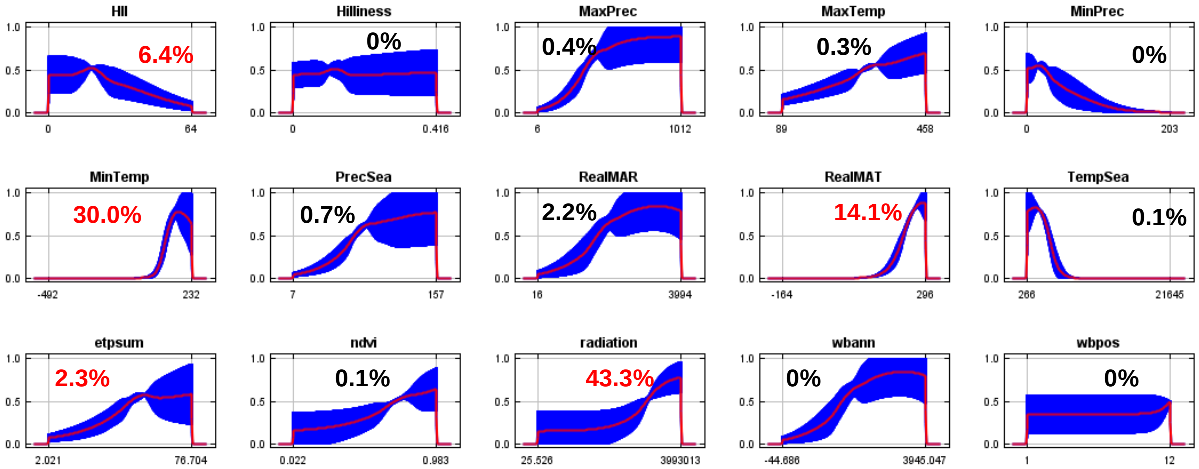

#40 – Alpine pika (*Ochotona alpina*)
n = 16
Expert: Sumiya Ganzorig, Hokkaido University
Expert evaluation: Poor
Data: Modern and historic
Envelope: Climatic and habitat
Dispersal distance: 10km/year (Similar ecology to *O.pallasi*)
Status: UNMODELLABLE; **Included in final analysis:** X

Model evaluation metric	
AUC	0.99
Omission rate	0.00
Sensitivity	1.00
Specificity	0.99
Proportion correct	0.99
**Kappa**	**0.29**
True Skill Statistic	0.99

Summary: The Alpine pika's bioclimatic envelope is predicted to decrease by 10% with a ~4° mean latitudinal polewards shift and a mean decrease in elevation of ~80m. 95% of the permutation importance of the model was contributed to by minimum precipitation (50.1%), mean annual temperature (31.1%), number of months with a positive water balance (10.9%), minimum temperature (2.3%) and annual evapotranspiration (1.3%).

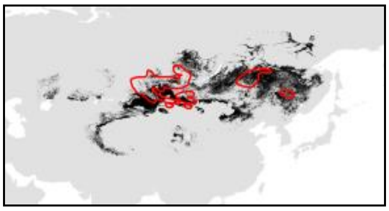

a) ~ 1930s (1900-1949)

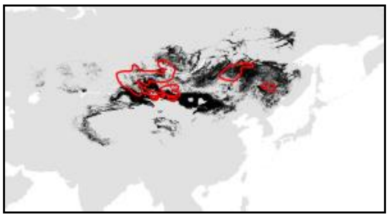

b) ~ 1980s (1950-2000)

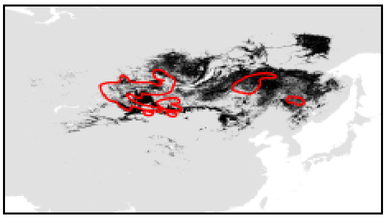

c) ~ 2020s (2010-2039)

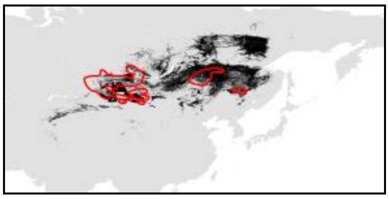

d) ~ 2050s (2040-2069)

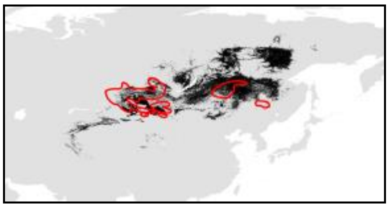

e) ~ 2080s (2070-2099)

Black = predicted suitable envelope

Red polygon = IUCN range (Smith, A.T. & Johnston, C.H. 2008)

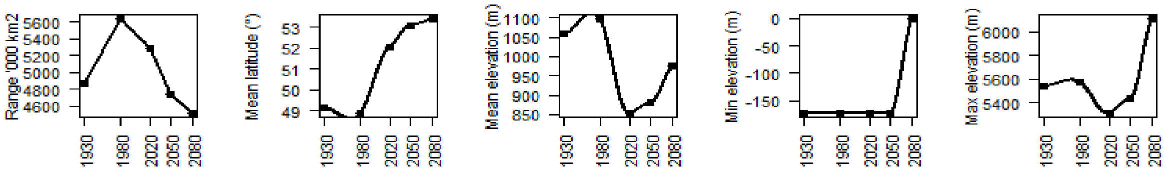

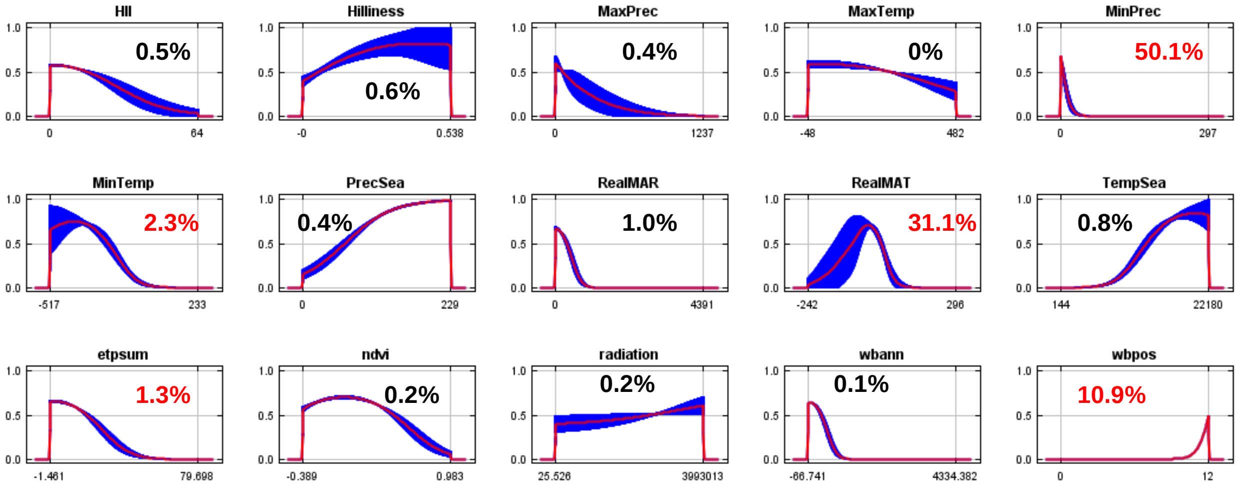

#41 – Silver pika (*Ochotona argentata*)
n = 4
Expert: Andrew Smith, Arizona State University
Expert evaluation: Poor
Data: Only modern
Envelope: Climatic and habitat
Dispersal distance: 3km/year (Asian pikas, range 1-15)
Status: UNMODELLABLE; **Included in final analysis:** X

Model evaluation metric	
AUC	1.00
Omission rate	0.00
Sensitivity	1.00
Specificity	1.00
Proportion correct	1.00
**Kappa**	**1.00**
True Skill Statistic	1.00

Summary: The Silver pika's bioclimatic envelope is predicted to decrease by 80% with a ~4° mean latitudinal polewards shift and a mean increase in elevation of ~360m driven by an increase in minimum elevation. 95% of the permutation importance of the model was contributed to by minimum precipitation (62.7%), human influence index (17.1%), mean annual precipitation (8.6%), minimum temperature (5.4%) and annual evapotranspiration (1.8%).

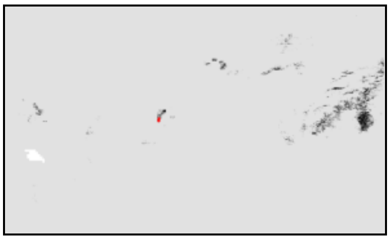

a) ~ 1930s (1900-1949)

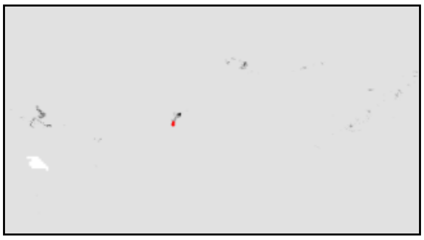

b) ~ 1980s (1950-2000)

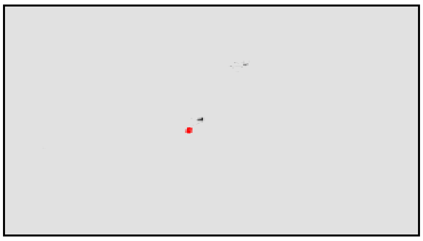

c) ~ 2020s (2010-2039)

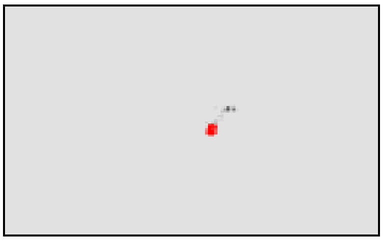

d) ~ 2050s (2040-2069)

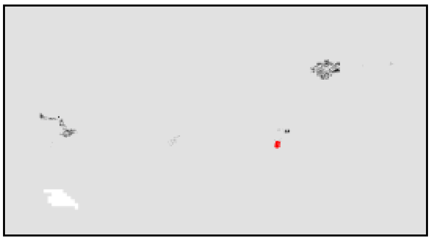

e) ~ 2080s (2070-2099)

Black = predicted suitable envelope

Red polygon = IUCN range (Smith, A.T. & Johnston, C.H. 2008)

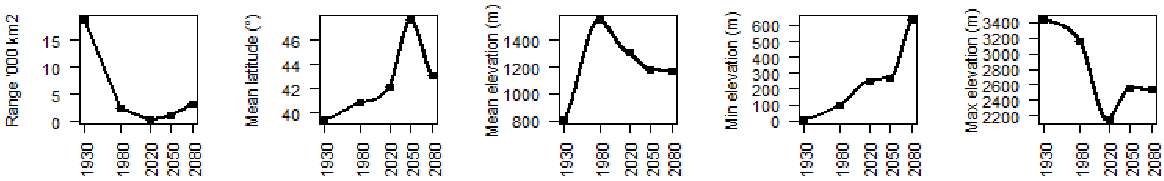

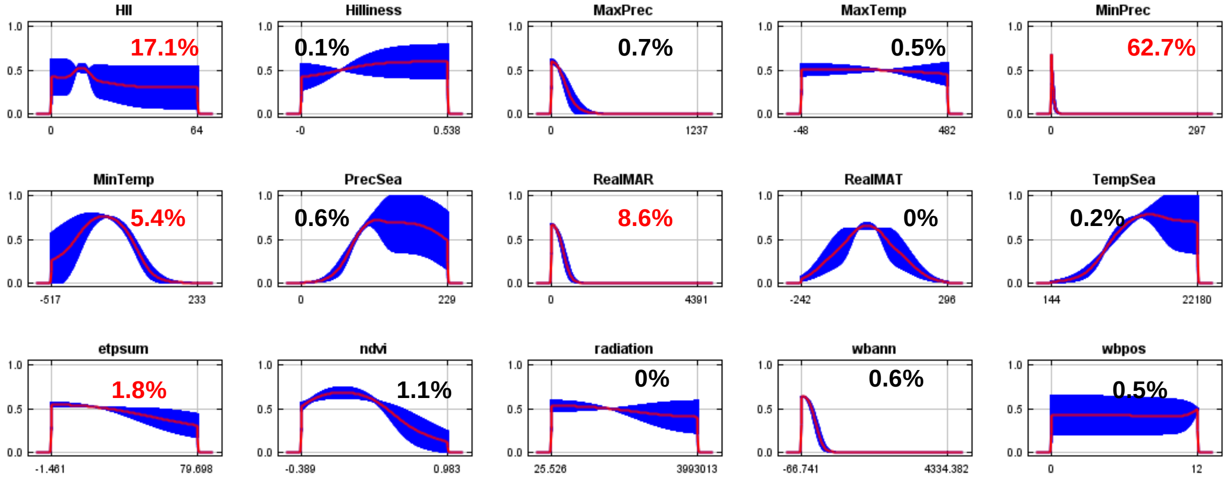

#42 – Gansu pika (*Ochotona cansus*)
n = 38
Expert: Andrew Smith, Arizona State University
Expert evaluation: Medium
Data: Modern and historic
Envelope: Climatic and habitat
Dispersal distance: 1.5km/year (Similar ecology to *O.roylei*)
Status: MODELLABLE; **Included in final analysis:** √

Model evaluation metric	
AUC	0.95
Omission rate	0.11
Sensitivity	0.89
Specificity	0.99
Proportion correct	0.99
**Kappa**	**0.61**
True Skill Statistic	0.89

Summary: The Gansu pika's bioclimatic envelope is predicted to decrease by 60% with a ~0.4^o mean latitudinal shift towards the Equator and a mean increase in elevation of ~230m driven by an increase in minimum elevation. 95% of the permutation importance of the model was contributed to by minimum precipitation (89.8%) and minimum temperature (6.9%).

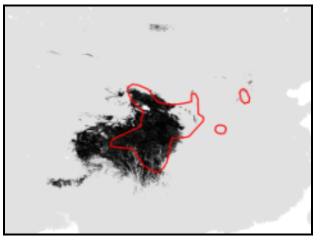

a) ~ 1930s (1900-1949)

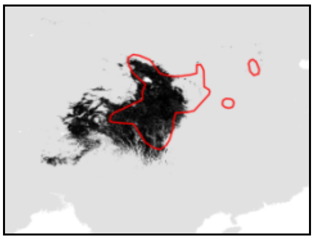

b) ~ 1980s (1950-2000)

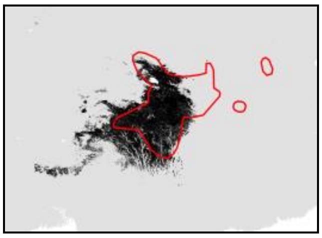

c) ~ 2020s (2010-2039)

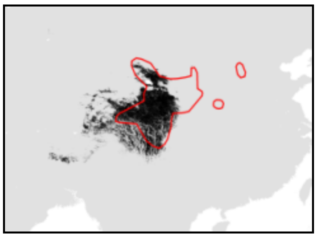

d) ~ 2050s (2040-2069)

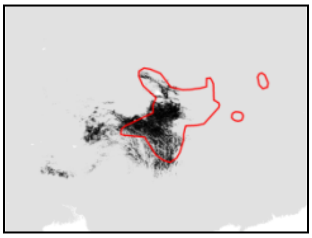

e) ~ 2080s (2070-2099)

Black = predicted suitable envelope

Red polygon = IUCN range (Smith, A.T. & Johnston, C.H. 2008)

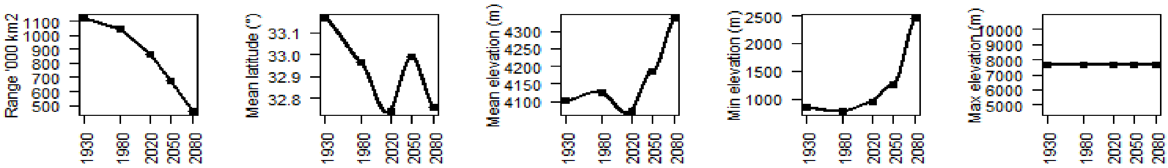

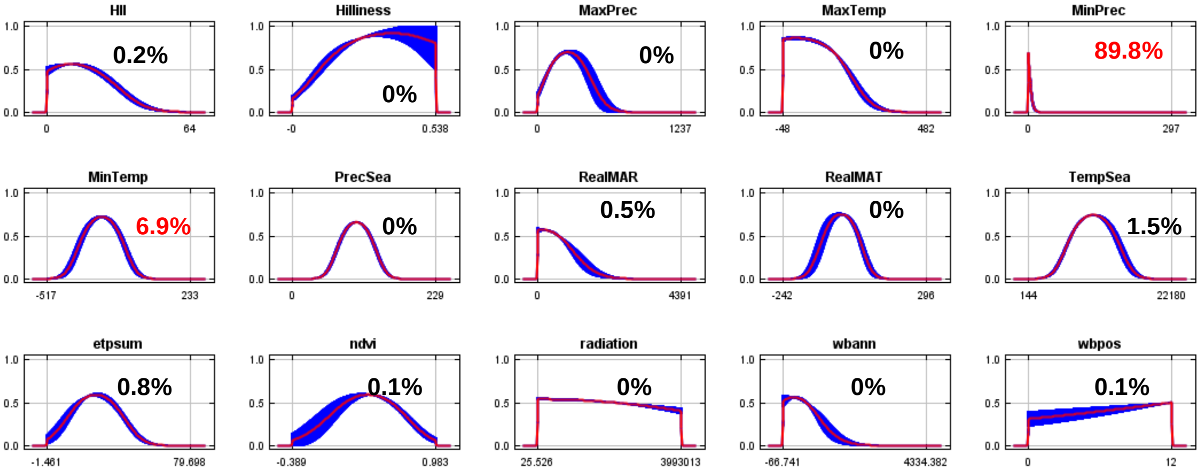

Model evaluation metric	
AUC	0.95
Omission rate	0.10
Sensitivity	0.90
Specificity	0.99
Proportion correct	0.99
**Kappa**	**0.86**
True Skill Statistic	0.90

#43 – Collared pika (*Ochotona collaris*)
n = 193
Expert: Hayley Lanier, University of Michigan & David Hik, University of Alberta
Expert evaluation: Poor
Data: Modern and historic
Envelope: Climatic and habitat
Dispersal distance: 1km/year (Expert)
Status: UNMODELLABLE; **Included in final analysis:** X

Summary: The Collared pika's bioclimatic envelope is predicted to increase by 20% with a ~2° mean latitudinal polewards shift and a mean decrease in elevation of ~140m. 95% of the permutation importance of the model was contributed to by annual evapotranspiration (86.7%), mean annual temperature (3.3%), normalised difference vegetation index (3.2%) and maximum temperature (2.0%).

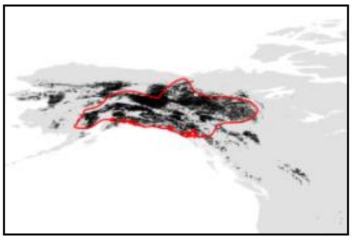

a) ~ 1930s (1900-1949)

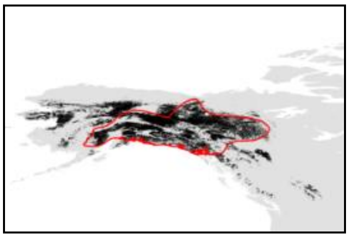

b) ~ 1980s (1950-2000)

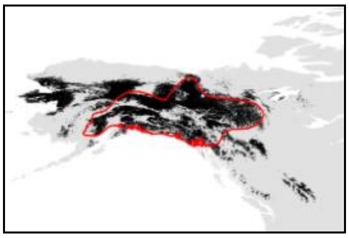

c) ~ 2020s (2010-2039)

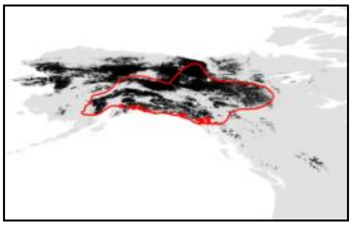

d) ~ 2050s (2040-2069)

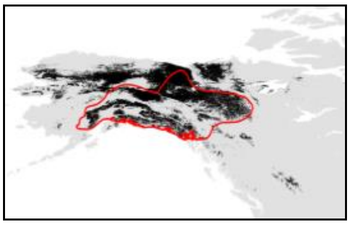

e) ~ 2080s (2070-2099)

Black = predicted suitable envelope

Red polygon = IUCN range (Smith, A.T. & Johnston, C.H. 2008)

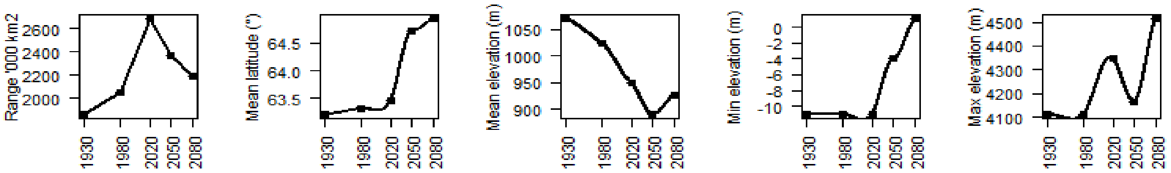

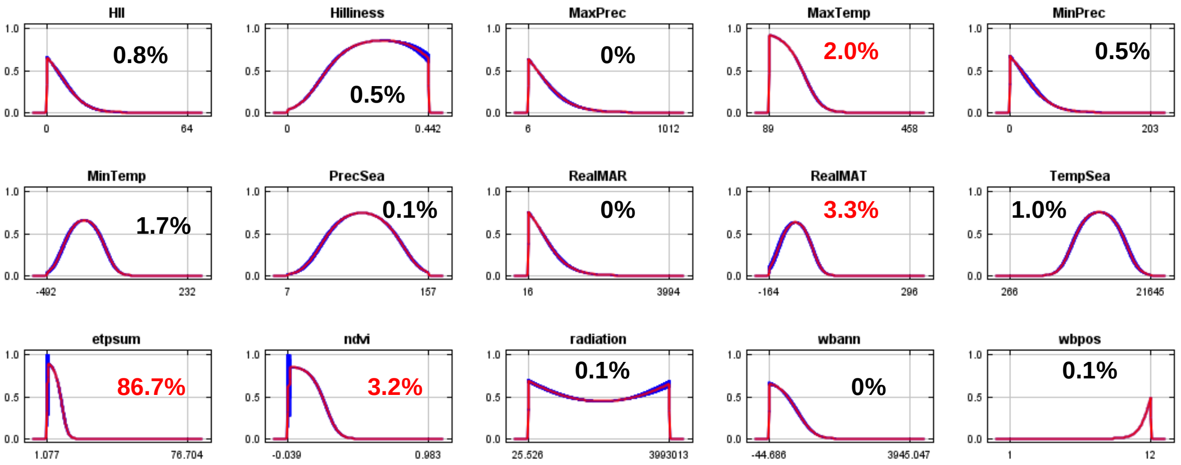

#44 – Plateau pika (*Ochotona curzoniae*)
n = 131
Expert: Andrew Smith, Arizona State University
Expert evaluation: Good
Data: Only modern
Envelope: Climatic and habitat
Dispersal distance: 0.1km/year (Expert)
Status: MODELLABLE; **Included in final analysis:** √

Model evaluation metric	
AUC	0.94
Omission rate	0.11
Sensitivity	0.89
Specificity	0.99
Proportion correct	0.99
**Kappa**	**0.76**
True Skill Statistic	0.88

Summary: The Plateau pika's bioclimatic envelope is predicted to decrease by 30% with a ~1° mean latitudinal shift towards the Equator and a mean increase in elevation of ~700m driven by an increase in minimum and maximum elevation. 95% of the permutation importance of the model was contributed to by minimum precipitation (48.0%), maximum temperature (42.0%) and annual evapotranspiration (6.8%).

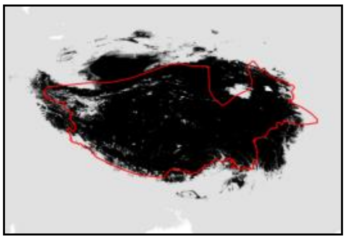
a) ~ 1930s (1900-1949)

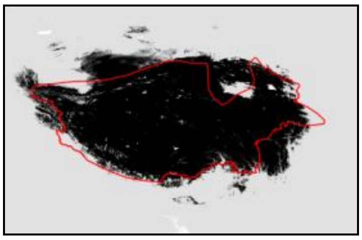
b) ~ 1980s (1950-2000)

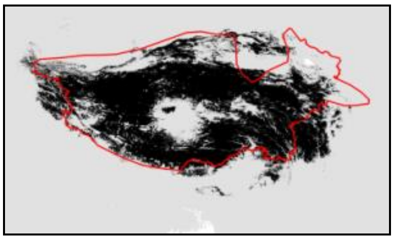
c) ~ 2020s (2010-2039)

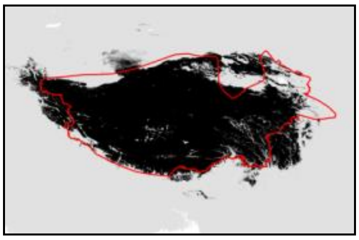
d) ~ 2050s (2040-2069)

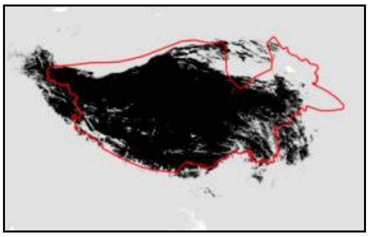
e) ~ 2080s (2070-2099)

Black = predicted suitable envelope

Red polygon = IUCN range (Smith, A.T. & Johnston, C.H. 2008)

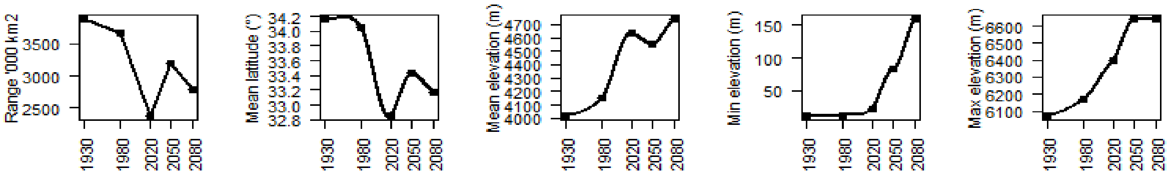

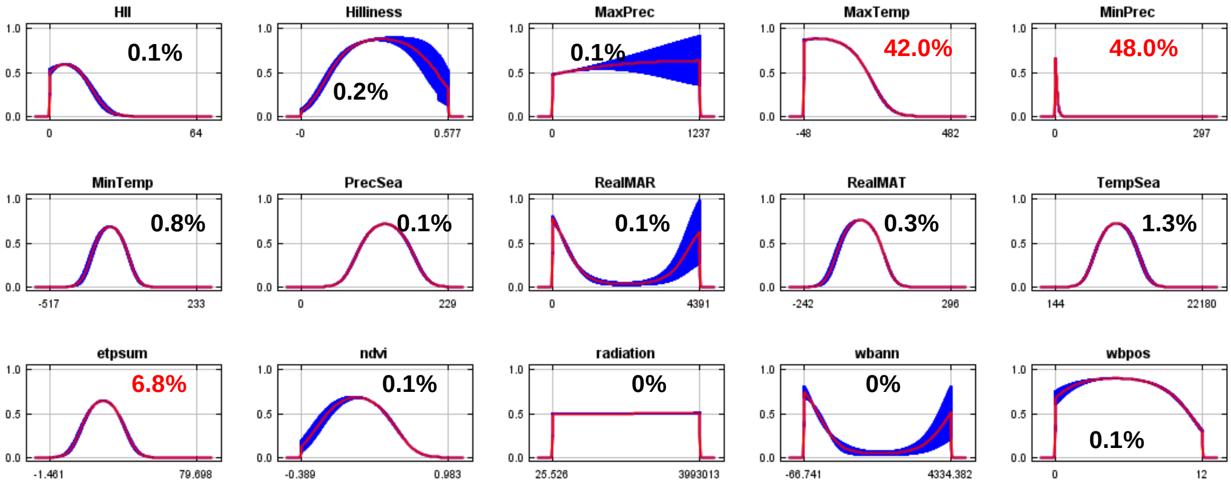

#45 – Daurian pika (*Ochotona dauurica*)
n = 131
Expert: Andrew Smith, Arizona State University
Expert evaluation: Medium
Data: Only modern
Envelope: Climatic and habitat
Dispersal distance: 0.1km/year (Similar ecology to *O.curzoniae*)
Status: MODELLABLE; **Included in final analysis:** √

Model evaluation metric	
AUC	0.95
Omission rate	0.10
Sensitivity	0.90
Specificity	0.99
Proportion correct	0.99
**Kappa**	**0.66**
True Skill Statistic	0.89

Summary: The Daurian pika's bioclimatic envelope is predicted to decrease by 25% with a ~1° mean latitudinal polewards shift and a mean decrease in elevation of ~60m. 95% of the permutation importance of the model was contributed to by minimum precipitation (92.3%) and number of months with a positive water balance (2.7%).

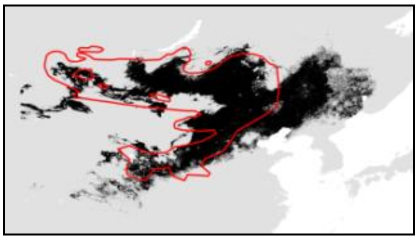
a) ~ 1930s (1900-1949)

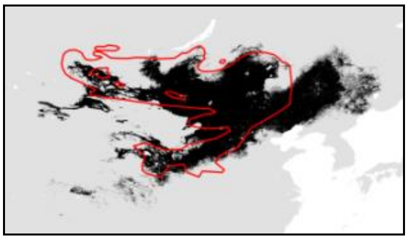
b) ~ 1980s (1950-2000)

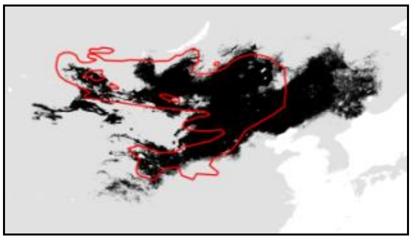
c) ~ 2020s (2010-2039)

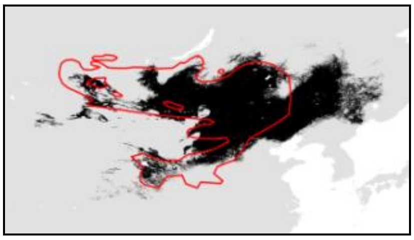
d) ~ 2050s (2040-2069)

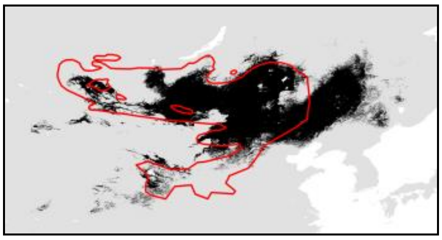
e) ~ 2080s (2070-2099)

Black = predicted suitable envelope

Red polygon = IUCN range (Smith, A.T. & Johnston, C.H. 2008)

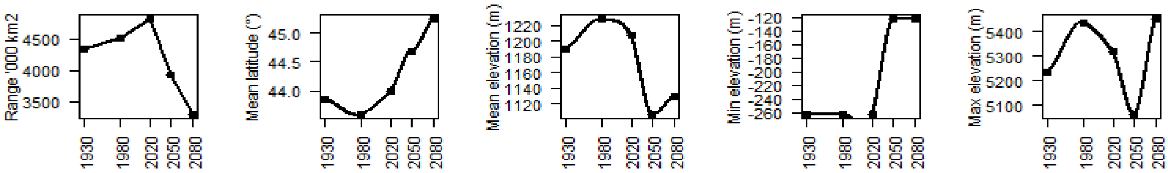

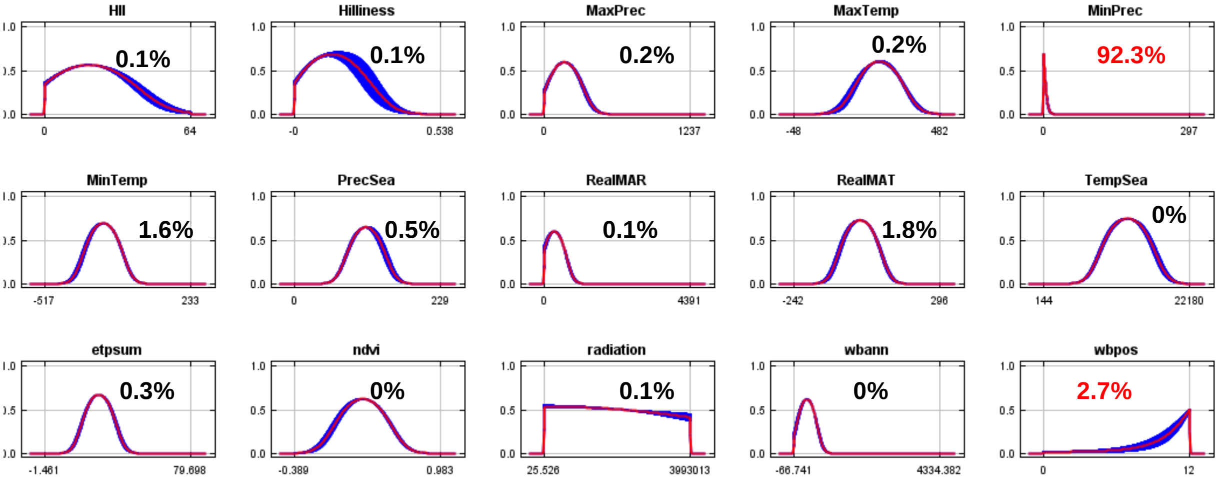

#46 – Chinese red pika (*Ochotona erythrotis*)
n = 39
Expert: Andrew Smith, Arizona State University
Expert evaluation: Poor
Data: Modern and historic
Envelope: Climatic and habitat
Dispersal distance: 3km/year (Asian pikas, range 1-15)
Status: UNMODELLABLE; **Included in final analysis:** X

Model evaluation metric	
AUC	0.95
Omission rate	0.10
Sensitivity	0.90
Specificity	0.99
Proportion correct	0.99
**Kappa**	**0.53**
True Skill Statistic	0.89

Summary: The Chinese red pika's bioclimatic envelope is predicted to decrease by 20% with a ~3° mean latitudinal polewards shift and a mean decrease in elevation of ~2400m driven by a decrease in minimum elevation. 95% of the permutation importance of the model was contributed to by minimum precipitation (87.2%), minimum temperature (7.2%) and mean annual temperature (2.9%).

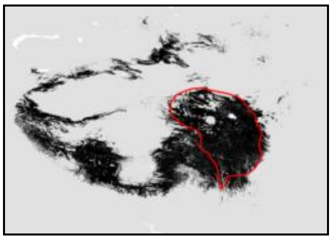
a) ~ 1930s (1900-1949)

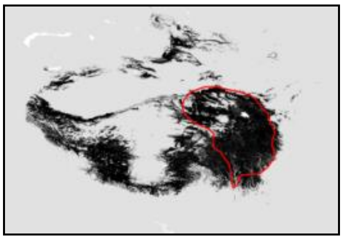
b) ~ 1980s (1950-2000)

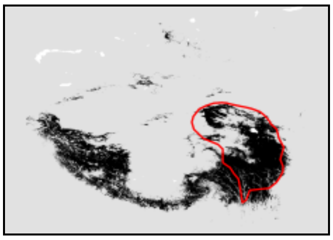
c) ~ 2020s (2010-2039)

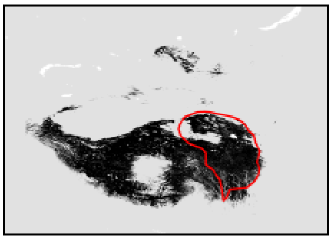
d) ~ 2050s (2040-2069)

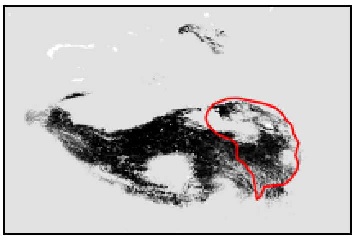
e) ~ 2080s (2070-2099)

Black = predicted suitable envelope

Red polygon = IUCN range (Smith, A.T. & Johnston, C.H. 2008)

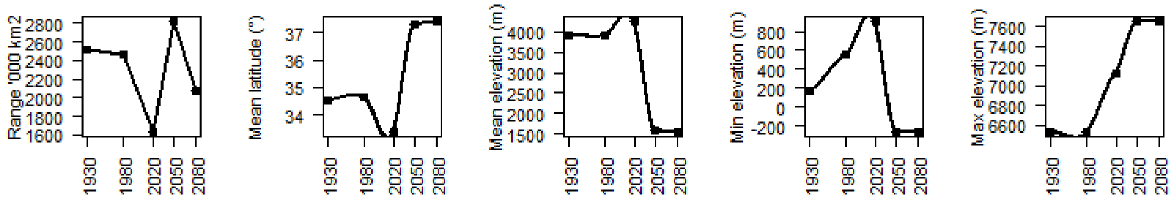

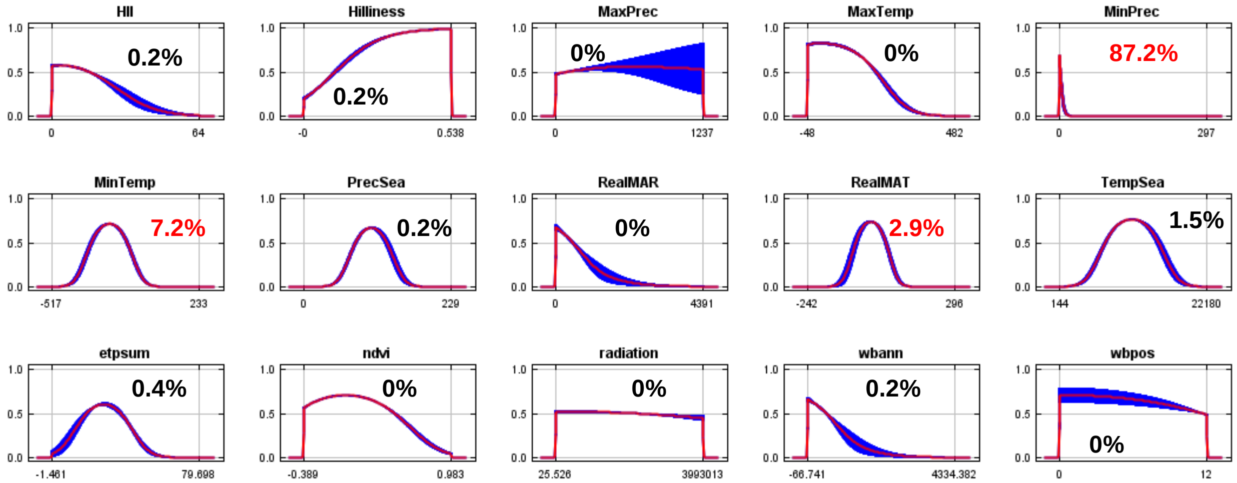

#47 – Forrest's pika (*Ochotona forresti*)
n = 9
Expert: Andrew Smith, Arizona State University
Expert evaluation: Poor
Data: Only modern
Envelope: Climatic and habitat
Dispersal distance: 3km/year (Asian pikas, range 1-15)
Status: UNMODELLABLE; **Included in final analysis:** X

Model evaluation metric	
AUC	0.95
Omission rate	0.10
Sensitivity	0.90
Specificity	0.99
Proportion correct	0.99
**Kappa**	**0.53**
True Skill Statistic	0.89

Summary: The Forrest's pika's bioclimatic envelope is predicted to decrease by 40% with a ~1° mean latitudinal polewards shift and a mean increase in elevation of ~600m driven by an increase in maximum elevation. 95% of the permutation importance of the model was contributed to by minimum precipitation (36.8%), temperature seasonality (20.6%), normalised difference vegetation index (12.1%), maximum temperature (9.3%), precipitation seasonality (7.6%), human influence index (6.2%) and surface roughness index (4.2%).

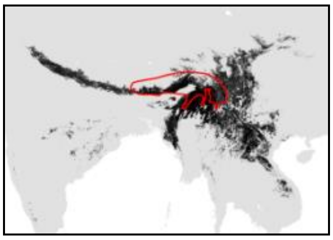

a) ~ 1930s (1900-1949)

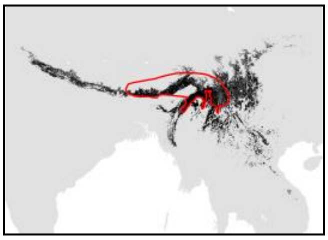

b) ~ 1980s (1950-2000)

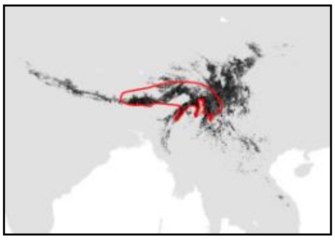

c) ~ 2020s (2010-2039)

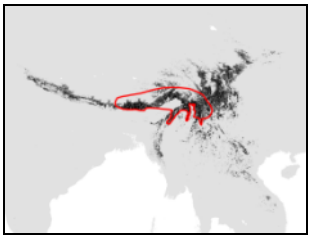

d) ~ 2050s (2040-2069)

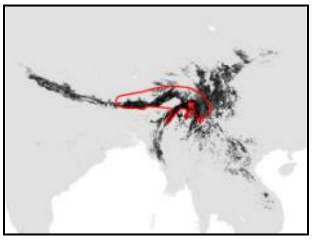

e) ~ 2080s (2070-2099)

Black = predicted suitable envelope

Red polygon = IUCN range (Smith, A.T. & Johnston, C.H. 2008)

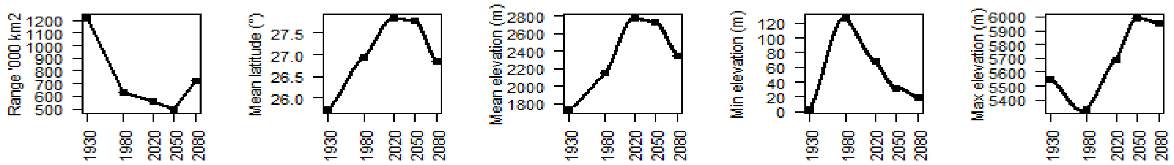

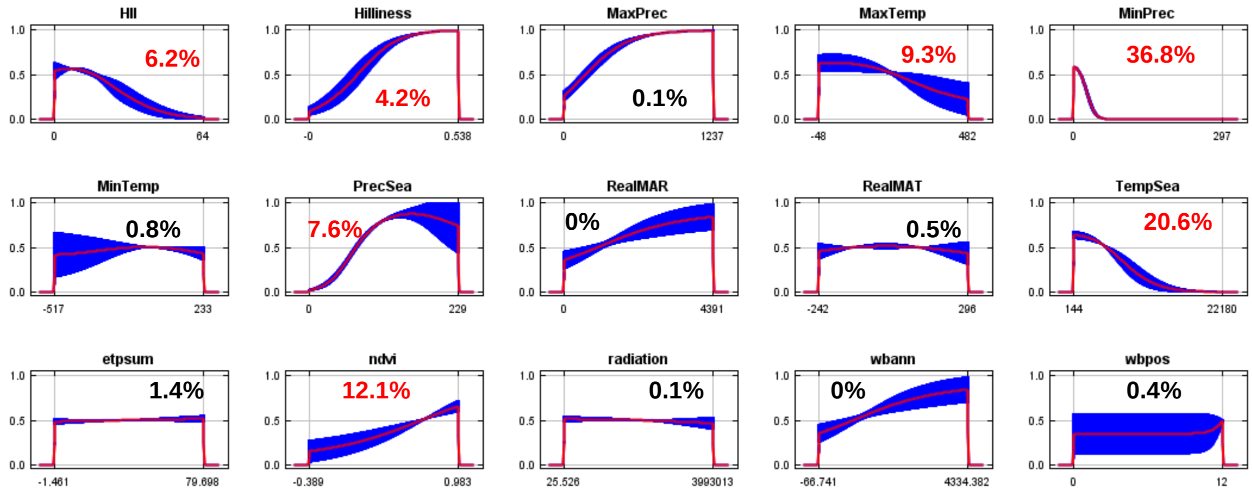

#48 – Glover's pika (*Ochotona gloveri*)
n = 22
Expert: Andrew Smith, Arizona State University
Expert evaluation: Medium
Data: Only modern
Envelope: Climatic and habitat
Dispersal distance: 3km/year (Asian pikas, range 1-15)
Status: MODELLABLE; **Included in final analysis:** √

Model evaluation metric	
AUC	0.99
Omission rate	0.00
Sensitivity	1.00
Specificity	0.99
Proportion correct	0.99
**Kappa**	**0.65**
True Skill Statistic	0.99

Summary: The Glover's pika's bioclimatic envelope is predicted to decrease by 50% with a ~0.5° mean latitudinal polewards shift and a mean increase in elevation of ~270m driven by an increase in minimum and maximum elevation. 95% of the permutation importance of the model was contributed to by minimum precipitation (46.3%), minimum temperature (28.8%), mean annual temperature (12.7%), human influence index (3.8%), temperature seasonality (3.1%) and precipitation seasonality (1.8%).

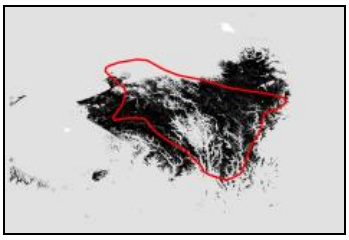
a) ~ 1930s (1900-1949)

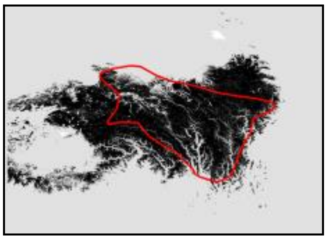
b) ~ 1980s (1950-2000)

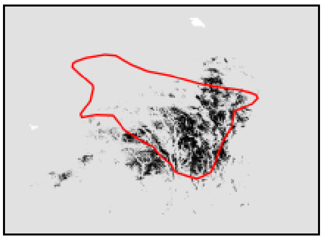
c) ~ 2020s (2010-2039)

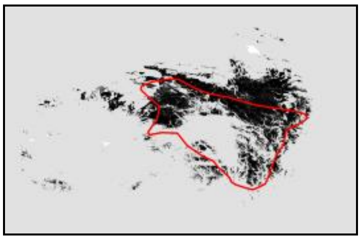
d) ~ 2050s (2040-2069)

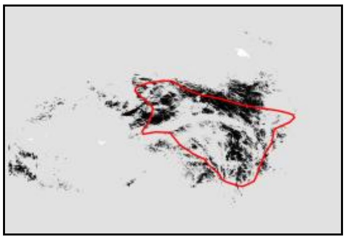
e) ~ 2080s (2070-2099)

Black = predicted suitable envelope

Red polygon = IUCN range (Smith, A.T. & Johnston, C.H. 2008)

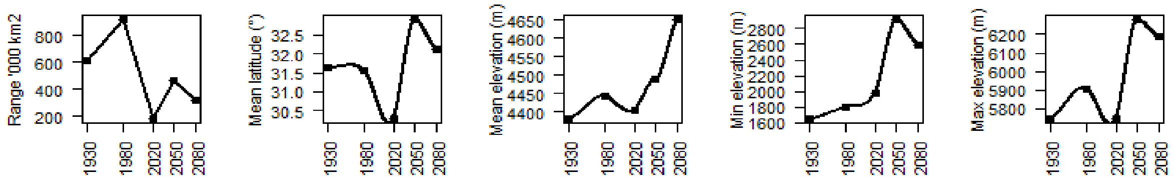

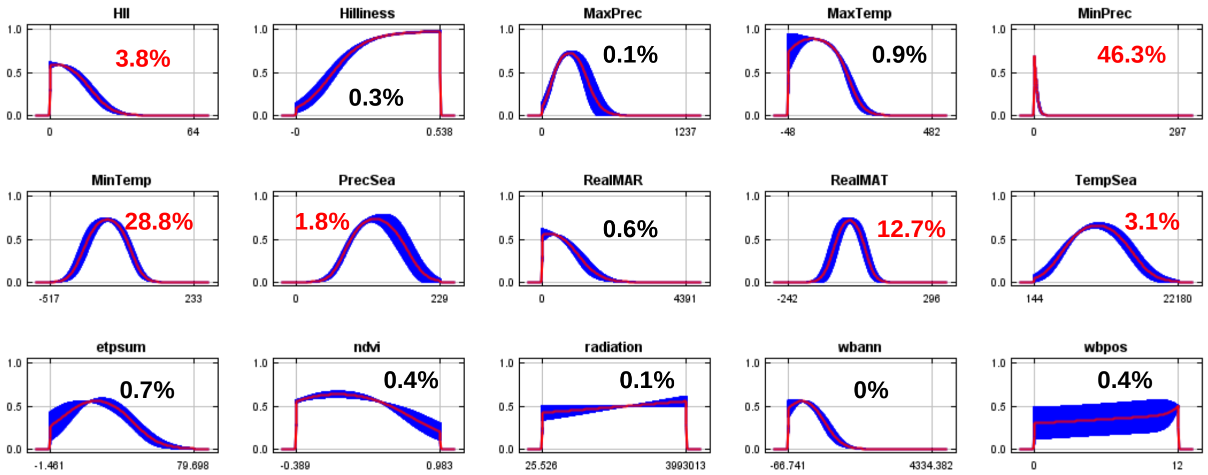

#49 – Hoffmann's pika (*Ochotona hoffmanni*)
n = 5
Expert: Andrey Lissovsky, Zoological Museum of Moscow State University
Expert evaluation: Medium
Data: Modern and historic
Envelope: Climatic and habitat
Dispersal distance: 3km/year (Asian pikas, range 1-15)
Status: MODELLABLE; **Included in final analysis:** √

Model evaluation metric	
AUC	0.99
Omission rate	0.00
Sensitivity	1.00
Specificity	0.99
Proportion correct	0.99
**Kappa**	**0.91**
True Skill Statistic	0.99

Summary: The Hoffmann's pika's bioclimatic envelope is predicted to decrease by 90% with a ~1° mean latitudinal shift towards the Equator and a mean increase in elevation of ~470m driven by an increase in minimum elevation. 95% of the permutation importance of the model was contributed to by precipitation seasonality (73.2%), mean annual temperature (12.6%), minimum temperature (6.5%) and minimum precipitation (5.3%).

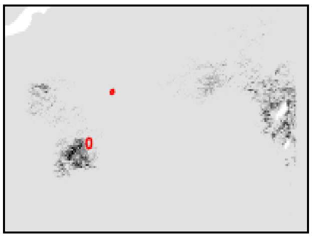
a) ~ 1930s (1900-1949)

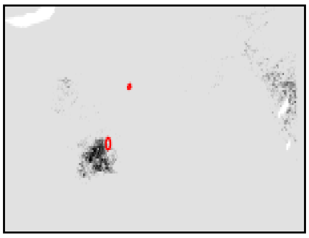
b) ~ 1980s (1950-2000)

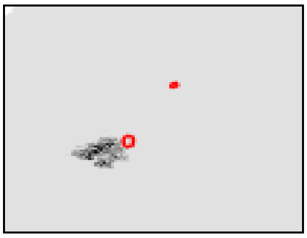
c) ~ 2020s (2010-2039)

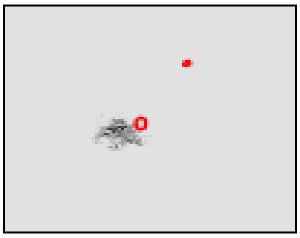
d) ~ 2050s (2040-2069)

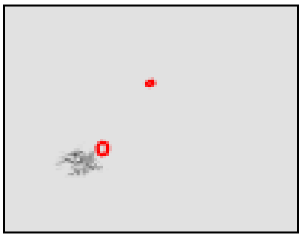
e) ~ 2080s (2070-2099)

Black = predicted suitable envelope

Red polygon = IUCN range (Smith, A.T. & Johnston, C.H. 2008)

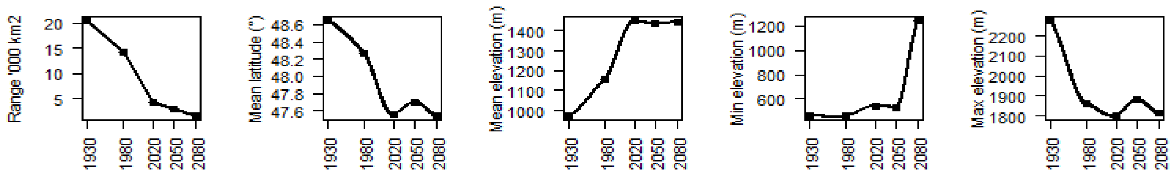

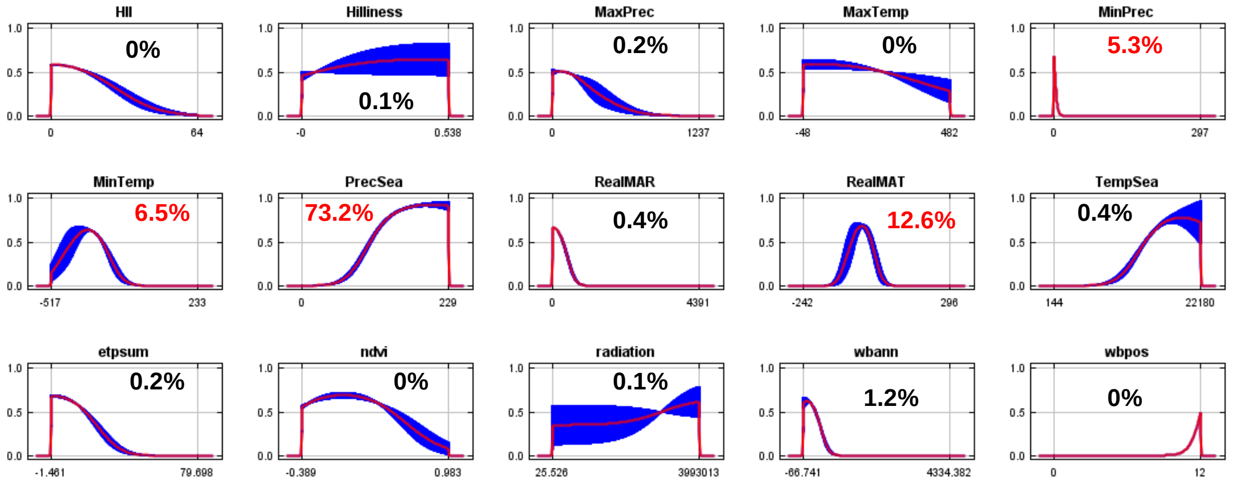

#50 – **Siberian pika** (*Ochotona hyperborea*)
n = 16
Expert: Julia Witczuk, Warsaw Agricultural University, Poland
Expert evaluation: Poor
Data: Modern and historic
Envelope: Climatic and habitat
Dispersal distance: 10km/year (Similar ecology to *O.alpina*)
Status: UNMODELLABLE; **Included in final analysis:** X

Model evaluation metric	
AUC	0.97
Omission rate	0.06
Sensitivity	0.94
Specificity	0.99
Proportion correct	0.99
**Kappa**	**0.40**
True Skill Statistic	0.93

Summary: The Siberian pika's bioclimatic envelope is predicted to decrease by 100% with a ~5° mean latitudinal polewards shift and a mean increase in elevation of ~200m driven by an increase in minimum and maximum elevation. 95% of the permutation importance of the model was contributed to by mean annual temperature (72.5%), annual evapotranspiration (10.4%), minimum precipitation (6.4%), human influence index (2.8%), normalised difference vegetation index (2.6%) and annual water balance (1.8%).

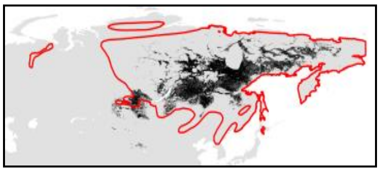
a) ~ 1930s (1900-1949)

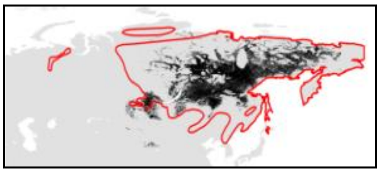
b) ~ 1980s (1950-2000)

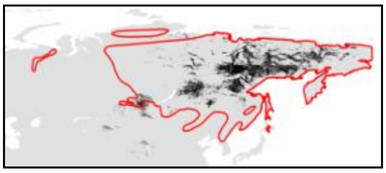
c) ~ 2020s (2010-2039)

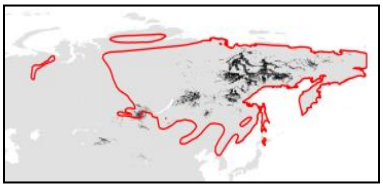
d) ~ 2050s (2040-2069)

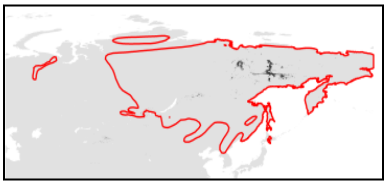
e) ~ 2080s (2070-2099)

Black = predicted suitable envelope

Red polygon = IUCN range (Smith, A.T. & Johnston, C.H. 2008)

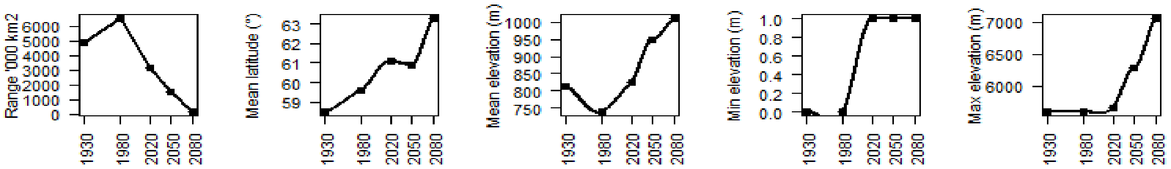

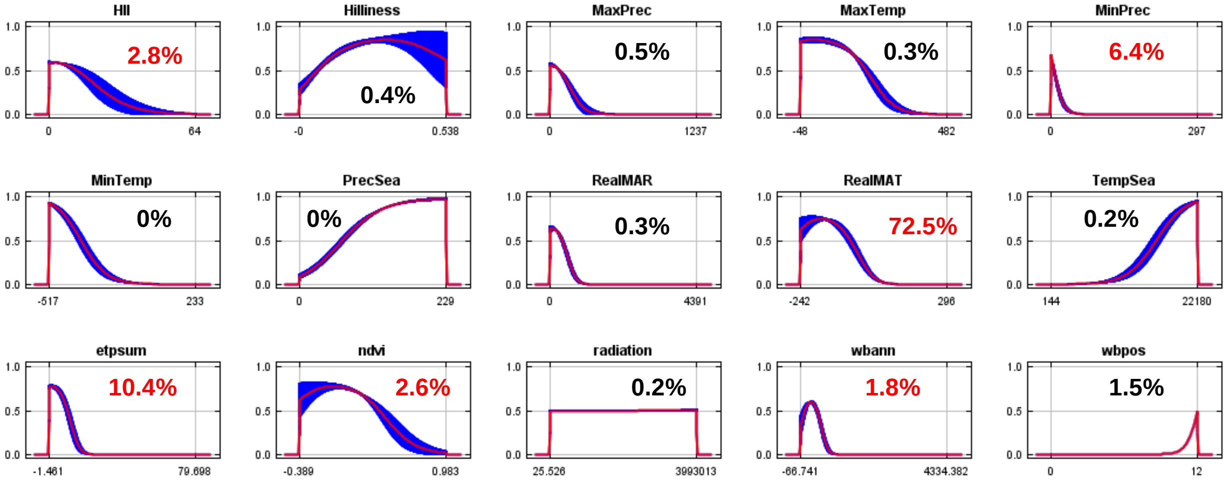

#51– Ili pika (*Ochotona iliensis*)
n = 11
Expert: Andrew Smith, Arizona State University
Expert evaluation: Poor
Data: Only modern
Envelope: Climatic and habitat
Dispersal distance: 1km/year (Similar ecology to *O.koslowi*)
Status: UNMODELLABLE; **Included in final analysis:** X

Model evaluation metric	
AUC	0.99
Omission rate	0.00
Sensitivity	1.00
Specificity	0.99
Proportion correct	0.99
**Kappa**	**0.55**
True Skill Statistic	0.99

Summary: The Ili pika's bioclimatic envelope is predicted to decrease by 20% with a ~1° mean latitudinal polewards shift and a mean increase in elevation of ~80m. 95% of the permutation importance of the model was contributed to by minimum precipitation (52.7%), minimum temperature (22.2%), mean annual temperature (8.8%), maximum precipitation (6.5%), temperature seasonality (3.0%) and human influence index (2.2%).

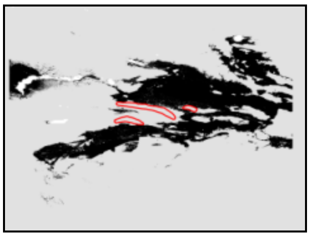
a) ~ 1930s (1900-1949)

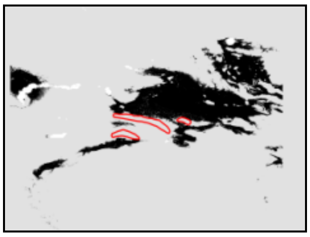
b) ~ 1980s (1950-2000)

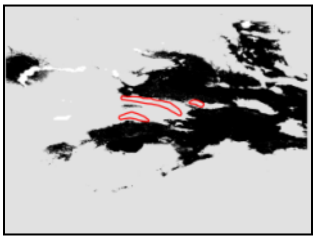
c) ~ 2020s (2010-2039)

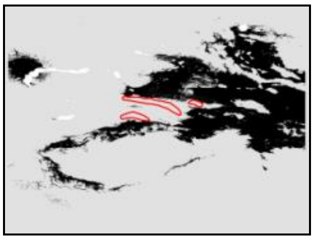
d) ~ 2050s (2040-2069)

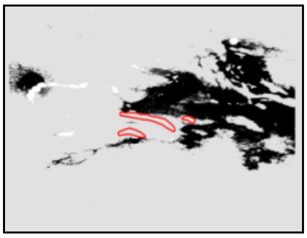
e) ~ 2080s (2070-2099)

Black = predicted suitable envelope

Red polygon = IUCN range (Smith, A.T. & Johnston, C.H. 2008)

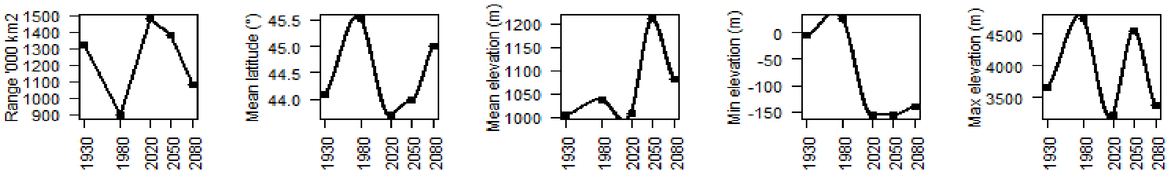

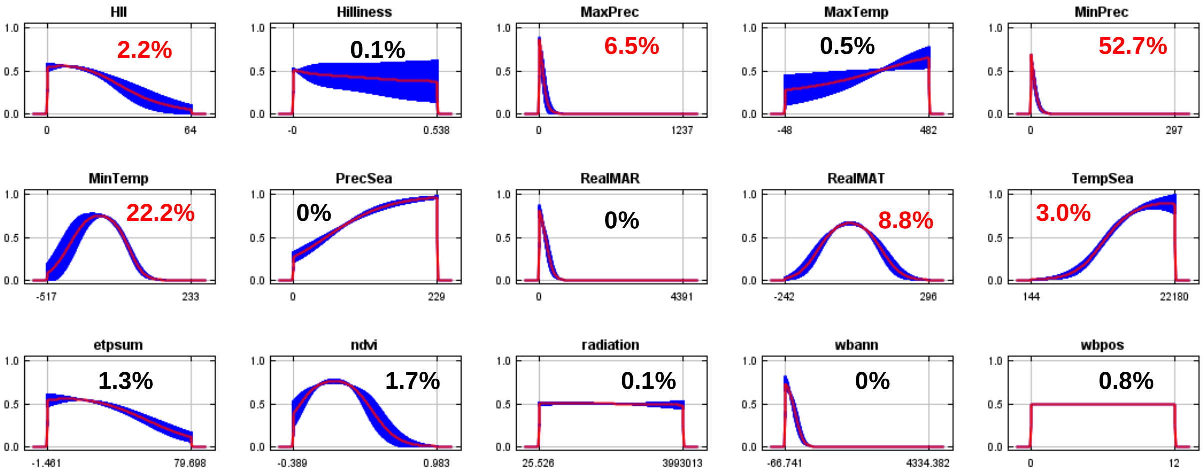

#52 – Kozlov's pika (*Ochotona koslowi*)
n = 5
Expert: Andrew Smith, Arizona State University
Expert evaluation: Medium
Data: Only modern
Envelope: Climatic and habitat
Dispersal distance: 1km/year (Expert)
Status: MODELLABLE; **Included in final analysis:** √

Model evaluation metric	
AUC	0.99
Omission rate	0.00
Sensitivity	1.00
Specificity	0.99
Proportion correct	0.99
**Kappa**	**0.86**
True Skill Statistic	0.99

Summary: The Kozlov's pika's bioclimatic envelope is predicted to decrease by 100% (total extinction). 95% of the permutation importance of the model was contributed to by mean annual precipitation (66.8%), minimum precipitation (24.2%) and human influence index (5.8%).

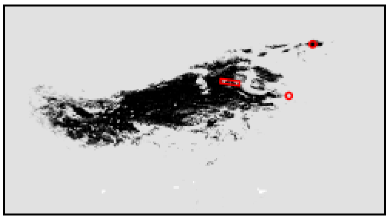
a) ~ 1930s (1900-1949)

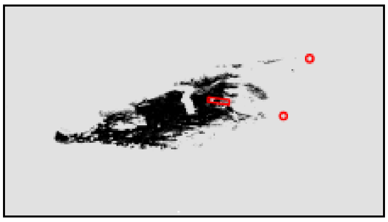
b) ~ 1980s (1950-2000)

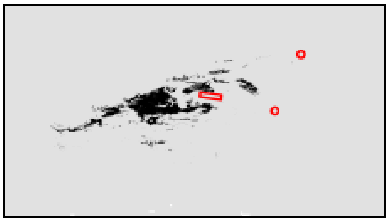
c) ~ 2020s (2010-2039)

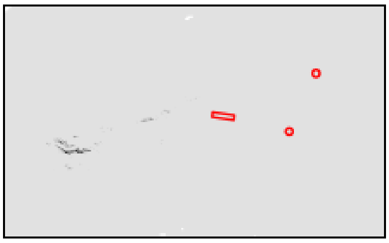
d) ~ 2050s (2040-2069)

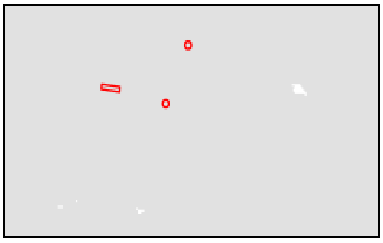
e) ~ 2080s (2070-2099)

Black = predicted suitable envelope

Red polygon = IUCN range (Smith, A.T. & Johnston, C.H. 2008)

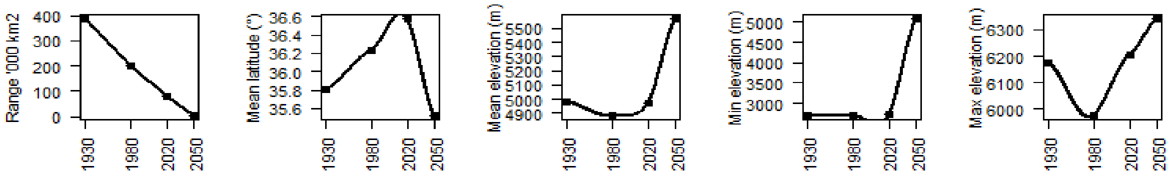

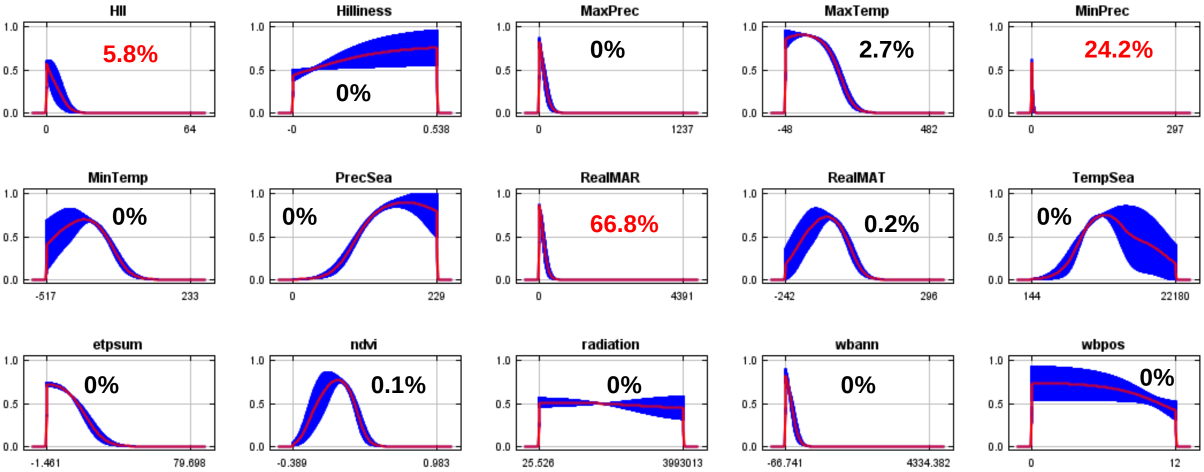

#53 – Ladak pika (*Ochotona ladacensis*)
n = 18
Expert: Andrew Smith, Arizona State University
Expert evaluation: Medium
Data: Modern and historic
Envelope: Climatic and habitat
Dispersal distance: 0.05km/year (Similar ecology to *O.curzoniae*)
Status: MODELLABLE; **Included in final analysis:** √

Model evaluation metric	
AUC	0.99
Omission rate	0.00
Sensitivity	1.00
Specificity	0.99
Proportion correct	0.99
**Kappa**	**0.63**
True Skill Statistic	0.99

Summary: The Ladak pika's bioclimatic envelope is predicted to decrease by 70% with a ~1° mean latitudinal shift towards the Equator and a mean increase in elevation of ~1400m driven by an increase in minimum and maximum elevation. 95% of the permutation importance of the model was contributed to by mean annual precipitation (47.9%), minimum precipitation (41.7%) and minimum temperature (5.7%).

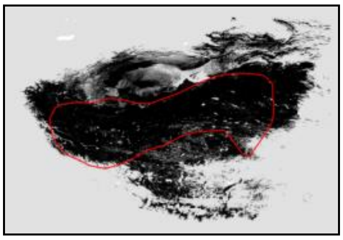
a) ~ 1930s (1900-1949)

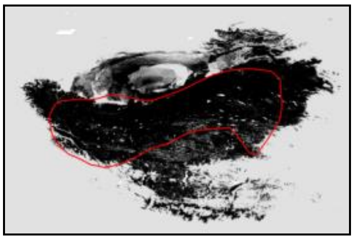
b) ~ 1980s (1950-2000)

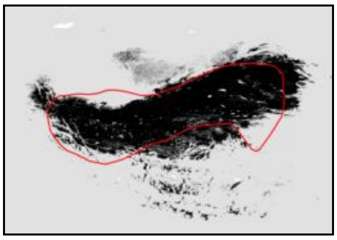
c) ~ 2020s (2010-2039)

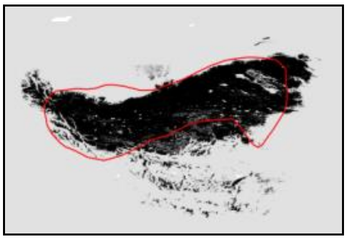
d) ~ 2050s (2040-2069)

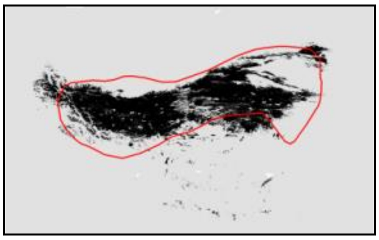
e) ~ 2080s (2070-2099)

Black = predicted suitable envelope

Red polygon = IUCN range (Smith, A.T. & Johnston, C.H. 2008)

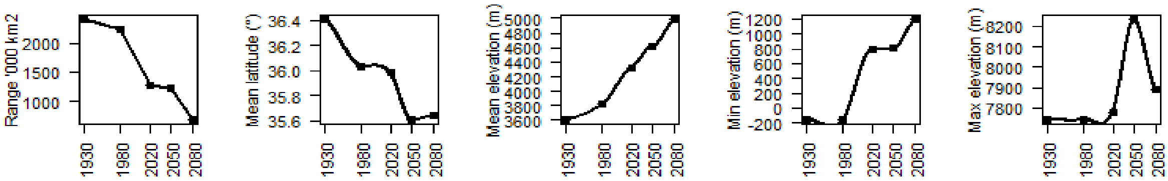

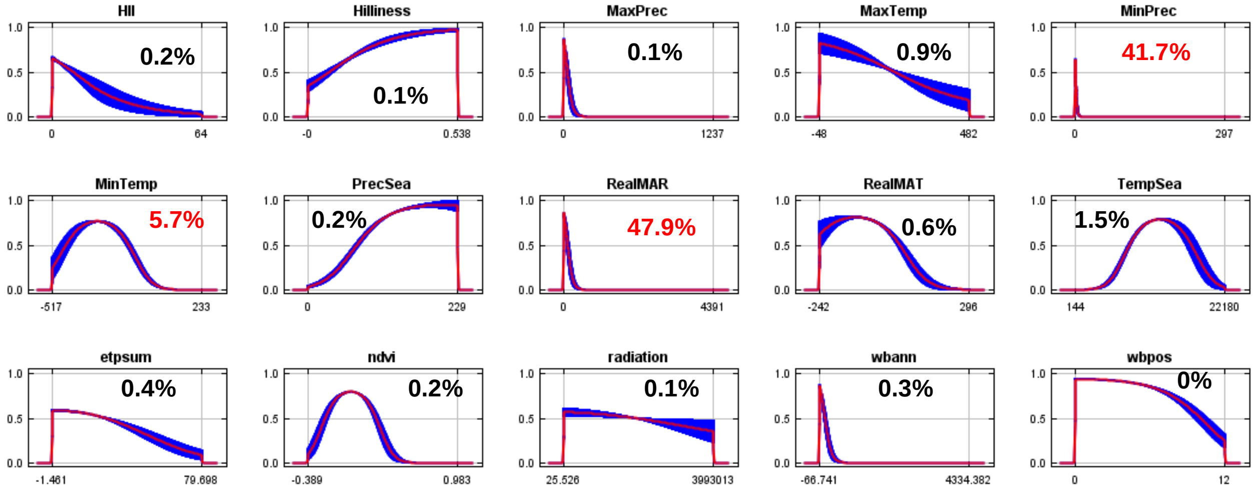

#54 – Large-eared pika (*Ochotona macrotis*)
n = 49
Expert: Nishma Dahal, National Centre for Biological Sciences, India
Expert evaluation: Medium
Data: Modern and historic
Envelope: Climatic and habitat
Dispersal distance: 1km/year (Similar ecology to *O.roylei*)
Status: MODELLABLE; **Included in final analysis:** √

Model evaluation metric	
AUC	0.94
Omission rate	0.10
Sensitivity	0.90
Specificity	0.99
Proportion correct	0.99
**Kappa**	**0.43**
True Skill Statistic	0.89

Summary: The Large-eared pika's bioclimatic envelope is predicted to decrease by 40% with a ~1° mean latitudinal shift towards the Equator and a mean increase in elevation of ~880m driven by an increase in minimum elevation. 95% of the permutation importance of the model was contributed to by minimum precipitation (87.3%), minimum temperature (5.8%) and mean annual temperature (2.1%).

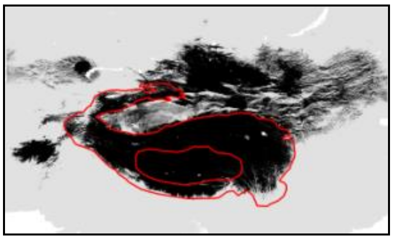
a) ~ 1930s (1900-1949)

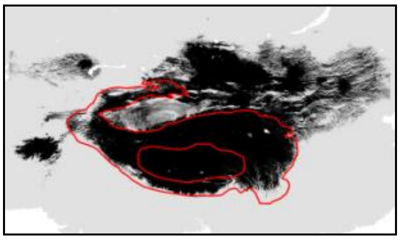
b) ~ 1980s (1950-2000)

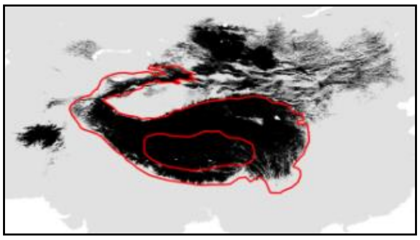
c) ~ 2020s (2010-2039)

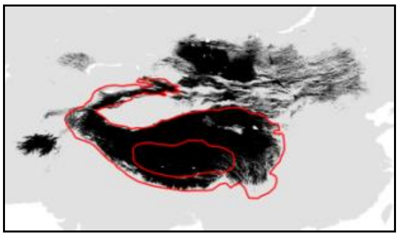
d) ~ 2050s (2040-2069)

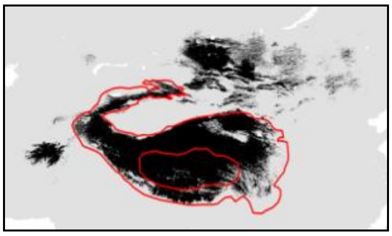
e) ~ 2080s (2070-2099)

Black = predicted suitable envelope

Red polygon = IUCN range (Smith, A.T. & Johnston, C.H. 2008)

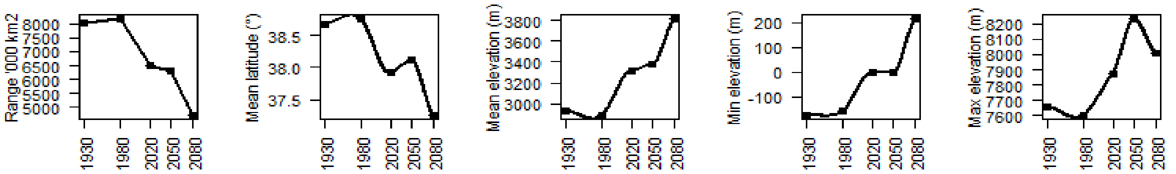

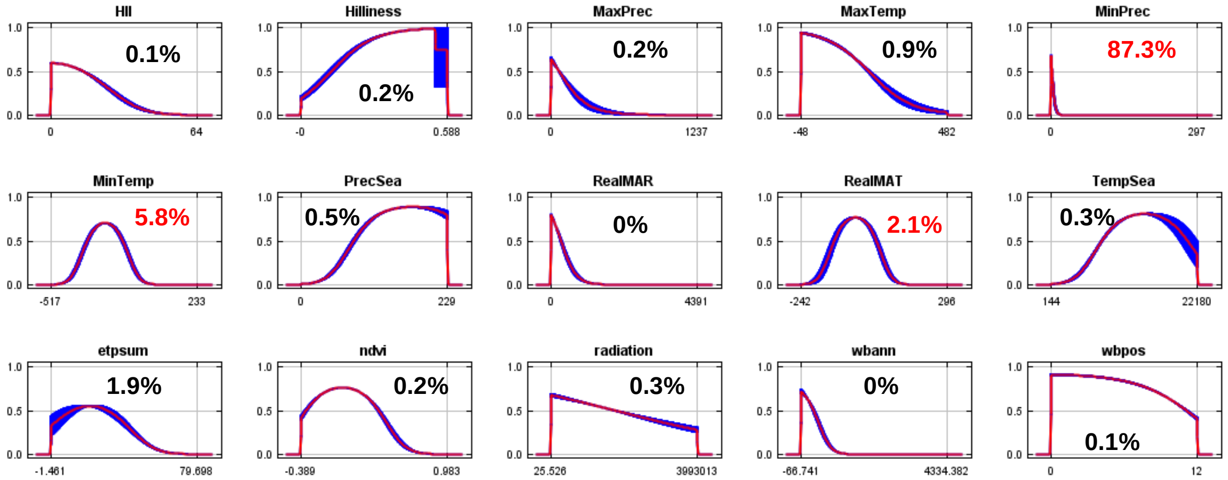

#55 – Nubra's pika (*Ochotona nubrica*)
n = 13
Expert: Nishma Dahal, National Centre for Biological Sciences, India
Expert evaluation: Medium
Data: Only modern
Envelope: Climatic and habitat
Dispersal distance: 0.05km/year (Similar ecology to *O.curzoniae*)
Status: UNMODELLABLE; **Included in final analysis:** X

Model evaluation metric	
AUC	0.99
Omission rate	0.00
Sensitivity	1.00
Specificity	0.99
Proportion correct	0.99
**Kappa**	**0.35**
True Skill Statistic	0.99

Summary: The Nubra's pika's bioclimatic envelope is predicted to increase by 1% with no latitudinal polewards shift, but a mean increase in elevation of ~200m driven by an increase in minimum and maximum elevation. 95% of the permutation importance of the model was contributed to by minimum precipitation (76.1%) and mean annual temperature (20.9%).

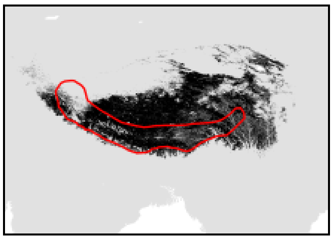
a) ~ 1930s (1900-1949)

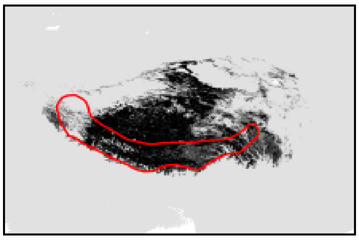
b) ~ 1980s (1950-2000)

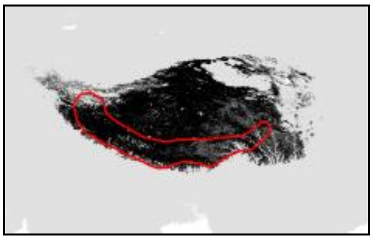
c) ~ 2020s (2010-2039)

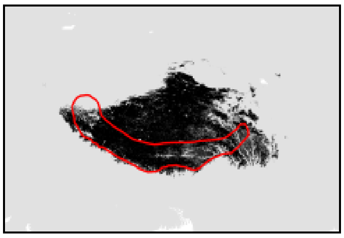
d) ~ 2050s (2040-2069)

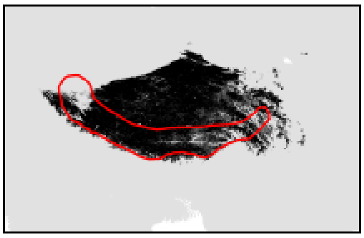
e) ~ 2080s (2070-2099)

Black = predicted suitable envelope

Red polygon = IUCN range (Smith, A.T. & Boyer, A. F. 2008)

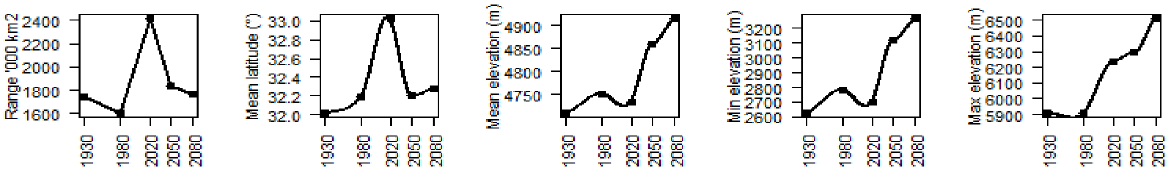

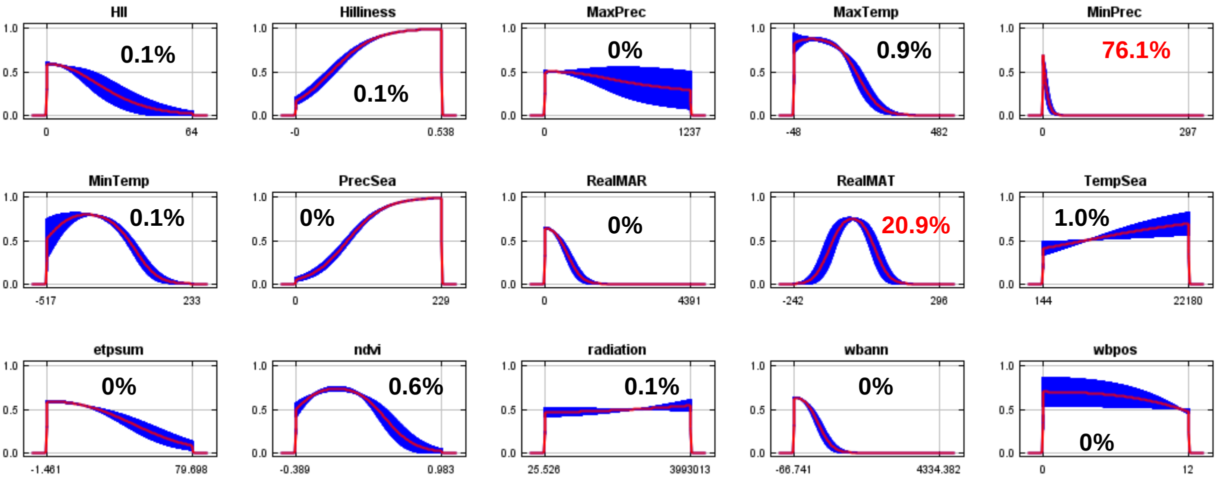

#56 – Pallas's pika (*Ochotona pallasi*)
n = 19
Expert: Andrew Smith, Arizona State University
Expert evaluation: Medium
Data: Only modern
Envelope: Climatic and habitat
Dispersal distance: 10km/year (Sokolov, V.E. *et al*., 2009)
Status: MODELLABLE; **Included in final analysis:** √

Model evaluation metric	
AUC	0.99
Omission rate	0.00
Sensitivity	1.00
Specificity	0.99
Proportion correct	0.99
**Kappa**	**0.46**
True Skill Statistic	0.99

Summary: The Pallas's pika's bioclimatic envelope is predicted to decrease by 60% with a ~2° mean latitudinal polewards shift and a mean increase in elevation of ~40m driven by an increase in minimum elevation. 95% of the permutation importance of the model was contributed to by minimum precipitation (46.1%), mean annual precipitation (35.5%), mean annual temperature (9.4%) and minimum temperature (6.7%).

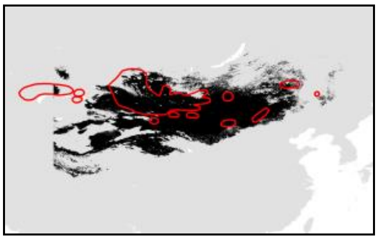
a) ~ 1930s (1900-1949)

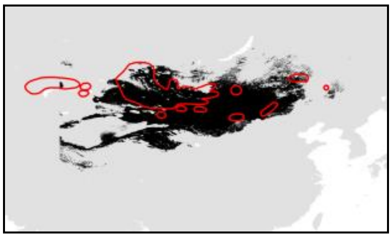
b) ~ 1980s (1950-2000)

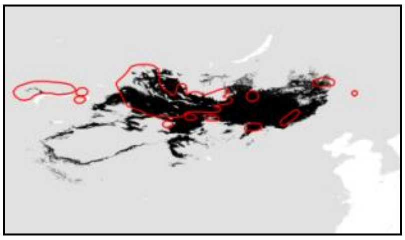
c) ~ 2020s (2010-2039)

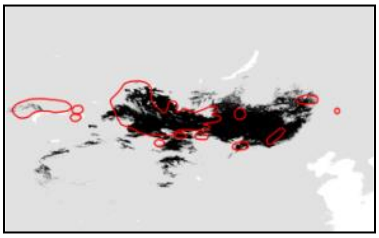
d) ~ 2050s (2040-2069)

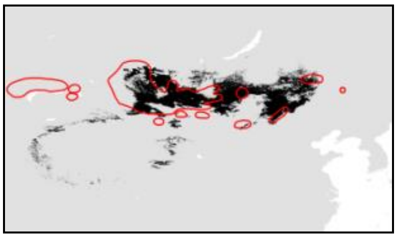
e) ~ 2080s (2070-2099)

Black = predicted suitable envelope

Red polygon = IUCN range (Smith, A.T. & Johnston, C.H. 2008)

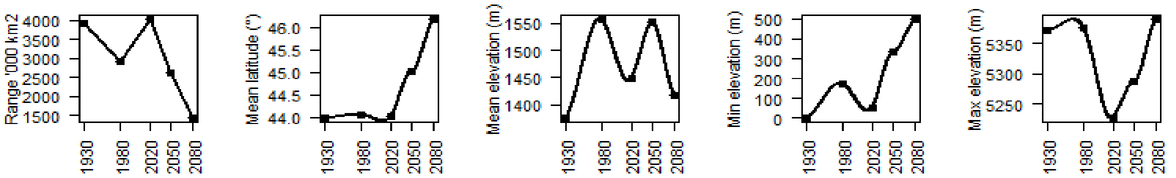

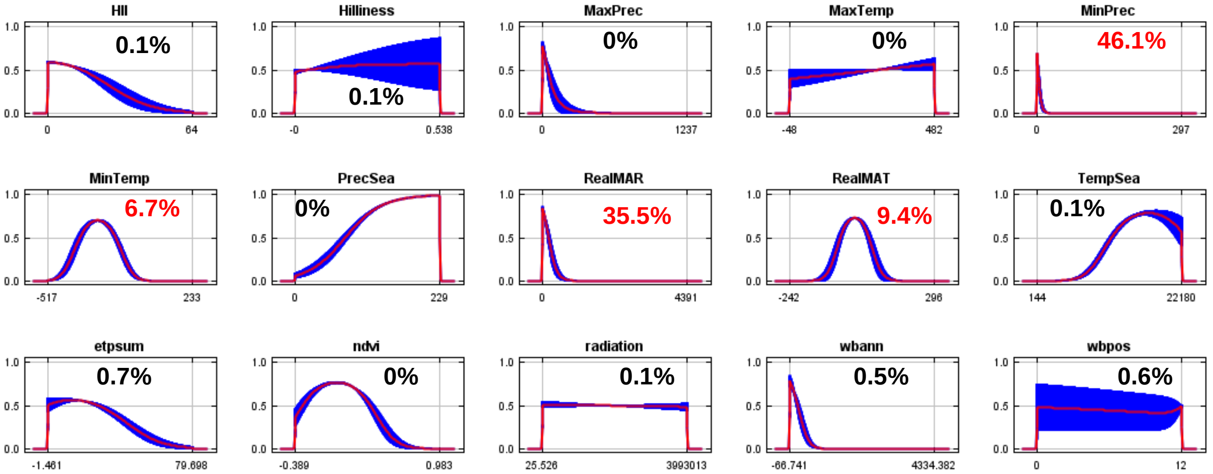

#57 – American pika (*Ochotona princeps*)
n = 670
Expert: Andrew Smith, Arizona State University
Expert evaluation: Medium
Data: Only modern
Envelope: Climatic and habitat
Dispersal distance: 16.1km/year (Expert)
Status: MODELLABLE; **Included in final analysis:** √

Model evaluation metric	
AUC	0.95
Omission rate	0.10
Sensitivity	0.9
Specificity	0.99
Proportion correct	0.98
**Kappa**	**0.87**
True Skill Statistic	0.89

Summary: The American pika's bioclimatic envelope is predicted to decrease by 25% with a ~1° mean latitudinal polewards shift and a mean decrease in elevation of ~10m driven by an decrease in minimum elevation. 95% of the permutation importance of the model was contributed to by mean annual temperature (88.1%), annual evapotranspiration (5.0%) and maximum temperature (2.8%).

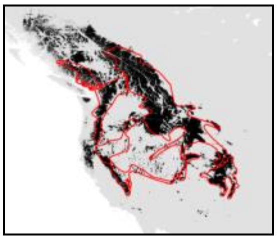
a) ~ 1930s (1900-1949)

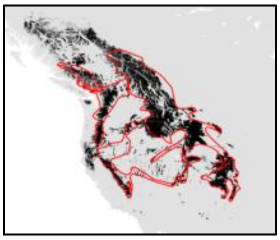
b) ~ 1980s (1950-2000)

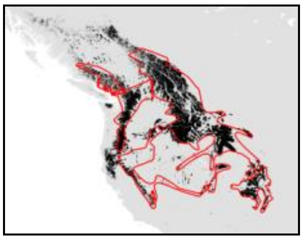
c) ~ 2020s (2010-2039)

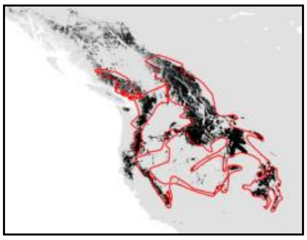
d) ~ 2050s (2040-2069)

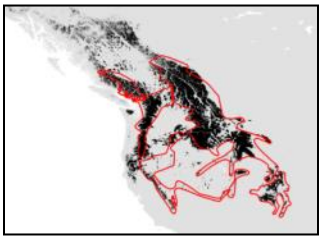
e) ~ 2080s (2070-2099)

Black = predicted suitable envelope

Red polygon = IUCN range (Beever, E. & Smith, A.T. 2011)

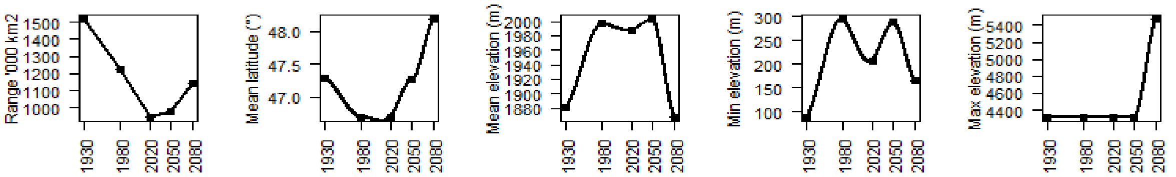

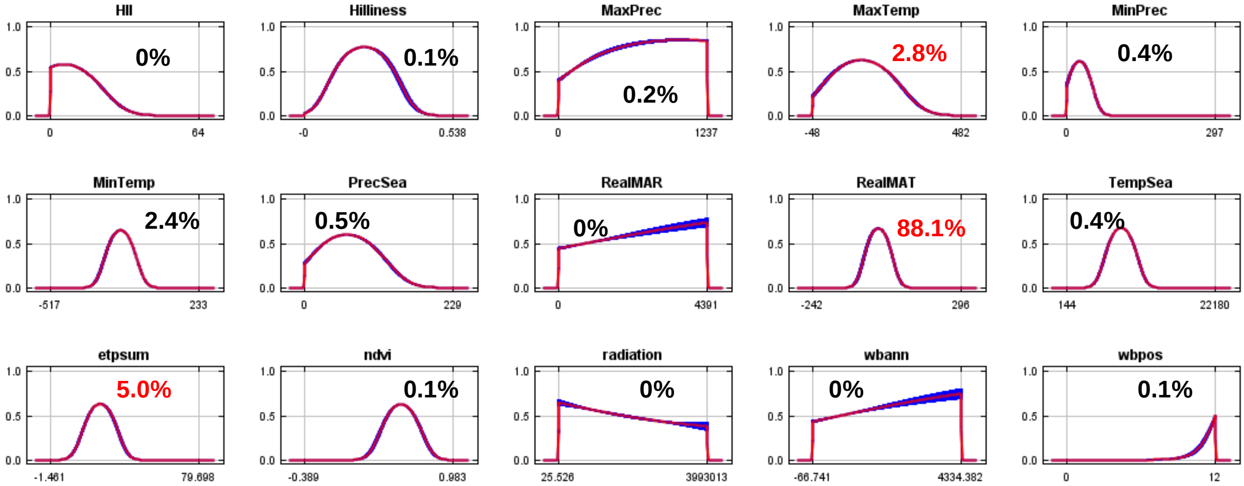

#58 – Little pika (*Ochotona pusilla*)
n = 30
Expert: Andrew Smith, Arizona State University
Expert evaluation: Medium
Data: Modern and historic
Envelope: Climatic and habitat
Dispersal distance: 4km/year (Sokolov, V.E. *et al*., 2009)
Status: MODELLABLE; **Included in final analysis:** √

Model evaluation metric	
AUC	0.93
Omission rate	0.13
Sensitivity	0.87
Specificity	0.99
Proportion correct	0.99
**Kappa**	**0.58**
True Skill Statistic	0.86

Summary: The Little pika's bioclimatic envelope is predicted to increase by 20% with a ~1° mean latitudinal polewards shift and a mean increase in elevation of ~70m driven by an increase in maximum elevation. 95% of the permutation importance of the model was contributed to by temperature seasonality (70.2%), annual water balance (13.3%), mean annual temperature (10.2%) and minimum temperature (4.5%).

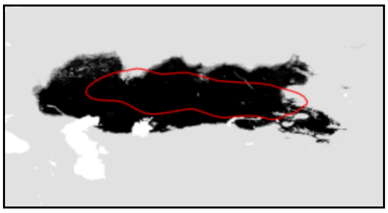
a) ~ 1930s (1900-1949)

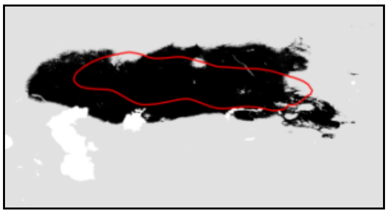
b) ~ 1980s (1950-2000)

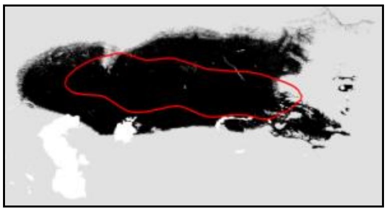
c) ~ 2020s (2010-2039)

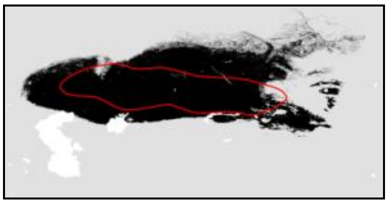
d) ~ 2050s (2040-2069)

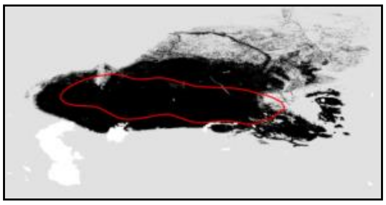
e) ~ 2080s (2070-2099)

Black = predicted suitable envelope

Red polygon = IUCN range (Smith, A.T. & Johnston, C.H. 2008)

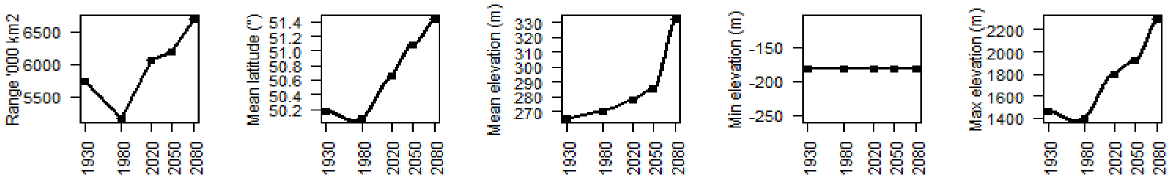

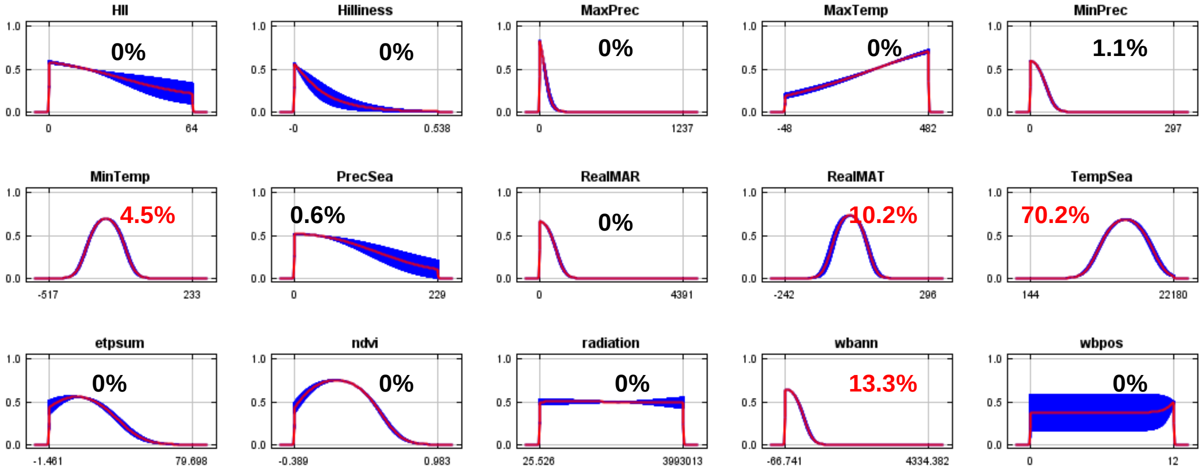

#59 – Royle's pika (*Ochotona roylei*)
n = 22
Expert: Sabuj Bhattacharya, Wildlife Institute of India
Expert evaluation: Medium
Data: Modern and historic
Envelope: Climatic and habitat
Dispersal distance: 1km/year (Expert)
Status: MODELLABLE; **Included in final analysis:** √

Model evaluation metric	
AUC	0.98
Omission rate	0.05
Sensitivity	0.95
Specificity	0.99
Proportion correct	0.99
**Kappa**	**0.76**
True Skill Statistic	0.95

Summary: The Royle's pika's bioclimatic envelope is predicted to decrease by 20% with no latitudinal polewards shift and a mean increase in elevation of ~340m driven by an increase in minimum elevation. 95% of the permutation importance of the model was contributed to by minimum precipitation (53.2%), maximum temperature (20.4%), temperature seasonality (10.2%), human influence index (5.5%), surface roughness index (4.2%) and precipitation seasonality (3.2%).

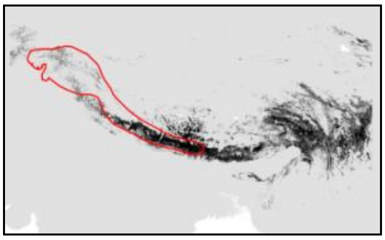
a) ~ 1930s (1900-1949)

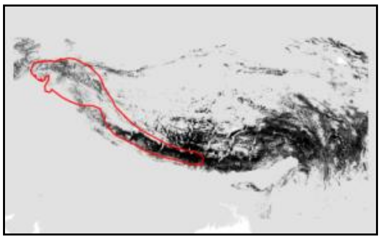
b) ~ 1980s (1950-2000)

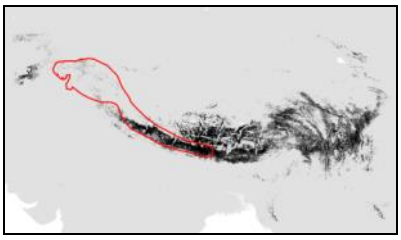
c) ~ 2020s (2010-2039)

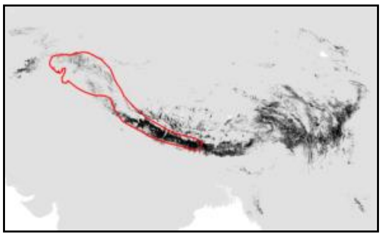
d) ~ 2050s (2040-2069)

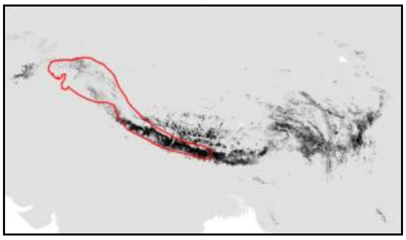
e) ~ 2080s (2070-2099)

Black = predicted suitable envelope

Red polygon = IUCN range (Smith, A.T. & Boyer, A.F. 2008)

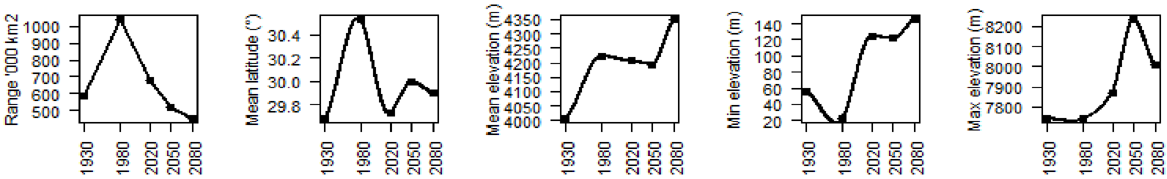

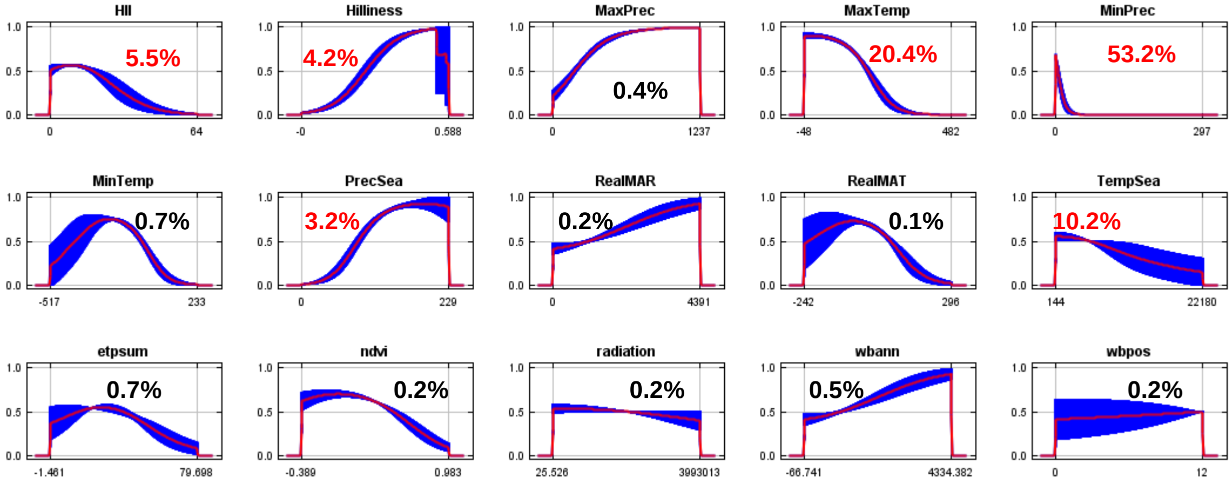

#60 – Afghan pika (*Ochotona rufescens*)
n = 17
Expert: Chelmala Srinivasulu, Osmania University, India
Expert evaluation: Medium
Data: Modern and historic
Envelope: Climatic and habitat
Dispersal distance: 3km/year (Asian pikas, range 1-15)
Status: MODELLABLE; **Included in final analysis:** √

Model evaluation metric	
AUC	0.99
Omission rate	0.00
Sensitivity	1.00
Specificity	0.99
Proportion correct	0.99
**Kappa**	**0.74**
True Skill Statistic	0.99

Summary: The Afghan pika's bioclimatic envelope is predicted to increase by 5% with a ~1° mean latitudinal polewards shift and a mean increase in elevation of ~380m driven by an increase in maximum elevation. 95% of the permutation importance of the model was contributed to by minimum precipitation (89.2%), minimum temperature (5.5%) and normalised difference vegetation index (2.3%).

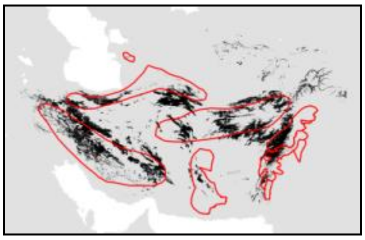

a) ~ 1930s (1900-1949)

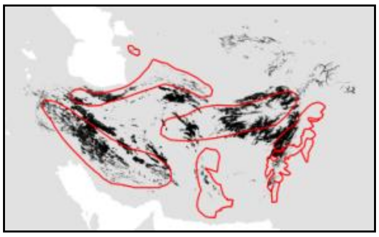

b) ~ 1980s (1950-2000)

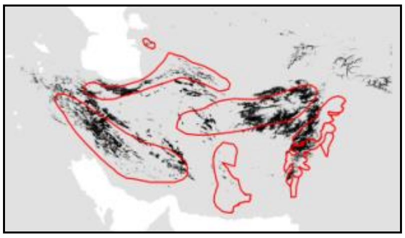

c) ~ 2020s (2010-2039)

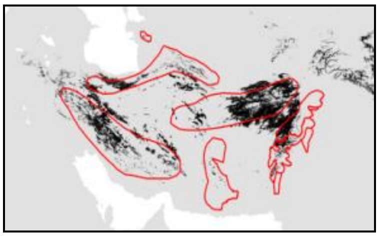

d) ~ 2050s (2040-2069)

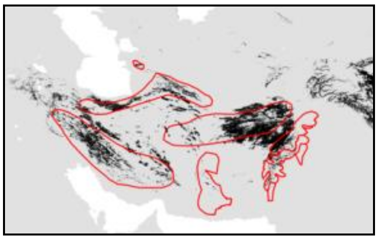

e) ~ 2080s (2070-2099)

Black = predicted suitable envelope

Red polygon = IUCN range (Smith, A.T. & Boyer, A.F. 2008)

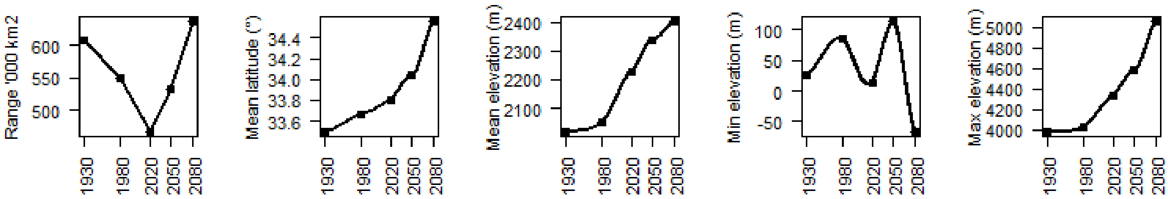

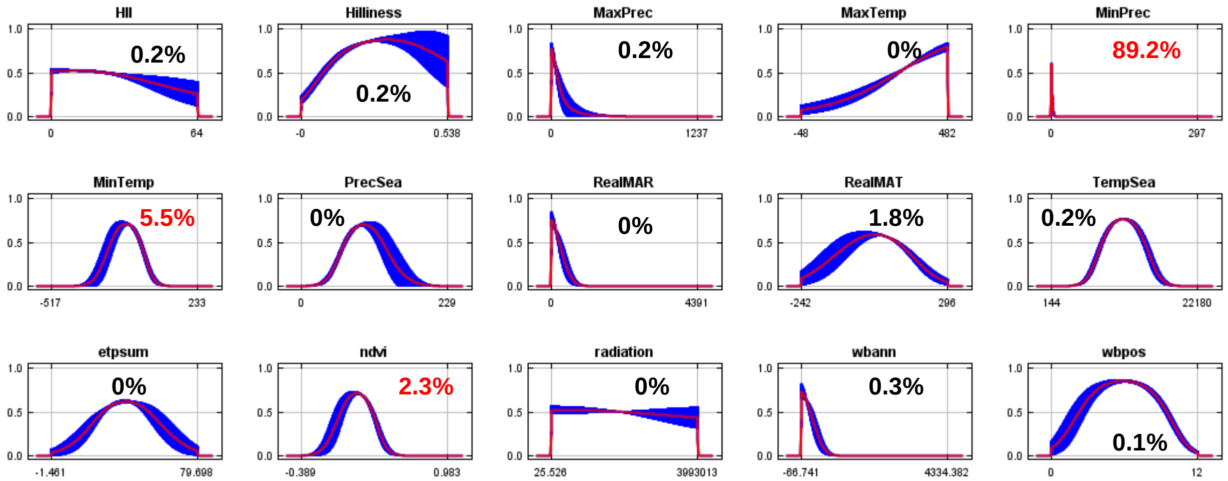

Model evaluation metric	
AUC	0.99
Omission rate	0.00
Sensitivity	1.00
Specificity	0.99
Proportion correct	0.99
**Kappa**	**0.25**
True Skill Statistic	0.99

#61 – Turkestan red pika (*Ochotona rutila*)
n = 13
Expert: Andrey Lissovsky, Zoological Museum of Moscow State University
Expert evaluation: Poor
Data: Modern and historic
Envelope: Climatic and habitat
Dispersal distance: 3km/year (Asian pikas, range 1-15)
Status: UNMODELLABLE; **Included in final analysis:** X

Summary: The Turkestan red pika's bioclimatic envelope is predicted to decrease by 10% with a ~1° mean latitudinal shift towards the Equator and a mean increase in elevation of ~630m driven by an increase in maximum elevation. 95% of the permutation importance of the model was contributed to by minimum precipitation (82.5%), minimum temperature (5.9%), human influence index (3.0%), precipitation seasonality (2.5%) and surface roughness index (1.7%).

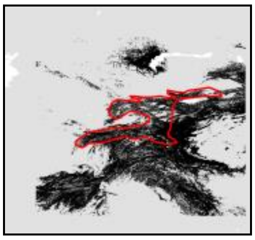
a) ~ 1930s (1900-1949)

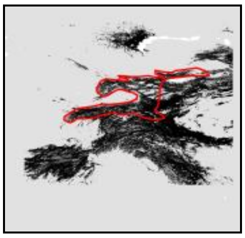
b) ~ 1980s (1950-2000)

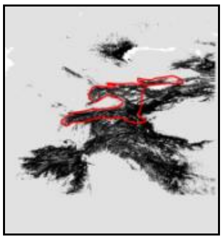
c) ~ 2020s (2010-2039)

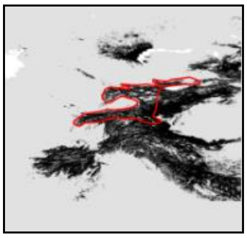
d) ~ 2050s (2040-2069)

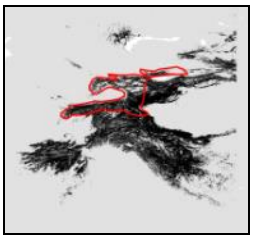
e) ~ 2080s (2070-2099)

Black = predicted suitable envelope

Red polygon = IUCN range (Smith, A.T. & Boyer, A.F. 2008)

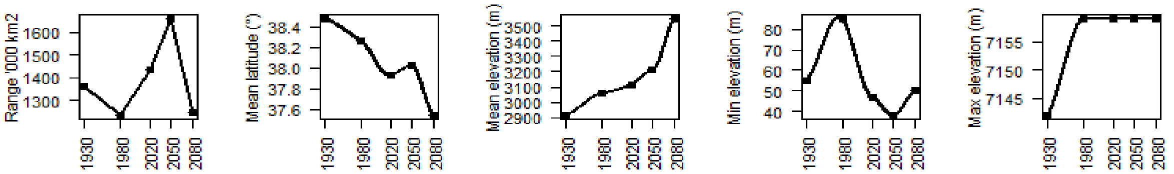

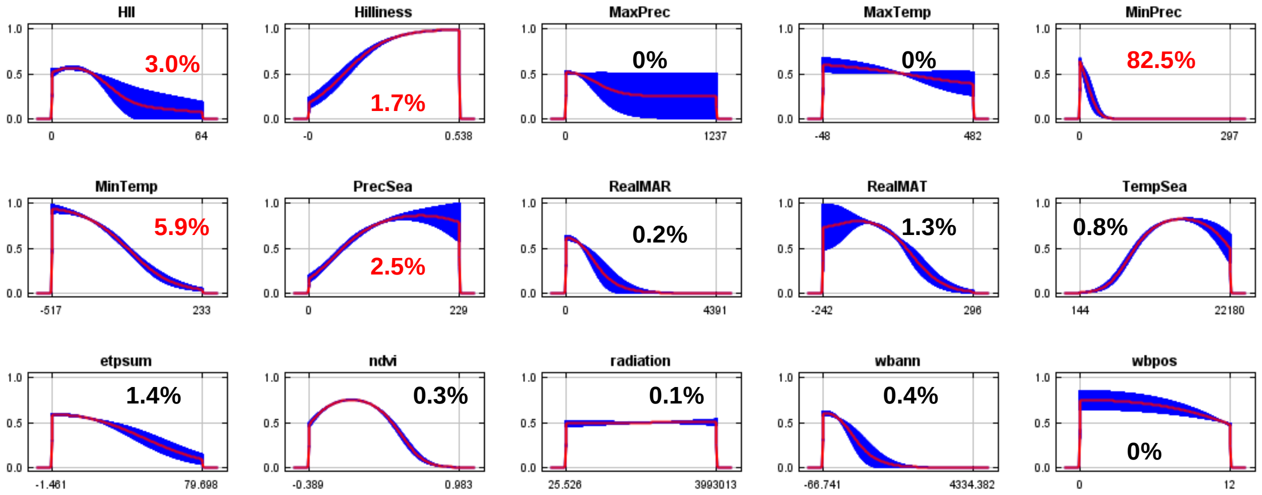

#62 – Moupin pika (*Ochotona thibetana*)
n = 95
Expert: Deyan Ge, Institute of Zoology, Chinese Academy of Sciences
Expert evaluation: Poor
Data: Modern and historic
Envelope: Climatic and habitat
Dispersal distance: 2km/year (Similar ecology to *O.roylei*)
Status: UNMODELLABLE; **Included in final analysis:** X

Model evaluation metric	
AUC	0.93
Omission rate	0.13
Sensitivity	0.87
Specificity	0.99
Proportion correct	0.99
**Kappa**	**0.52**
True Skill Statistic	0.86

Summary: The Moupin pika's bioclimatic envelope is predicted to increase by 10% with a ~2° mean latitudinal polewards shift and a mean increase in elevation of ~370m driven by an increase in maximum elevation. 95% of the permutation importance of the model was contributed to by minimum precipitation (43.5%), maximum temperature (38.5%), temperature seasonality (7.7%), annual evapotranspiration (3.7%) and mean annual precipitation (3.1%).

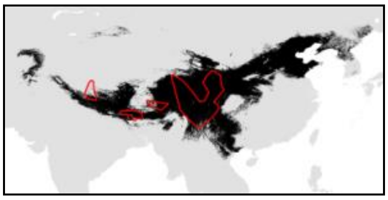

a) ~ 1930s (1900-1949)

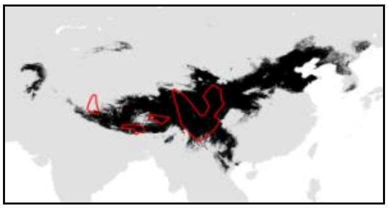

b) ~ 1980s (1950-2000)

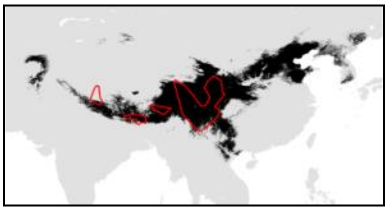

c) ~ 2020s (2010-2039)

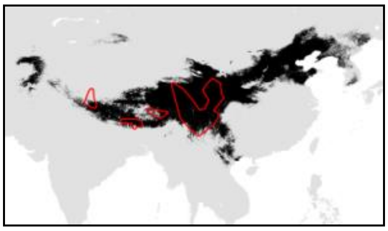

d) ~ 2050s (2040-2069)

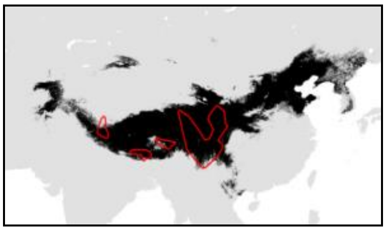

e) ~ 2080s (2070-2099)

Black = predicted suitable envelope

Red polygon = IUCN range (Smith, A.T. & Boyer, A.F. 2008)

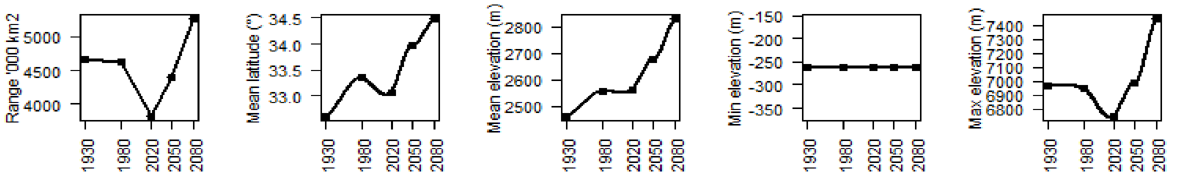

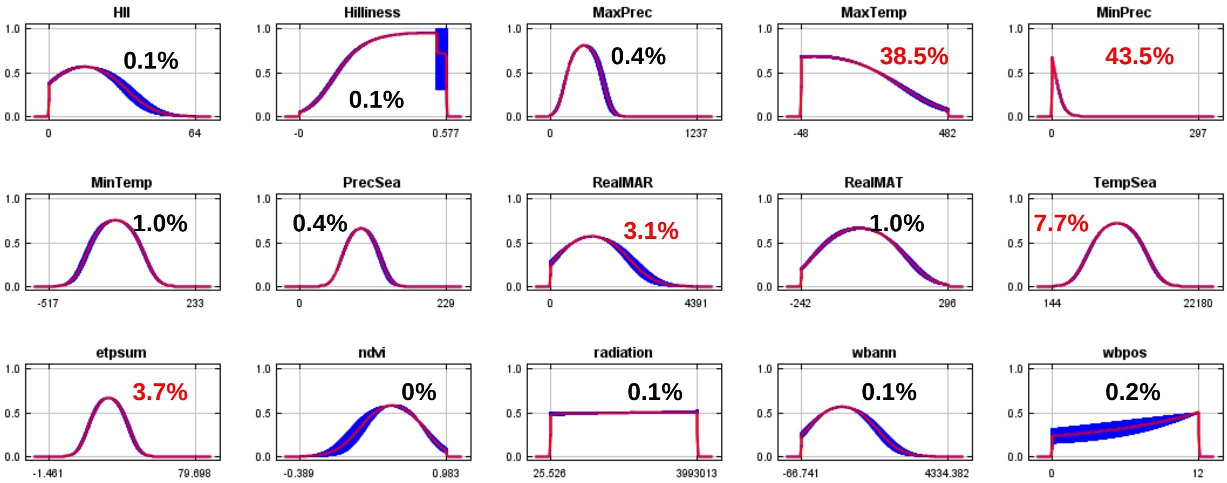

#63 – Thomas's pika (*Ochotona thomasi*)
n = 16
Expert: Andrew Smith, Arizona State University
Expert evaluation: Good
Data: Modern and historic
Envelope: Climatic and habitat
Dispersal distance: 1km/year (Similar ecology to *O.koslowi*)
Status: MODELLABLE; **Included in final analysis:** √

Model evaluation metric	
AUC	0.99
Omission rate	0.00
Sensitivity	1.00
Specificity	0.99
Proportion correct	0.99
**Kappa**	**0.58**
True Skill Statistic	0.99

Summary: The Thomas's pika's bioclimatic envelope is predicted to decrease by 70% with a ~1.5° mean latitudinal polewards shift and a mean increase in elevation of ~590m driven by an increase in maximum and minimum elevation. 95% of the permutation importance of the model was contributed to by minimum precipitation (56.6%), mean annual temperature (23.4%), minimum temperature (14.2%) and evapotranspiration (2.8%).

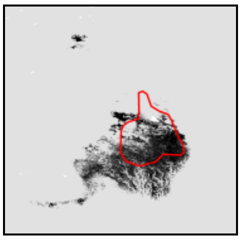
a) ~ 1930s (1900-1949)

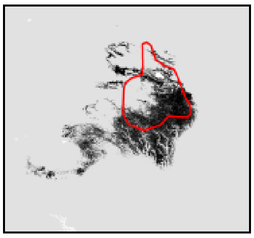
b) ~ 1980s (1950-2000)

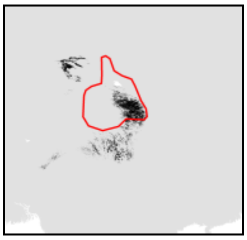
c) ~ 2020s (2010-2039)

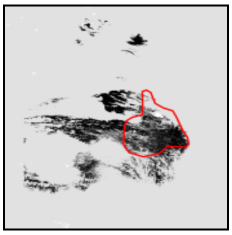
d) ~ 2050s (2040-2069)

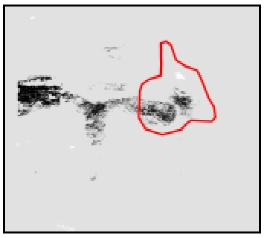
e) ~ 2080s (2070-2099)

Black = predicted suitable envelope

Red polygon = IUCN range (Smith, A.T. & Boyer, A.F. 2008)

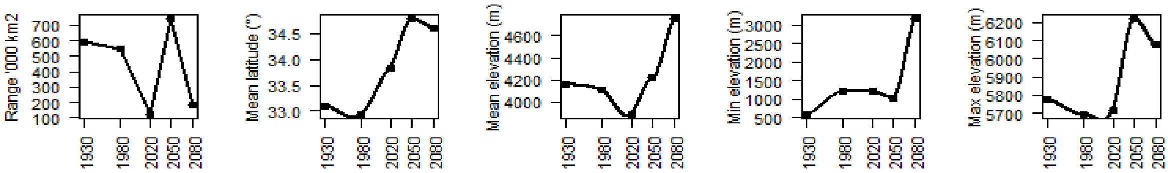

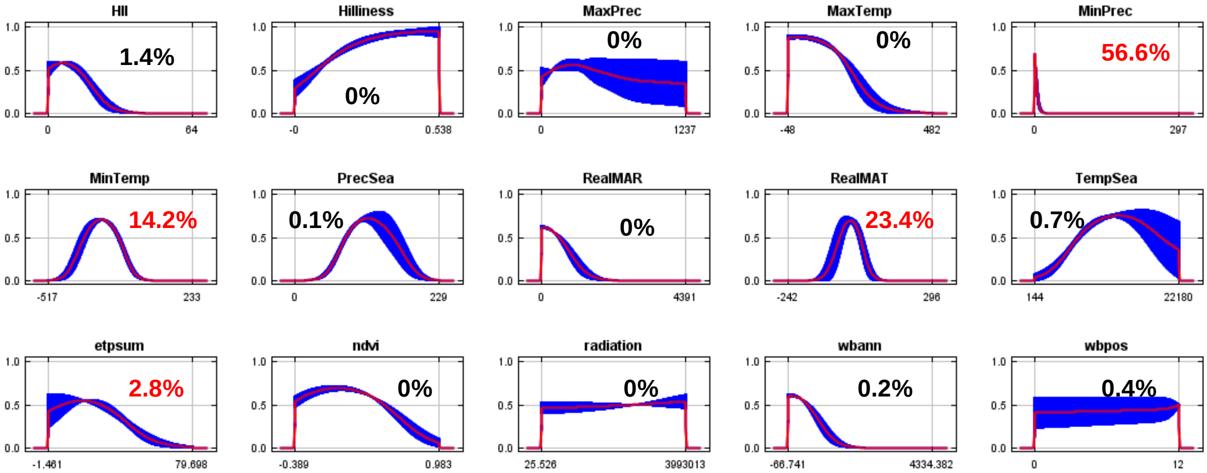

#64 – Turuchan pika (*Ochotona turuchanensis*)
n = 30
Expert: Andrey Lissovsky, Zoological Museum of Moscow State University
Expert evaluation: Medium
Data: Modern and historic
Envelope: Climatic and habitat
Dispersal distance: 15km/year (Expert)
Status: MODELLABLE; **Included in final analysis:** √

Model evaluation metric	
AUC	0.93
Omission rate	0.13
Sensitivity	0.87
Specificity	0.99
Proportion correct	0.99
**Kappa**	**0.50**
True Skill Statistic	0.86

Summary: The Turuchan pika's bioclimatic envelope is predicted to increase by 50% with a ~2° mean latitudinal polewards shift and a mean increase in elevation of ~70m driven by an increase in minimum elevation. 95% of the permutation importance of the model was contributed to by minimum temperature (21.6%), minimum precipitation (19.9%), human influence index (15.2%), annual evapotranspiration (10.9%).

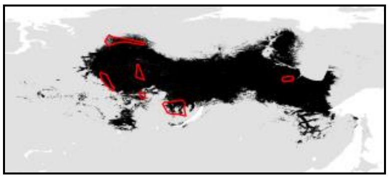
a) ~ 1930s (1900-1949)

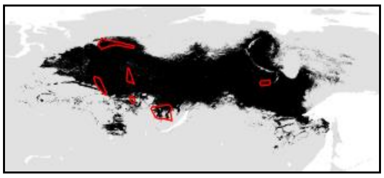
b) ~ 1980s (1950-2000)

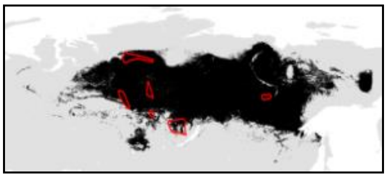
c) ~ 2020s (2010-2039)

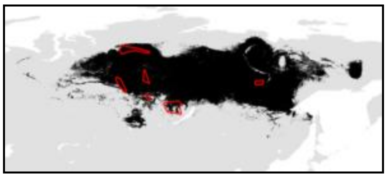
d) ~ 2050s (2040-2069)

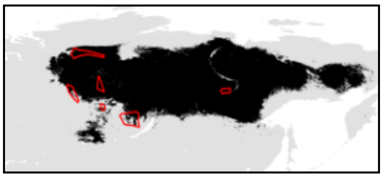
e) ~ 2080s (2070-2099)

Black = predicted suitable envelope

Red polygon = IUCN range (Smith, A.T. & Johnston, C.H. 2008)

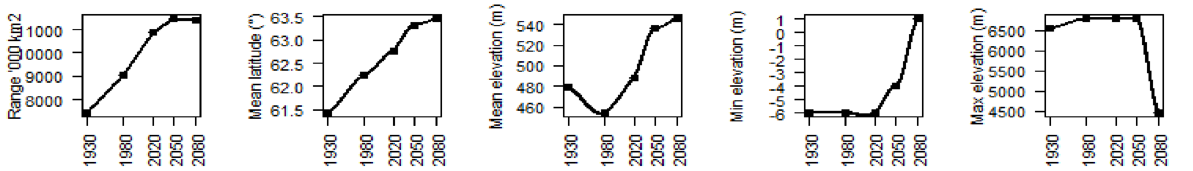

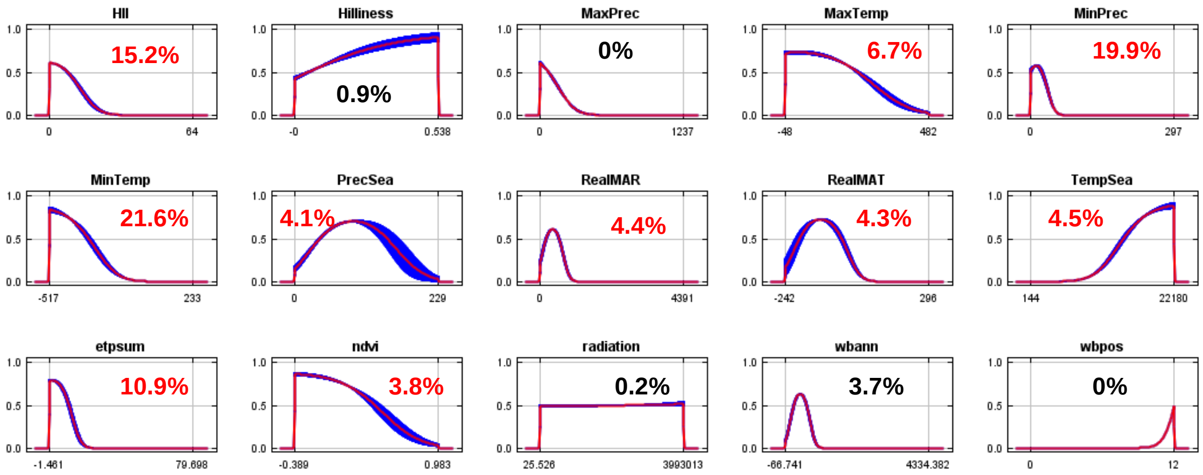

#65 – European rabbit (*Oryctolagus cunciculus*)
n = 22,712
Expert: Neil Reid, Queen's University Belfast
Expert evaluation: Medium
Data: Modern and historic
Envelope: Climatic and habitat
Dispersal distance: 1km/year (Expert)
Status: UNMODELLABLE; **Included in final analysis:** X

Model evaluation metric	
AUC	0.62
Omission rate	0.16
Sensitivity	0.84
Specificity	0.39
Proportion correct	0.62
**Kappa**	**0.23**
True Skill Statistic	0.23

Summary: The European rabbit's bioclimatic envelope is predicted to increase by 30% with a ~2° mean latitudinal polewards shift and a mean decrease in elevation of ~10m. 95% of the permutation importance of the model was contributed to by annual evapotranspiration (21.1%), mean annual temperature (19.0%), minimum temperature (13.9%), annual water balance (12.3%), mean annual precipitation (11.2%), human influence index (9.8%), precipitation seasonality (7.6%) and normalised difference vegetation index (2.9%).

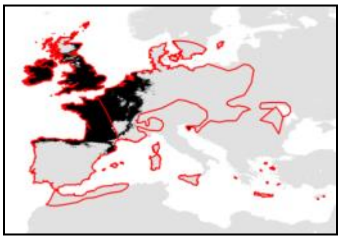

a) ~ 1930s (1900-1949)

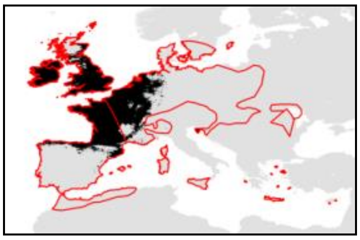

b) ~ 1980s (1950-2000)

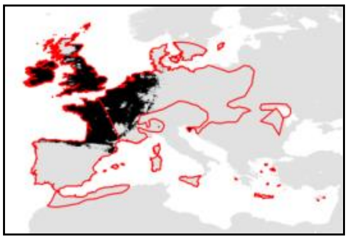

c) ~ 2020s (2010-2039)

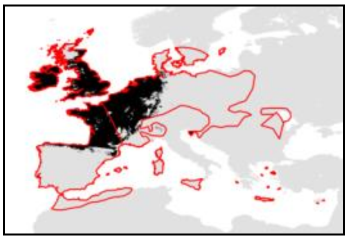

d) ~ 2050s (2040-2069)

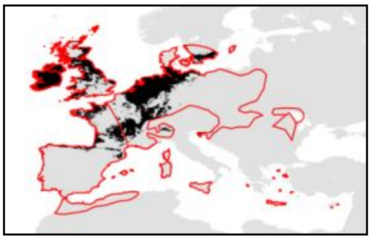

e) ~ 2080s (2070-2099)

Black = predicted suitable envelope

Red polygon = IUCN range (Smith, A.T. & Boyer, A.F. 2008)

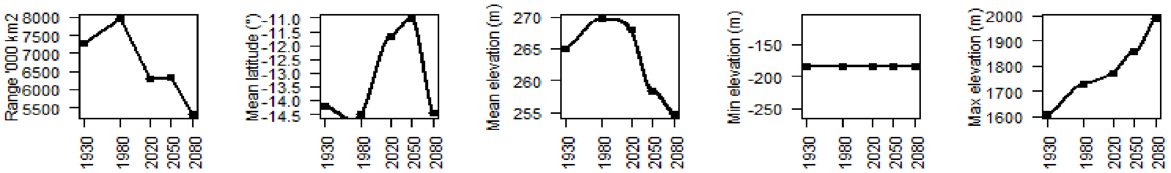

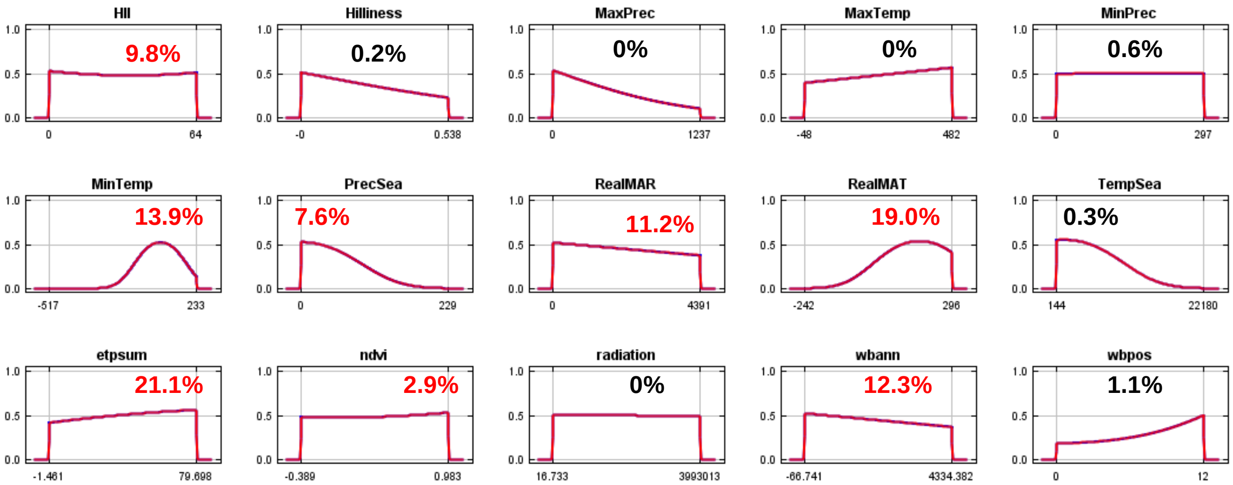

#66 – Amami rabbit (*Pentalagus furnessi*)
n = 9
Expert: Fumio Yamada, Forestry and Forest Products Research Institute, Japan
Expert evaluation: Good
Data: Modern and historic
Envelope: Climatic and habitat
Dispersal distance: 0.01km/year (Expert)
Status: MODELLABLE; **Included in final analysis:** √

Model evaluation metric	
AUC	0.99
Omission rate	0.00
Sensitivity	0.99
Specificity	0.99
Proportion correct	0.99
**Kappa**	**0.95**
True Skill Statistic	0.99

Summary: The Amami rabbit's bioclimatic envelope is predicted to increase by 150% with a ~1° mean latitudinal shift towards the Equator and a mean decrease in elevation of ~25m. 95% of the permutation importance of the model was contributed to by minimum temperature (97.3%), temperature seasonality (1.7%) and annual evapotranspiration (0.8%).

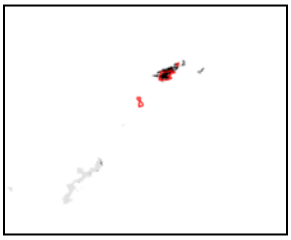
a) ~ 1930s (1900-1949)

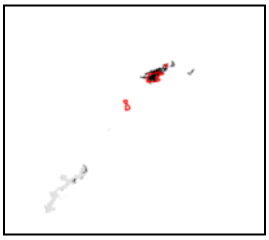
b) ~ 1980s (1950-2000)

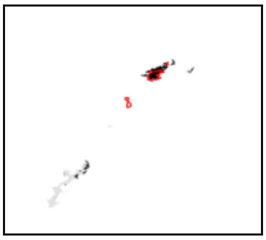
c) ~ 2020s (2010-2039)

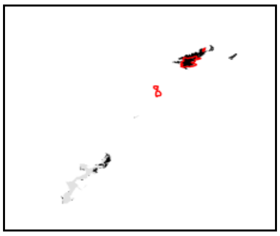
d) ~ 2050s (2040-2069)

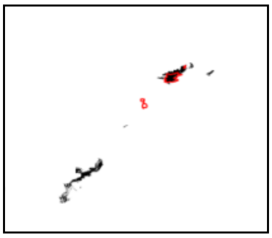
e) ~ 2080s (2070-2099)

Black = predicted suitable envelope

Red polygon = IUCN range (Yamada, F & Sugimura, K. 2008)

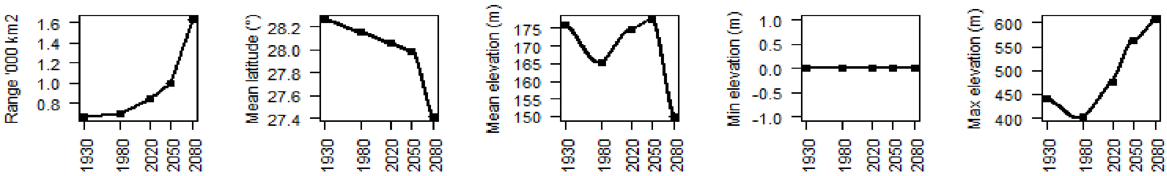

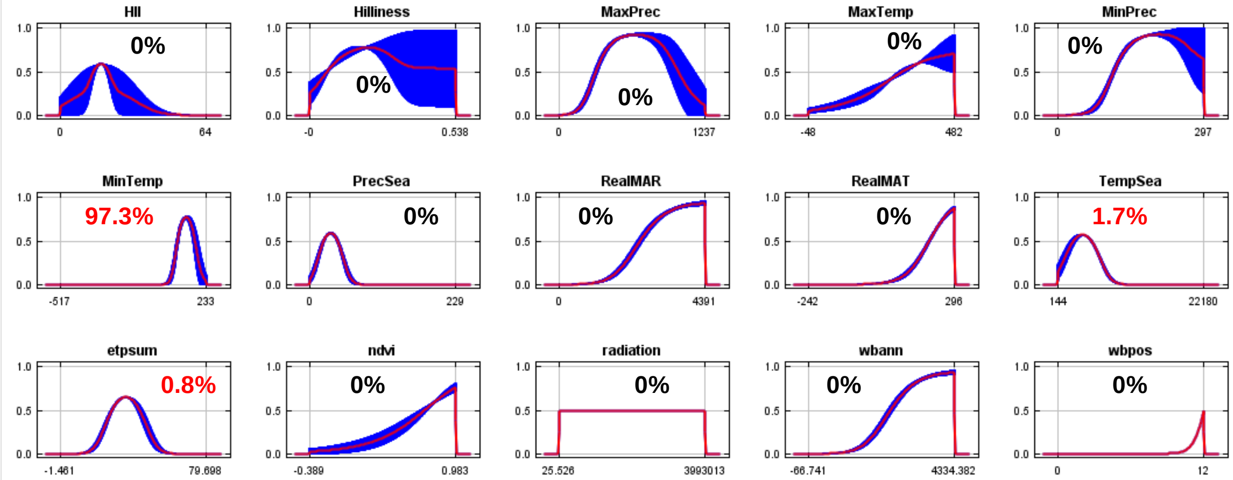

#67 – Bunyoro rabbit (*Poelagus marjorita*)
n = 8
Expert: David Happold, Australian National University
Expert evaluation: Poor
Data: Only modern
Envelope: Climatic and habitat
Dispersal distance: 2km/year (Similar ecology to Pronolagus sp.)
Status: UNMODELLABLE; **Included in final analysis:** X

Model evaluation metric	
AUC	0.99
Omission rate	0.00
Sensitivity	1.00
Specificity	0.99
Proportion correct	0.99
**Kappa**	**0.89**
True Skill Statistic	0.99

Summary: The Bunyoro rabbit's bioclimatic envelope is predicted to decrease by 90% with a ~1° mean latitudinal shift towards the Equator and a mean increase in elevation of ~200m driven by an increase in minimum elevation.. 95% of the permutation importance of the model was contributed to by annual evapotranspiration (61.7%), temperature seasonality (31.4%) and number of months with a positive water balance (6.3%).

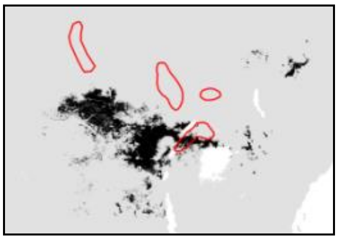
a) ~ 1930s (1900-1949)

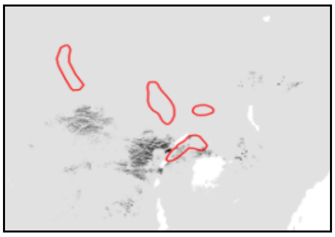
b) ~ 1980s (1950-2000)

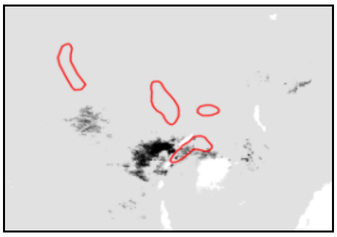
c) ~ 2020s (2010-2039)

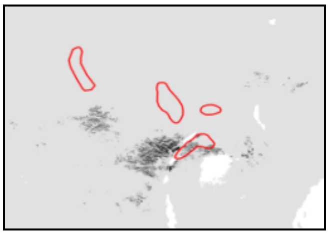
d) ~ 2050s (2040-2069)

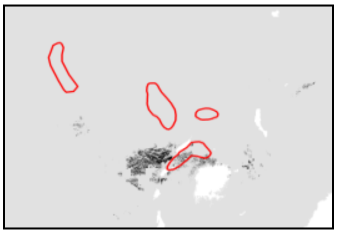
e) ~ 2080s (2070-2099)

Black = predicted suitable envelope

Red polygon = IUCN range (Collins, K. & Smith, A.T. 2008)

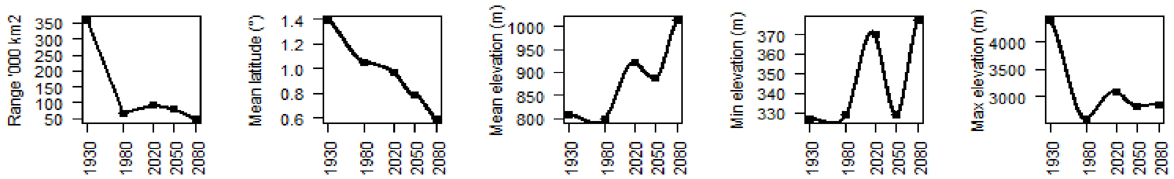

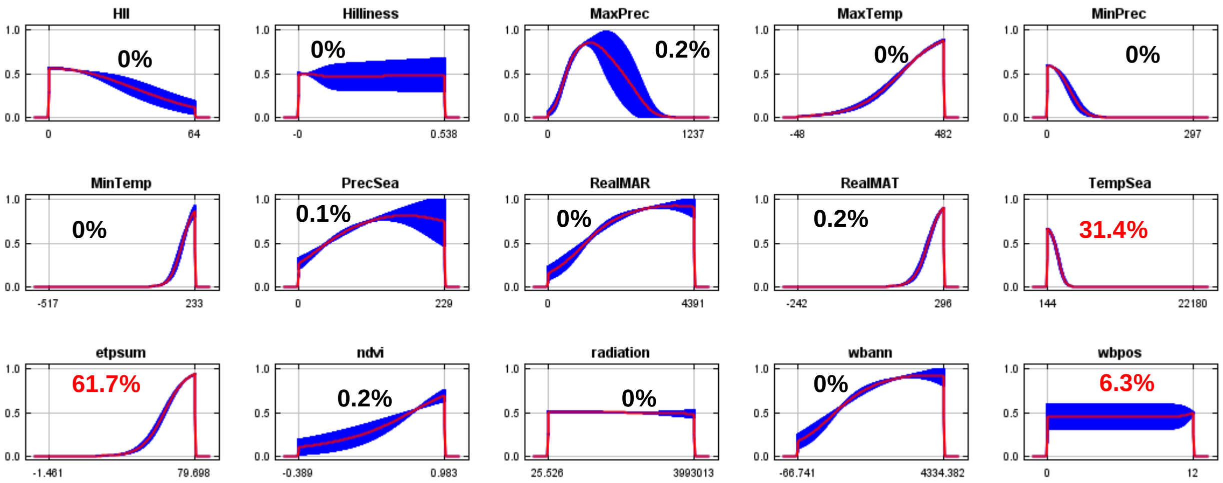

#68 – Greater red rock hare (*Pronolagus crassicaudatus*)
n = 7
Expert: Kai Collins, University of Pretoria
Expert evaluation: Poor
Data: Only modern
Envelope: Climatic and habitat
Dispersal distance: 2km/year (Similar ecology to *P.randensis*)
Status: UNMODELLABLE; **Included in final analysis:** X

Model evaluation metric	
AUC	0.99
Omission rate	0.00
Sensitivity	1.00
Specificity	0.98
Proportion correct	0.98
**Kappa**	**0.06**
True Skill Statistic	0.98

Summary: The Greater red rock hare's bioclimatic envelope is predicted to decrease by 65% with a ~3° mean latitudinal polewards shift and a mean decrease in elevation of ~20m driven by a decrease in maximum elevation. 95% of the permutation importance of the model was contributed to by human influence index (31.7%), temperature seasonality (29.3%) solar radiation (16.9%) and minimum precipitation (18.3%).

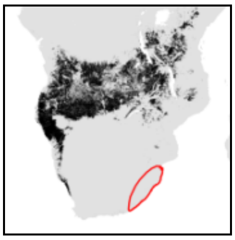
a) ~ 1930s (1900-1949)

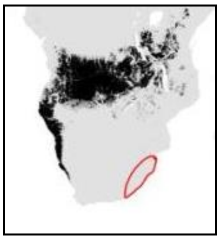
b) ~ 1980s (1950-2000)

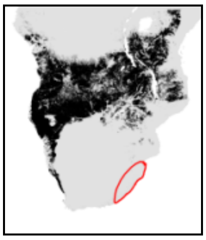
c) ~ 2020s (2010-2039)

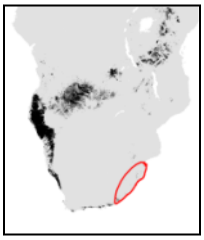
d) ~ 2050s (2040-2069)

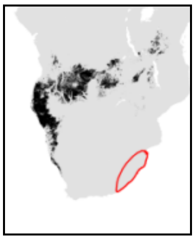
e) ~ 2080s (2070-2099)

Black = predicted suitable envelope

Red polygon = IUCN range (Matthee, C., *et al*. 2008)

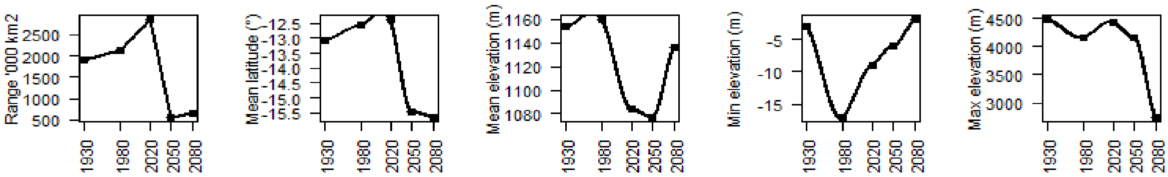

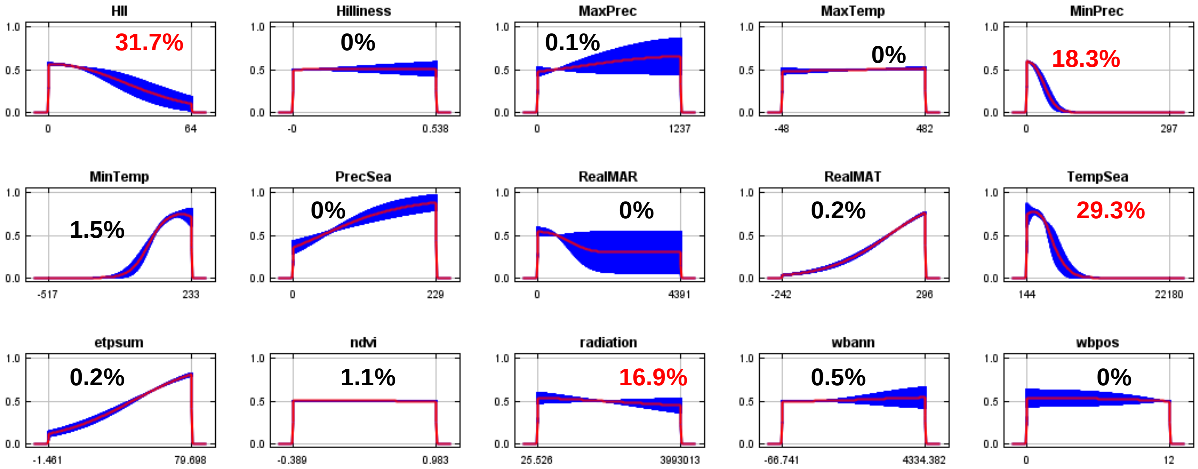

#69 – Jameson's red rock hare (*Pronolagus randensis*)
n = 27
Expert: Kai Collins, University of Pretoria
Expert evaluation: Poor
Data: Modern and historic
Envelope: Climatic and habitat
Dispersal distance: 2km/year (Expert)
Status: UNMODELLABLE; **Included in final analysis:** X

Model evaluation metric	
AUC	0.98
Omission rate	0.04
Sensitivity	0.96
Specificity	0.99
Proportion correct	0.99
**Kappa**	**0.55**
True Skill Statistic	0.96

Summary: The Jameson's red rock hare's bioclimatic envelope is predicted to decrease by 70% with a ~5° mean latitudinal polewards shift and a mean increase in elevation of ~325m driven by an increase in maximum and minimum elevation. 95% of the permutation importance of the model was contributed to by mean annual temperature (46.2%), maximum temperature (23.3%), minimum precipitation (16.3%), minimum temperature (7.0%) and temperature seasonality (1.4%).

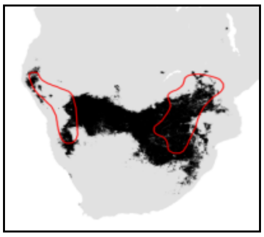
a) ~ 1930s (1900-1949)

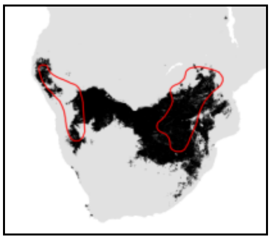
b) ~ 1980s (1950-2000)

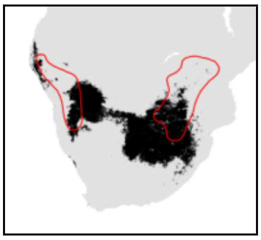
c) ~ 2020s (2010-2039)

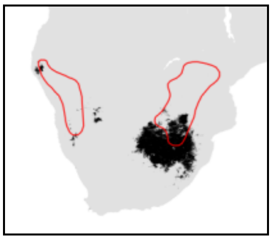
d) ~ 2050s (2040-2069)

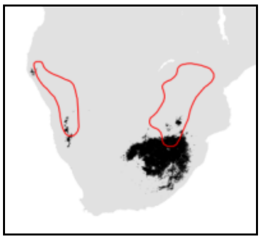
e) ~ 2080s (2070-2099)

Black = predicted suitable envelope

Red polygon = IUCN range (Matthee, C., *et al*. 2008)

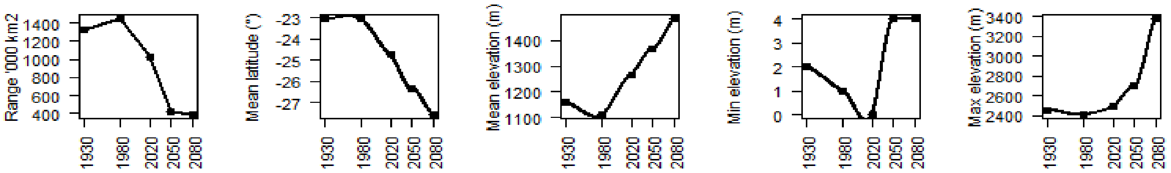

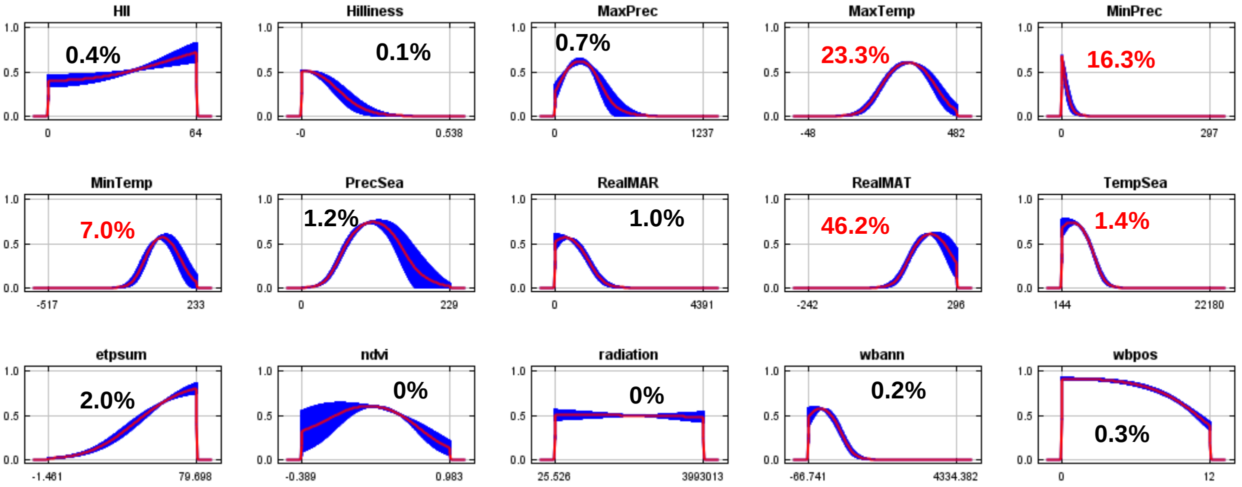

#70 – Smith's red rock hare (*Pronolagus rupestris*)
n = 9
Expert: Kai Collins, University of Pretoria
Expert evaluation: Poor
Data: Modern and historic
Envelope: Climatic and habitat
Dispersal distance: 2km/year (Similar ecology to *P.randensis*)
Status: UNMODELLABLE; **Included in final analysis:** X

Model evaluation metric	
AUC	0.99
Omission rate	0.00
Sensitivity	1.00
Specificity	0.98
Proportion correct	0.98
**Kappa**	**0.07**
True Skill Statistic	0.98

Summary: The Smith's red rock hare's bioclimatic envelope is predicted to decrease by 60% with a ~5° mean latitudinal polewards shift and a mean decrease in elevation of ~60m. 95% of the permutation importance of the model was contributed to by temperature seasonality (44.0%), minimum precipitation (18.5%), mean annual precipitation (18.3%), normalised difference vegetation index (6.3%), human influence index (5.2%) and minimum temperature (3.4%).

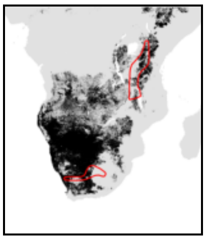
a) ~ 1930s (1900-1949)

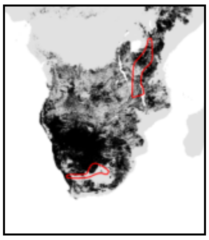
b) ~ 1980s (1950-2000)

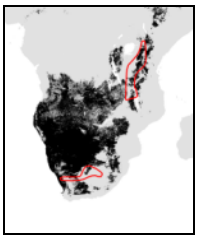
c) ~ 2020s (2010-2039)

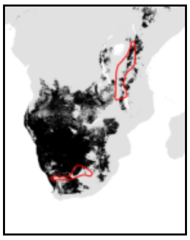
d) ~ 2050s (2040-2069)

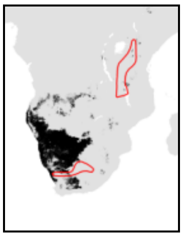
e) ~ 2080s (2070-2099)

Black = predicted suitable envelope

Red polygon = IUCN range (Smith, A.T. & Boyer, A.F. 2008)

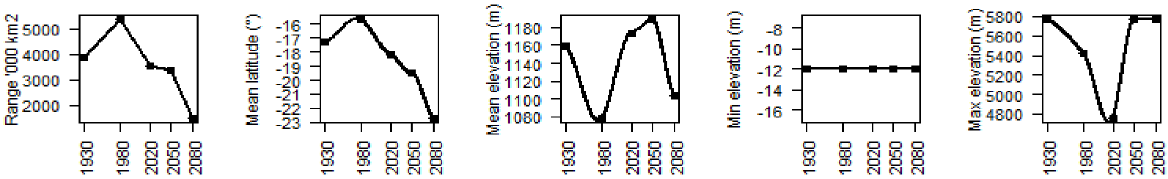

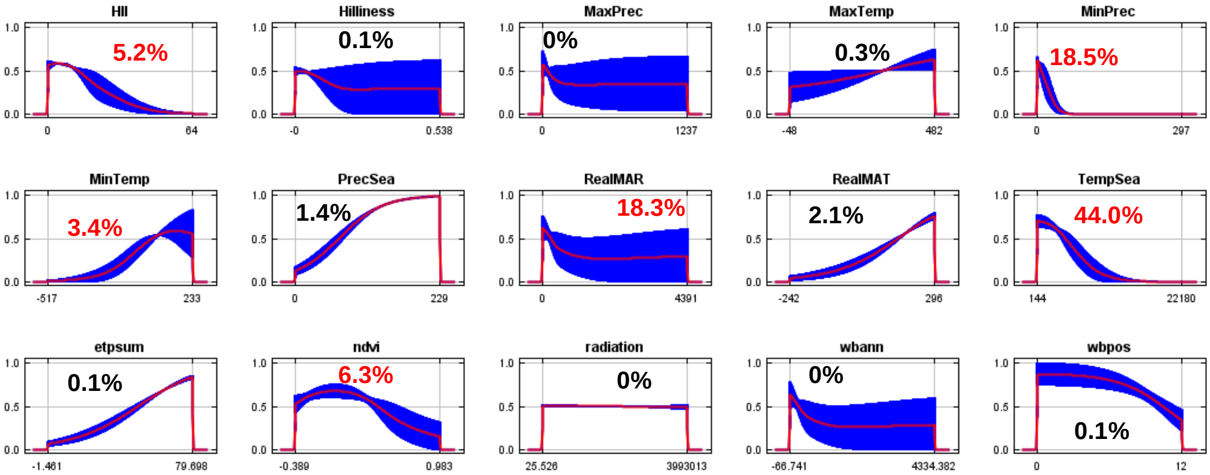

#71 – Hewitt's red rock hare (*Pronolagus saundersiae*)
n = 9
Expert: Kai Collins, University of Pretoria
Expert evaluation: Poor
Data: Modern and historic
Envelope: Climatic and habitat
Dispersal distance: 2km/year (Similar ecology to *P.randensis*)
Status: UNMODELLABLE; **Included in final analysis:** X

Model evaluation metric	
AUC	1.00
Omission rate	0.00
Sensitivity	1.00
Specificity	1.00
Proportion correct	1.00
**Kappa**	**1.00**
True Skill Statistic	1.00

Summary: The Hewitt's red rock hare's bioclimatic envelope is predicted to decrease by 100% with a ~1° mean latitudinal polewards shift and a mean increase in elevation of ~15m driven by an increase in minimum elevation. 95% of the permutation importance of the model was contributed to by temperature seasonality (28.7%), precipitation seasonality (24.3%), maximum temperature (18.0%), annual evapotranspiration (16.0%), human influence index (5.6%), minimum temperature (1.9%) and minimum precipitation (1.6%).

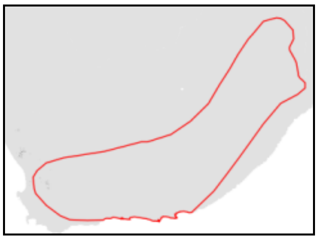
a) ~ 1930s (1900-1949)

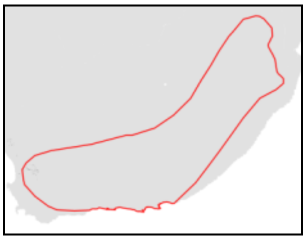
b) ~ 1980s (1950-2000)

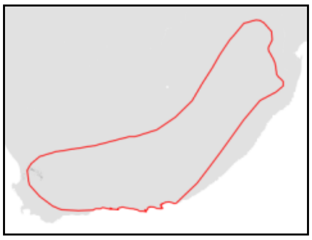
c) ~ 2020s (2010-2039)

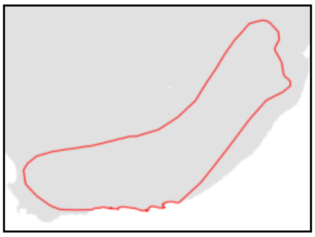
d) ~ 2050s (2040-2069)

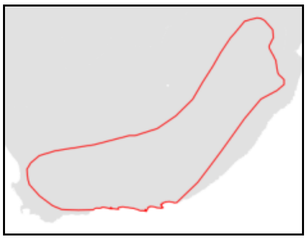
e) ~ 2080s (2070-2099)

Black = predicted suitable envelope

Red polygon = IUCN range (Smith, A.T. & Johnston, C.H. 2008)

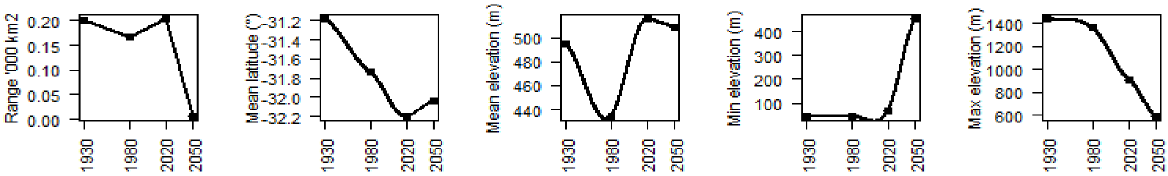

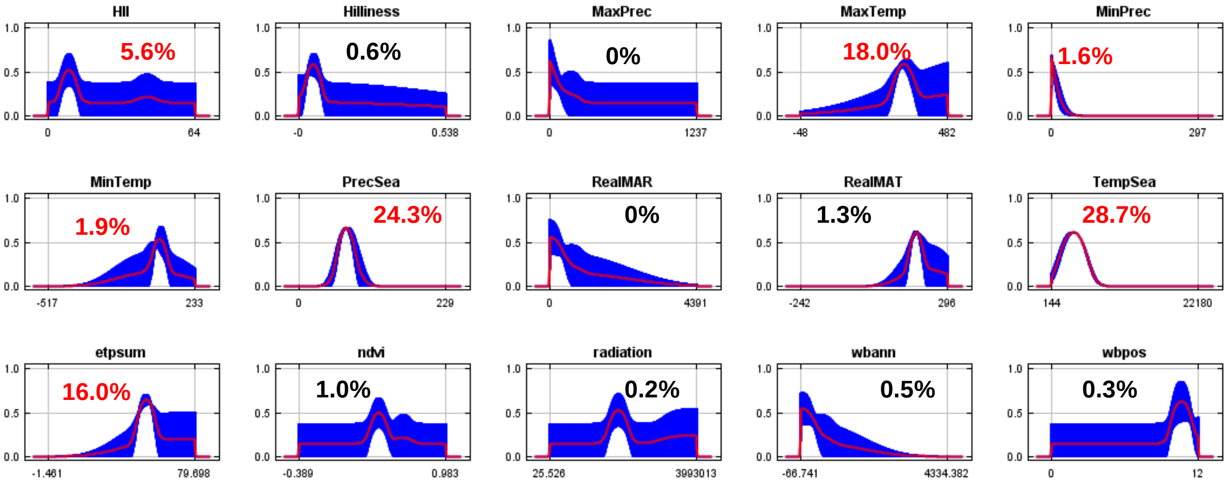

#72 – Volcano rabbit (*Romerolagus diazi*)
n = 31
Expert: Jose Antonio Martinez-Garcia, Universidad Autónoma Metropolitana, Mexico
Expert evaluation: Poor
Data: Only modern
Envelope: Climatic and habitat
Dispersal distance: 0.01km/year (Island species, range 0.01-0.01)
Status: UNMODELLABLE; **Included in final analysis:** X

Model evaluation metric	
AUC	0.95
Omission rate	0.10
Sensitivity	0.90
Specificity	0.99
Proportion correct	0.99
**Kappa**	**0.79**
True Skill Statistic	0.90

Summary: The Volcano rabbit's bioclimatic envelope is predicted to decrease by 100% with a ~0.2° mean latitudinal polewards shift and a mean increase in elevation of ~1500m driven by increases in minimum and maximum elevation. 95% of the permutation importance of the model was contributed to by temperature seasonality (75.2%), precipitation seasonality (16.2%) and minimum temperature (6.8%).

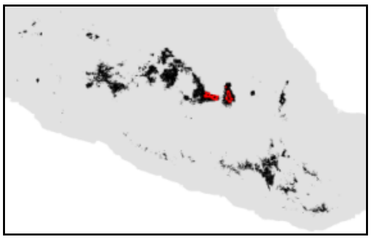
a) ~ 1930s (1900-1949)

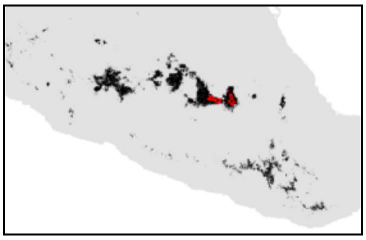
b) ~ 1980s (1950-2000)

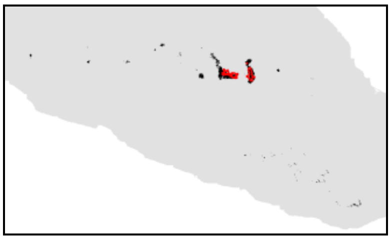
c) ~ 2020s (2010-2039)

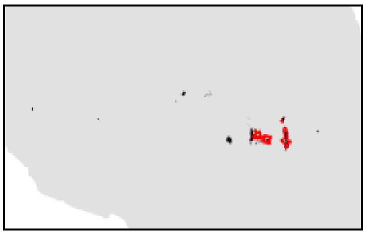
d) ~ 2050s (2040-2069)

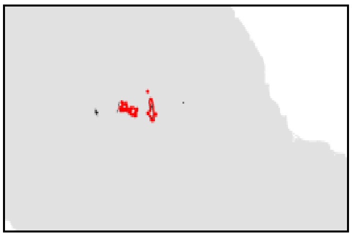
e) ~ 2080s (2070-2099)

Black = predicted suitable envelope

Red polygon = IUCN range (Malpica, F.J.,*et al*. 2008)

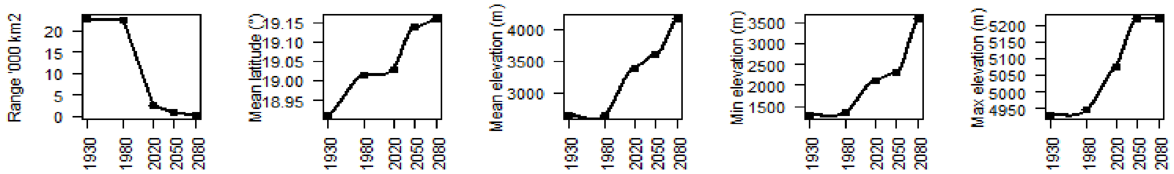

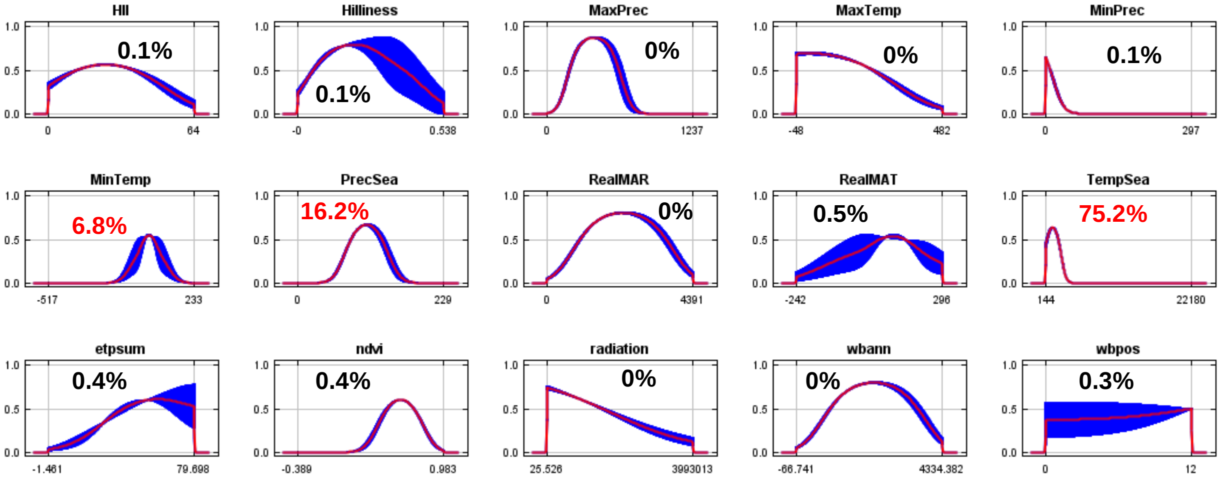

#73 – Swamp rabbit (*Sylvilagus aquaticus*)
n = 66
Expert: Robert Kissell, Memphis State University
Expert evaluation: Medium
Data: Modern and historic
Envelope: Climatic and habitat
Dispersal distance: 25km/year (Expert)
Status: MODELLABLE; **Included in final analysis:** √

Model evaluation metric	
AUC	0.95
Omission rate	0.09
Sensitivity	0.91
Specificity	0.99
Proportion correct	0.99
**Kappa**	**0.76**
True Skill Statistic	0.91

Summary: The Swamp rabbit's bioclimatic envelope is predicted to increase by 200% with a ~2° mean latitudinal polewards shift and a mean increase in elevation of ~60m driven by an increase in maximum elevation. 95% of the permutation importance of the model was contributed to by temperature seasonality (38.1%), mean annual temperature (36.7%), mean annual precipitation (12.7%), precipitation seasonality (4.7%) and minimum precipitation (3.6%).

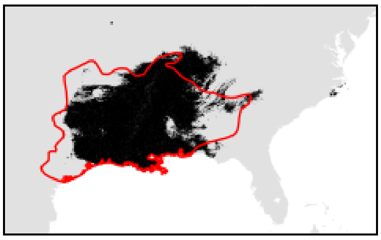

a) ~ 1930s (1900-1949)

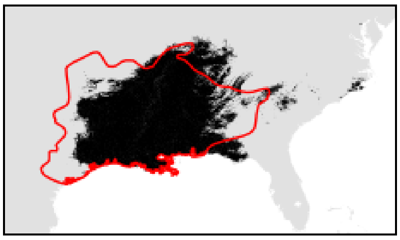

b) ~ 1980s (1950-2000)

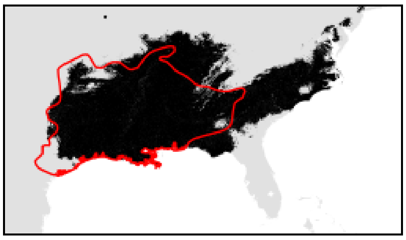

c) ~ 2020s (2010-2039)

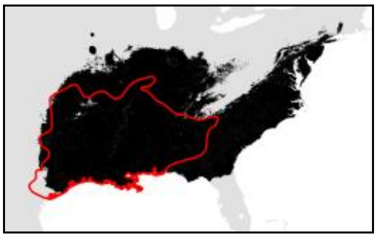

d) ~ 2050s (2040-2069)

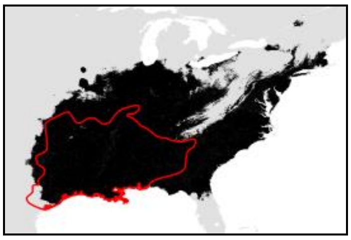

e) ~ 2080s (2070-2099)

Black = predicted suitable envelope

Red polygon = IUCN range (Smith, A.T. & Boyer, A.F. 2008)

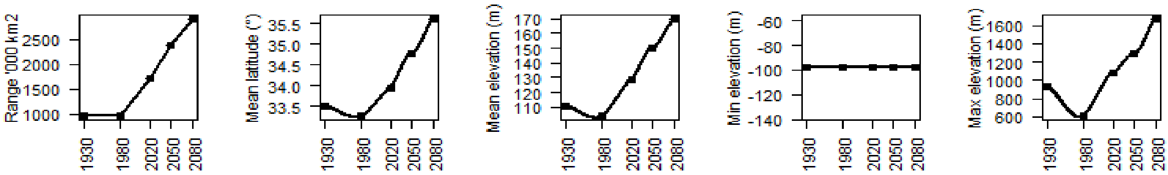

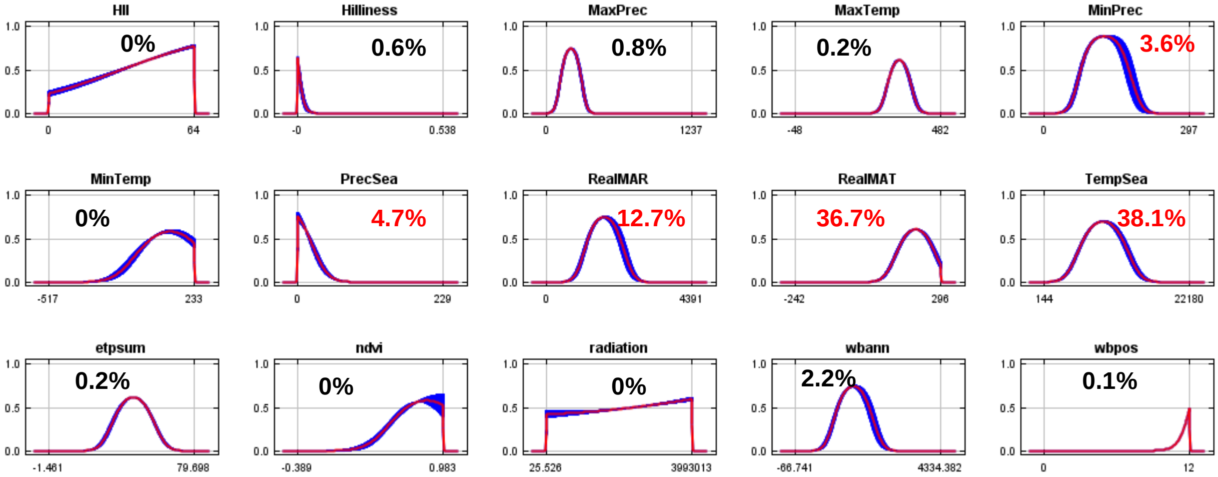

#74 – Desert cottontail (*Sylvilagus audubonii*)
n = 1040
Expert: Consuelo Lorenzo, Departamento Conservación de la Biodiversidad, Chiapas
Expert evaluation: Medium
Data: Modern and historic
Envelope: Climatic and habitat
Dispersal distance: 7.5km/year (Similar ecology to *S.palustris*)
Status: MODELLABLE; **Included in final analysis:** √

Model evaluation metric	
AUC	0.94
Omission rate	0.08
Sensitivity	0.92
Specificity	0.96
Proportion correct	0.96
**Kappa**	**0.78**
True Skill Statistic	0.88

Summary: The Desert cottontail's bioclimatic envelope is predicted to increase by 5% with a ~1° mean latitudinal polewards shift and a mean increase in elevation of ~30m driven by an increase in minimum elevation. 95% of the permutation importance of the model was contributed to by precipitation seasonality (36.6%), annual evapotranspiration (27.1%), mean annual temperature (13.6%), minimum temperature (9.1%), maximum temperature (3.0%), annual water balance (2.1%), minimum precipitation (1.9%) and human influence index (1.7%).

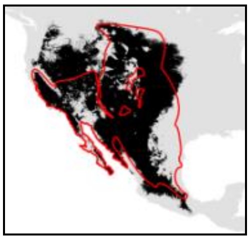

a) ~ 1930s (1900-1949)

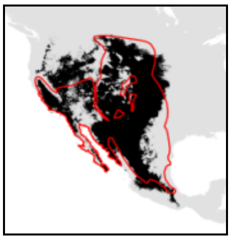

b) ~ 1980s (1950-2000)

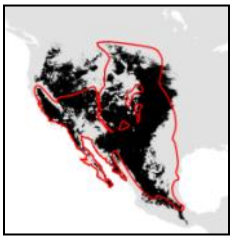

c) ~ 2020s (2010-2039)

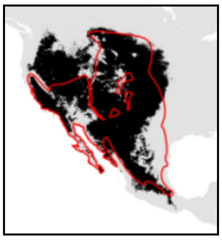

d) ~ 2050s (2040-2069)

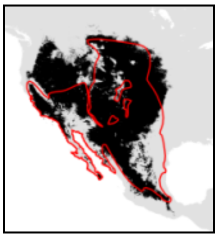

e) ~ 2080s (2070-2099)

Black = predicted suitable envelope

Red polygon = IUCN range (Romero Malpica, F.J. & Rangel Cordero, H. 2008)

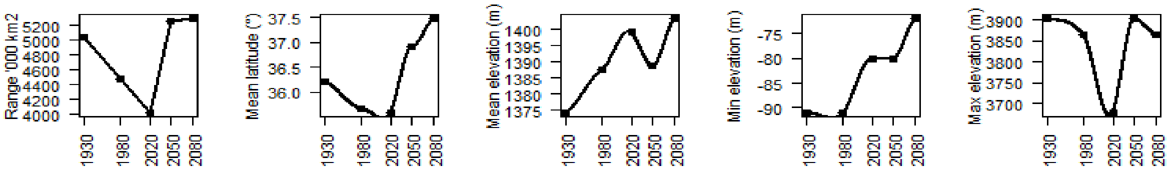

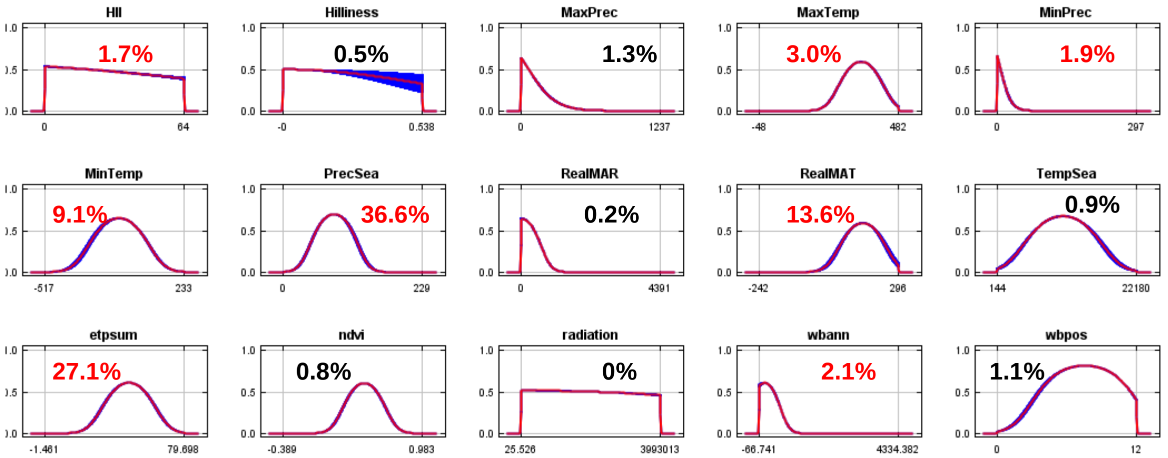

#75 – Brush rabbit (*Sylvilagus bachmani*)
n = 263
Expert: Consuelo Lorenzo, Departamento Conservación de la Biodiversidad, Chiapas
Expert evaluation: Medium
Data: Modern and historic
Envelope: Climatic and habitat
Dispersal distance: 3km/year (Similar ecology to *S.transitionalis*)
Status: MODELLABLE; **Included in final analysis:** √

Model evaluation metric	
AUC	0.96
Omission rate	0.08
Sensitivity	0.92
Specificity	0.99
Proportion correct	0.99
**Kappa**	**0.89**
True Skill Statistic	0.91

Summary: The Brush rabbit's bioclimatic envelope is predicted to decrease by 15% with a ~3° mean latitudinal polewards shift and a mean decrease in elevation of ~10m. 95% of the permutation importance of the model was contributed to by mean annual temperature (31.5%), minimum precipitation (24.5%), precipitation seasonality (14.4%), mean annual precipitation (7.1%), annual water balance (6.4%), minimum temperature (5.1%), normalised difference vegetation index (4.1%) and temperature seasonality (2.7%).

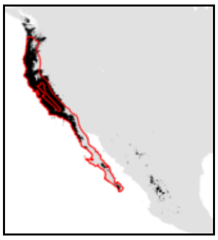
a) ~ 1930s (1900-1949)

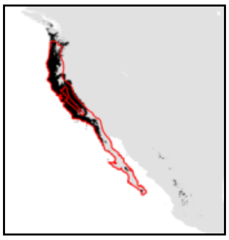
b) ~ 1980s (1950-2000)

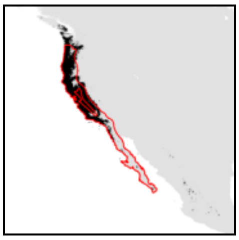
c) ~ 2020s (2010-2039)

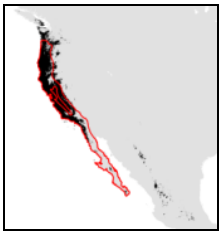
d) ~ 2050s (2040-2069)

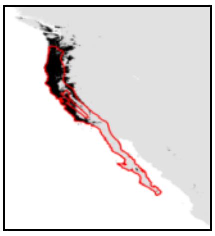
e) ~ 2080s (2070-2099)

Black = predicted suitable envelope

Red polygon = IUCN range (Romero Malpica, F.J.*et al*. 2008)

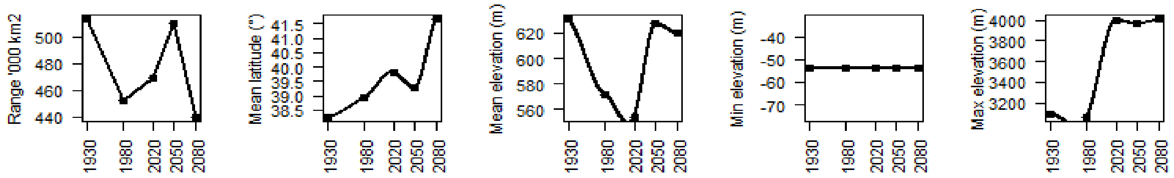

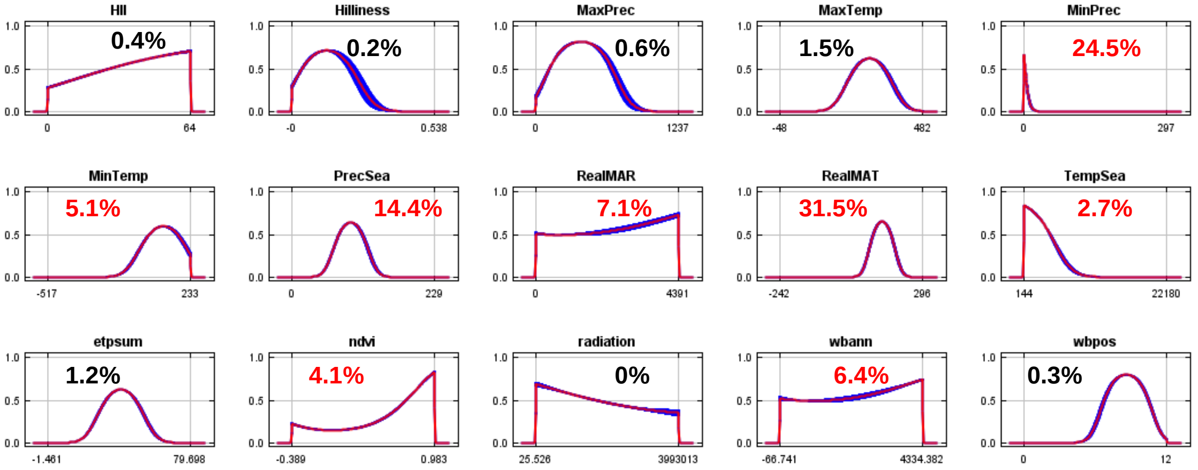

#76 – Forest rabbit (*Sylvilagus brasiliensis*)
n = 181
Expert: Jorge Salazar-Bravo, Texas Tech University
Expert evaluation: Medium
Data: Modern and historic
Envelope: Climatic and habitat
Dispersal distance: 7.5km/year (Similar ecology to *S.palustris*)
Status: MODELLABLE; **Included in final analysis:** √

Model evaluation metric	
AUC	0.95
Omission rate	0.10
Sensitivity	0.90
Specificity	0.99
Proportion correct	0.99
**Kappa**	**0.73**
True Skill Statistic	0.89

Summary: The Forest rabbit's bioclimatic envelope is predicted to decrease by 50% with a ~6° mean latitudinal polewards shift and a mean increase in elevation of ~210m driven by an increase in minimum elevation. 95% of the permutation importance of the model was contributed to by annual evapotranspiration (71.4%), temperature seasonality (11.6%), normalised difference vegetation index (4.9%) and minimum temperature (4.6%).

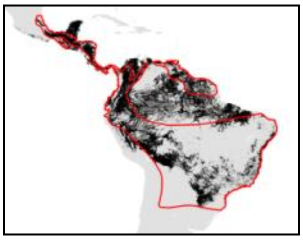
a) ~ 1930s (1900-1949)

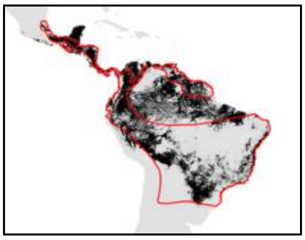
b) ~ 1980s (1950-2000)

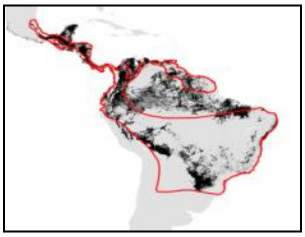
c) ~ 2020s (2010-2039)

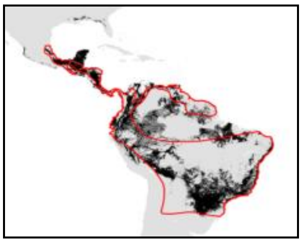
d) ~ 2050s (2040-2069)

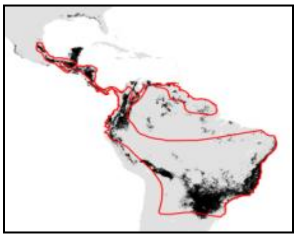
e) ~ 2080s (2070-2099)

Black = predicted suitable envelope

Red polygon = IUCN range (Romero Malpica, F.J. & Rangel Cordero, H. 2008)

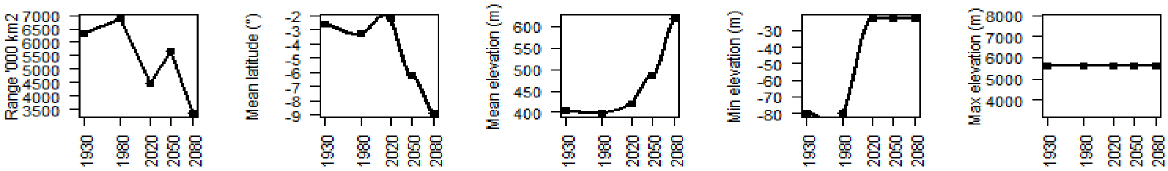

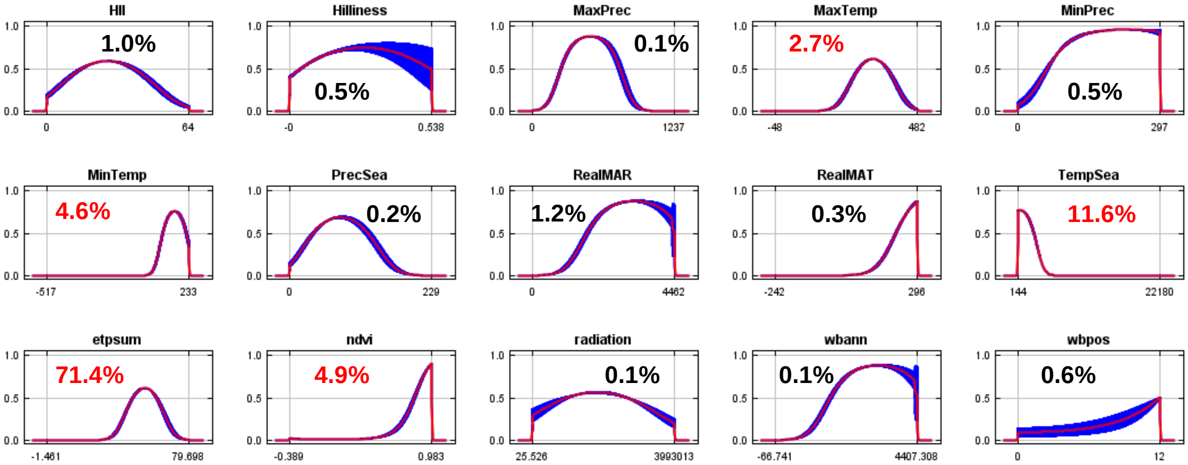

#77 – Manzano mountain cottontail (*Sylvilagus cognatus*)
n = 7
Expert: Jennifer Frey, New Mexico State University
Expert evaluation: Medium
Data: Modern and historic
Envelope: Climatic and habitat
Dispersal distance: 0.01km/year (Similar ecology to *R.diazi*)
Status: MODELLABLE; **Included in final analysis:** √

Model evaluation metric	
AUC	0.99
Omission rate	0.00
Sensitivity	1.00
Specificity	0.99
Proportion correct	0.99
**Kappa**	**0.52**
True Skill Statistic	0.99

Summary: The Manzano mountain cottontail's bioclimatic envelope is predicted to decrease by 90% with a ~2° mean latitudinal polewards shift and a mean increase in elevation of ~230m driven by an increase in minimum elevation. 95% of the permutation importance of the model was contributed to by annual water balance (40.8%), minimum temperature (21.7%), precipitation seasonality (11.3%), mean annual temperature (8.2%), temperature seasonality (6.1%), minimum precipitation (4.3%) and mean annual precipitation (2.6%).

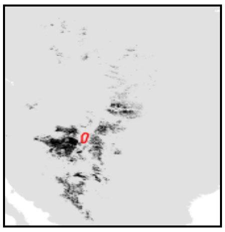

a) ~ 1930s (1900-1949)

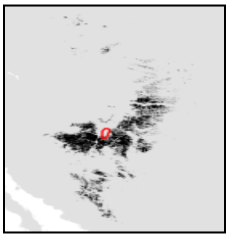

b) ~ 1980s (1950-2000)

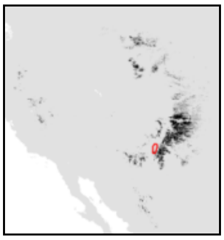

c) ~ 2020s (2010-2039)

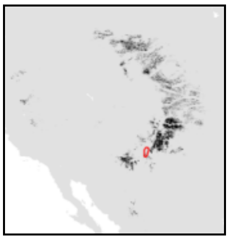

d) ~ 2050s (2040-2069)

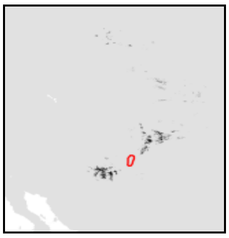

e) ~ 2080s (2070-2099)

Black = predicted suitable envelope

Red polygon = IUCN range (Smith, A.T. & Boyer, A. F. 2008)

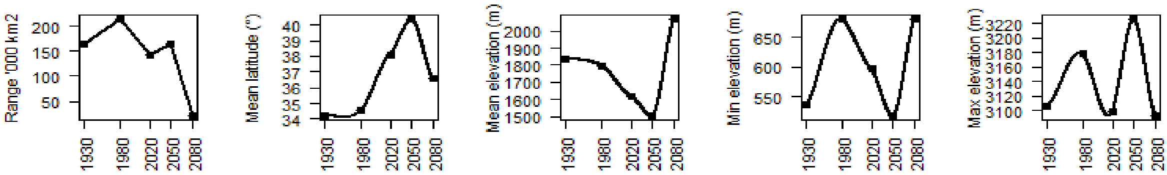

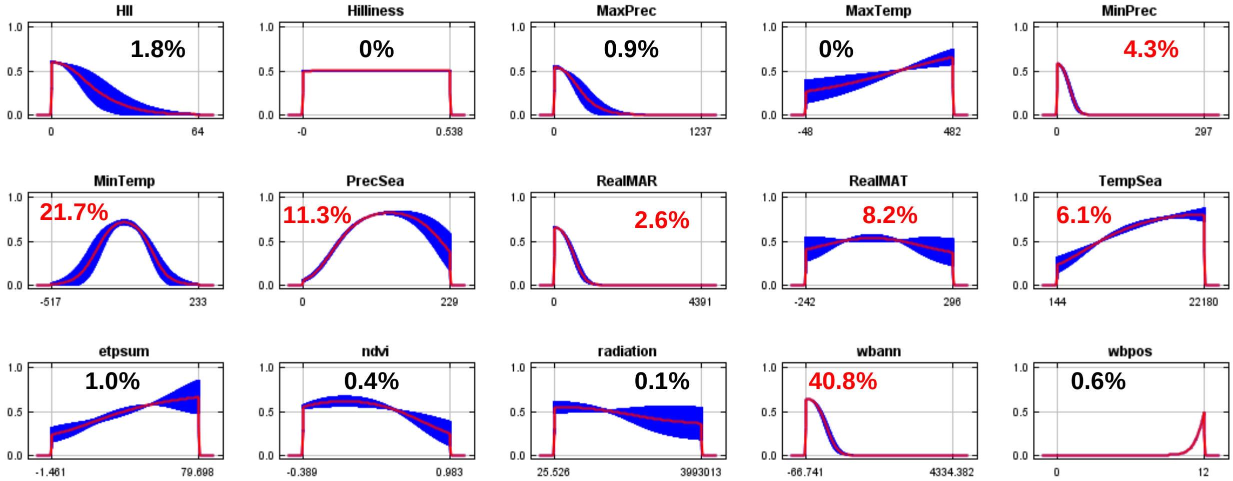

#78 – Mexican cottontail (*Sylvilagus cunicularius*)
n = 76
Expert: Jorge Vazquez, Laboratorio de Ecología del Comportamiento, UAT-UNAM
Expert evaluation: Medium
Data: Only modern
Envelope: Climatic and habitat
Dispersal distance: 7.5km/year (Similar ecology to *S.palustris*)
Status: MODELLABLE; **Included in final analysis:** √

**Model evaluation metric**	
AUC	0.95
Omission rate	0.10
Sensitivity	0.90
Specificity	0.99
Proportion correct	0.99
**Kappa**	**0.73**
True Skill Statistic	0.89

Summary: The Mexican cottontail's bioclimatic envelope is predicted to decrease by 15% with a ~0.5^{o} mean latitudinal polewards shift and a mean increase in elevation of ~200m driven by an increase in minimum elevation. 95% of the permutation importance of the model was contributed to by temperature seasonality (48.3%), precipitation seasonality (42.8%), normalised difference vegetation index (3.5%) and minimum temperature (2.2%).

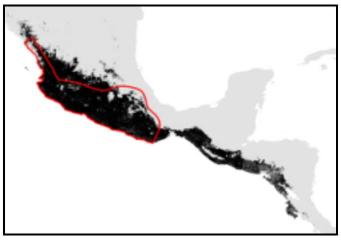
a) ~ 1930s (1900-1949)

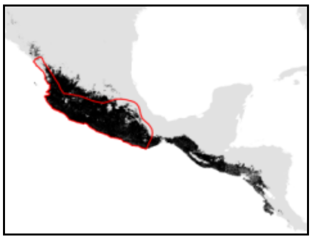
b) ~ 1980s (1950-2000)

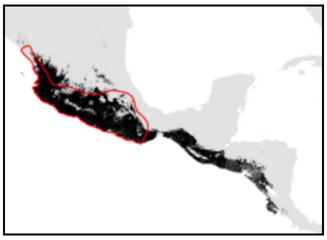
c) ~ 2020s (2010-2039)

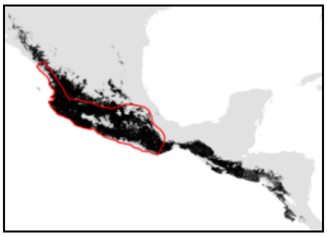
d) ~ 2050s (2040-2069)

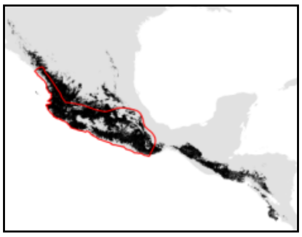
e) ~ 2080s (2070-2099)

Black = predicted suitable envelope

Red polygon = IUCN range (Romero Malpica, F.J. & Rangel Cordero, H. 2008)

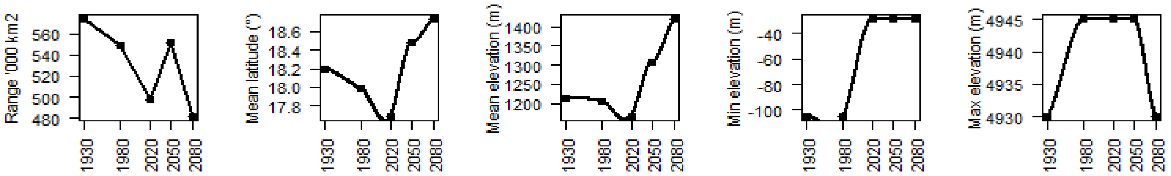

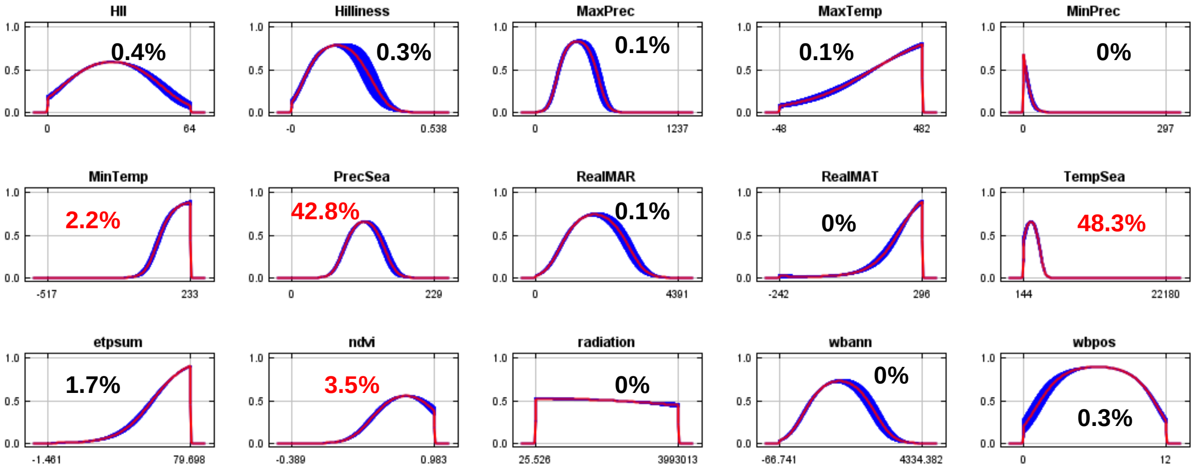

#79 – Dice's cottontail (*Sylvilagus dicei*)
n = 8
Expert: Jan Schipper, Arizona State University
Expert evaluation: Poor
Data: Only modern
Envelope: Climatic and habitat
Dispersal distance: 0.01km/year (Similar ecology to *S.cognatus*)
Status: UNMODELLABLE; **Included in final analysis:** X

Model evaluation metric	
AUC	0.99
Omission rate	0.00
Sensitivity	1.00
Specificity	0.99
Proportion correct	0.99
**Kappa**	**0.73**
True Skill Statistic	0.99

Summary: The Dice's cottontail's bioclimatic envelope is predicted to decrease by 50% with a ~1° mean latitudinal shift towards the Equator and a mean decrease in elevation of ~50m driven by a decrease in maximum elevation. 95% of the permutation importance of the model was contributed to by temperature seasonality (93.7%) and annual water balance (2.6%).

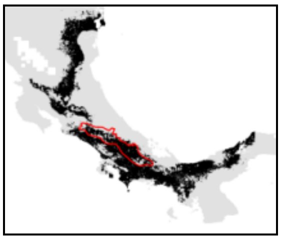

a) ~ 1930s (1900-1949)

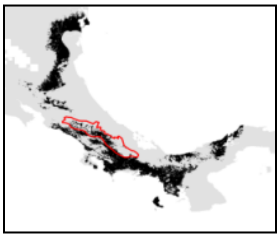

b) ~ 1980s (1950-2000)

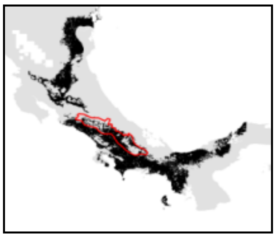

c) ~ 2020s (2010-2039)

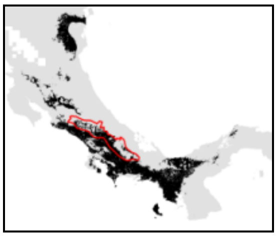

d) ~ 2050s (2040-2069)

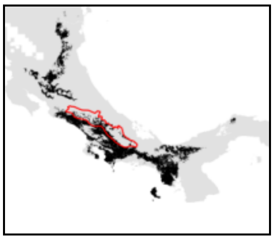

e) ~ 2080s (2070-2099)

Black = predicted suitable envelope

Red polygon = IUCN range (Smith, A.T. & Boyer, A.F. 2008)

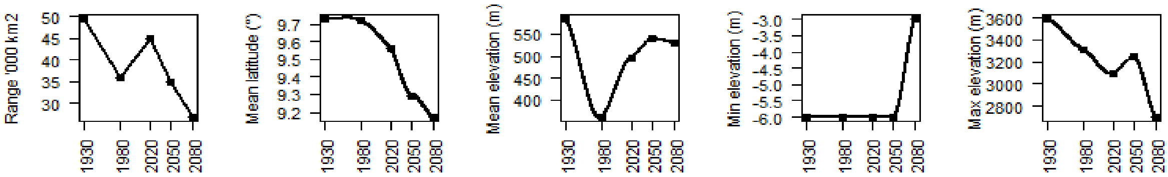

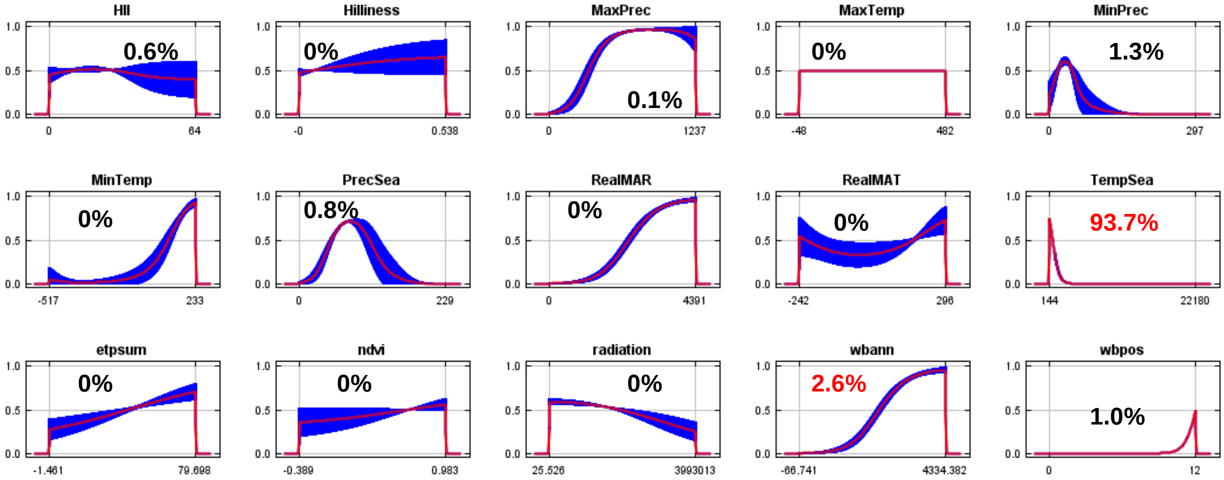

#80 – Eastern cottontail (*Sylvilagus floridanus*)
n = 1104
Expert: Jorge Vazquez, Laboratorio de Ecología del Comportamiento, UAT-UNAM
Expert evaluation: Medium
Data: Modern and historic
Envelope: Climatic and habitat
Dispersal distance: 7.5km/year (Similar ecology to *S.palustris*)
Status: MODELLABLE; **Included in final analysis:** √

Model evaluation metric	
AUC	0.92
Omission rate	0.09
Sensitivity	0.91
Specificity	0.93
Proportion correct	0.93
**Kappa**	**0.69**
True Skill Statistic	0.84

Summary: The Eastern cottontail's bioclimatic envelope is predicted to increase by 20% with a ~2° mean latitudinal polewards shift and a mean increase in elevation of ~90m driven by an increase in minimum and maximum elevation. 95% of the permutation importance of the model was contributed to by annual evapotranspiration (29.5%), precipitation seasonality (26.2%), minimum temperature (11.5%), temperature seasonality (8.5%), minimum precipitation (8.1%), maximum temperature (3.3%), normalised difference vegetation index (2.9%), maximum precipitation (2.8%) and annual water balance (2.3%).

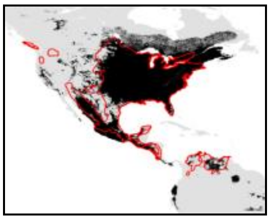
a) ~ 1930s (1900-1949)

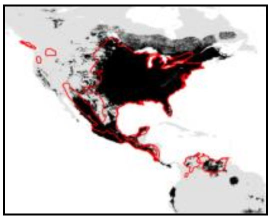
b) ~ 1980s (1950-2000)

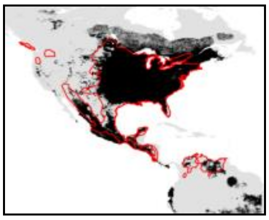
c) ~ 2020s (2010-2039)

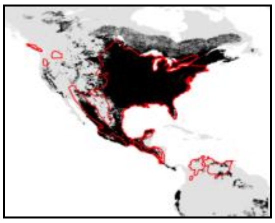
d) ~ 2050s (2040-2069)

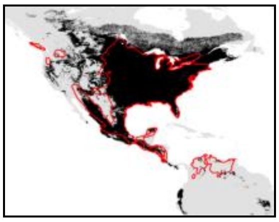
e) ~ 2080s (2070-2099)

Black = predicted suitable envelope

Red polygon = IUCN range (Romero Malpica, F.J. & Rangel Cordero, H. 2008)

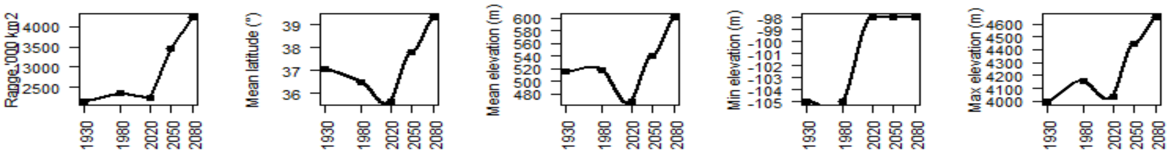

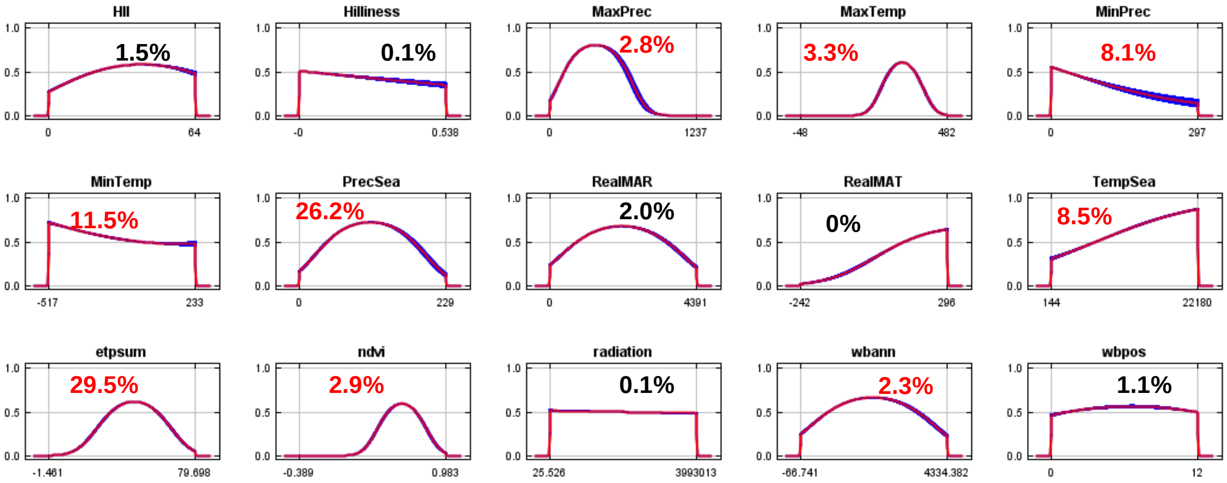

#81 – Tres Marias cottontail (*Sylvilagus graysoni*)
n = 6
Expert: Consuelo Lorenzo, Departamento Conservación de la Biodiversidad, Chiapas
Expert evaluation: Good
Data: Modern and historic
Envelope: Climatic and habitat
Dispersal distance: 0.01km/year (Island species, range 0.01-0.01)
Status: MODELLABLE; **Included in final analysis:** √

Model evaluation metric	
AUC	1.00
Omission rate	0.00
Sensitivity	1.00
Specificity	1.00
Proportion correct	1.00
**Kappa**	**1.00**
True Skill Statistic	1.00

Summary: The Tres Marias cottontail's bioclimatic envelope is predicted to decrease by 20% with a no latitudinal polewards shift and a mean increase in elevation of ~25m driven by an increase in minimum elevation. 95% of the permutation importance of the model was contributed to by human influence index (56.0%), minimum precipitation (32.4%), precipitation seasonality (5.2%) and annual evapotranspiration (3.2%).

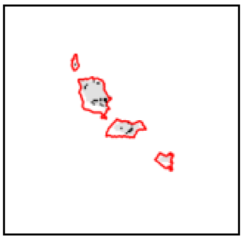
a) ~ 1930s (1900-1949)

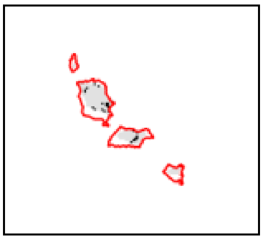
b) ~ 1980s (1950-2000)

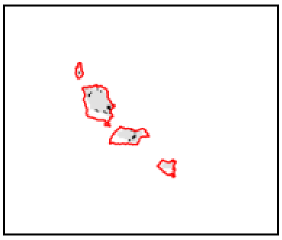
c) ~ 2020s (2010-2039)

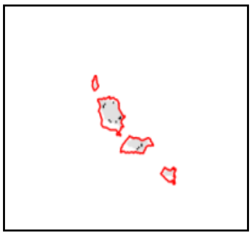
d) ~ 2050s (2040-2069)

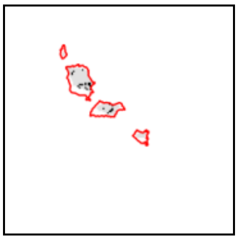
e) ~ 2080s (2070-2099)

Black = predicted suitable envelope

Red polygon = IUCN range (Romero Malpica, F.J. & Rangel Cordero, H. 2008)

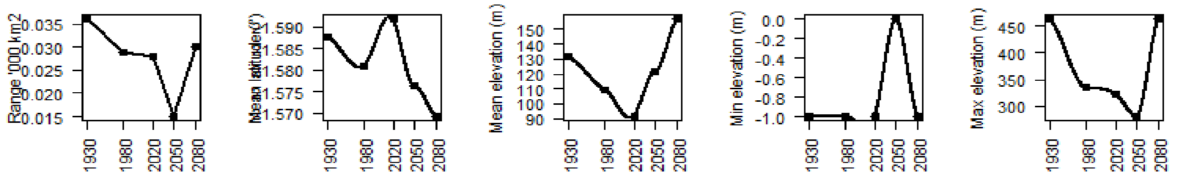

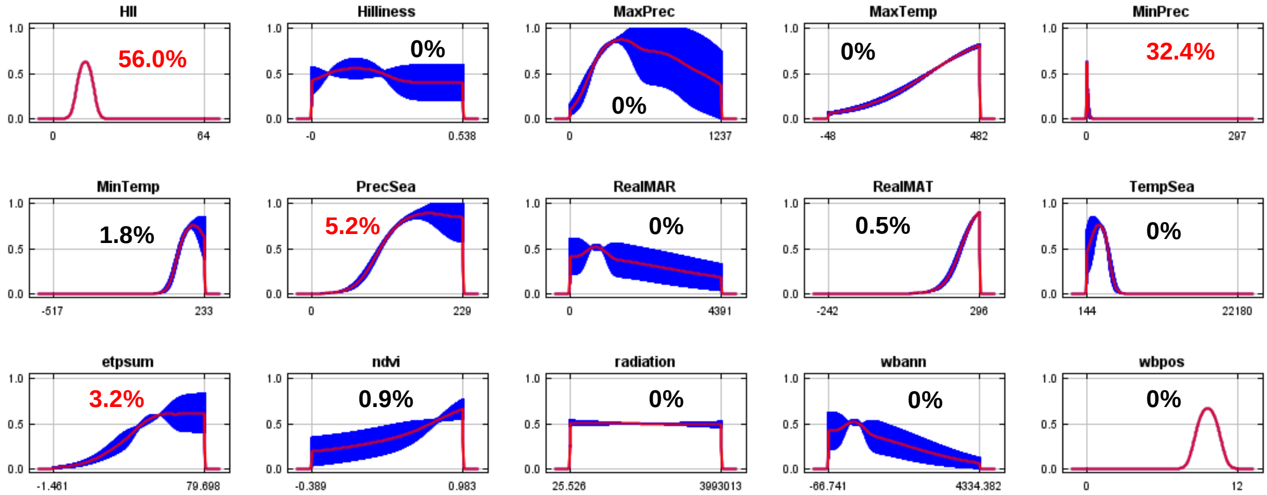

#82 – Omilteme cottontail (*Sylvilagus insonus*)
n = 3
Expert: Alejandro Velazquez, UNAM-Canada
Expert evaluation: Good
Data: Only modern
Envelope: Climatic and habitat
Dispersal distance: 0.01km/year (Similar ecology to *S.dicei*)
Status: MODELLABLE; **Included in final analysis:** √

Model evaluation metric	
AUC	1.00
Omission rate	0.00
Sensitivity	1.00
Specificity	1.00
Proportion correct	1.00
**Kappa**	**1.00**
True Skill Statistic	1.00

Summary: The Omilteme cottontail's bioclimatic envelope is predicted to decrease by 80% with a no latitudinal polewards shift and a mean increase in elevation of ~120m driven by an increase in maximum elevation. 95% of the permutation importance of the model was contributed to by normalised difference vegetation index (78.5%), precipitation seasonality (11.3%) and temperature seasonality (5.3%).

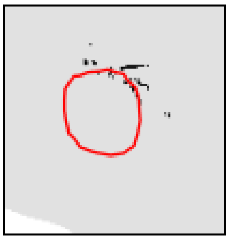
a) ~ 1930s (1900-1949)

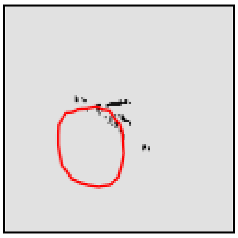
b) ~ 1980s (1950-2000)

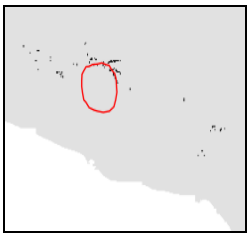
c) ~ 2020s (2010-2039)

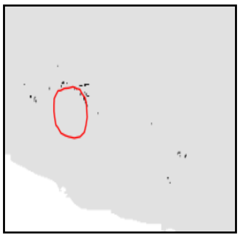
d) ~ 2050s (2040-2069)

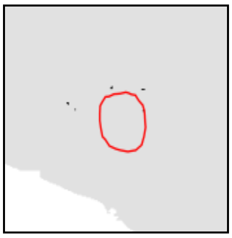
e) ~ 2080s (2070-2099)

Black = predicted suitable envelope

Red polygon = IUCN range (Romero Malpica, F.J. & Rangel Cordero, H. 2008)

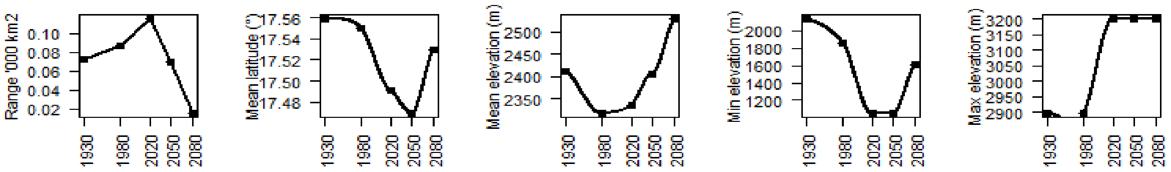

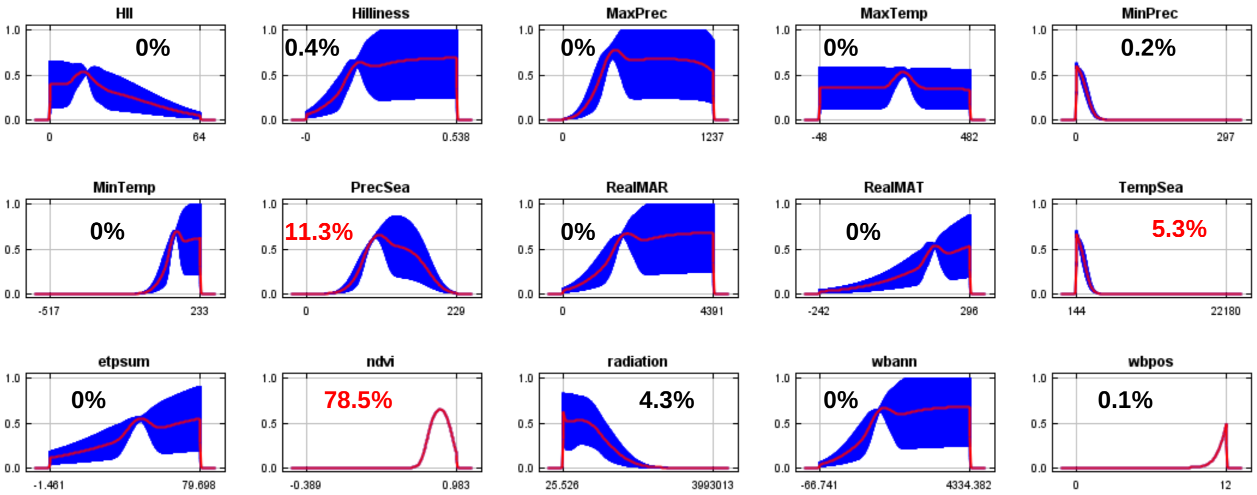

AUC	1.00
Omission rate	0.00
Sensitivity	1.00
Specificity	1.00
Proportion correct	1.00
**Kappa**	**1.00**
True Skill Statistic	1.00

#83 – San Jose brush rabbit (*Sylvilagus mansuetus*)
n = 9
Expert: Tamara Rioja Pardela, Universidad de Ciencias y Artes de Chiapas, Mexico
Expert evaluation: Good
Data: Only modern
Envelope: Climatic and habitat
Dispersal distance: 0.01km/year (Island species, range 0.01-0.01)
Status: MODELLABLE; **Included in final analysis:** √

Summary: The San Jose brush rabbit's bioclimatic envelope is predicted to decrease by 25% with a no latitudinal polewards shift and a mean increase in elevation of ~30m driven by an increase in minimum elevation. 95% of the permutation importance of the model was contributed to by temperature seasonality (36.7%), human influence index (34.0%), minimum precipitation (21.8%) and precipitation seasonality (3.2%).

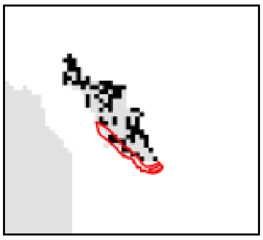
a) ~ 1930s (1900-1949)

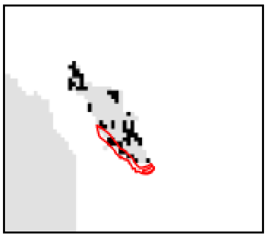
b) ~ 1980s (1950-2000)

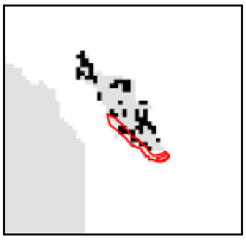
c) ~ 2020s (2010-2039)

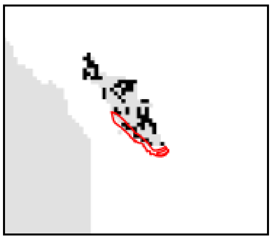
d) ~ 2050s (2040-2069)

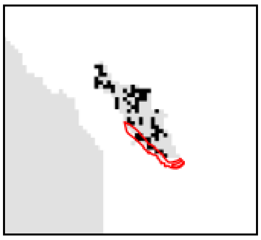
e) ~ 2080s (2070-2099)

Black = predicted suitable envelope

Red polygon = IUCN range (Lorenzo, C. & Álvarez-Castañeda, S. 2011)

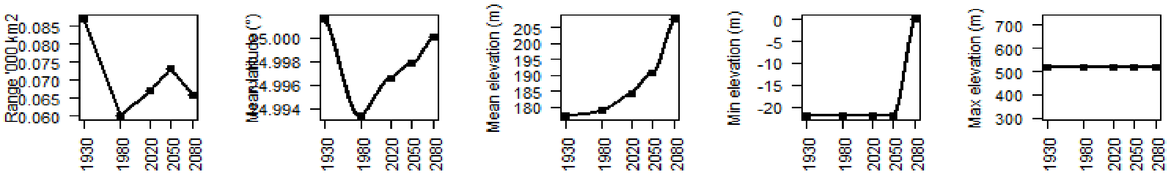

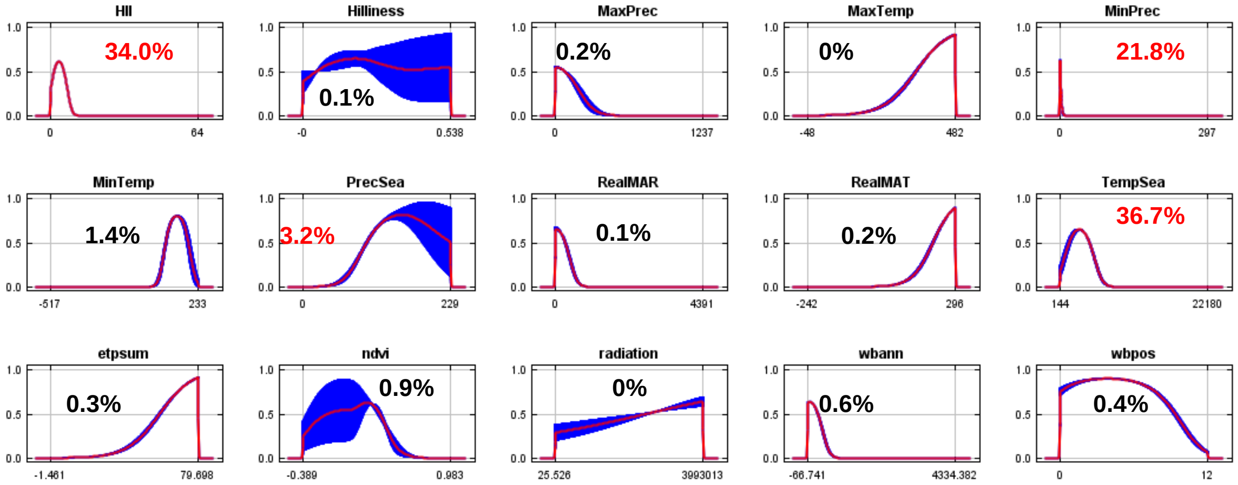

#84 – Mountain cottontail (*Sylvilagus nuttallii*)
n = 290
Expert: Jennifer Frey, New Mexico State University
Expert evaluation: Medium
Data: Modern and historic
Envelope: Climatic and habitat
Dispersal distance: 7.5km/year (Similar ecology to *S.palustris*)
Status: MODELLABLE; **Included in final analysis:** √

AUC	0.95
Omission rate	0.09
Sensitivity	0.91
Specificity	0.99
Proportion correct	0.99
**Kappa**	**0.78**
True Skill Statistic	0.90

Summary: The Mountain cottontail's bioclimatic envelope is predicted to decrease by 20% with a ~1° mean latitudinal polewards shift and a mean increase in elevation of ~40m driven by an increase in minimum elevation. 95% of the permutation importance of the model was contributed to by mean annual temperature (64.0%), maximum temperature (26.9%), temperature seasonality (2.8%) and minimum precipitation (1.4%).

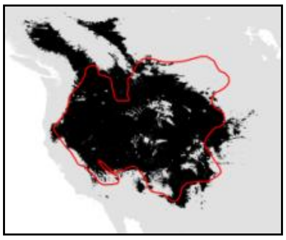
a) ~ 1930s (1900-1949)

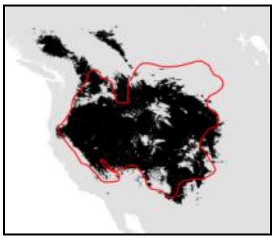
b) ~ 1980s (1950-2000)

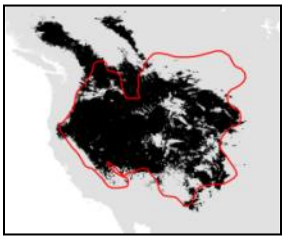
c) ~ 2020s (2010-2039)

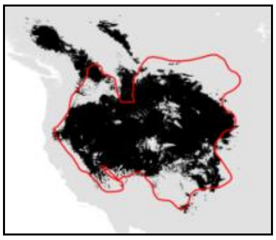
d) ~ 2050s (2040-2069)

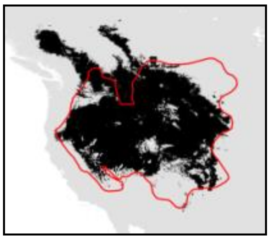
e) ~ 2080s (2070-2099)

Black = predicted suitable envelope

Red polygon = IUCN range (Smith, A.T. & Boyer, A.F. 2008)

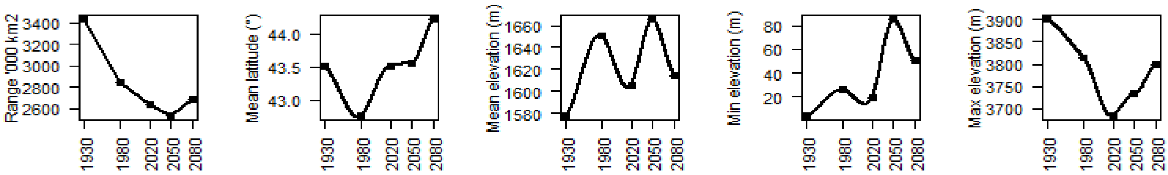

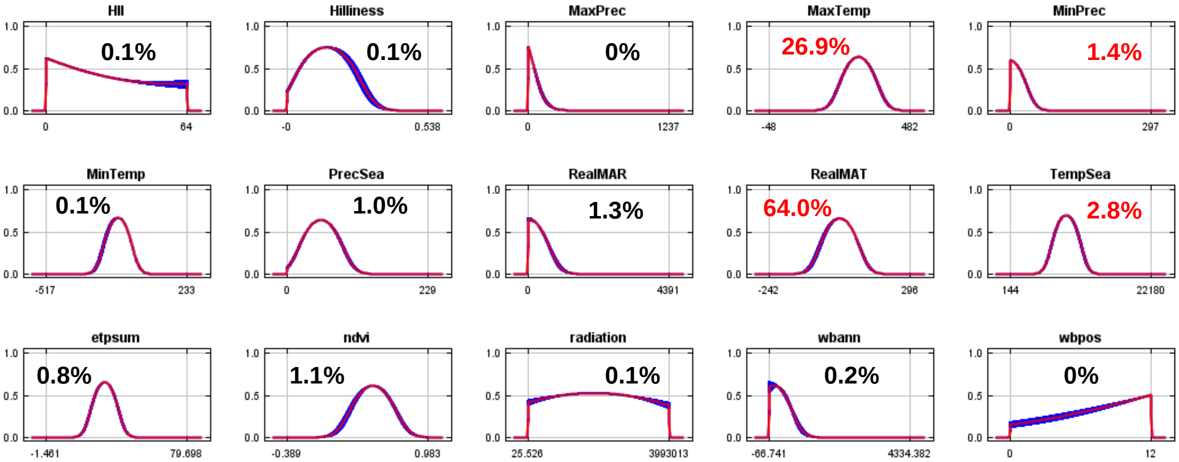

#85 – Appalachian cottontail (*Sylvilagus obscurus*)
n = 39
Expert: Michael Barbour, Alabama Natural Heritage Program
Expert evaluation: Medium
Data: Modern and historic
Envelope: Climatic only
Dispersal distance: 0.01km/year (Similar ecology to *S.dicei*)
Status: MODELLABLE; **Included in final analysis:** √

AUC	0.97
Omission rate	0.05
Sensitivity	0.95
Specificity	0.99
Proportion correct	0.99
**Kappa**	**0.73**
True Skill Statistic	0.95

Summary: The Appalachian cottontail's bioclimatic envelope is predicted to decrease by 10% with a ~3° mean latitudinal polewards shift and a mean increase in elevation of ~70m. 95% of the permutation importance of the model was contributed to by annual evapotranspiration (42.0%), minimum temperature (24.2%), minimum precipitation (18.8%) and precipitation seasonality (10.0%).

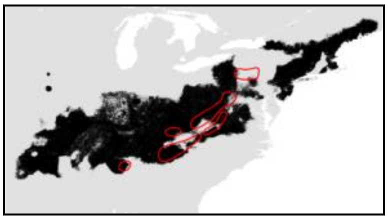
a) ~ 1930s (1900-1949)

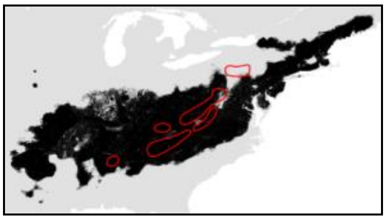
b) ~ 1980s (1950-2000)

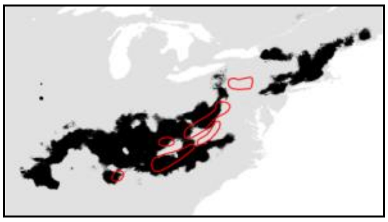
c) ~ 2020s (2010-2039)

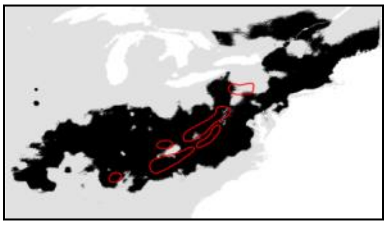
d) ~ 2050s (2040-2069)

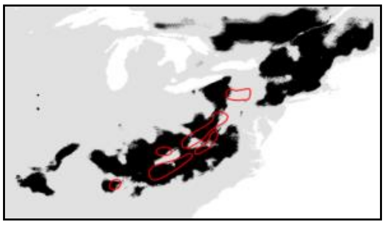
e) ~ 2080s (2070-2099)

Black = predicted suitable envelope

Red polygon = IUCN range (Barry, R. & Lazell, J. 2008)

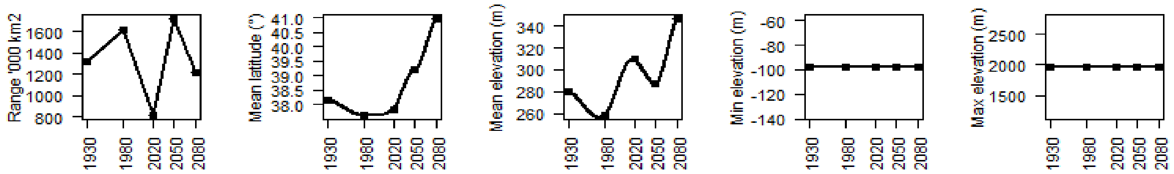

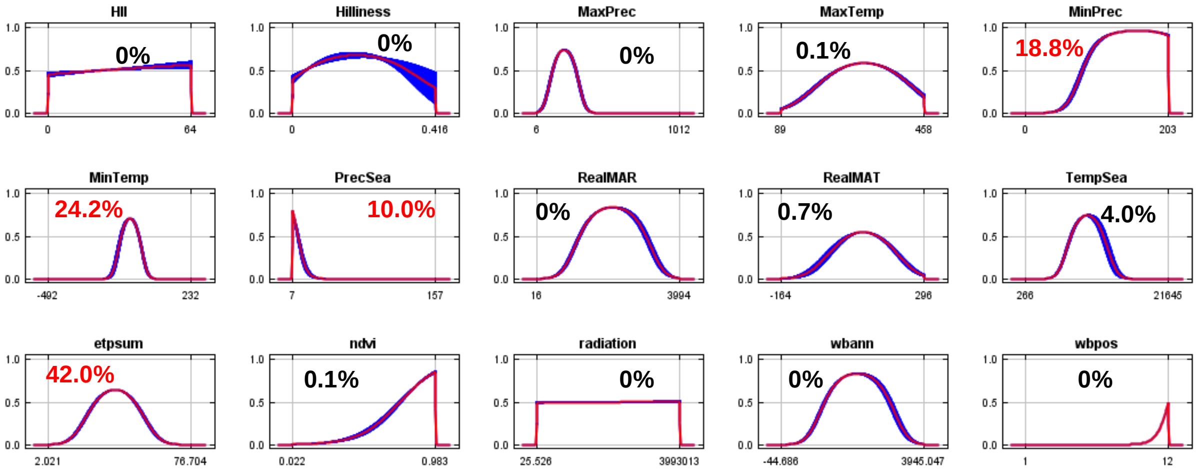

#86 – Marsh rabbit (*Sylvilagus palustris*)
n = 25
Expert: Bob McCleery, University of Florida
Expert evaluation: Good
Data: Only modern
Envelope: Climatic and habitat
Dispersal distance: 7.5km/year (Expert)
Status: MODELLABLE; **Included in final analysis:** √

AUC	0.99
Omission rate	0.00
Sensitivity	1.00
Specificity	0.99
Proportion correct	0.99
**Kappa**	**0.75**
True Skill Statistic	0.99

Summary: The Marsh rabbit's bioclimatic envelope is predicted to increase by 90% with a ~1° mean latitudinal polewards shift and a mean increase in elevation of ~2m driven by an increase in maximum elevation. 95% of the permutation importance of the model was contributed to by surface roughness index (73.1%), minimum precipitation (18.3%) and precipitation seasonality (4.9%).

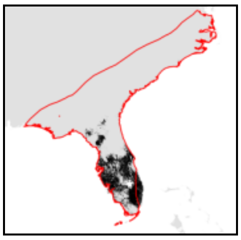

a) ~ 1930s (1900-1949)

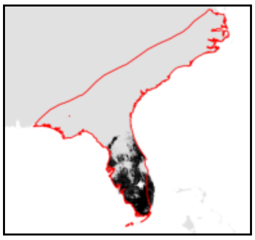

b) ~ 1980s (1950-2000)

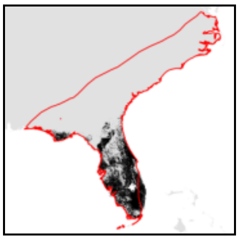

c) ~ 2020s (2010-2039)

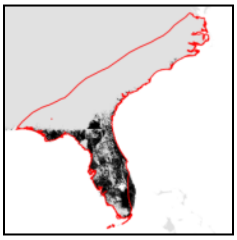

d) ~ 2050s (2040-2069)

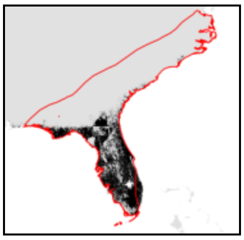

e) ~ 2080s (2070-2099)

Black = predicted suitable envelope

Red polygon = IUCN range (Faulhaber, C.A. & Smith, A.T. 2008)

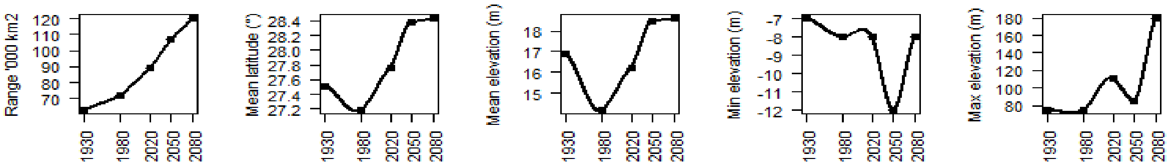

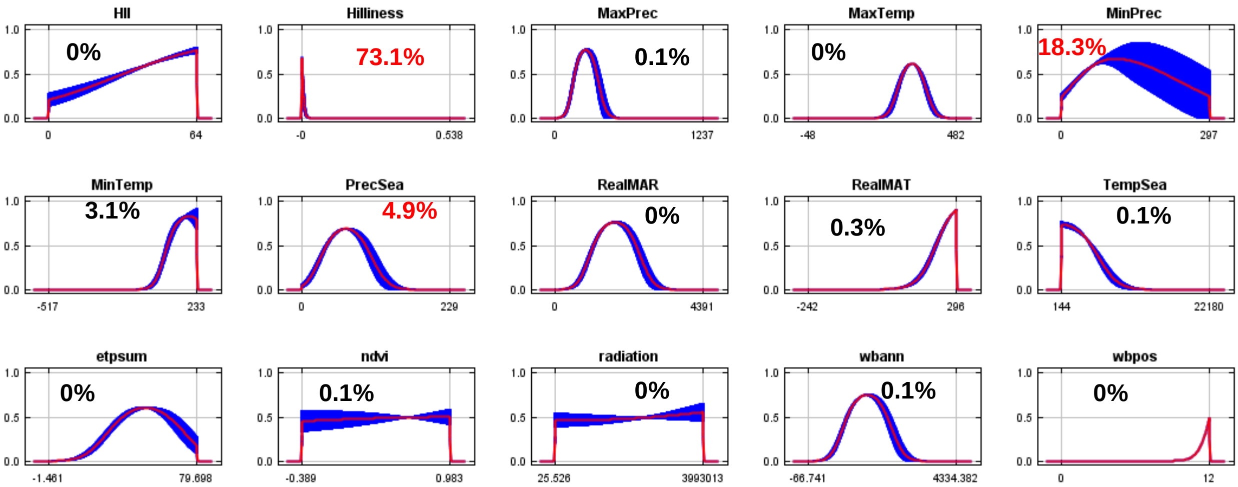

#87 – Robust cottontail (*Sylvilagus robustus*)
n = 9
Expert: Dana Lee, Oklahoma State University
Expert evaluation: Poor
Data: Modern and historic
Envelope: Climatic and habitat
Dispersal distance: 0.01km/year (Similar ecology to *S.dicei*)
Status: UNMODELLABLE; **Included in final analysis:** X

AUC	0.94
Omission rate	0.11
Sensitivity	0.89
Specificity	0.99
Proportion correct	0.99
**Kappa**	**0.27**
True Skill Statistic	0.88

Summary: The Robust cottontail's bioclimatic envelope is predicted to decrease by 90% with a ~4° mean latitudinal polewards shift and a mean increase in elevation of ~480m driven by an increase in minimum elevation. 95% of the permutation importance of the model was contributed to by precipitation seasonality (68.5%), minimum precipitation (8.2%), temperature seasonality (7.1%), minimum temperature (4.5%), mean annual precipitation (3.6%), annual water balance (2.6%) and number of months with a positive water balance (1.6%).

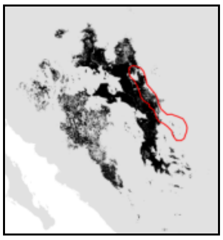

a) ~ 1930s (1900-1949)

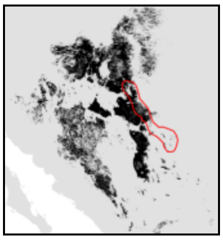

b) ~ 1980s (1950-2000)

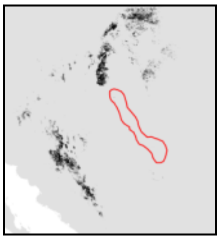

c) ~ 2020s (2010-2039)

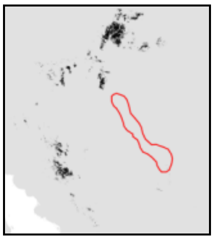

d) ~ 2050s (2040-2069)

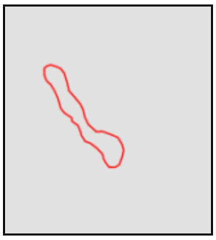

e) ~ 2080s (2070-2099)

Black = predicted suitable envelope

Red polygon = IUCN range (Ruedas, L. & Smith, A.T. 2008)

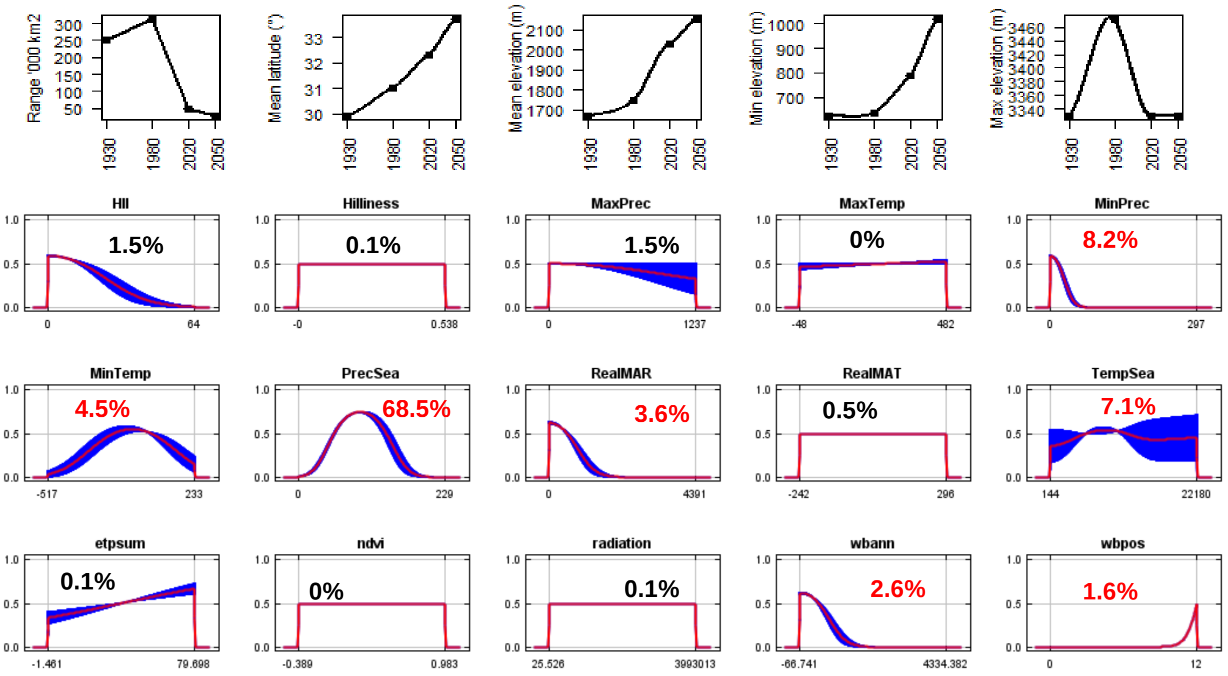

**#88 – New England cottontail** (*Sylvilagus transitionalis*)	
AUC	0.99
Omission rate	0.00
Sensitivity	1.00
Specificity	0.99
Proportion correct	0.99
**Kappa**	**0.68**
True Skill Statistic	0.99

#88 – New England cottontail (*Sylvilagus transitionalis*)
n = 18
Expert: John Litvaitis, University of New Hampshire
Expert evaluation: Medium
Data: Modern and historic
Envelope: Climatic and habitat
Dispersal distance: 3km/year (Expert)
Status: MODELLABLE; **Included in final analysis:** √

Summary: The New England cottontail's bioclimatic envelope is predicted to increase by 110% with a ~1° mean latitudinal polewards shift and a mean increase in elevation of ~70m driven by an increase in maximum elevation. 95% of the permutation importance of the model was contributed to by annual evapotranspiration (23.5%), temperature seasonality (22.2%), minimum temperature (17.4%), normalised difference vegetation index (16.0%), mean annual temperature (8.6%), maximum temperature (5.9%) and minimum precipitation (5.9%).

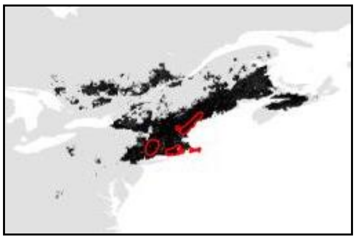

a) ~ 1930s (1900-1949)

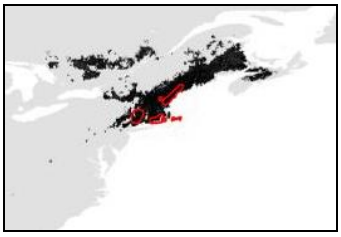

b) ~ 1980s (1950-2000)

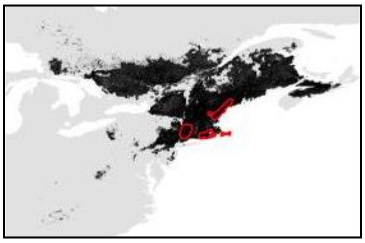

c) ~ 2020s (2010-2039)

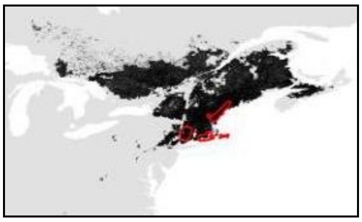

d) ~ 2050s (2040-2069)

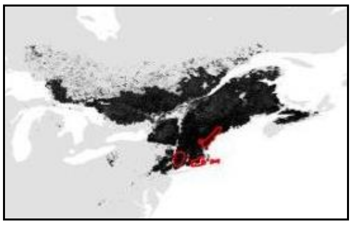

e) ~ 2080s (2070-2099)

Black = predicted suitable envelope

Red polygon = IUCN range (Barry, R., *et al*. 2008)

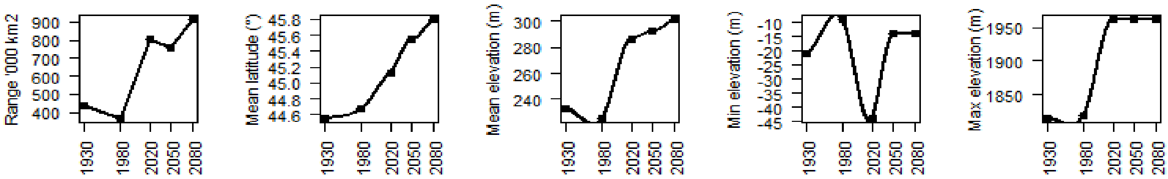

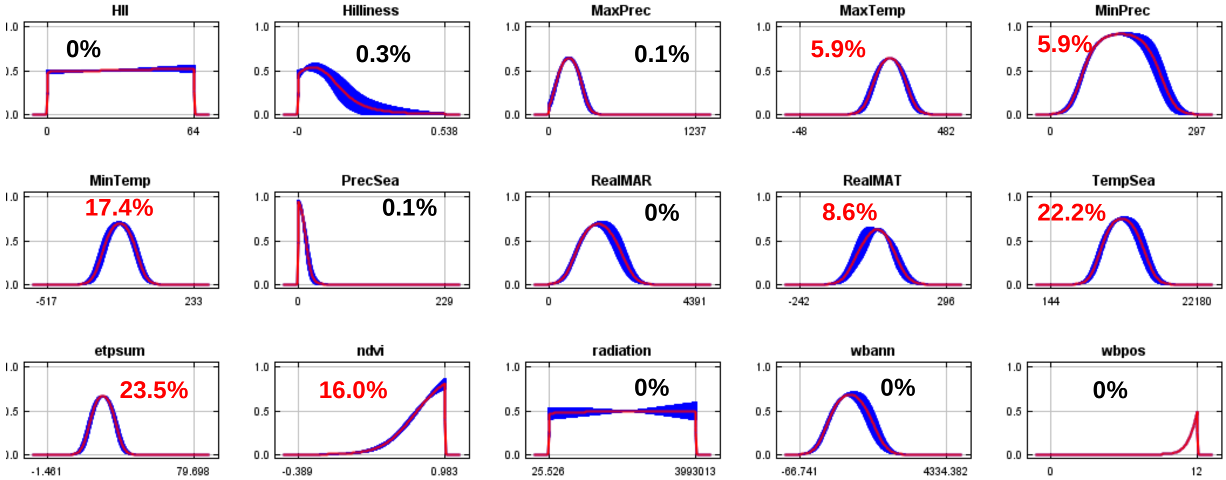

**#89 – Venezuelan lowland rabbit** (*Sylvilagus varynaensis*)	

#89 – Venezuelan lowland rabbit (*Sylvilagus varynaensis*)
n = 6
Expert: Daniel Lew, Venezuelan Institute of Scientific Research, Ecology Centre, Biodiversity Unit
Expert evaluation: Poor
Data: Only modern
Envelope: Climatic and habitat
Dispersal distance: 3km/year (Similar ecology to *S.transitionalis*)
Status: UNMODELLABLE; **Included in final analysis:** X

AUC	0.99
Omission rate	0.00
Sensitivity	1.00
Specificity	0.99
Proportion correct	0.99
**Kappa**	**0.92**
True Skill Statistic	0.99

Summary: The Venezuelan lowland rabbit's bioclimatic envelope is predicted to decrease by 100% with a ~1.5° mean latitudinal polewards shift and a mean increase in elevation of ~275m driven by an increase in minimum elevation. 95% of the permutation importance of the model was contributed to by temperature seasonality (97.7%).

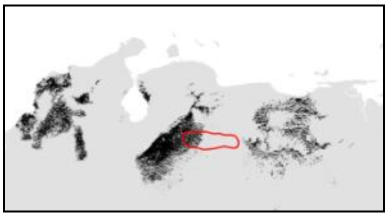
a) ~ 1930s (1900-1949)

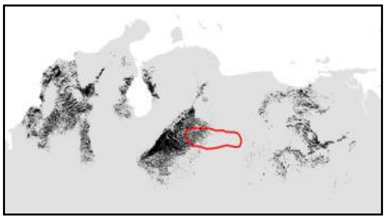
b) ~ 1980s (1950-2000)

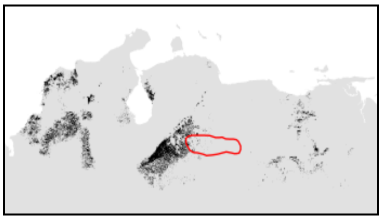
c) ~ 2020s (2010-2039)

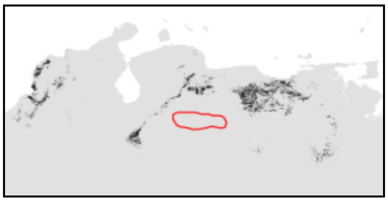

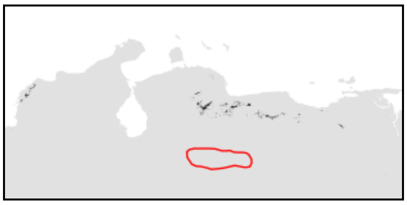

Black = predicted suitable envelope

Red polygon = IUCN range (Durant, P. & Guevara, M.A. 2008)

d) ~ 2050s (2040-2069)

e) ~ 2080s (2070-2099)

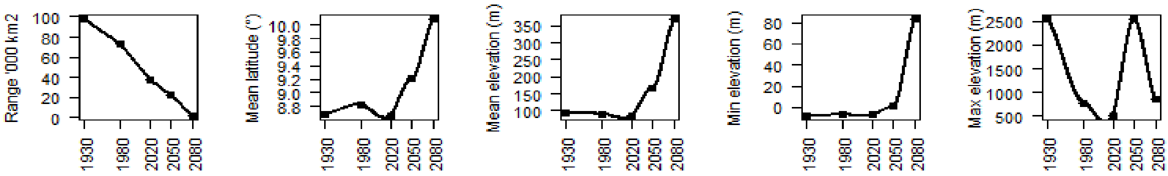

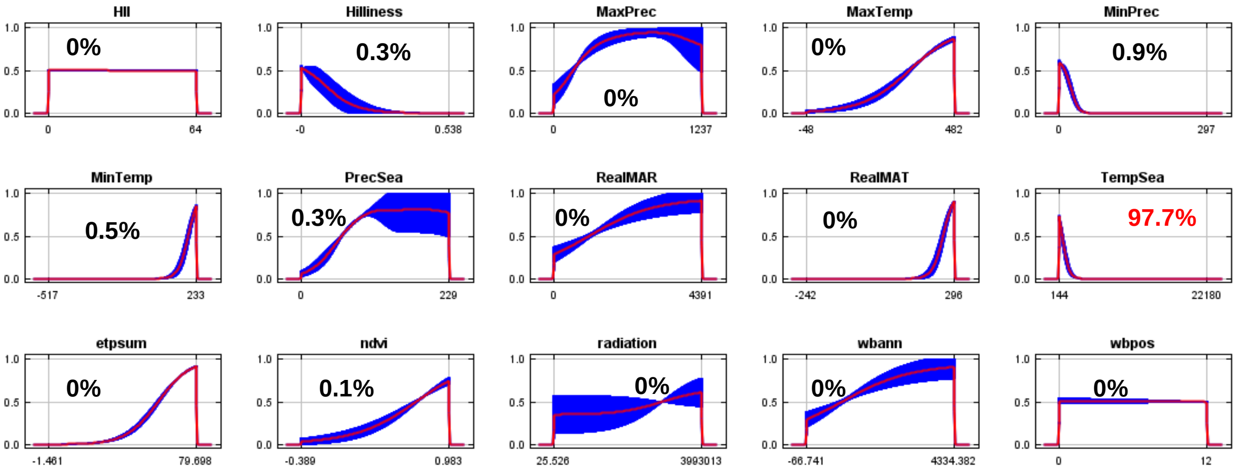

Supplement: S2 Supporting Information — (PDF) [file pone.0122267.s002.pdf]
