# Supplementary material for: Expertly Validated Models and Phylogenetically-Controlled Analysis Suggests Responses to Climate Change Are Related to Species Traits in the Order Lagomorpha
Source: PLoS One. 2015 Apr 15;10(4):e0122267. doi: 10.1371/journal.pone.0122267 (PMC4398435; doi:10.1371/journal.pone.0122267)
Supplement: S3 Supporting Information — Fig. A. Framework for assessing whether species were “modellable” or “unmodellable” based on Kappa values and expert evaluation classification. Fig. B. Trait values for all three lagomorph groups (rabbits, hares & jackrabbits, and pikas). Fig. C. Full lagomorph phylogeny used in phylogenetically controlled regressions. Fig. D. Percentage change in predicted lagomorph species richness from the 1930s to 2080s. Table B. Results of generalised least square models characterising predicted lagomorph bioclimatic envelope changes. Table C. Results of phylogenetically-controlled generalised least square regressions. (PDF) [file pone.0122267.s003.pdf]

S3 Supporting Information.

Table A. Lagomorph experts, institutions and species evaluated.

Expert	Institution	Species evaluated
Alejandro Velazquez	UNAM-Canada	*Lepus californicus*<br>*Sylvilagus insonus*
Andrew Smith	Arizona State University	*Ochotona argentata*<br>*Ochotona curzoniae*<br>*Ochotona dauurica*<br>*Ochotona erythrotis*<br>*Ochotona forresti*<br>*Ochotona gloveri*<br>*Ochotona iliensis*<br>*Ochotona koslowi*<br>*Ochotona ladacensis*<br>*Ochotona princeps*<br>*Ochotona pusilla*<br>*Ochotona thomasi*
Andrew Tilker	University of Texas Austin	*Nesolagus timminsi*
Andrey Lissovsky	Zoological Museum of Moscow State University	*Ochotona hoffmanni*<br>*Ochotona pallasi*<br>*Ochotona rutile*<br>*Ochotona turuchanensis*
Arturo Carillo-Reyes	Universidad de Ciencias y Artes de Chiapas	*Lepus flavigularis*
Bob McCleery	University of Florida	*Sylvilagus palustris*
Charles Krebs	University of British Colombia	*Lepus americanus*
Chelmala Srinivasulu	Osmania University, India	*Lepus tibetanus*<br>*Lepus tolai*<br>*Ochotona rufescens*
Consuelo Lorenzo	Departamento Conservación de la Biodiversidad, Chiapas	*Sylvilagus audubonii*<br>*Sylvilagus bachmani*<br>*Sylvilagus graysoni*
Dana Lee	Oklahoma State University	*Sylvilagus robustus*
Daniel Lew	Venezuelan Institute of Scientific Research, Ecology Centre, Biodiversity Unit	*Sylvilagus varynaensis*
David Gray	Grayhound Information Services	*Lepus arcticus*
David Happold	Australian National University	*Poelagus marjorita*<br>*Pronolagus saundersiae*
Deyan Ge	Institute of *Zoology*, Chinese Academy of Sciences	*Lepus mandschuricus*<br>*Ochotona thibetana*
Eric Waltari	City University of New York	*Lepus othus*<br>*Lepus townsendii*
Francesco Angelici	Italian Foundation of Vertebrate Zoology	*Lepus corsicanus*
Fumio Yamada	Forestry and Forest Products Research Institute, Japan	*Pentalagus furnessi*
Gopinathan Maheswaran	Zoological Survey of India	*Caprolagus hispidus*<br>*Lepus nigricollis*
Hariyo Wibisono	Wildlife Conservation Society, Indonesia	*Nesolagus netscheri*
Hayley Lanier	University of Michigan	*Ochotona collaris*
Jan Schipper	Arizona State University	*Sylvilagus dicei*
Jennifer Frey	New Mexico State University	*Lepus callotis*<br>*Sylvilagus cognatus*<br>*Sylvilagus nuttallii*
John Flux	IUCN Lagomorph Specialist Group	*Lepus capensis*<br>*Lepus microtis*
John Litvaitis	University of New Hampshire	*Sylvilagus transitionalis*
Jorge Salazar-Bravo	Texas Tech University	*Sylvilagus brasiliensis*

Jorge Vazquez	Laboratorio de Ecología del Comportamiento, UAT-UNAM	*Sylvilagus cunicularius*<br>*Sylvilagus floridanus*
Jose Antonio Martinez-Garcia	Universidad Autónoma Metropolitana, Mexico	*Romerolagus diazi*
Julia Witczuk	Warsaw Agricultural University, Poland	*Ochotona hyperborea*
Kai Collins	University of Pretoria	*Bunolagus monticularis*<br>*Lepus saxatilis*<br>*Pronolagus crassicaudatus*<br>*Pronolagus randensis*<br>*Pronolagus rupestris*
Koji Shimano	Shinshu University, Japan	*Lepus brachyurus*
Michael Barbour	Alabama Natural Heritage Program	*Sylvilagus obscurus*
Neil Reid	Queen's University Belfast	*Lepus europaeus*<br>*Lepus timidus*<br>*Oryctolagus cuniculus*
Nishma Dahal	National Centre for Biological Sciences, India	*Ochotona macrotis*<br>*Ochotona nubrica*
Paul Krausman	University of Montana	*Lepus alleni*
Pelayo Acevedo	University of Porto	*Lepus castroviejoi*<br>*Lepus granatensis*
Penny Becker	Washington Dept. of Fish & Wildlife, USA	*Brachylagus idahoensis*
Robert Kissell	Memphis State University	*Sylvilagus aquaticus*
Rudy Boonstra	University of Toronto Scarborough	*Lepus americanus*
Sabuj Bhattacharya	Wildlife Institute of India	*Ochotona roylei*
Sumiya Ganzorig	Hokkaido University	*Ochotona alpina*
Tamara Rioja Pardela	Universidad de Ciencias y Artes de Chiapas, Mexico	*Lepus insularis*<br>*Sylvilagus mansuetus*
Thomas Gray	WWF Greater Mekong	*Lepus peguensis*
Weihe Yang	Institute of Zoology, Chinese Academy of Sciences	*Lepus comus*<br>*Lepus coreanus*<br>*Lepus oiostolus*<br>*Lepus sinensis*<br>*Lepus yarkandensis*
Youhua Chen	Wuhan University, China	*Lepus hainanus*
Zelalem Tolesa	Addis Ababa University	*Lepus fagani*<br>*Lepus habessinicus*<br>*Lepus starcki*

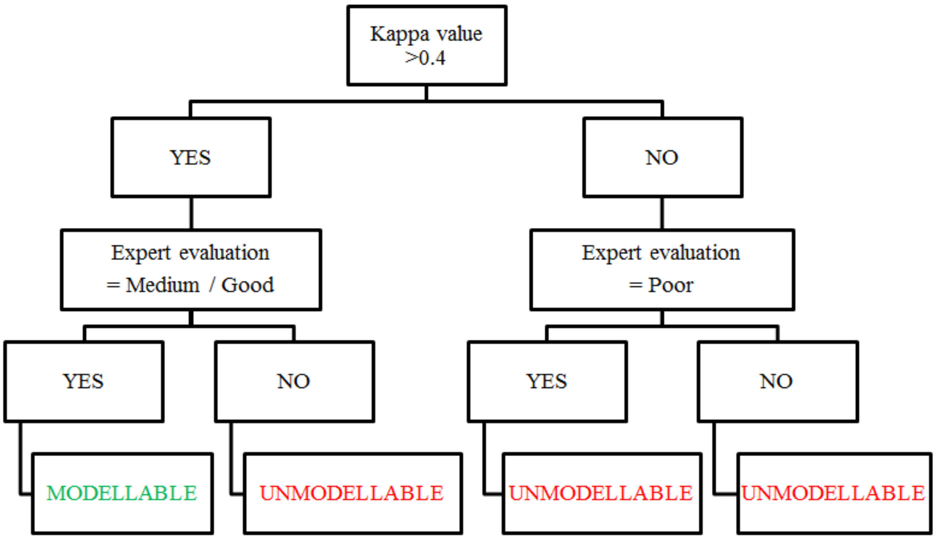

Fig. A. Framework for assessing whether species were "modellable" or "unmodellable" based on Kappa values and expert evaluation classification.

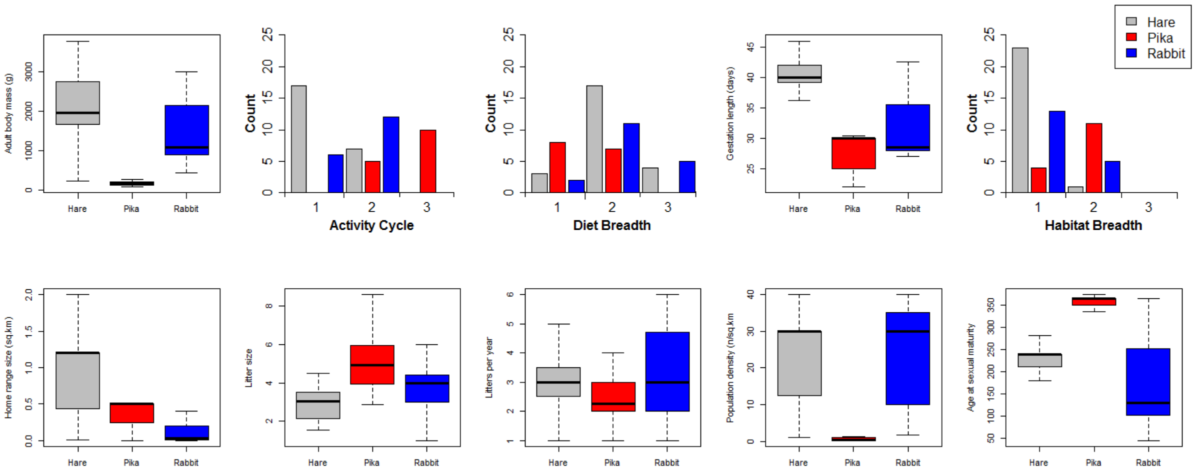

Fig. B. Trait values for all three lagomorph groups (rabbits, hares & jackrabbits, and pikas). Pikas are shown in red, rabbits in blue and hares and jackrabbits in grey. Activity cycle, diet breadth and habitat breadth are categorical variables and are therefore represented as bar plots.

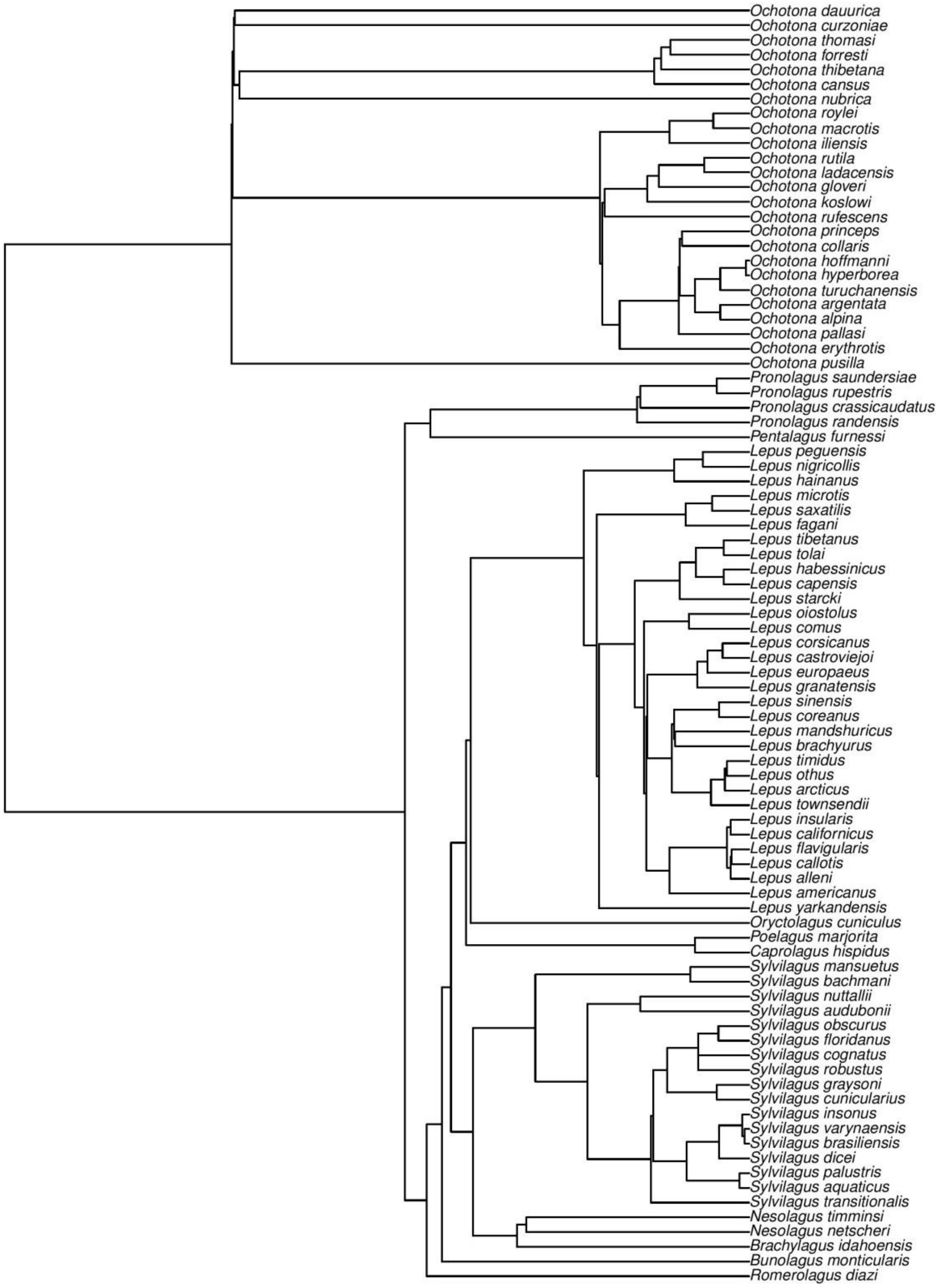

Fig. C. Full lagomorph phylogeny used in phylogenetically controlled regressions.

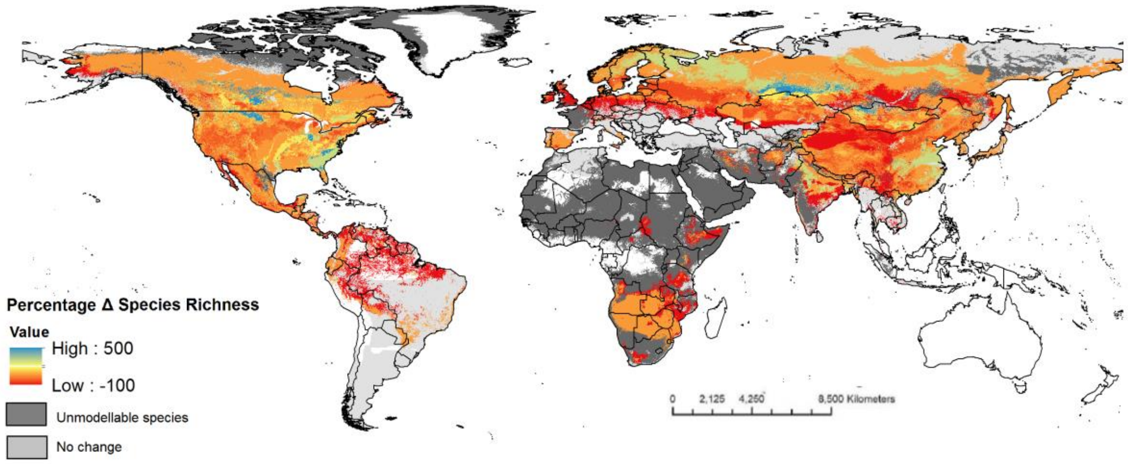

Fig. D. Percentage change in predicted lagomorph species richness from the 1930s to 2080s. Light grey areas indicate no change in species richness and dark grey indicates areas occupied by “unmodellable” species with uncertain outcomes.

Table B. Results of generalised least square models characterising predicted lagomorph bioclimatic envelope changes. Significant *p* values are in bold. Group refers to lagomorph taxonomy, i.e. pikas, rabbits and hares & jackrabbits.

Response variable	Term	*F*	*df*	*p*
Range change (km)	Group	0.004	2, 229	0.950
$F_{df=1,234}$=0.586, *p*=0.445	Year	3.710	1, 228	**0.026**
	Group: Year	0.537	2, 226	0.585
Mean latitudinal change (^{o})	Group	12.798	2, 229	**<0.001**
$F_{df=1,234}$=13.460, *p*<0.001	Year	1.603	1, 228	0.204
	Group: Year	0.448	2, 226	0.640
Mean elevation change (m)	Group	19.458	2, 229	**<0.001**
$F_{df=1,234}$=44.184, *p*<0.001	Year	21.140	1, 228	**<0.001**
	Group: Year	3.541	2, 226	**0.031**

Table C. Results of phylogenetically-controlled generalised least square regressions. Significant *p* values for model-averaged coefficients are in bold. *F* and *p* values for the top model are listed under each response variables; asterisks (*) indicate traits in the top model. Lambda (λ) confidence intervals and significance from 0 and 1 are also shown.

**Response variable**	**Trait**	***β* ± s.e.**	***F***	***p***
Range change (km)	Adult body mass (g)*	0.258 ± 0.112	2.308	**0.021**
$F_{df=4, 52}$=4.28, *p*=0.005	Diet breadth	0.137 ± 0.088	1.552	0.121
	Gestation length (days)	-0.154 ± 0.121	1.269	0.204
Lambda 95%CI=0, 0.147	Litters per year	0.090 ± 0.084	1.073	0.283
N.S. from 0, *p*<0.01 from 1	Litter size	-0.062 ± 0.098	0.634	0.526
	Home range size (km^2)	-0.069 ± 0.099	0.698	0.485
	Population density (n/km^2)	-0.079 ± 0.090	0.878	0.380
	Age at sexual maturity	-0.062 ± 0.089	0.694	0.488
	Activity cycle	0.020 ± 0.134	0.151	0.880
	Habitat breadth	0.050 ± 0.101	0.493	0.622
Mean latitudinal change (^{o})	Adult body mass (g)*	0.196 ± 0.099	1.989	**0.047**
$F_{df=5, 49}$=6.10, *p*<0.001	Diet breadth*	0.181 ± 0.082	2.190	**0.029**
	Litter size*	0.128 ± 0.097	1.320	0.187
Lambda 95%CI=0, 0.209	Litters per year*	0.215 ± 0.079	2.731	**0.006**
N.S. from 0, *p*<0.01 from 1	Activity cycle	0.097 ± 0.124	0.787	0.431
	Age at sexual maturity	-0.106 ± 0.084	1.254	0.210
	Home range size (km^2)	-0.088 ± 0.113	0.787	0.438
	Gestation length (days)	0.147 ± 0.132	1.112	0.266
	Habitat breadth	0.028 ± 0.093	0.304	0.761
	Population density (n/km^2)	0.027 ± 0.084	0.318	0.750
Mean elevation change (m)	Adult body mass (g)*	-0.183 ± 0.091	2.019	**0.043**
$F_{df=2, 50}$=5.92, *p*=0.005	Gestation length (days)	0.099 ± 0.106	0.932	0.351
	Diet breadth	-0.110 ± 0.079	1.386	0.166
Lambda 95%CI=0, 0.205	Home range size (km^2)	0.074 ± 0.075	0.986	0.324
N.S. from 0, *p*<0.01 from 1	Litters per year	-0.057 ± 0.070	0.813	0.416
	Age at sexual maturity	0.055 ± 0.075	0.731	0.324
	Activity cycle	0.075 ± 0.105	0.708	0.479
	Population density (n/km^2)	0.064 ± 0.071	0.902	0.367
	Litter size	0.019 ± 0.079	0.235	0.814
	Habitat breadth	-0.005 ± 0.085	0.058	0.953
Max. elevation change (m)	Litters per year *	0.160 ± 0.092	1.746	0.081
$F_{df=2, 53}$=3.54, *p*=0.036	Gestation length (days)	-0.136 ± 0.116	1.169	0.242
	Habitat breadth	0.095 ± 0.102	0.934	0.351
Lambda 95%CI=0, 0.384	Age at sexual maturity	-0.098 ± 0.096	1.020	0.308
N.S. from 0, *p*<0.01 from 1	Activity cycle	0.041 ± 0.118	0.348	0.728
	Litter size	0.014 ± 0.104	0.137	0.891
	Population density (n/km^2)	0.054 ± 0.091	0.596	0.551
	Adult body mass (g)	0.034 ± 0.121	0.280	0.780
	Home range size (km^2)	-0.003 ± 0.107	0.029	0.977
	Diet breadth	0.004 ± 0.093	0.047	0.962
